# Global Stability of Minkowski Space for the Einstein–Vlasov System in the Harmonic Gauge

**DOI:** 10.1007/s00205-019-01425-1

**Published:** 2019-07-24

**Authors:** Hans Lindblad, Martin Taylor

**Affiliations:** 1 Department of Mathematics, Johns Hopkins University, 3400 N. Charles Street, Baltimore, MD 21218 USA; 2 Department of Mathematics, Imperial College London, South Kensington Campus, London, SW7 2AZ UK

## Abstract

Minkowski space is shown to be globally stable as a solution to the massive Einstein–Vlasov system. The proof is based on a harmonic gauge in which the equations reduce to a system of quasilinear wave equations for the metric, satisfying the weak null condition, coupled to a transport equation for the Vlasov particle distribution function. Central to the proof is a collection of vector fields used to control the particle distribution function, a function of both spacetime and momentum variables. The vector fields are derived using a general procedure, are adapted to the geometry of the solution and reduce to the generators of the symmetries of Minkowski space when restricted to acting on spacetime functions. Moreover, when specialising to the case of vacuum, the proof provides a simplification of previous stability works.

## Introduction

### The Einstein–Vlasov System

The Einstein–Vlasov system provides a statistical description of a collection of collisionless particles, interacting via gravity as described by Einstein’s general theory of relativity. A fundamental problem is to understand the long time dynamics of solutions of this system. The problem is a great challenge even in the absence of particles, and global works on the *vacuum Einstein equations* all either involve simplifying symmetry assumptions or solutions arising from small data. In the asymptotically flat setting, small data solutions of the vacuum Einstein equations were first shown to exist globally and disperse to Minkowski space in the monumental work of Christodoulou–Klainerman [14]. An alternative proof of the stability of Minkowski space was later given by Lindblad–Rodnianski [37] in which a global *harmonic coordinate* system was constructed, described below. For the Einstein–Vlasov system, the global properties of small data solutions were first understood when the initial data were assumed to be spherically symmetric, an assumption under which the equations simplify dramatically, by Rein–Rendall [41] in the *massive case*, when all particles are assumed to have mass 1, and by Dafermos [15] in the *massless case*, when all particles are assumed to have mass 0. See also work of Andréasson–Kunze–Rein [4] for a global existence result in spherical symmetry for a class of large “outgoing” data. Without the simplifying assumption of spherical symmetry, small data solutions of the massless Einstein–Vlasov system were later understood by Taylor [46].

In this work the problem of the stability of Minkowski space for the Einstein–Vlasov system, without any symmetry assumptions, is addressed in the case that all particles have mass 1 (and can easily be adapted to the case that all particles have any fixed mass $m>0$). The system takes the form

1.1 $$\begin{aligned}&Ric(g)_{\mu \nu } - \frac{1}{2} R(g) g_{\mu \nu } = T_{\mu \nu }, \end{aligned}$$

1.2 $$\begin{aligned}&T^{\mu \nu } (t,x) = \int _{\mathcal {P}_{(t,x)}} f(t,x,p) p^{\mu } p^{\nu } \frac{\sqrt{\vert \det g \vert }}{p^0}\,\mathrm{d}p^1 \mathrm{d}p^2 \mathrm{d}p^3,\end{aligned}$$

1.3 $$\begin{aligned}&\mathbf {X}(f) = 0, \end{aligned}$$

where the unknown is a $3+1$ dimensional manifold $\mathcal {M}$ with Lorentzian metric *g*, together with a particle distribution function $f: \mathcal {P}\rightarrow [0,\infty )$, where the *mass shell*$\mathcal {P}$ is defined by

$$\begin{aligned} \mathcal {P}= \{ (t,x,p^0,p) \in T\mathcal {M}\mid (p^0,p) \text { future directed, } g_{\mu \nu }p^{\mu }p^{\nu } = -1 \}. \end{aligned}$$

Here *Ric*(*g*) and *R*(*g*) denote the Ricci and scalar curvature of *g* respectively. A coordinate system (*t*, *x*) for $\mathcal {M}$, with *t* a *time function* (that is the one form *dt* is timelike with respect to the metric *g*), defines a coordinate system $(t,x,p^0,p)$ for the tangent bundle $T\mathcal {M}$ of $\mathcal {M}$*conjugate* to (*t*, *x*), where $(t,x^i,p^0,p^i)$ denotes the point

$$\begin{aligned} p^0 \partial _t \vert _{(t,x)} + p^i \partial _{x^i} \vert _{(t,x)} \in T\mathcal {M}. \end{aligned}$$

The mass shell relation in the definition of $\mathcal {P}$,

1.4 $$\begin{aligned} g_{\mu \nu }p^{\mu }p^{\nu } = -1, \end{aligned}$$

should be viewed as defining $p^0$ as a function of $(t,x^1,x^2,x^3,p^1,p^2,p^3)$. Here Greek indices run over 0, 1, 2, 3, lower case Latin indices run over 1, 2, 3, and often the notation $t=x^0$ is used. The vector $\mathbf {X}$ is the generator of the geodesic flow of $\mathcal {M}$ which, with respect to the coordinate system (*t*, *x*, *p*) for $\mathcal {P}$, takes the form

1.5 $$\begin{aligned} \mathbf {X}= p^{\mu } \partial _{x^{\mu }} - p^{\alpha } p^{\beta } \Gamma _{\alpha \beta }^{i} \partial _{p^{i}}. \end{aligned}$$

The volume form $(\sqrt{\vert \det g \vert }/{p^0})\,\mathrm{d}p^1 \mathrm{d}p^2 \mathrm{d}p^3$ in (1.2) is the induced volume form of the spacelike hypersurface $\mathcal {P}_{(t,x)} \!\subset \! T_{(t,x)}\mathcal {M}$ when the tangent space $T_{(t,x)} \mathcal {M}$ is endowed with the metric $g_{\mu \nu }(t,x)\,\mathrm{d}p^{\mu } \mathrm{d}p^{\nu }$ induced by *g* on $\mathcal {M}$.

### The Global Existence Theorem

#### The Initial Value Problem and the Global Existence Theorem

In the Cauchy problem for the system (1.1)–(1.3) one prescribes an *initial data set*, which consists of a Riemannian 3 manifold $(\Sigma , \overline{g})$ together with a symmetric (0, 2) tensor *k* on $\Sigma$, and an initial particle distribution $f_0$, satisfying the *constraint equations*

$$\begin{aligned} \overline{\mathrm {div}} k_j - (d \overline{\text {tr}} k)_j = T_{0j}, \qquad \overline{R} + (\overline{\text {tr}} k)^2 - \vert k\vert ^2_{\overline{g}} = 2 T_{00} \end{aligned}$$

for $j=1,2,3$. Here $\overline{\mathrm {div}}$, $\overline{\text {tr}}$, $\overline{R}$ denote the divergence, trace and scalar curvature of $\overline{g}$ respectively, and $T_{00}, T_{0j}$ denote (what will become) the 00 and 0*j* components of the energy momentum tensor. The topology of $\Sigma$ will here always be assumed to be that of $\mathbb {R}^3$. A theorem of Choquet-Bruhat [10], based on previous work by Choquet-Bruhat [9] and Choquet-Bruhat–Geroch [12] on the vacuum Einstein equations, (see also the recent textbook of Ringström [42]) guarantees that, for any initial data set as above, there exists a globally hyperbolic solution $(\mathcal {M},g,f)$ of the system (1.1)–(1.3) which attains the initial data, in the sense that there exists an imbedding $\iota : \Sigma \rightarrow \mathcal {M}$ under the pullback of which the induced first and second fundamental form of *g* are $\overline{g}$ and *k* respectively, and the restriction of *f* to the mass shell over $\iota (\Sigma )$ is given by $f_0$. As in [37], the proof is based on the *harmonic gauge* (or *wave gauge*), that is a system of coordinates $x^{\mu }$ satisfying

1.6 $$\begin{aligned} \square _g x^{\mu } = 0, \end{aligned}$$

where $\square _g$ denotes the geometric wave operator of the metric *g*.

The initial data set

1.7 $$\begin{aligned} \left( \mathbb {R}^3,e, k \equiv 0, f_0 \equiv 0\right) , \end{aligned}$$

where *e* denotes the Euclidean metric on $\mathbb {R}^3$, satisfies the constraint equations and gives rise to Minkowski space, the trivial solution of the system (1.1)–(1.3). The main result of this work concerns solutions arising from initial data sufficiently close to the trivial initial data set (1.7). The initial data will be assumed to be *asymptotically flat* in the sense that $\Sigma$ is diffeomorphic to $\mathbb {R}^3$ and there exists a global coordinate chart $(x^1,x^2,x^3)$ of $\Sigma$ and $M \ge 0$ and $0<\gamma <1$ such that

1.8 $$\begin{aligned} \overline{g}_{ij} = \left( 1 + \frac{M}{r} \right) \delta _{ij} + o(r^{-1-\gamma }), \qquad k_{ij} = o(r^{-2-\gamma }), \qquad \text {as } r = \vert x \vert \rightarrow \infty . \end{aligned}$$

For such $\overline{g}$, *k*, write

$$\begin{aligned} \overline{g}_{ij} = \delta _{ij} + \overline{h}^0_{ij} + \overline{h}^1_{ij},\quad \text {where}\quad \overline{h}^0_{ij}(x) = \chi (r) \delta _{ij}M/r, \end{aligned}$$

and $\chi$ is a smooth cut off function such that $0 \le \chi \le 1$, $\chi (s) = 1$ for $s \ge {3}/{4}$ and $\chi (s) = 0$ for $s \le {1}/{2}$.

For given $\gamma >0$ and such an initial data set, define, for any $N\ge 0$, the initial energy

1.9 $$\begin{aligned} \mathcal {E}_N = {\sum }_{\vert I \vert \le N} \left( \Vert (1+r)^{{1}/{2} + \gamma + \vert I \vert } \nabla \nabla ^I \overline{h}^1 \Vert _{L^2(\Sigma )} + \Vert (1+r)^{{1}/{2} + \gamma + \vert I \vert } \nabla ^I k \Vert _{L^2(\Sigma )} \right) , \end{aligned}$$

where $\nabla = (\partial _{x^1},\partial _{x^2}, \partial _{x^3})$ denotes the coordinate gradient, and the initial norms for $f_0$:

1.10 $$\begin{aligned} \mathbb {D}_N = {\sum }_{k + \ell \le N} \Vert \partial _{x}^{k} \partial _{p}^{\ell } f_0 \Vert _{L^\infty }, \qquad \mathcal {V}_N = {\sum }_{k + \ell \le N} \Vert \partial _{x}^{k} \partial _{p}^{\ell } f_0 \Vert _{L^2_x L^2_p}. \end{aligned}$$

By the Sobolev inequality there exists a constant *C* such that $\mathbb {D}_{N-4} \le C \mathcal {V}_N$ for any $N \ge 0$.

The main result of this work is the following:

##### Theorem 1.1

Let $(\Sigma , \overline{g}, k,f_0)$ be an initial data set for the Einstein–Vlasov system (1.1)–(1.3), asymptotically flat in the above sense, such that $f_0$ is compactly supported. For any $0<\gamma <1$ and $N \ge 11$, there exists $\varepsilon _0>0$ (depending on the size of $\mathrm {supp}(f_0)$) such that, for all $\varepsilon \le \varepsilon _0$ and all initial data satisfying

$$\begin{aligned} \mathcal {E}_N + \mathcal {V}_N + M \le \varepsilon , \end{aligned}$$

there exists a unique future geodesically complete solution of (1.1)–(1.3), attaining the given data, together with a global system of harmonic coordinates $(t,x^1,x^2,x^3)$, relative to which the solution asymptotically decays to Minkowski space.

The precise sense in which the spacetimes of Theorem 1.1 asymptotically decay to Minkowski space is captured in the estimates (1.15), (1.16), (6.5), (6.6), (6.8), (6.9), (6.10) and (6.11) below.

The analogue of Theorem 1.1 for the massless Einstein–Vlasov system (which is the system (1.1)–(1.3) but with the mass shell relation (1.4) replaced with the relation $g_{\mu \nu } p^{\mu } p^{\nu } = 0$, so that all particles travel through spacetime along null geodesics, as opposed to unit timelike geodesics in the massive case considered here) was resolved by Taylor [46]. It should be noted that the difficulties encountered in [46] are very different to the difficulties encountered in the present work. The work [46] involves a *double null gauge* (in contrast to the harmonic gauge employed here) and, as is shown as part of the bootstrap argument in the proof, *f*, being initially supported in a spatially compact set, is supported only in the *wave zone*$\vert x \vert \sim t$ in a region of finite retarded length. Due to the slow decay of the metric in the wave zone, the work [46] relies crucially in exploiting a *null structure*^1^ present in the Vlasov equation.^2^ In contrast, the main difficulties in the present work arise in the *interior region*$\vert x \vert < t$. The “Minkowski vector fields” (see Section 1.5.1 below), which one would use to control the decoupled Vlasov equation on a fixed Minkowski background, are insufficient by themselves for the proof of Theorem 1.1 and have to be further adapted to the geometry of the spacetimes considered. See Section 1.5 below for a further discussion of the vector fields used in the proof.

The assumption in Theorem 1.1 that $f_0$ is compactly supported is made for simplicity and, we believe, our method can be extended to relax this assumption. The assumption implies that the solution *f* is supported, at late times, away from the wave zone $\vert x \vert \sim t$. The issue of the slow decay of the metric *g* in the wave zone is therefore not relevant in the proof of Theorem 1.1 when controlling the solution of the Vlasov equation *f*, and its derivatives. Were $f_0$ not compactly supported, this slow decay of *g* would be relevant and it would be necessary to exploit a form of null structure present in the Vlasov equation, as in the *massless case* discussed above, together with the weak null structure of Einstein’s equations in wave coordinates.

A similar stability result to Theorem 1.1 was shown independently by Fajman–Joudioux–Smulevici [19]. We remark on some of the differences between the two proofs in Section 1.5 after presenting our argument.

There have been a number of related stability works on the Einstein–Vlasov system. In addition to those discussed earlier, there has been related work by Ringström [42] on the Einstein–Vlasov system in the presence of a positive cosmological constant, where the analogue of the Minkowski solution is the de Sitter spacetime. See also [5]. A stability result for a class of cosmological spacetimes was shown in $2+1$ dimensions with vanishing cosmological constant by Fajman [16], and later extended to $3+1$ dimensions by Andersson–Fajman [2]. See also the recent work of Moschidis [39] on the instability of the anti-de Sitter spacetime for a related spherically symmetric model in the presence of a negative cosmological constant. A much more comprehensive overview of work on the Einstein–Vlasov system can be found in the review paper of Andréasson [3].

There has also been work on the problem of the stability of Minkowski space for the Einstein equations coupled to various other matter models [7, 20, 22, 28–30, 43, 48].

Small data solutions of the Vlasov–Poisson system, the non-relativistic analogue of the Einstein–Vlasov system, were studied in [6, 23], and those of the Vlasov–Maxwell system in [21]. We note in particular works of Smulevici [44] and Fajman–Joudioux–Smulevici [18] (following [17]) on the asymptotic properties of small data solutions of the Vlasov–Poisson and the related Vlasov–Nordström systems respectively, where issues related to those described in Section 1.5.2 arise and are resolved using an alternative approach. Moreover, there are global existence results for general data for Vlasov–Poisson [38, 40] and Vlasov–Nordström [8].

#### Small Data Global Existence for the Reduced Einstein–Vlasov System

Following [37], the proof of Theorem 1.1 is based on a harmonic gauge (1.6), relative to which the Einstein equations^3^ (1.1) take the form of a system of quasilinear wave equations

1.11 $$\begin{aligned} \widetilde{\square }_g \, g_{\mu \nu } = F_{\mu \nu } (g)(\partial g, \partial g)+\widehat{T}_{\mu \nu } , \quad \text {where} \quad \widetilde{\square }_g=g^{\alpha \beta }\partial _\alpha \partial _\beta , \end{aligned}$$

where $\widehat{T}_{\mu \nu } := T_{\mu \nu } - \frac{1}{2}g_{\mu \nu }{\text {tr}}_g T$ and $F_{\mu \nu }(u)(v,v)$ depends quadratically on *v*. The system (1.2), (1.3), (1.11) is known as the *reduced Einstein–Vlasov system*. The condition that the coordinates $x^{\mu }$ satisfy the harmonic gauge condition (1.6) is equivalent to the metric in the coordinates $x^{\mu }$ satisfying the *wave coordinate condition*

1.12 $$\begin{aligned} g^{\alpha \beta } \partial _{\alpha } g_{\beta \mu } = \frac{1}{2} g^{\alpha \beta } \partial _{\mu } g_{\alpha \beta }, \quad \text {for}\quad \mu = 0,1,2,3. \end{aligned}$$

Let $m = \text {diag}\,(-1,1,1,1)$ denote the Minkowski metric in Cartesian coordinates and, for a solution *g* of the reduced Einstein equations (1.11), write

1.13 $$\begin{aligned} g = m + h^0 + h^1, \quad \text {where} \quad h^0_{\mu \nu }(t,x) ={\chi }\big (\tfrac{r}{1+t}\big ) \frac{M}{r} \delta _{\mu \nu }, \end{aligned}$$

where $\chi (s)=1$, when $s>3/4$ and $\chi (s)=0$, when $s<1/2$. For a given $0< \gamma < 1$, $0<\mu < 1-\gamma$, we define the energy at time *t* as

1.14 $$\begin{aligned}&E_N(t) = \sum _{\vert I \vert \le N} \Vert w^{\frac{1}{2}} \partial Z^I h^1 (t,\cdot ) \Vert _{L^2}^2,\nonumber \\&\quad \text {where} \quad w(t,x) = {\left\{ \begin{array}{ll} (1+|r-t|)^{1+2\gamma },\,\,\, &{} r>t\\ 1+(1+|r-t|)^{-2\mu }, \quad &{} r\le t. \end{array}\right. } \end{aligned}$$

Here *I* denotes a multi index and $Z^I$ denotes a combination of |*I*| of the vector fields

$$\begin{aligned} \Omega _{ij} = x^i \partial _{x^j} - x^j \partial _{x^i}, \quad B_{i} = x^i \partial _t + t \partial _{x^i}, \quad S = t\partial _t + x^k \partial _{x^k},\quad \text {and}\quad \partial _\alpha , \end{aligned}$$

for $i,j=1,2,3$ and $\alpha =0,1,2,3$. Let $\vert \cdot \vert$ denote the norm, $\vert x \vert = \big ( (x^1)^2 + (x^2)^2 + (x^3)^2 \big )^{{1}\!/{2}}$, and $\vert h(t,x) \vert = \sum _{\alpha ,\beta =0}^3 \vert h_{\alpha \beta }(t,x)\vert$, $\left| \Gamma (t,x) \right| = \sum _{\alpha ,\beta ,\gamma =0}^3 \big \vert \Gamma ^{\alpha }_{\beta \gamma } (t,x) \big \vert$, and similarly $\Vert h(t,\cdot ) \Vert _{L^2} = \sum _{\alpha ,\beta =0}^3 \Vert h_{\alpha \beta }(t,\cdot )\Vert _{L^2}$ etc.. The notation $A \lesssim B$ will be used if there exists a universal constant *C* such that $A \le C B$.

Theorem 1.1 follows as a corollary of the following theorem:

##### Theorem 1.2

For any $0<\gamma <1$, $K,K'>0$ and $N\ge 11$, there exists $\varepsilon _0>0$ such that, for any data $(g_{\mu \nu }, \partial _t g_{\mu \nu },f) \vert _{t=0}$ for the reduced Einstein–Vlasov system (1.2), (1.3), (1.11) which satisfy the smallness condition

$$\begin{aligned} E_N(0)^{\frac{1}{2}} + \mathcal {V}_N + M < \varepsilon \end{aligned}$$

for any $\varepsilon \le \varepsilon _0$ and the wave coordinate condition (1.12), and such that for

$$\begin{aligned} \mathrm {supp}(f\vert _{t=0}) \subset \{ \vert x \vert \le K, \vert p \vert \le K' \} \end{aligned}$$

there exists a global solution attaining the data such that

1.15 $$\begin{aligned} \left( E_N(t) \right) ^{\frac{1}{2}} + {\sum }_{\vert I \vert \le N} (1+t) \Vert Z^I T^{\mu \nu }(t,\cdot ) \Vert _{L^2} \le C_N \varepsilon (1+t)^{C_N' \varepsilon } \end{aligned}$$

for all $t\ge 0$, along with the decay estimates

1.16 $$\begin{aligned} |Z^I h^1(t,x)|\le \frac{C_N^\prime \varepsilon (1+t)^{C_N'\varepsilon }}{(1+t+r) (1+q_+)^{\gamma }}, \quad |I|\le N-3,\quad q_+={\left\{ \begin{array}{ll} r-t,\,\,\, &{} r>t\\ 0, \quad &{} r\le t, \end{array}\right. } \end{aligned}$$

and the estimates (6.5), (6.6), (6.8), (6.9), (6.10) and (6.11) stated in Section 6.

Note that the wave coordinate condition (1.12), if satisfied initially, is propagated in time by the reduced Einstein–Vlasov system (1.2), (1.3), (1.11) (this fact is standard; see, for example, Section 4 of [36], which requires only minor modifications for the presence of matter).

Given an initial data set $(\Sigma ,\overline{g},k,f_0)$ as in Theorem 1.1, define initial data for the reduced equations

$$\begin{aligned} g_{ij} \vert _{t=0} = \overline{g}_{ij}, \quad g_{00} \vert _{t=0} = - a^2, \quad g_{0i} \vert _{t=0} = 0,\quad a(x)^2 = \left( 1 - \chi (r) {M}/{r}\right) , \end{aligned}$$

and

$$\begin{aligned} \partial _t g_{ij} \vert _{t=0}= & {} -2a k_{ij}, \quad \partial _t g_{00} \vert _{t=0} = 2 a^3 \overline{g}^{ij} k_{ij},\\ \partial _t g_{0i} \vert _{t=0}= & {} a^2 \overline{g}^{jk} \partial _j \overline{g}_{ik} - \frac{a^2}{2} \overline{g}^{jk} \partial _i \overline{g}_{jk} - a \partial _i a. \end{aligned}$$

One can show that, with this choice, $\left( E_N(0) \right) ^{{1}/{2}} \lesssim \mathcal {E}_N,$ where $\mathcal {E}_N$ given by (1.9) is the norm of geometric data, and moreover $(g_{\mu \nu }, \partial _t g_{\mu \nu }) \vert _{t=0}$ satisfy the wave coordinate condition (1.12), see, for example, [36, 37].

It is therefore clear that Theorem 1.1 follows from Theorem 1.2 (the future causal geodesic completeness can be shown as in [36]) and so the goal of the paper is to establish the proof of Theorem 1.2.

### Estimates for the Vlasov Matter

In what follows it is convenient, instead of parameterising the mass shell $\mathcal {P}$ by (*t*, *x*, *p*), to instead parameterise it by $(t,x,{\,\widehat{\!p\!}\,\,})$, where

$$\begin{aligned} {\,\widehat{\!p\!}\,\,}^i = {p^i}/{p^0} \end{aligned}$$

for $i=1,2,3$. Note that, by the mass shell relation (1.4), in Minkowski space $(p^0)^2 = 1 + (p^1)^2 + (p^2)^2 + (p^3)^2$ and, under a mild smallness condition on $g - m$, $\vert {\,\widehat{\!p\!}\,\,}\vert < 1$. Abusing notation slightly, we will write $f(t,x,{\,\widehat{\!p\!}\,\,})$ for the solution of the Vlasov equation (1.3).

Let $\{ \Sigma _t \}$ denote the level hypersurfaces of the time coordinate *t*, and let $X(s,t,x,{\,\widehat{\!p\!}\,\,})^i$, ${\,\widehat{\!P\!}\,\,}(s,t,x,{\,\widehat{\!p\!}\,\,})^i$ denote solutions of the geodesic equations (see Section 2)

1.17 $$\begin{aligned} \frac{\mathrm{d} X^i}{\mathrm{d}s}(s,t,x,{\,\widehat{\!p\!}\,\,})= & {} {\,\widehat{\!P\!}\,\,}^i(s,t,x,{\,\widehat{\!p\!}\,\,}), \nonumber \\ \frac{\mathrm{d} {\,\widehat{\!P\!}\,\,}^i}{\mathrm{d}s}(s,t,x,{\,\widehat{\!p\!}\,\,})= & {} {\,\widehat{\!\Gamma \!}\,\,}^i (s, X(s,t,x,{\,\widehat{\!p\!}\,\,}), {\,\widehat{\!P\!}\,\,}(s,t,x,{\,\widehat{\!p\!}\,\,})), \end{aligned}$$

normalised so that $(s,X(s,t,x,{\,\widehat{\!p\!}\,\,})) \in \Sigma _s$, with

$$\begin{aligned} X^i(t,t,x,{\,\widehat{\!p\!}\,\,}) = x^i, \quad {\,\widehat{\!P\!}\,\,}^i(t,t,x,{\,\widehat{\!p\!}\,\,}) = {\,\widehat{\!p\!}\,\,}^i. \end{aligned}$$

Here

$$\begin{aligned} {\,\widehat{\!\Gamma \!}\,\,}^{\mu } (t,x,{\,\widehat{\!p\!}\,\,}) = \Gamma _{\alpha \beta }^0(t,x){\,\widehat{\!p\!}\,\,}^{\alpha } {\,\widehat{\!p\!}\,\,}^{\beta } {\,\widehat{\!p\!}\,\,}^{\mu } - \Gamma _{\alpha \beta }^{\mu } (t,x) {\,\widehat{\!p\!}\,\,}^{\alpha }{\,\widehat{\!p\!}\,\,}^{\beta },\quad {\,\widehat{\!p\!}\,\,}^0=1, \end{aligned}$$

where $\Gamma ^{\alpha }_{\beta \gamma }$ are the Christoffel symbols of the metric *g* with respect to a given coordinate chart $(t,x^1,x^2,x^3)$. Define $X(s,t,x,{\,\widehat{\!p\!}\,\,})^0 = s$ and ${\,\widehat{\!P\!}\,\,}(s,t,x,{\,\widehat{\!p\!}\,\,})^0 = 1$. The notation *X*(*s*), ${\,\widehat{\!P\!}\,\,}(s)$ will sometimes be used for $X(s,t,x,{\,\widehat{\!p\!}\,\,})$, ${\,\widehat{\!P\!}\,\,}(s,t,x,{\,\widehat{\!p\!}\,\,})$ when it is clear from the context which point $(t,x,{\,\widehat{\!p\!}\,\,})$ is meant, and the notation ${\,\widehat{\!X\!}\,\,}(s)=(s,X(s))$ will sometimes be used.

It follows that the Vlasov equation (1.3) can be rewritten as

1.18 $$\begin{aligned} f(t,x,{\,\widehat{\!p\!}\,\,}){=} f(s,X(s,t,x,{\,\widehat{\!p\!}\,\,}),{\,\widehat{\!P\!}\,\,}(s,t,x,{\,\widehat{\!p\!}\,\,})){=}f_0(X(0,t,x,{\,\widehat{\!p\!}\,\,}),{\,\widehat{\!P\!}\,\,}(0,t,x,{\,\widehat{\!p\!}\,\,})) \end{aligned}$$

for all *s*. The notation (*y*, *q*) will be used to denote points in the mass shell over the initial hypersurface, $\mathcal {P}\vert _{t=0}$. In Theorem 1.2 it is assumed that $f_0$ has compact support; $|y|\le K$ and $|q|\le K'$ for $(y,q) \in \mathrm {supp}(f_0)$, for some constants *K*, $K'$. Under relatively mild smallness assumptions on $h=g-m$, see Proposition 2.1, it follows that there exists $c<1$, depending only on $K'$, such that solutions of the Vlasov equation satisfy

1.19 $$\begin{aligned} \mathrm {supp}(f) \subset \{ (t,x,p);\, |x|\le K+ct,\, \,|p\,|\le K'+1,\, \,|\widehat{p}\,|\le c\}. \end{aligned}$$

The main new difficulties in the proof of Theorem 1.2, arising from the coupling to the Vlasov equation, are resolved in the following theorem, which is appealed to in the proof of Theorem 1.2:

#### Theorem 1.3

For a given $t\ge 0$ and $N\ge 1$, suppose that g is a Lorentzian metric such that the Christoffel symbols of *g* with respect to a global coordinate system $(t,x^1,x^2,x^3)$, for some ${1}/{2}<a<1$, satisfy

1.20 $$\begin{aligned} \left| Z^I \Gamma (t',x) \right| \le \frac{C_N^\prime \varepsilon }{(1+t')^{1+a}}, \quad \text {for } \vert x \vert \le ct' + K, \quad \vert I \vert \le \left\lfloor \frac{N}{2} \right\rfloor +2 \end{aligned}$$

for all $t'\!\in [0,t]$. Then there exists $\varepsilon _0>0$ such that, for $\varepsilon <\varepsilon _0$ and any solution *f* of the Vlasov equation (1.3) satisfying

$$\begin{aligned} \mathrm {supp}(f) \subset \{ (t,x,p);\, |x|\le K+ct,\, \,|\widehat{p}\,|\le c\}, \end{aligned}$$

and metric satisfying (1.20) and $\vert g -m \vert \le \varepsilon$, the components of the energy momentum tensor $T^{\mu \nu } (t,x)$ satisfy

$$\begin{aligned}&\Vert \big ( Z^I T^{\mu \nu } \big ) (t,\cdot ) \Vert _{L^{1}} \le D_k\mathcal {V}_{k } \\&\quad +D_k\mathbb {D}_{k^\prime } \Bigg ( \sum _{\vert J \vert \le \vert I \vert -1} \frac{\Vert ( Z^J \Gamma )(t,\cdot ) \Vert _{L^2}}{(1+t)^{a-1\!/2}} + \sum _{\vert J \vert \le \vert I \vert + 1} \int _{0}^t \frac{\Vert ( Z^J \Gamma )(s,\cdot ) \Vert _{L^2}}{(1+s)^{{1}\!/{2}+a}}\, \mathrm{d}s \Bigg ) \end{aligned}$$

for $\vert I \vert \le N-1$, where $k=|I|$ and $k^\prime =\left\lfloor {k }/{2} \right\rfloor +1$, and

$$\begin{aligned} \Vert \big ( Z^I T^{\mu \nu } \big ) (t,\cdot ) \Vert _{L^{2}}\le & {} \frac{D_k\mathcal {V}_{k}}{(1+t)^{{3}/{2}}} +D_k\mathbb {D}_{k^\prime } \Bigg (\sum _{\vert J \vert \le \vert I \vert -1} \frac{\Vert ( Z^J \Gamma )(t,\cdot ) \Vert _{L^2}}{(1+t)^{1+a}}\nonumber \\&\quad + \sum _{\vert J \vert \le \vert I \vert } \frac{1}{(1+t)^{{3}/{2}}} \int _{0}^t \frac{\Vert ( Z^J \Gamma )(s,\cdot ) \Vert _{L^2}}{(1+s)^{{1}\!/{2}}}\, \mathrm{d}s \Bigg ) \end{aligned}$$

for $\vert I \vert \le N$. Here the constants $D_k$ depend only on $C_N'$, *K*, $K'$ and *c*, and $\varepsilon _0$ depends only on *c*.

In the proof of Theorem 1.2, the $L^2$ estimates of Theorem 1.3 are used to prove energy estimates for the metric *g*; see the discussion in Section 1.4.2. The $L^1$ estimates of Theorem 1.3 are used to recover the pointwise decay (1.20) for the lower order derivatives of the Christoffel symbols $\Gamma$; see the discussion in Section 1.4.7 below.

#### Remark 1.4

The proof of Theorem 1.3 still applies when $a \ge 1$, though the theorem is only used in the proof of Theorem 1.2 for some fixed $\frac{1}{2}< a< 1$. The case of $a = 1$ is omitted in order to avoid logarithmic factors. The proof of an appropriate result when $a > 1$ is much simpler, although, when Theorem 1.3 is used in the proof of Theorem 1.2, one could not hope for the assumptions (1.20) to hold with $a > 1$, see Section 1.5.2.

#### Remark 1.5

In Section 5 a better $L^2$ estimate in terms of *t* behaviour, compared with the $L^2$ estimate of Theorem 1.3, is shown to hold for $Z^I T^{\mu \nu }$, which involves one extra derivative of $\Gamma$; see Proposition 5.8. It is important however to use the $L^2$ estimate which does not lose a derivative in the proof of Theorem 1.2.

### Overview of the Global Existence Theorem for the Reduced Einstein Equations

It should be noted from the outset that the reduced Einstein–Vlasov system (1.2), (1.3), (1.11) is a system of quasilinear wave equations coupled to a transport equation. It is well known that the general quasilinear wave equation does not necessarily admit global solutions for all small data [24, 25]. The *null condition*, an algebraic condition on the nonlinearity of such equations, was introduced by Klainerman [26], and used independently by Klainerman [27] and Christodoulou [13], as a sufficient condition that small data solutions exist globally in time and are asymptotically free. However, as was noticed by Lindblad [32, 33] and Alinhac [1], there are quasilinear equations that do not satisfy the null condition but still admit global solutions for all sufficiently small data. In fact, the classical null condition fails to be satisfied by the vacuum Einstein equations in the harmonic gauge ((1.11) with $T\equiv 0$), though it was noticed by Lindblad–Rodnianski [35] that they satisfy a *weak null condition*, which they used to prove a small data global existence theorem [36, 37].

The proof of Theorem 1.2 follows the strategy adopted in [37]. The new difficulties, of course, arise from the coupling to the Vlasov equation. A fundamental feature of the problem arises from the fact that, whilst the slowest decay of solutions to wave equations occurs in the *wave zone*, where $t \sim r$, the slowest decay of solutions of the massive Vlasov equation occurs in the interior region $t>r$. The most direct way to exploit this fact is to impose that $f_0$ has compact support, in which case, as will be shown in Proposition 2.1, the support condition (1.19) holds and *f* actually vanishes in the wave zone at late times.

Since they have been described at length elsewhere, the difficulties associated to the failure of (1.11) to satisfy the classical null condition are only briefly discussed here. Suffice it to say that there is a rich structure in the equations (1.11) which is exploited heavily (see the further discussion in the introductions to [36, 37]). The main new features of this work are contained in the proof of Theorem 1.3. Indeed, for a given inhomogeneous term $\widehat{T}$ in (1.11) which satisfies the support conditions and estimates of Theorem 1.3, the small data global existence theorem of [37] mostly goes through unchanged. An outline is given below, including a discussion of some observations which lead to simplifications compared with the proof in [37] (most notably the fact that the structure of the equations are better preserved under commuation with modified Lie derivatives, described in Section 1.4.4 below, but also the use of a simpler $L^{\infty }$–$L^{\infty }$ estimate, described in Section 1.4.6).

The proof of Theorem 1.2 is based on a continuity argument. One assumes that the bounds

1.21 $$\begin{aligned} E_N(t)^{\frac{1}{2}} \le C_N \varepsilon (1+t)^{\delta }, \quad {\sum }_{\vert I \vert \le N-1} \Vert Z^I T^{\mu \nu } (t,\cdot ) \Vert _{L^1} \le C_N \varepsilon \end{aligned}$$

hold for all $t\in [0,T_*]$ for some time $T_*>0$ and some fixed constants $C_N$ and $\delta$, and the main objective is to use the Einstein equations to prove that the bounds (1.21) in fact hold with better constants, provided the initial data are sufficiently small.

#### The Contribution of the Mass

The first step in the proof of Theorem 1.2 is to identify the contribution of the mass *M*. Recall the decomposition of the metric (1.13) and note that the energy $E_N$ is defined in terms of $h^1$. Had the energy been defined with $h=g-m$ in place of $h^1$, it would not be finite unless $M=0$, in which case it follows from the Positive Mass Theorem [45, 47] that the constraint equations imply that the solution is trivial. The contribution of the mass is therefore identified explicitly using the decomposition (1.13), and the reduced Einstein equations are recast as a system of equations for $h^1$:

1.22 $$\begin{aligned} \widetilde{\square }_g \, h^1_{\mu \nu } = F_{\mu \nu } (h)(\partial h, \partial h)+\widehat{T}_{\mu \nu } - \widetilde{\square }_g \, h^0_{\mu \nu }. \end{aligned}$$

The term $\widetilde{\square }_g \, h^0_{\mu \nu }$ is treated as an error term. Note that $h^0$ is defined so that $\square \, h^0_{\mu \nu }$, which is a good approximation to $\widetilde{\square }_g \, h^0_{\mu \nu }$, is supported away from the wave zone $t \sim r$ and so only contributes in the interior region, where $h^1$ will be shown to have fast decay.

#### Energy Inequality with Weights

An important ingredient in the procedure to recover the assumption on the energy (1.21) is the energy inequality with weights,

1.23 $$\begin{aligned}&\int _{\Sigma _{t}} |\partial \phi |^{2}w\, \mathrm{d}x + \!\int _{0}^{t}\!\! \int _{\Sigma _{\tau }} |\overline{\partial }\phi |^{2}\,w^{\,\prime } \,\mathrm{d}x\mathrm{d}\tau \nonumber \\&\quad \le 8\!\int _{\Sigma _{0}}|\partial \phi |^{2}\,w \mathrm{d}x+ \!\int _0^t\!\!\frac{C\varepsilon }{1+\tau }\int _{\Sigma _{\tau }} |\partial \phi |^{2} w \,\mathrm{d}x \, \mathrm{d}\tau \nonumber \\&\qquad +16 \int _0^t\Big (\int _{\Sigma _{\tau }}|\widetilde{\square }_g \phi |^2 w\mathrm{d}x\Big )^{\!1/2}\Big (\int _{\Sigma _{\tau }} |\partial \phi |^2 w \,\mathrm{d}x\Big )^{\!1/2}\!\, \mathrm{d}\tau , \end{aligned}$$

which holds, for any suitably regular function $\phi$, under mild assumptions on the metric *g* (see Lemma 6.29), where the weight *w* is as in (1.14) and $\overline{\partial }_{\mu } = \partial _{\mu } - \frac{1}{2}L_{\mu }(\partial _r - \partial _t)$, with $L = \partial _t + \partial _r$, denotes the derivatives tangential to the outgoing Minkowski light cones. The inequality will be applied to the system (1.22) after commuting with vector fields.

It is in the energy inequality (1.23) that the $L^2$ estimates of Theorem 1.3 are used. The proof of Theorem 1.2, using Theorem 1.3, is given in detail in Section 7, but we briefly illustrate the use of the $L^2$ estimates of Theorem 1.3 here. Setting $Q_N(t) = \sup _{0\le s \le t} \sum _{\vert I \vert \le N} \Vert w^{\frac{1}{2}} \partial Z^I h^1 (s,\cdot ) \Vert _{L^2}$, it follows from Theorem 1.3 that

1.24 $$\begin{aligned} \Vert Z^I {\,\widehat{\!T\!}\,\,} (\tau ,\cdot ) \Vert _{L^2} \le \frac{C\varepsilon }{(1+\tau )^{\frac{3}{2}}} + \frac{C\varepsilon }{(1+\tau )} Q_N(t) \end{aligned}$$

(see Section 7 for details of how estimates for ${\,\widehat{\!T\!}\,\,}^{\mu \nu }$ follow from estimates for $T^{\mu \nu }$). By the reduced Einstein equations (1.22) and the energy inequality (1.23),

1.25 $$\begin{aligned}&Q_N(t) \lesssim Q_N(0)\nonumber \\&\quad + C \varepsilon \int _0^t \frac{Q_N(\tau )}{1+\tau }\,\mathrm{d}\tau +\sum _{\vert I \vert \le N} \int _0^t \Vert [Z^I, \widetilde{\square }_g] h^1 (s,\cdot )\Vert _{L^2} + \Vert Z^I \widetilde{\square }_g h^0 (s,\cdot )\Vert _{L^2} \nonumber \\&\quad + \Vert Z^I F_{\mu \nu } (s,\cdot )\Vert _{L^2} + \Vert Z^I {\,\widehat{\!T\!}\,\,} (s,\cdot )\Vert _{L^2} \, \mathrm{d}s. \end{aligned}$$

The first four terms on the right hand side arise already in [37] and so, combining estimates which will be shown for these terms in Section 6 (see also the discussion below) with (1.24), the bound (1.25) implies that

$$\begin{aligned} Q_N(t) \le C \varepsilon (1+t)^{C\varepsilon } + C \varepsilon \int _0^t \frac{Q_N(\tau )}{1+\tau }\,\mathrm{d}\tau , \end{aligned}$$

and so the Grönwall inequality yields

$$\begin{aligned} Q_N(t) \le C\varepsilon (1+t)^{2C\varepsilon }. \end{aligned}$$

In the proof of Theorem 1.2, such a bound for $Q_N$ will lead to a recovery of the assumption (1.21) on $E_N$ with better constants provided $C_N$ is chosen to be sufficiently large and $\varepsilon$ is sufficiently small.

#### The Structure of the Nonlinear Terms

As discussed above, whether a given quasilinear wave equation admits global solutions for small data or not depends on the structure of the nonlinear terms (moreover, the main analysis of the nonlinear terms is relevant in the wave zone, where ${{\,\widehat{\!T\!}\,\,}}^{\mu \nu }$ vanishes at late times and so plays no role in this discussion). A closer inspection of the nonlinearity in (1.22) reveals (see [11, 37]) that

1.26 $$\begin{aligned} F_{\mu \nu }(h)(\partial h,\partial h) = P(\partial _{\mu } h,\partial _{\nu } h) + Q_{\mu \nu }(\partial h,\partial h)+G_{\mu \nu }(h)(\partial h,\partial h), \end{aligned}$$

where $Q_{\mu \nu }(\partial h,\partial h)$ is a linear combination of *null forms* (satisfing $\vert Q_{\mu \nu }(\partial h,\partial h) \vert \lesssim \vert \overline{\partial } h \vert \vert \partial h \vert$), $\vert G_{\mu \nu }(h)(\partial h,\partial h) \vert \lesssim \vert h \vert \vert \partial h \vert ^2$ denote cubic terms and

1.27 $$\begin{aligned} P(\partial _{\mu }h,\partial _{\nu } h) = \frac{1}{2} m^{\alpha \alpha ^\prime }m^{\beta \beta ^\prime } \partial _\mu h_{\alpha \beta } \, \partial _\nu h_{\alpha ^\prime \beta ^\prime } - \frac{1}{4} m^{\alpha \alpha ^\prime }\partial _\mu h_{\alpha \alpha ^\prime } \, m^{\beta \beta ^\prime }\partial _\nu h_{\beta \beta ^\prime }. \end{aligned}$$

Clearly the failure of the semilinear terms of the system (1.22) to satisfy the classical null condition arises in the $P(\partial _{\mu } h, \partial _{\nu } h)$ terms. In [35] it was observed that the semilinear terms of (1.22), after being decomposed with respect to a null frame $\mathcal {N} = \{ \underline{L}, L, S_1, S_2 \}$, where

1.28 $$\begin{aligned} \underline{L}=\partial _t-\partial _r, \quad L=\partial _t+\partial _r, \quad S_1,S_2\in \mathbf {S}^2, \quad \langle S_i,S_j\rangle =\delta _{ij} \end{aligned}$$

possess a *weak null structure*. It is well known that, for solutions of wave equations, derivatives tangential to the outgoing light cones $\overline{\partial }\in \mathcal {T}=\{L,S_1,S_2\}$ decay faster and so, neglecting such $\overline{\partial } h$ derivatives of *h*,

1.29 $$\begin{aligned} \partial _\mu h\sim L_\mu \partial _q h,\quad \text {where}\quad \partial _q=(\partial _r-\partial _t)/2, \quad L_\mu =m_{\mu \nu } L^\nu , \end{aligned}$$

and, neglecting cubic terms and quadratic terms involving at least one tangential derivative,

1.30 $$\begin{aligned} \widetilde{\square }_g h_{\mu \nu }\sim L_\mu L_\nu P(\partial _q h,\partial _q h). \end{aligned}$$

For vectors *U*, *V*, define $(\widetilde{\square }_g h)_{UV} = U^{\mu } V^{\nu } \widetilde{\square }_g h_{\mu \nu }$. With respect to the null frame (1.28), the Einstein equations (1.22) become

1.31 $$\begin{aligned} (\widetilde{\square }_g h)_{TU}\sim 0, \quad T\in \mathcal {T},U\in \mathcal {N}\quad (\widetilde{\square }_g h)_{\underline{L}\underline{L}}\sim 4 P(\partial _q h,\partial _q h), \end{aligned}$$

since $T^\mu L_\mu =0$ for $T\in \mathcal {T}$. Decomposing with respect to the null frame (1.28), the term $P(\partial _q h,\partial _q h)$ is equal to $P_{\mathcal {N}}(\partial _{q} h, \partial _{q} h)$, where

1.32 $$\begin{aligned} P_{\!\mathcal {N}}(D,E)= & {} -\big (D_{LL} E_{\underline{L}\underline{L}} +D_{\underline{L}\underline{L}} E_{{L}{L}}\big )/8 -\big (2D_{AB}E^{AB}- D_{\!\! A}^{\, A} E_{B}^{\,\,\,B}\big )/4\nonumber \\&+\big (2D_{A L}E^{A}_{\,\,\, \underline{L}} +2D_{A \underline{L}}E^A_{\,\,\,{L}}- D_{\!\!A}^{\, A} E_{L\underline{L}}-D_{L\underline{L}}E_{\!A}^{\,\,A}\big )/4 \end{aligned}$$

(see [36]). Except for the $\partial _q h_{LL} \partial _q h_{\underline{L} \underline{L}}$ term, $P_{\!\mathcal {N}}( \partial _{q} h, \partial _{q} h)$ only involves the non $\underline{L} \underline{L}$ components of *h*. The wave coordinate condition (1.12) with respect to the null frame becomes

1.33 $$\begin{aligned} \partial _q h_{L T}\sim 0,\quad T\in \mathcal {T},\quad \delta ^{AB}\partial _q h_{AB}\sim 0,\quad A,B\in \mathcal {S}=\{S_1,S_2\}, \end{aligned}$$

neglecting tangential derivatives and quadratic terms, see [36]. In particular, the $\partial _q h_{LL} \partial _q h_{\underline{L} \underline{L}}$ term in $P_{\!\mathcal {N}}( \partial _{q} h, \partial _{q} h)$ can be neglected. The asymptotic identity (1.33) moreover implies (see equation (6.47)) that the leading order behaviour of $P_N(\partial _{q} h, \partial _{q} h)$ is contained in $P_{\!\mathcal {S}}(\partial _q h,\partial _q h)$, where

1.34 $$\begin{aligned}&P_{\mathcal {S}} (D,E)= -\widehat{D}_{AB}\, \widehat{E}^{AB}\!/2,\quad A,B\in \mathcal {S},\nonumber \\&\quad \text {where} \quad \widehat{D}_{AB}=D_{AB}-\delta _{AB}\,/{\!\!\!\!\text {tr}}\,D/2,\quad \,/{\!\!\!\!\text {tr}}\,D=\delta ^{AB}D_{AB}. \end{aligned}$$

A decoupling therefore occurs in the semilinear terms of (1.22), modulo terms which are cubic or involve at least one “good” $\overline{\partial }$ derivative, and the right hand side of the second identity in (1.31) only depends on components we have better control on by the first identity in (1.31).

A further failure of (1.22) to satisfy the classical null condition arises in the *quasilinear terms*. Expressing the inverse of the metric $g_{\mu \nu }$ as

$$\begin{aligned} g^{\mu \nu } = m^{\mu \nu }+H^{\mu \nu }, \end{aligned}$$

the reduced wave operator takes the form

$$\begin{aligned} \widetilde{\square }_g = m^{\alpha \beta } \partial _{\alpha } \partial _{\beta } + H^{\alpha \beta } \partial _{\alpha } \partial _{\beta }, \end{aligned}$$

which differs from the Minkowski wave operator $\square$ only by the term $H^{\underline{L} \underline{L}} \partial _q^2$, plus terms which involve at least one tangential $\overline{\partial }$ derivative. This main quasilinear term is controlled by first rewriting the wave coordinate condition (1.12) as

1.35 $$\begin{aligned} \partial _\mu \widehat{H}^{\mu \nu \!} = W^{\nu }(h,\partial h)\quad \text {where}\quad \widehat{H}^{\mu \nu } = H^{\mu \nu }\!-m^{\mu \nu } \text {tr}_m H_{\!}/2,\quad \text {tr}_m H=m_{\alpha \beta } H^{\alpha \beta }, \end{aligned}$$

where $\vert W^{\nu }(h,\partial h) \vert \lesssim \vert h \vert \vert \partial h \vert$ is quadratic, and using the formula

$$\begin{aligned} \partial _\mu F^{\mu \nu } = L_\mu \partial _q F^{\mu \nu } -\underline{L}_{\,\mu } \partial _s F^{\mu \nu } + A_\mu \partial _A F^{\mu \nu }, \end{aligned}$$

for any $F^{\mu \nu }$, to rewrite $\partial _\mu \widehat{H}^{\mu \nu }$ in terms of the null frame. Here $\partial _s=(\partial _r+\partial _t)/2$. This gives

$$\begin{aligned} \partial _q H^{\underline{L}\underline{L}} = L( H^{L \underline{L}} ) - \partial _A H^{A \underline{L}} + W^{\underline{L}}(h,\partial h), \end{aligned}$$

that is $\partial _q H^{\underline{L}\underline{L}}$ is equal to quadratic terms plus terms involving only tangential $\overline{\partial }$ derivatives. Integrating $\partial _q H^{\underline{L}\underline{L}}$ from initial data $\{t=0\}$ then gives that $H^{\underline{L} \underline{L}}$ is approximately equal to the main contribution of its corresponding initial value, 2*M* / *r*.

#### Commutation

In order to apply the energy inequality (1.23) to improve the higher order energy bounds (1.21), it is necessary to commute the system (1.22) with the vector fields *Z*. Instead of commuting with the vector fields *Z* directly as in [37], notice that, for any function $\phi$,

$$\begin{aligned} \widetilde{\square }_g Z \phi= & {} Z \left( \widetilde{\square }_g \phi \right) + 2 g^{\alpha \beta } \partial _{\alpha } Z^{\mu } \partial _{\beta } \partial _{\mu } \phi - Z(g^{\alpha \beta }) \partial _{\alpha } \partial _{\beta } \phi \\= & {} Z \left( \widetilde{\square }_g \phi \right) - (\mathcal {L}_Z g^{\alpha \beta }) \partial _{\alpha } \partial _{\beta } \phi , \end{aligned}$$

where the fact that $\partial _{\mu } \partial _{\nu } Z^{\lambda } = 0$ for each *Z* and $\mu , \nu , \lambda = 0,1,2,3$ has been used. Here $\mathcal {L}_Z$ denotes the Lie derivative along the vector field *Z* (see Section 6.3 for a coordinate definition). The procedure of commuting the system (1.22) therefore becomes computationally much simpler if it is instead commuted with the Lie derivatives along the vector fields, $\mathcal {L}_Z$. In fact, the procedure simplifies further by commuting with a modified Lie derivative $\widehat{\mathcal {L}}$, defined in the (*t*, *x*) coordinates by the formula

$$\begin{aligned} \widehat{{\mathcal {L}}}_Z K^{\alpha _1\dots \alpha _r}_{\beta _1\dots \beta _s} = {{\mathcal {L}}}_Z K^{\alpha _1\dots \alpha _r}_{\beta _1\dots \beta _s} + \tfrac{r-s}{4}(\partial _\gamma Z^\gamma )K^{\alpha _1\dots \alpha _r}_{\beta _1\dots \beta _s}. \end{aligned}$$

The modified Lie derivative has the property that $\widehat{\mathcal {L}}_Z m = 0$ for each of the vector fields *Z* and moreover a computation shows that, in the case that $\phi _{\mu \nu }$ is a (0, 2) tensor, the commutation property

1.36 $$\begin{aligned} \widetilde{\square }_g \widehat{\mathcal {L}}_Z \phi _{\mu \nu } = \mathcal {L}_Z \left( \widetilde{\square }_g \phi _{\mu \nu } \right) - (\widehat{\mathcal {L}}_Z H^{\alpha \beta }) \partial _{\alpha } \partial _{\beta } \phi _{\mu \nu } \end{aligned}$$

holds for each of the vector fields *Z*.

The commutation error in (1.36) can be controlled by $(\widehat{\mathcal {L}}_Z H^{\underline{L}\underline{L}}) \partial ^2 \phi _{\mu \nu }$, plus terms which involve at least one tangential derivative of $\phi _{\mu \nu }$

$$\begin{aligned} \vert (\widehat{\mathcal {L}}_Z H^{\alpha \beta }) \partial _{\alpha } \partial _{\beta } \phi _{\mu \nu } \vert \lesssim \vert \widehat{\mathcal {L}}_Z H^{\underline{L}\underline{L}} \vert \vert \partial ^2 \phi \vert + \vert \widehat{\mathcal {L}}_Z H \vert \vert \overline{\partial } \partial \phi \vert . \end{aligned}$$

The Lie derivative along any of the vector fields *Z* commutes with partial derivatives $\partial$ (see Proposition 6.24). This fact leads to the commutation formula

$$\begin{aligned} \partial _\mu \widehat{{\mathcal {L}}}_Z \widehat{H}^{\mu \nu }=\big (\widehat{{\mathcal {L}}}_Z +\tfrac{\partial _\gamma Z^\gamma }{2}\big )\partial _\mu \widehat{H}^{\mu \nu }, \end{aligned}$$

involving the modified Lie derivative. The term $\widehat{\mathcal {L}}_Z H^{\underline{L}\underline{L}}$ is then controlled easily by using the formula (1.35) and repeating the argument, described in Section 1.4.3, used to control $H^{\underline{L}\underline{L}}$ itself.

When applying the commutation formula (1.36) to the reduced Einstein equations (1.22), it in particular becomes necessary to estimate the Lie derivative of the nonlinear terms, $\mathcal {L}_Z^I \left( F_{\mu \nu }(h)(\partial h, \partial h) \right)$. Recall the nonlinear terms take the form (1.26). The modified Lie derivative also simplifies the process of understanding derivatives of the nonlinear terms due to the following product rule. Let $h_{\alpha \beta }$ and $k_{\alpha \beta }$ be (0, 2) tensors and let $S_{\mu \nu }(\partial h,\partial k)$ be a (0, 2) tensor which is a quadratic form in the (0, 3) tensors $\partial h$ and $\partial k$ with two contractions with the Minkowski metric (in particular $P(\partial _\mu h,\partial _\nu k)$ or $Q_{\mu \nu }(\partial h,\partial k)$). Then

$$\begin{aligned} {{\mathcal {L}}}_Z\,\big ( S_{\mu \nu }(\partial h,\partial k)\big ) =S_{\mu \nu }(\partial \widehat{{\mathcal {L}}}_Z h,k)+S_{\mu \nu }(\partial h,\partial \widehat{{\mathcal {L}}}_Z k), \end{aligned}$$

and so the desirable structure of the nonlinear terms $P(\partial _\mu h,\partial _\nu h)$ and $Q_{\mu \nu }(\partial h,\partial k)$ described in Section 1.4.3 is preserved after applying Lie derivatives.

The Lie derivatives of the energy momentum tensor are controlled by Theorem 1.3 since, for any function $\phi$, the quantities $\sum _{\vert I \vert \le N} \vert Z^I \phi \vert$ and $\sum _{\vert I \vert \le N} \vert \widehat{{\mathcal {L}}}_Z^I \phi \vert$ are comparable.

#### The Klainerman–Sobolev Inequality with Weights

In order to control the derivatives of the nonlinear terms and the error terms arising from commuting the system (1.22), described in Section 1.4.4, when using the energy inequality (1.23), pointwise estimates for lower order derivatives of the solution are first shown to hold. The *Klainerman–Sobolev Inequality* can be used to derive non-sharp bounds for $\vert \partial Z^I h^1 \vert$ for $\vert I \vert \le N-3$ (see equation (6.10)) directly from the bound on the energy (1.21). These pointwise bounds can be integrated from $\{t=0\}$ to also give pointwise bounds for $\vert Z^I h^1\vert$ for $\vert I \vert \le N-3$ which, using the fact that $\vert \overline{\partial } \phi \vert \le \frac{C}{1+t+r} \sum _{\vert I \vert = 1} \vert Z^I \phi \vert$ for any function $\phi$, lead to strong pointwise estimates for all components of $\overline{\partial } Z^I h^1$ for $\vert I \vert \le N-4$. See Section 6.1 for more details.

Since, without restricting things to tangential derivatives, it is only true that $\vert \partial \phi \vert \le \frac{C}{1+\vert t-r\vert } \sum _{\vert I \vert = 1} \vert Z^I \phi \vert$ for any function $\phi$, the Klainerman–Sobolev Inequality does not directly lead to good pointwise estimates for all derivatives of $h^1$. Some further improvement is necessary to control the terms in the energy estimate and recover the inequality (1.21).

#### $L^{\infty }$–$L^{\infty }$ Estimate for the Wave Equation

The pointwise decay obtained for the transverse derivative of certain components of $h^1$ “for free” from the wave coordinate condition is not sufficient in the wave zone $t\sim r$ to close the energy estimate. The decay in this region is further improved by an $L^{\infty }$–$L^{\infty }$ estimate, obtained by integrating the equations along the outgoing characteristics of the wave equation. In fact, instead of using the estimate for the full wave operator $\widetilde{\square }_g$, as in [37], it suffices to use the estimate for the operator $\square _0 = \big ( m^{\alpha \beta } - \frac{M}{r} \chi \big ( \frac{r}{1+t} \big ) \delta ^{\alpha \beta } \big ) \partial _{\alpha } \partial _{\beta }$. Moreover, using the pointwise decay obtained from the Klainerman–Sobolev inequality and the wave coordinate condition, it can be seen that, for the purposes of this estimate, the essential contribution of the failure of (1.11) to satisfy the classical null condition is present in the $P_{\!\mathcal {S}}(\partial _q h,\partial _q h)$ terms, defined by (1.34). See Proposition 6.20 for a precise statement of this, and Lemma 6.21 for a proof of the $L^{\infty }$–$L^{\infty }$ inequality. The fact that *f* is supported away from the wave zone can be shown using only the decay obtained from the Klainerman–Sobolev Inequality, and so the $\widehat{T}$ term in (1.22) plays no role in Lemma 6.21. The pointwise decay of higher order Lie derivatives of $h^1$ is similarly improved in Section 6.4.

#### The Hörmander $L^1$–$L^{\infty }$ Inequality

Whilst the pointwise decay for lower order derivatives of *h* described above is sufficient to recover the assumptions (1.21) in the vacuum (when $T^{\mu \nu } \equiv 0$), the interior decay is not sufficient to satisfy the assumptions of Theorem 1.3. In Proposition 6.11 the Hörmander $L^1$–$L^{\infty }$ inequality, Lemma 6.7, is used, together with the assumptions (1.21) on the $L^1$ norms of $Z^I T^{\mu \nu }(t,\cdot )$ and on the energy $E_N(t)$ of $h^1$, in order to improve the interior decay of $h^1$ and lower order derivatives. This improved decay ensures that the assumptions of Theorem 1.3 are satisfied and hence the theorem can be appealed to in order to recover the assumptions (1.21) on the $L^1$ norms of $Z^I T^{\mu \nu }$, and to control the $L^2$ norms of $Z^I T^{\mu \nu }$ arising when the energy inequality (1.23) is used to improve the assumptions (1.21) on the energy $E_N$. See Section 7 for further details on the completion of the proof of Theorem 1.2.

### Vector Fields for the Vlasov Equation

The remaining difficulty in the proof of Theorem 1.2 is in establishing the $L^1$ and $L^2$ estimates of the vector fields applied to components of the energy momentum tensor, $Z^I T^{\mu \nu }(t,x)$, of Theorem 1.3. For simplicity, we outline here how bounds are obtained for $Z \rho (t,x)$, for $Z = \Omega _{ij}, B_i, S$, where $\rho (t,x)$ is the momentum average of *f*, defined by

$$\begin{aligned} \rho (t,x) := \int f(t,x,{\,\widehat{\!p\!}\,\,})\, \mathrm{d} {\,\widehat{\!p\!}\,\,}. \end{aligned}$$

The bounds for $Z \rho (t,x)$ will follow from bounds of the form

1.37 $$\begin{aligned} \left| (\overline{Z}f)(t, x, {\,\widehat{\!p\!}\,\,}) \right| \le C, \end{aligned}$$

for a suitable collection of vector fields $\overline{Z}$, acting on functions of $(t,x,{\,\widehat{\!p\!}\,\,})$, which reduce to the $Z = \Omega _{ij}, B_i, S$ vector fields when acting on spacetime functions, that is functions of (*t*, *x*) only.

Throughout this section, and in Sections 4 and 5, it is convenient, instead of considering initial data to be given at $t=0$, to consider initial data for the Vlasov equation to be given at $t=t_0$ for some $t_0\ge 1$.^4^ It follows from the form of the Vlasov equation (1.18) that

1.38 $$\begin{aligned} \overline{Z}f(t,x,{\,\widehat{\!p\!}\,\,})= & {} \overline{Z}\big ( X(t_0,t,x,{\,\widehat{\!p\!}\,\,})^i \big ) (\partial _{x^i} f) (t_0,X(t_0), {\,\widehat{\!P\!}\,\,}(t_0)) \nonumber \\&+ \overline{Z}\big ( {\,\widehat{\!P\!}\,\,}(t_0,t,x,{\,\widehat{\!p\!}\,\,})^i \big ) (\partial _{{\,\widehat{\!p\!}\,\,}^i} f) (t_0,X(t_0), {\,\widehat{\!P\!}\,\,}(t_0)), \end{aligned}$$

(where $X(t_0,t,x,{\,\widehat{\!p\!}\,\,}), {\,\widehat{\!P\!}\,\,}(t_0,t,x,{\,\widehat{\!p\!}\,\,})$ are abbreviated to $X(t_0), {\,\widehat{\!P\!}\,\,}(t_0)$ respectively) for any vector $\overline{Z}$. Since derivatives of $f|_{t=t_0}$ are explicitly determined by initial data and behave like *f*, an estimate for $\vert \overline{Z}f(t,x,{\,\widehat{\!p\!}\,\,}) \vert$ will follow from appropriate estimates for $\vert \overline{Z}\left( X(t_0,t,x,{\,\widehat{\!p\!}\,\,})^i \right) \vert$ and $\vert \overline{Z}\big ( {\,\widehat{\!P\!}\,\,}(t_0,t,x,{\,\widehat{\!p\!}\,\,})^i \big ) \vert$.

#### General Procedure and Vector Fields in Minkowski Space

A natural way to extend a given vector field *Z* on $\mathcal {M}$ to a vector field on $\mathcal {P}$, which by construction will have the property that $\overline{Z}(X(t_0)^i)$ satisfy good bounds, is as follows. For a given vector field *Z* on $\mathcal {M}$, let $\Phi ^Z_{\lambda } : \mathcal {M} \rightarrow \mathcal {M}$ denote the associated one parameter family of diffeomorphisms, so that

$$\begin{aligned} \frac{\mathrm{d} \Phi ^Z_{\lambda }(t,x)}{\mathrm{d}\lambda } \bigg \vert _{\lambda = 0} = Z\vert _{(t,x)}. \end{aligned}$$

Under a mild assumption on g, for fixed $\tau$ any point $(t,x,{\,\widehat{\!p\!}\,\,}) \in \mathcal {P}$ with $t >\tau$ can be uniquely described by a pair of points $\{ (t,x), (\tau ,y) \}$ in $\mathcal {M}$, where

1.39 $$\begin{aligned} y=X(\tau ,t,x,{\,\widehat{\!p\!}\,\,}) \end{aligned}$$

is the point where the geodesic emanating from (*t*, *x*) with velocity ${\,\widehat{\!p\!}\,\,}$ intersects the hypersurface $\Sigma _{\tau }$ (recall that $\{ \Sigma _t \}$ denotes the level hypersurfaces of the function *t*), that is $(t,x,{\,\widehat{\!p\!}\,\,}) \in \mathcal {P}$ can be parameterised by $\{ (t,x), (\tau ,y) \}$ to get^5^

$$\begin{aligned} (t,x,{\,\widehat{\!p\!}\,\,}) = (t,x,{\,\widehat{\!p\!}\,\,}_X(t,x,\tau ,y)). \end{aligned}$$

The subscript *X* is used in ${\,\widehat{\!p\!}\,\,}_X(t,x,\tau ,y)$ to emphasise that ${\,\widehat{\!p\!}\,\,}$ is parametrised by *y* using the geodesics *X* (in contrast to suitable approximations to the geodesics, as will be considered later). Now the action of $\Phi ^Z_{\lambda }$ on (*t*, *x*) and $(\tau ,y)$ induces an action on $\mathcal {P}$ at time *t*, given by

$$\begin{aligned} \overline{\Phi }^{Z,X}_{\lambda ,\tau }(t,x,{\,\widehat{\!p\!}\,\,}) := \left( \Phi ^Z_{\lambda }(t,x), {\,\widehat{\!p\!}\,\,}_X\left( \Phi ^Z_{\lambda }(t,x), \Phi ^Z_{\lambda }(\tau ,y) \right) \right) . \end{aligned}$$

For fixed $t_0$ we define the vector field $\overline{Z}$ by

$$\begin{aligned} \overline{Z}\vert _{(t,x,{\,\widehat{\!p\!}\,\,})} = \frac{\mathrm{d} \overline{\Phi }^{Z,X}_{\lambda ,\tau }(t,x,{\,\widehat{\!p\!}\,\,})}{\mathrm{d}\lambda } \bigg \vert _{\lambda = 0,\,\tau =t_0}. \end{aligned}$$

A computation shows that

1.40 $$\begin{aligned} \overline{Z}\vert _{(t,x,{\,\widehat{\!p\!}\,\,})} \left( X(t_0,t,x,{\,\widehat{\!p\!}\,\,})^i \right) = Z^i \vert _{(t_0,X(t_0))} - Z^0 \vert _{(t_0,X(t_0))} {\,\widehat{\!P\!}\,\,}(t_0,t,x,{\,\widehat{\!p\!}\,\,})^i, \end{aligned}$$

which results in a good bound for $\vert \overline{Z}(X(t_0)^i) \vert$. In particular, if $X^i(t_0,t,x,{\,\widehat{\!p\!}\,\,})$ and ${\,\widehat{\!P\!}\,\,}^i(t_0,t,x,{\,\widehat{\!p\!}\,\,})$ are bounded in the support of $f(t_0,X(t_0), {\,\widehat{\!P\!}\,\,}(t_0))$, equation (1.40) guarantees that $\vert \overline{Z}(X(t_0)^i) \vert$ is bounded by a constant.

To see that (1.40) indeed holds, first note that the left hand side is the derivative of $X\big (t_0,\overline{\Phi }^{Z,X}_{\lambda ,t_0}(t,x,{\,\widehat{\!p\!}\,\,})\big )^i$ with respect to $\lambda$ at $\lambda =0$. Also the first term on the right hand side is the derivative of $\Phi ^Z_{\lambda }(t_0,y)^i$ at $\lambda =0$. The equality (1.40) follows from taking the derivative with respect to $\lambda$, and setting $\lambda =0$, of both sides of the identity

1.41 $$\begin{aligned} X\big (\Phi ^Z_{\lambda }(t_0,y)^0,\overline{\Phi }^{Z,X}_{\lambda ,t_0}(t,x,{\,\widehat{\!p\!}\,\,})\big )^i =\Phi ^Z_{\lambda }(t_0,y)^i. \end{aligned}$$

In Minkowski space, *y* has the explicit form $y=x-(t-\tau ){\,\widehat{\!p\!}\,\,}$ and, when *Z* is chosen to be $\Omega _{ij}, B_i, S$, a straightforward computation, see Section 3, shows that the resulting vectors $\overline{Z}^M\!$ take the form,

$$\begin{aligned} \overline{\Omega }_{ij}^M&= x^i \partial _{x^j} - x^j \partial _{x^i} {+} {\,\widehat{\!p\!}\,\,}^i \partial _{{\,\widehat{\!p\!}\,\,}^j} {-} {\,\widehat{\!p\!}\,\,}^j \partial _{{\,\widehat{\!p\!}\,\,}^i}, \quad \overline{B}_i^M = x^i \partial _t + t \partial _{x^i} + \left( \delta _i^j - {\,\widehat{\!p\!}\,\,}^i {\,\widehat{\!p\!}\,\,}^j \right) \partial _{{\,\widehat{\!p\!}\,\,}^j}, \\ \overline{S}^M&= t\partial _t + x^k \partial _{x^k}. \end{aligned}$$

Let $\widehat{q}_X(t,x,t_0,y)=\widehat{P}(t_0,t,x,p)$ be the initial momentum of the geodesic going through $(t,x,\widehat{p})$. By differentiating the equality

$$\begin{aligned} {\,\widehat{\!P\!}\,\,}\left( \Phi _{\lambda }^Z (t_0,y)^0, \overline{\Phi }^{Z,X}_{\lambda ,t_0}(t,x,{\,\widehat{\!p\!}\,\,}) \right) ^i = \widehat{q}_X \left( \Phi _{\lambda }^Z(t,x), \Phi _{\lambda }^Z(t_0,y) \right) ^i, \end{aligned}$$

with respect to $\lambda$, one can similarly obtain an estimate for $\overline{Z}({\,\widehat{\!P\!}\,\,}^i(t_0))$. In the simple case of Minkowski space, $\widehat{q}_X$ has the explicit form

$$\begin{aligned} \widehat{q}_X \left( \Phi _{\lambda }^Z(t,x), \Phi _{\lambda }^Z(t_0,y) \right) ^i = \frac{\Phi _{\lambda }^Z(t,x)^i - \Phi _{\lambda }^Z(t_0,y)^i}{\Phi _{\lambda }^Z(t,x)^0 - \Phi _{\lambda }^Z(t_0,y)^0}, \end{aligned}$$

and so

$$\begin{aligned} \overline{Z}\vert _{(t,x,{\,\widehat{\!p\!}\,\,})} \left( {\,\widehat{\!P\!}\,\,}(t_0,t,x,{\,\widehat{\!p\!}\,\,})^i \right) = \frac{Z^i \vert _{(t,x)} - Z^i\vert _{(t_0,X(t_0))}}{t-t_0} - \frac{Z^0 \vert _{(t,x)} - Z^0\vert _{(t_0,X(t_0))}}{t-t_0} {\,\widehat{\!p\!}\,\,}^i, \end{aligned}$$

since ${\mathrm{d}{\,\widehat{\!P\!}\,\,}}\!/{ \mathrm{d}s} = 0$ in Minkowski space, which leads to a good bound for $\vert \overline{Z}({\,\widehat{\!P\!}\,\,}^i(t_0)) \vert$ and, together with (1.40), results in bounds of the form (1.37) for solutions of the Vlasov equation on Minkowski space. Such bounds lead to bounds on $Z \rho (t,x)$, since

$$\begin{aligned} \Omega _{i\!j\,} \rho (t,x)= & {} \!\int \! \overline{\Omega }^M_{i\!j}\! f (t,x,{\,\widehat{\!p\!}\,\,})\, \mathrm{d}{\,\widehat{\!p\!}\,\,},\\ B_{{}_{\!}i} \rho (t,x)= & {} \!\int \! \overline{B}^M_{\!i} \!\!f (t,x,{\,\widehat{\!p\!}\,\,}) - 4 {\,\widehat{\!p\!}\,\,}^i f (t,x,{\,\widehat{\!p\!}\,\,}) \,\mathrm{d}{\,\widehat{\!p\!}\,\,},\\ S \rho (t,x)= & {} \!\int \! \overline{S}^M \!\!f(t,x,{\,\widehat{\!p\!}\,\,}) \,\mathrm{d}{\,\widehat{\!p\!}\,\,}. \end{aligned}$$

The rotation vector fields $\overline{\Omega }^M_{ij}$ and a form of the scaling vector field $\overline{S}^M$ were used in [46] for small data solutions of the massless Einstein–Vlasov system (note though that the above procedure of using *Z* to define $\overline{Z}$ breaks down when the mass shell $\mathcal {P}$ becomes the set of null vectors, as is the case for the massless Einstein–Vlasov system).^6^ The vector fields $\overline{\Omega }^M_{ij}$, $\overline{B}^M_i$, $\overline{S}^M$ were also used in the work [17] on the Vlasov–Nordström system, where the authors notice that the rotations $\overline{\Omega }^M_{ij}$ and the boosts $\overline{B}^M_i$ are the *complete lifts* of the spacetime rotations and boosts, and hence generate symmetries of the tangent bundle.

#### Vector Fields Used in the Proof of Theorem 1.3

In the proof of Theorem 1.2, in order to obtain good estimates for $Z^I T^{\mu \nu }(t,x)$, it is necessary to obtain bounds of the form (1.37), now for solutions of the Vlasov equation on the spacetimes being constructed. The sharp interior decay rate of the Christoffel symbols in the spacetimes which are constructed, as we plan to show in forthcoming work, is

1.42 $$\begin{aligned} \left| \, \Gamma ^{\alpha }_{\beta \gamma }(t,x) \right| \le \frac{C}{t^2} \quad \text {for } \vert x \vert \le c t +K, \end{aligned}$$

where $0<c<1$ and $K \ge 0$.^7^ On a spacetime whose Christoffel symbols decay as such, it can be shown that the Minkowski vector fields $\overline{Z}^M$ of the previous section only satisfy

$$\begin{aligned} \big \vert \overline{Z}^M f(t,x,{\,\widehat{\!p\!}\,\,}) \big \vert \le C \log t, \end{aligned}$$

for solutions *f* of the Vlasov equation. This logarithmic loss compounds at higher orders, and cannot be used to recover the sharp bounds (1.42) in the context of Theorem 1.2.

The proof of Theorem 1.2 is therefore based on a different collection of vector fields, $\overline{Z}$, which are adapted to the geometry of the background spacetime and again reduce to $Z = \Omega _{ij}, B_i, S$ when acting on spacetime functions, and satisfy a good bound of the form (1.37) when applied to solutions *f* of the Vlasov equation. The vector fields can be derived using the procedure described in Section 1.5.1, which in fact did not rely on the background spacetime being Minkowski space. Instead of the expression (1.39), the components $y^i$ are defined using approximations $X_2(s,t,x,{\,\widehat{\!p\!}\,\,})$ to the true geodesics $X(s,t,x,{\,\widehat{\!p\!}\,\,})$ of the spacetimes to be constructed^8^. One could also use the geodesics themselves, however doing so involves estimating the components $T^{\mu \nu }$ at the top order in a slightly different way to how they are estimated at lower orders. We choose to use the approximations to the geodesics so that $T^{\mu \nu }$ can be estimated in the same way at all orders. A derivation of the vector fields obtained using this procedure is given in Section 3, but here the failure of the Minkowski vector fields $\overline{Z}^M$ are identified explicitly and shown how to be appropriately corrected. The two procedures agree up to lower order terms.

Instead of the sharp interior bounds (1.42), the proof of Theorem 1.3 requires only the weaker bounds

1.43 $$\begin{aligned} \left| \, Z^I \Gamma ^{\alpha }_{\beta \gamma } (t,x) \right| \le \frac{C}{t^{1+a}}, \end{aligned}$$

for $\vert I \vert \le \left\lfloor N/2 \right\rfloor + 2$, where $\frac{1}{2}< a < 1$. Consider first the rotation vector fields, and recall the expression (1.38). The rotation vector fields $\overline{\Omega }_{ij}$ are defined using approximations to the geodesics. The geodesics take the form

$$\begin{aligned} X(s,t,x,{\,\widehat{\!p\!}\,\,})^k = x^k - (t-s){\,\widehat{\!p\!}\,\,}^k - \int _s^t (s' - s) {\,\widehat{\!\Gamma \!}\,\,}^k(s',X(s'),{\,\widehat{\!P\!}\,\,}(s')) \,\mathrm{d}s'. \end{aligned}$$

Using the fact that

$$\begin{aligned} {\,\widehat{\!P\!}\,\,}(s,t,x,{\,\widehat{\!p\!}\,\,})^k\sim & {} {\,\widehat{\!p\!}\,\,}^k \sim \frac{x^k}{t}, \nonumber \\ X(s,t,x,{\,\widehat{\!p\!}\,\,})^k\sim & {} x^k - (t-s) {\,\widehat{\!p\!}\,\,}^k \sim x^k - (t-s) \frac{x^k}{t} = s \frac{x^k}{t}, \end{aligned}$$

where each of the first approximations arise by replacing ${\,\widehat{\!P\!}\,\,}(s,t,x,{\,\widehat{\!p\!}\,\,})^k$ and $X(s,t,x,{\,\widehat{\!p\!}\,\,})^k$ by their respective values in Minkowski space, and the second arise from the fact that $\frac{x^i}{t} \sim {\,\widehat{\!p\!}\,\,}^i$, which holds asymptotically along each given geodesic (see Proposition 2.2 below), the approximations to the geodesics are defined as

1.44 $$\begin{aligned} X_2(s,t,x,{\,\widehat{\!p\!}\,\,})^k = x^k - (t-s) {\,\widehat{\!p\!}\,\,}^k - \int _s^t (s'-s) {\,\widehat{\!\Gamma \!}\,\,}^k \left( s', s'\frac{x}{t}, \frac{x}{t} \right) \, \mathrm{d}s' \end{aligned}$$

for $t_0 \le s \le t$. It will be shown in Section 2 that $X_2(s,t,x,{\,\widehat{\!p\!}\,\,})^k$ are good approximations to the geodesics $X(s,t,x,{\,\widehat{\!p\!}\,\,})^k$ in the sense that

1.45 $$\begin{aligned} \left| X_2(s,t,x,{\,\widehat{\!p\!}\,\,}) - X(s,t,x,{\,\widehat{\!p\!}\,\,}) \right| \le C \end{aligned}$$

for all $t_0\le s \le t$ and $k=1,2,3$. The idea is now to construct vector fields so that the vector fields applied to $X_2$ are bounded. Then one can show that (1.45) is true with $X_2 - X$ replaced by $\overline{Z}(X_2-X)$. See Section 4 for more details. The approximations $X_2$ have the desirable property, which will be exploited below, that

1.46 $$\begin{aligned} \partial _{{\,\widehat{\!p\!}\,\,}^l} \left( X_2(s,t,x,{\,\widehat{\!p\!}\,\,})^k - \big ( x^k - (t-s){\,\widehat{\!p\!}\,\,}^k \big ) \right) =0 \end{aligned}$$

vanishes (and in particular does not involve derivatives of $\Gamma$).

Applying the Minkowski rotation vector fields to the approximations $X_2$ gives

1.47 $$\begin{aligned} \overline{\Omega }_{ij}^M \left( X_2(t_0,t,x,{\,\widehat{\!p\!}\,\,})^k \right)&= \big ( x^i - (t-t_0){\,\widehat{\!p\!}\,\,}^i \big ) \delta _j^k - \big ( x^j - (t-t_0){\,\widehat{\!p\!}\,\,}^j \big ) \delta _i^k \nonumber \\&\quad - \overline{\Omega }_{ij}^M \left( \int _{t_0}^t (s' - t_0) {\,\widehat{\!\Gamma \!}\,\,}^k(s',s'\frac{x}{t},\frac{x}{t}) \, \mathrm{d}s' \right) \nonumber \\&= \ X_2(t_0,t,x,{\,\widehat{\!p\!}\,\,})^i \delta _j^k - X_2(t_0,t,x,{\,\widehat{\!p\!}\,\,})^j \delta _i^k\\&\quad + \int _{t_0}^t (s' - t_0) \left[ {\,\widehat{\!\Gamma \!}\,\,}^i(s',s'\frac{x}{t},\frac{x}{t}) \delta _j^k - {\,\widehat{\!\Gamma \!}\,\,}^j(s',s'\frac{x}{t},\frac{x}{t}) \delta _i^k \right. \nonumber \\&\quad \left. - \Omega _{ij} \left( {\,\widehat{\!\Gamma \!}\,\,}^k \left( s',s'\frac{x}{t},\frac{x}{t}\right) \right) \right] \, \mathrm{d}s'. \nonumber \end{aligned}$$

In the final equality, for $(t,x,{\,\widehat{\!p\!}\,\,}) \in \mathrm {supp}(f)$, the first two terms are bounded (as can be seen from (1.45) and the fact that $f\vert _{t=t_0}$ has compact support). However on a spacetime only satisfying the bounds (1.43), the terms on the last line in general grow in *t*. The vector fields $\overline{\Omega }_{ij}$ are defined so that these terms are removed:

$$\begin{aligned} \overline{\Omega }_{ij} = \overline{\Omega }_{ij}^M + \mathring{\Omega }_{ij}^l \partial _{{\,\widehat{\!p\!}\,\,}^l}, \end{aligned}$$

where if the functions $\mathring{\Omega }_{ij}^l$ are defined as

$$\begin{aligned} \mathring{\Omega }_{ij}^l(t,x) =&\int _{t_0}^t \frac{s' - t_0}{t-t_0} \Big [ {\,\widehat{\!\Gamma \!}\,\,}^i\left( s',s'\frac{x}{t} ,\frac{x}{t} \right) \delta _j^l - {\,\widehat{\!\Gamma \!}\,\,}^j\left( s',s'\frac{x}{t} ,\frac{x}{t} \right) \delta _i^l \\&-\overline{\Omega }_{ij}^M \left( {\,\widehat{\!\Gamma \!}\,\,}^l\left( s',s'\frac{x}{t} ,\frac{x}{t} \right) \right) \Big ] \, \mathrm{d}s', \end{aligned}$$

it follows from (1.46) and (1.47) that

1.48 $$\begin{aligned} \overline{\Omega }_{ij} \big ( X_2(t_0,t,x,{\,\widehat{\!p\!}\,\,})^k \big )= & {} \overline{\Omega }_{ij}^M \big ( X_2(t_0,t,x,{\,\widehat{\!p\!}\,\,})^k \big ) - (t-t_0) \mathring{\Omega }_{ij}^k(t,x) \nonumber \\= & {} X_2(t_0,t,x,{\,\widehat{\!p\!}\,\,})^i \delta _j^k - X_2(t_0,t,x,{\,\widehat{\!p\!}\,\,})^j \delta _i^k, \end{aligned}$$

and so

1.49 $$\begin{aligned} \left| \, \overline{\Omega }_{ij} \left( X_2(t_0,t,x,{\,\widehat{\!p\!}\,\,})^k \right) \right| \le C \end{aligned}$$

for $(t,x,{\,\widehat{\!p\!}\,\,}) \in \mathrm {supp}(f)$ and $k=1,2,3$. It can similarly be shown (see Section 4.2) that

$$\begin{aligned} \big \vert \, \overline{\Omega }_{ij} \big ( X(t_0,t,x,{\,\widehat{\!p\!}\,\,})^k \big ) \big \vert + \big \vert \, \overline{\Omega }_{ij} \big ( {\,\widehat{\!P\!}\,\,}(t_0,t,x,{\,\widehat{\!p\!}\,\,})^k \big ) \big \vert \le C, \end{aligned}$$

which, by (1.38), leads to a good bound for $\overline{\Omega }_{ij} f(t,x,{\,\widehat{\!p\!}\,\,})$.

We remark that the identity (1.48) can be expressed as

1.50 $$\begin{aligned} \overline{\Omega }_{ij} \big ( X_2(t_0,t,x,{\,\widehat{\!p\!}\,\,})^k \big ) = \Omega ^k\big |_{(t_0,X_2(t_0,t,x,{\,\widehat{\!p\!}\,\,}))}, \end{aligned}$$

which is exactly (1.40) when $Z = \Omega _{ij}$ and *X* is replaced by $X_2$. The rotation vector fields $\overline{\Omega }_{ij}$ therefore arise by following the procedure in Section 1.5.1 with *X* replaced by $X_2$; see Section 3.

Since the functions $\mathring{\Omega }_{ij}^l$ do not depend on ${\,\widehat{\!p\!}\,\,}$,

$$\begin{aligned}&\Omega _{ij} \left( \int f(t,x,{\,\widehat{\!p\!}\,\,}) \,\mathrm{d} {\,\widehat{\!p\!}\,\,}\right) \\&\quad = \int \left( \overline{\Omega }_{ij} - \mathring{\Omega }_{ij}^l \partial _{{\,\widehat{\!p\!}\,\,}^l} \right) f(t,x,{\,\widehat{\!p\!}\,\,}) \,\mathrm{d}{\,\widehat{\!p\!}\,\,}= \int \overline{\Omega }_{ij} f(t,x,{\,\widehat{\!p\!}\,\,}) \,\mathrm{d}{\,\widehat{\!p\!}\,\,}, \end{aligned}$$

and so the good bounds for $\overline{\Omega }_{ij} f(t,x,{\,\widehat{\!p\!}\,\,})$ lead to good bounds for $\Omega _{ij} \rho (t,x)$.

Had the true geodesics been used to define the vector fields $\overline{\Omega }_{ij}$, the functions $\mathring{\Omega }_{ij}^l$ would have involved a term of the form $\int _{t_0}^t ({s' - t_0})({t-t_0})^{-1} \overline{\Omega }_{ij}^M \big ( {\,\widehat{\!\Gamma \!}\,\,}^k\big ( s',X(s'),{\,\widehat{\!P\!}\,\,}(s') \big ) \big )\, \mathrm{d}s'$ and so $\partial _{{\,\widehat{\!p\!}\,\,}^k} \mathring{\Omega }_{ij}^l$ would involve second order derivatives of $\Gamma$. The average $\int \mathring{\Omega }_{ij}^l \partial _{{\,\widehat{\!p\!}\,\,}^l} f (t,x,{\,\widehat{\!p\!}\,\,}) \,\mathrm{d} {\,\widehat{\!p\!}\,\,}$ has better decay than the term $\mathring{\Omega }_{ij}^l \partial _{{\,\widehat{\!p\!}\,\,}^l} f (t,x,{\,\widehat{\!p\!}\,\,})$ does pointwise and so, to exploit this fact when obtaining a bound for $\Omega _{ij} \rho (t,x)$, it is necessary to integrate this term by parts $\int \mathring{\Omega }_{ij}^l \partial _{{\,\widehat{\!p\!}\,\,}^l} f (t,x,{\,\widehat{\!p\!}\,\,})\, \mathrm{d} {\,\widehat{\!p\!}\,\,}= - \int \big ( \partial _{{\,\widehat{\!p\!}\,\,}^l} \mathring{\Omega }_{ij}^l \big ) f (t,x,{\,\widehat{\!p\!}\,\,}) \,\mathrm{d} {\,\widehat{\!p\!}\,\,}$. If the true geodesics are used to define $\overline{\Omega }_{ij}$ then, in the setting of Theorem 1.2, the fact that $\partial _{{\,\widehat{\!p\!}\,\,}^k} \mathring{\Omega }_{ij}^l$ involves second order derivatives of $\Gamma$ would mean that this integration by parts cannot be used when estimating derivatives of $T^{\mu \nu }$ at the top order, and so $T^{\mu \nu }$ would be estimated in a slightly different way at the top order.

The approximations to the geodesics $X_2(s,t,x,{\,\widehat{\!p\!}\,\,})$ are used in a similar manner in Section 4.1 to define vector fields, $\overline{B}_i$ and $\overline{S}$.^9^

In Hwang–Rendall–Velázquez [23] derivatives of the average $\int f(t,x,p)\, \mathrm{d}p$ for solutions of the Vlasov–Poisson system are controlled and $L^{\infty }$ analogues of the estimates of Theorem 1.3 are obtained. The approach is different to that taken in the present work, though the estimates in [23] are obtained by similarly first controlling derivatives of the analogues of the maps *X*, *P*. The re-parameterisation (*t*, *x*, *y*) of $\mathcal {P}$ is also used, though whilst here it is only formally used to motivate the definition of the $\overline{Z}$ vector fields, in [23] $\partial _x$ derivatives, in the (*t*, *x*, *y*) coordinate system, of the analogue of the maps *X*, *P* are controlled.

It is also interesting to compare the present work with the independent work of Fajman–Joudioux–Smulevici [19]. The proof in [19] is also based on the vector field method (following [29, 37] in the vacuum) and, accordingly, the new elements of the proof also involve controlling vector fields applied to the components of the energy momentum tensor $T^{\mu \nu }$. The issue of the failure of the Minkowski vector fields applied to *f* to be bounded arises. A key step in [19] is therefore also to introduce a new collection of vector fields further adapted to the geometry of the spacetimes under consideration. The vector fields introduced are different to those introduced here. The construction in [19] proceeds roughly by, in the (*t*, *x*, *p*) coordinate system for $\mathcal {P}$, considering the vectors $\overline{Z}^M + C_Z(t,x,p)^{\mu } \partial _{x^{\mu }}$, where the coefficients $C_Z(t,x,p)^{\mu }$ are defined, by solving an inhomogeneous transport equation, so that $\overline{Z}^M+ C_Z(t,x,p)^{\mu } \partial _{x^{\mu }}$ has good commutation properties with $\mathbf {X}$. The solutions of the Vlasov equation in [19] are controlled by commuting the Vlasov equation (1.3) with these vector fields, in contrast to the present work where solutions of the Vlasov equation are controlled by expressing them in terms of geodesics (1.18) and commuting the geodesic equations (1.17) with vector fields.

### Outline of the Paper

In Section 2 the system (1.17) is used to prove the property (1.19) regarding the support of the matter, and the maps (1.44) are shown to be good approximations to the geodesics. In Section 3, which is not required for the proof of Theorem 1.2 or Theorem 1.3, the discussion of the vector fields in Section 1.5 is expanded on and a derivation of the vector fields used in the proof of Theorem 1.3 is given. In Section 4 combinations of the vector fields applied to the geodesics are estimated. In Section 5 the estimates of Section 4 are used to obtain estimates for spacetime vector fields applied to the components of the energy momentum tensor and hence prove Theorem 1.3. In Section 6 the solution of the reduced Einstein equations is estimated in terms of the components of the energy momentum tensor. The results of the previous sections are combined in Section 7 to give the proof of Theorem 1.2.

## The Support of the Matter and Approximations to the Geodesics

In this section the Vlasov equation on a fixed spacetime is considered. It is shown that under some assumptions on the metric the solution is supported, for large times, away from the *wave zone*$x \sim t$ provided $f_0$ is compactly supported. Curves $X_2$, which approximate the timelike geodesics *X*, are introduced, which are later used to define the $\overline{Z}$ vector fields.

Note that the characteristics of the operator $\frac{1}{p^0} \mathbf {X}$, where $\mathbf {X}$ is given by (1.5), solve

2.1 $$\begin{aligned} \frac{\mathrm{d}X^\mu }{ \mathrm{d}s} = \frac{P^\mu }{P^0}, \quad \frac{\mathrm{d} P^\mu }{ \mathrm{d}s} = - \Gamma _{\alpha \beta }^\mu (s,X(s)) \frac{P^{\alpha }}{P^0} P^{\beta }, \end{aligned}$$

and so

$$\begin{aligned} \frac{\mathrm{d} }{ \mathrm{d}s} \left( \frac{P^i}{P^0} \right) = \Gamma _{\alpha \beta }^0{\,\widehat{\!P\!}\,\,}^{\alpha } {\,\widehat{\!P\!}\,\,}^{\beta } {\,\widehat{\!P\!}\,\,}^{\mu } - \Gamma _{\alpha \beta }^{\mu } {\,\widehat{\!P\!}\,\,}^{\alpha }{\,\widehat{\!P\!}\,\,}^{\beta },\quad \text {where}\quad {\,\widehat{\!P\!}\,\,}^\alpha =P^\alpha /P^0. \end{aligned}$$

Hence

2.2 $$\begin{aligned} \frac{\mathrm{d} {X}^{\mu }}{\mathrm{d} s} = {{\,\widehat{\!P\!}\,\,}^{\mu }}, \quad \frac{\mathrm{d} {\,\widehat{\!P\!}\,\,}^\mu }{ \mathrm{d}s} = \widehat{\Gamma }\big ( s,X(s),{\,\widehat{\!P\!}\,\,}(s) \big )^\mu , \end{aligned}$$

where $$X^0=s$$, $${\,\widehat{\!P\!}\,\,}^0=1$$ and

2.3 $$\begin{aligned} \widehat{\Gamma }(t,x,{\,\widehat{\!p\!}\,\,})^\mu = {\,\widehat{\!p\!}\,\,}^\mu \Gamma _{\alpha \beta }^0(t,x){\,\widehat{\!p\!}\,\,}^\alpha {\,\widehat{\!p\!}\,\,}^\beta - \Gamma _{\alpha \beta }^\mu (t,x){\,\widehat{\!p\!}\,\,}^{\alpha }{\,\widehat{\!p\!}\,\,}^\beta . \end{aligned}$$

Let functions $\Lambda ^{\alpha \beta , \mu }_{\gamma }$ be defined so that

2.4 $$\begin{aligned} \widehat{\Gamma }(t,x,{\,\widehat{\!p\!}\,\,})^\mu =\Gamma (t,x)\cdot \Lambda ({\,\widehat{\!p\!}\,\,})^\mu := \Gamma ^{\gamma }_{\alpha \beta }(t,x) \Lambda ^{\alpha \beta , \mu }_{\gamma } ({\,\widehat{\!p\!}\,\,}), \end{aligned}$$

that is $\Lambda ^{\alpha \beta , 0}_{\gamma } ({\,\widehat{\!p\!}\,\,})=0$, $\Lambda ^{\alpha \beta , i}_{0} ({\,\widehat{\!p\!}\,\,})={\,\widehat{\!p\!}\,\,}^i{\,\widehat{\!p\!}\,\,}^{\alpha }{\,\widehat{\!p\!}\,\,}^\beta$ and $\Lambda ^{\alpha \beta , i}_{j} ({\,\widehat{\!p\!}\,\,})=-\delta _{\!j}^{\,\,i}{\,\widehat{\!p\!}\,\,}^{\alpha }{\,\widehat{\!p\!}\,\,}^\beta$, for $i=1,2,3$.

### Properties of the Support of the Matter

The results of this section will rely on the Christoffel symbols satisfying the assumptions

2.5 $$\begin{aligned} |Z^I \Gamma |\le \frac{c_{N^{\prime \prime }}^{\prime \prime }}{(1+t)^{1-\delta }(1+|t-r|)^{a+\delta }}, \quad \text {for}\quad |I|\le N^{\prime \prime } \end{aligned}$$

for various small $N^{\prime \prime }$. Then if (2.7) below holds in $\mathrm {supp}(f)$, the assumption (2.5) implies that

2.6 $$\begin{aligned} |Z^I \Gamma |\le \frac{c_{N^{\prime \prime }}^{\prime \prime \prime }}{(1+t)^{1+a}}, \quad \text {for}\quad |I|\le N^{\prime \prime },\quad \text {where}\quad c_{N^{\prime \prime }}^{\prime \prime \prime } =\frac{c_{N^{\prime \prime }}^{\prime \prime }K^{a+\delta }}{(1-c)^{a+\delta }} \end{aligned}$$

in $\mathrm {supp}(f)$. The notation (*y*, *q*) will be used for points in the mass shell over the initial hypersurface $\{t=0\}$, so $y \in \Sigma _{0}$, $q \in \mathcal {P}_{(0,y)}$.

The next result (Proposition 2.8) guarantees that

2.7 $$\begin{aligned} \left| x \right| \le c t + K,\quad K\ge 1 \quad \left| {\,\widehat{\!p\!}\,\,}\right| \le c <1 \end{aligned}$$

for $(t,x,{\,\widehat{\!p\!}\,\,}) \in \mathrm {supp}(f)$, for some constants *K* and *c*.

#### Proposition 2.1

Suppose that $|y|\le K$ and $|q|\le K^\prime$, for some $K,K'\ge 1$, and that for some fixed $a,\delta >0$

2.8 $$\begin{aligned} |g-m|\le c',\quad \text {and}\quad |\Gamma |\le \frac{c^{\prime \prime }}{(1+t)^{1-\delta }(1+|t-r|)^{a+\delta }}, \end{aligned}$$

where $c'={1}/({16(1+8{K^{\prime }}^2)})$ and $c^{\prime \prime }=\min {\big (2^{-\delta }{c'}^{\delta }K^{a}a,(1+2K/c')^{-\delta }\delta \big )}/({8(1+4{K^{\prime }})})$. Then with $c=1-c'$ we have

2.9 $$\begin{aligned} \left| P(s,0,y,q) \right|\le & {} K'+1, \quad \vert {P}(s,0,y,{\,\widehat{\!q\!}\,\,}) \vert \le c\,\vert {P}^0(s,0,y,{\,\widehat{\!q\!}\,\,}) \vert , \nonumber \\ \left| X(s,0,y,q) \right|\le & {} c s + K,\quad s\ge 0. \end{aligned}$$

#### Proof

Let $s_1$ be the largest number such that $| P(s,0,y,q)|\le 2K'$ for $0\le s\le s_1$. We will show that then it follows that $| P(s,0,y,q)|< K'+1$, for $0\le s\le s_1$, contradicting the maximality of $s_1$. Let $p=P(s,0,y,q)$. Since $g_{\alpha \beta }p^\alpha p^\beta =-1$ it follows that $\big | 1+|p_{\,}|^2-|p^0|^2\big |\le |g-m|(|p^0|^2+|p_{\,}|^2)$. Hence $(1-c')|p^0|^2\le 1+(1+c')|p_{\,}|^2$ and

$$\begin{aligned}&\frac{|p_{\,}|^2}{|p^0|^2\!}(1-c')\le {1+c'}-\frac{1}{|p^0|^2\!\!}\quad \Longrightarrow \quad \frac{|p_{\,}|^2}{|p^0|^2\!}\\&\quad \le \frac{1+c'}{1-c'}-\frac{1}{1+4{K'}^2\!}\le 1+4c'-\frac{1}{1 + (1 +c')4{K'}^2 }\le (1-c')^2=c^2, \end{aligned}$$

which proves the second and hence third part of (2.9), assuming the weaker bound of the first. It follows that

$$\begin{aligned} \frac{\mathrm{d}}{ \mathrm{d}s} \big (s-|X|\big )=1-\frac{X \cdot P}{\vert X \vert P^0} \ge 1-c, \end{aligned}$$

so $s-|X| \ge (1-c)s-K$ along a characteristic *X*(*s*). Therefore $|\Gamma \big (s,X(s)\big )|\le 2^{a+\delta }c^{\prime \prime }{c'}^{-a-\delta } s^{-1-a}$, when $s\ge 2K/c'$, and $|\Gamma |\le c^{\prime \prime }(1+s)^{-1+\delta }$, when $s\le 2K/c'$ along a characteristic. By (2.1) we have

$$\begin{aligned} \frac{\mathrm{d}}{ \mathrm{d}s} |P-q_{\,}|\le \big |\frac{\mathrm{d}}{ \mathrm{d}s} P\big |\le |\Gamma |(1+|P|), \end{aligned}$$

where

$$\begin{aligned} \int _{0}^t |\Gamma | \,\mathrm{d}s\le & {} \int _0^{2K/c'} \frac{c^{\prime \prime }}{(1+s)^{1-\delta }} \,\mathrm{d}s+\int _{2K/c'}^\infty \frac{2^{a+\delta }c^{\prime \prime }{c'}^{-a-\delta } }{s^{1+a}}\, \mathrm{d}s\\\le & {} \frac{c^{\prime \prime }(1+2K/c')^{\delta }}{\delta }+ \frac{2^{\delta }c^{\prime \prime }{c'}^{-\delta } }{aK^a} \le \frac{1}{4(1+4{K^{\prime }})}, \end{aligned}$$

by assumption. Hence, using the weak inductive assumption,

$$\begin{aligned} |P-q_{\,}|\le \int _0^t |\Gamma |(1+|P|)\, \mathrm{d}s\le \frac{1 }{4}. \end{aligned}$$

It follows that $|P(s,0,y,q)|\le K'+1/4$ in the support of *f*, and (2.9) follows.

$$\square $$

#### Proposition 2.2

If $\vert \Gamma (t,x) \vert \le c^{\prime \prime \prime }(1+t)^{-1-a}$ for $\vert x \vert \le ct +K$ then, for $(t,x,{\,\widehat{\!p\!}\,\,}) \in \mathrm {supp}(f)$ with $t\ge 1$,

2.10 $$\begin{aligned} \left| \frac{x}{t} - {\,\widehat{\!p\!}\,\,}\right| \le 2(K+c^{\prime \prime \prime })(1+t)^{-a}/(1-a). \end{aligned}$$

#### Proof

Note that any $(t,x,{\,\widehat{\!p\!}\,\,}) \in \mathrm {supp}(f)$ can be written as $(t,x,{\,\widehat{\!p\!}\,\,}) = (t,X(t,0,y,q),{\,\widehat{\!P\!}\,\,}(t,0,y,q))$ for some $(y,q) \in \mathrm {supp}(f_0)$. For (*y*, *q*) such that $(0,y,q) \in \mathrm {supp}(f)$,

$$\begin{aligned}&\left| \frac{\mathrm{d}}{\mathrm{d}s} \left( X(s,0,y,q)- s {\,\widehat{\!P\!}\,\,}(s,0,y,q) \right) \right| \\&\quad = \left| s {\,\widehat{\!\Gamma \!}\,\,}\left( s, X(s,0,y,q), {\,\widehat{\!P\!}\,\,}(s,0,y,q) \right) \right| \lesssim \frac{c^{\prime \prime \prime }}{(1+s)^a} \end{aligned}$$

for all $s\ge 0$, using the bound (2.8). The proof follows by integrating forwards from $s=0$ and using the fact that $\vert y \vert + \vert q \vert \le C$,

2.11 $$\begin{aligned} \left| X(s,0,y,q) - s {\,\widehat{\!P\!}\,\,}(s,0,y,q) \right| \le K + c^{\prime \prime \prime } (1+s)^{1-a}/(1-a), \end{aligned}$$

and dividing by *s*. $\quad \square$

### Translated Time Coordinate

Proposition 2.1 implies that $\vert x \vert \le c t +K$ for $t\ge 0$ in $\mathrm {supp}(T^{\mu \nu })$ or, equivalently, $\vert x \vert \le c \tilde{t}$ for $\tilde{t} \ge t_0$, where $t_0 := {K}/{c}$, and $\tilde{t} = t_0 + t$. It is convenient to use this translated time coordinate $\tilde{t}$ in what follows. In particular, the vector fields of Section 4 will be defined using this variable. The main advantage is that the spacetime vector fields $\tilde{Z} = \tilde{\Omega }_{ij}, \tilde{B}_i, \tilde{S}$ defined by

$$\begin{aligned} \tilde{\Omega }_{ij} = \Omega _{ij}, \quad \tilde{B}_i = \tilde{t} \partial _{x^i} + x^i \partial _t, \quad \tilde{S} = \tilde{t} \partial _t + x^k \partial _{x^k} \end{aligned}$$

satisfy, for any multi index *I*,

2.12 $$\begin{aligned} \tilde{t}^{\vert I \vert } \partial ^I = \sum _{\vert J \vert \le \vert I \vert } \Lambda _{IJ} \left( \frac{x}{\tilde{t}} \right) \tilde{Z}^J \quad \text {if } {\vert x \vert }/{\tilde{t}} \le c <1, \end{aligned}$$

for some smooth functions $\Lambda _{IJ}$. Estimates for $\partial ^I \tilde{Z}^J T^{\mu \nu }$ will then follow directly from estimates for $\tilde{Z}^I T^{\mu \nu }$, which are less cumbersome to obtain; see Section 5.4. For simplicity the $\tilde{}$ will always be omitted, and statements just made for $t\ge t_0$.

### Approximations to Geodesics

Define, for $(t,x,{\,\widehat{\!p\!}\,\,}) \in \mathrm {supp}(f)$ and $t_0 \le s \le t$,

2.13 $$\begin{aligned} X_2^i(s,t,x,{\,\widehat{\!p\!}\,\,})= & {} x^i - (t-s) {\,\widehat{\!p\!}\,\,}^i - \int _s^t (s' - s) {\,\widehat{\!\Gamma \!}\,\,}^i \left( s', s' \frac{x}{t}, \frac{x}{t} \right) \,\mathrm{d}s', \nonumber \\ \widehat{P}_2(s,t,x,{\,\widehat{\!p\!}\,\,})= & {} {\,\widehat{\!p\!}\,\,}^i + \int _s^t {\,\widehat{\!\Gamma \!}\,\,}^i \left( s', s' \frac{x}{t}, \frac{x}{t} \right) \,\mathrm{d}s', \end{aligned}$$

and set

$$\begin{aligned} \overline{X}(s,t,x,{\,\widehat{\!p\!}\,\,})^{i}&:= X(s,t,x,{\,\widehat{\!p\!}\,\,})^{i} - X_2(s,t,x,{\,\widehat{\!p\!}\,\,})^i,\\ \overline{P}(s,t,x,{\,\widehat{\!p\!}\,\,})^{i}&:= \frac{\mathrm{d}\overline{X}}{\mathrm{d}s}(s,t,x,{\,\widehat{\!p\!}\,\,}) = {\,\widehat{\!P\!}\,\,}(s,t,x,p)^{i} - {\,\widehat{\!P\!}\,\,}_{\!\!2}(s,t,x,p)^{i} \end{aligned}$$

for $i=1,2,3$. Note that

$$\begin{aligned} \overline{X}(t,t,x,{\,\widehat{\!p\!}\,\,})^i = \overline{P}(t,t,x,{\,\widehat{\!p\!}\,\,})^i = 0 \end{aligned}$$

for $i=1,2,3$. The geodesic equations (2.2) can be used to derive the following equations for $\overline{X}$ and $\overline{P}$:

2.14 $$\begin{aligned} \frac{\mathrm{d} \overline{X}^i}{\mathrm{d}s} =&\, \overline{P}^i; \end{aligned}$$

2.15 $$\begin{aligned} \frac{\mathrm{d} \overline{P}^i}{\mathrm{d}s} =&\, {\,\widehat{\!\Gamma \!}\,\,}^i \left( s, X(s,t,x,{\,\widehat{\!p\!}\,\,}), {\,\widehat{\!P\!}\,\,}(s,t,x,{\,\widehat{\!p\!}\,\,}) \right) - {\,\widehat{\!\Gamma \!}\,\,}^i \left( s, s\frac{x}{t}, \frac{x}{t} \right) . \end{aligned}$$

It follows from the next proposition that the curves $s\mapsto X_2(s,t,x,{\,\widehat{\!p\!}\,\,})$ are good approximations to the geodesics $s \mapsto X(s,t,x,{\,\widehat{\!p\!}\,\,})$. Recall, from Section 1.2.2, the notation $\lesssim$.

#### Proposition 2.3

Suppose $\vert \Gamma (t,x) \vert + t \vert \partial \Gamma (t,x) \vert \lesssim \varepsilon t^{-1-a}$ in $\mathrm {supp}(f)$. Given $t\ge t_0$ such that $(t,x,{\,\widehat{\!p\!}\,\,}) \in \mathrm {supp}(f)$,

2.16 $$\begin{aligned} s^{2a-1} \left| \overline{X}(s,t,x,{\,\widehat{\!p\!}\,\,})^i \right| + s^{2a} \left| \overline{P}(s,t,x,{\,\widehat{\!p\!}\,\,})^i \right| \lesssim \varepsilon \end{aligned}$$

for all $t_0 \le s \le t$ and $i=1,2,3$.

#### Proof

First note that equation (2.2) and the bounds assumed on $\Gamma$ imply

2.17 $$\begin{aligned} \left| \frac{\mathrm{d} {\,\widehat{\!P\!}\,\,}^i(s)}{\mathrm{d}s} \right| \lesssim \frac{\varepsilon }{s^{1+a}} \end{aligned}$$

for $t_0\le s \le t$. Note also that

$$\begin{aligned} X(s,t,x,{\,\widehat{\!p\!}\,\,})^i - s {\,\widehat{\!P\!}\,\,}(s,t,x,{\,\widehat{\!p\!}\,\,})^i = X(s,0,y,q)^i - s{\,\widehat{\!P\!}\,\,}(s,0,y,q)^i, \end{aligned}$$

where $y=X(0,t,x,{\,\widehat{\!p\!}\,\,})$, $q = {\,\widehat{\!P\!}\,\,}(0,t,x,{\,\widehat{\!p\!}\,\,})$. Proposition 2.2, and the integration of (2.17) from *s* to *t* and (2.11) then gives

$$\begin{aligned} \left| \frac{x^i}{t} - \frac{X(s)^i}{s} \right| \le \left| \frac{x^i}{t} - {\,\widehat{\!p\!}\,\,}^i \right| + \left| {\,\widehat{\!p\!}\,\,}^i - {\,\widehat{\!P\!}\,\,}(s)^i \right| + \left| {\,\widehat{\!P\!}\,\,}(s)^i - \frac{X(s)^i}{s} \right| \lesssim \frac{1}{s^{a}} \end{aligned}$$

for $t_0 \le s \le t$ and $i=1,2,3$. Hence

$$\begin{aligned} \left| \, \Gamma ^{\mu }_{\alpha \beta } \left( s, s\frac{x}{t} \right) - \Gamma ^{\mu }_{\alpha \beta } \left( s, X(s) \right) \right| \lesssim \Vert \partial \Gamma ^{\mu }_{\alpha \beta } (s, \cdot ) \Vert _{L^{\infty }} \left| s \frac{x}{t} - X(s) \right| \lesssim \frac{\varepsilon }{s^{1+2a}}. \end{aligned}$$

Moreover,

$$\begin{aligned} \left| \,{\,\widehat{\!P\!}\,\,}(s)^i - \frac{x^i}{t} \right| \le \left| {\,\widehat{\!P\!}\,\,}(s)^i - {\,\widehat{\!p\!}\,\,}^i \right| + \left| {\,\widehat{\!p\!}\,\,}^i - \frac{x^i}{t} \right| \lesssim \frac{1}{s^a} \end{aligned}$$

for $t_0 \le s \le t$, and so

$$\begin{aligned} \left| \, {\,\widehat{\!\Gamma \!}\,\,}^i \left( s, X(s), {\,\widehat{\!P\!}\,\,}(s) \right) - {\,\widehat{\!\Gamma \!}\,\,}^i \left( s, s \frac{x}{t}, \frac{x}{t} \right) \right| \lesssim \frac{\varepsilon }{s^{1+2a}}. \end{aligned}$$

Integrating equation (2.15) backwards from $s=t$, and using the fact that $\overline{P}(t,t,x,{\,\widehat{\!p\!}\,\,})^i = 0$, gives

$$\begin{aligned} \left| \,\overline{P}(s,t,x,{\,\widehat{\!p\!}\,\,})^i \right| \lesssim \frac{\varepsilon }{s^{2a}}, \end{aligned}$$

and integrating the equation (2.14) backwards from $s=t$ and using $\overline{X}(t,t,x,{\,\widehat{\!p\!}\,\,})^i = 0$ gives

$$\begin{aligned} \left| \,\overline{X}(s,t,x,{\,\widehat{\!p\!}\,\,})^i \right| \lesssim \varepsilon s^{1-2a}, \end{aligned}$$

since $$a> \frac{1}{2}$$. $$\quad \square $$

#### Corollary 2.4

Suppose $t\ge t_0$ is such that $(t,x,{\,\widehat{\!p\!}\,\,}) \in \mathrm {supp}(f)$ and $\vert \Gamma (t,x) \vert + t \vert \partial \Gamma (t,x) \vert \lesssim \varepsilon t^{-1-a}$. Then

$$\begin{aligned} \left| \, X_2(s,t,x,{\,\widehat{\!p\!}\,\,})^i \right| \lesssim s \end{aligned}$$

for $$t_0 \le s \le t$$ and $$i=1,2,3$$.

#### Proof

The proof is an immediate consequence of the first bound of (2.7), and Proposition 2.3. $\quad \square$

## The Vector Fields

The general procedure for using approximate geodesics to lift a vector field *Z* on $\mathcal {M}$ to a vector field $\overline{Z}$ on $\mathcal {P}$, outlined in Section 1.5 for geodesics, is described in more detail in Section 3.1. Here a formula for the action of the lifted vector fields on initial conditions for the approximate geodesics is derived. The essential property of the lifted vector fields is that they are bounded when applied to these initial conditions, which one can deduce from this formula. It is however computationally more convenient to define the vector fields from their action on initial conditions for the approximate geodesics, which we do in Section 3.2. Section 3.2 is independent of Section 3.1, but we include Section 3.1 for the purpose of conceptual justification of the vector fields and the formula mentioned above.

### Geometric Construction of Lifted Vector Fields Using Approximate Geodesics

#### Parametrization of Momentum Space with Physical Initial and Final Coordinates for the Approximate Geodesics

Given first approximations to the geodesics $X_1(s,t,x,{\,\widehat{\!p\!}\,\,})$ and ${\,\widehat{\!P\!}\,\,}_{\!\!1}(s,t,x,{\,\widehat{\!p\!}\,\,})$ we define the second approximations $X_2(s,t,x,{\,\widehat{\!p\!}\,\,})$ and ${\,\widehat{\!P\!}\,\,}_{\!\!2}(s,t,x,{\,\widehat{\!p\!}\,\,})$ to the geodesics through $(t,x,{\,\widehat{\!p\!}\,\,})$, to be the solutions of the system

3.1 $$\begin{aligned} \frac{\mathrm{d}}{\mathrm{d}s} {X}_2={\,\widehat{\!P\!}\,\,}_{\!2},\quad \frac{\mathrm{d}}{\mathrm{d}s} {\,\widehat{\!P\!}\,\,}_{\!2}={\Gamma }(X_1)\cdot \Lambda ({\,\widehat{\!P\!}\,\,}_{\!1\!}),\quad X_2(t)=x,\quad {\,\widehat{\!P\!}\,\,}_{\!2}(t)={\,\widehat{\!p\!}\,\,}. \end{aligned}$$

Under some mild assumptions on the metric *g*,^10^ for fixed $\tau$ any point $(t,x,{\,\widehat{\!p\!}\,\,}) \in \mathcal {P}$ with $t >\tau$ can be described uniquely by the pair of points $\{ (t,x), (\tau ,y) \}$ in $\mathcal {M}$, where

3.2 $$\begin{aligned} y=X_2(\tau ,t,x,{\,\widehat{\!p\!}\,\,}), \end{aligned}$$

is the point where the approximate geodesic $X_2$ emanating from (*t*, *x*) with velocity ${\,\widehat{\!p\!}\,\,}$ intersects the hypersurface $\Sigma _{\tau }$, that is $(t,x,{\,\widehat{\!p\!}\,\,}) \in \mathcal {P}$ can be parameterised by $\{ (t,x), (\tau ,y) \}$,

$$\begin{aligned} (t,x,{\,\widehat{\!p\!}\,\,}) = (t,x,{\,\widehat{\!p\!}\,\,}_{X_2}(t,x,\tau ,y)), \end{aligned}$$

where the subscript $X_2$ is used in ${\,\widehat{\!p\!}\,\,}_{X_2}(t,x,\tau ,y)$ to emphasise that ${\,\widehat{\!p\!}\,\,}$ is now parametrised by *y* using the approximations $X_2$ to the geodesics. Integrating (3.1) backwards from *t* to $t_0$ gives that ${\,\widehat{\!p\!}\,\,}={\,\widehat{\!p\!}\,\,}_{X_2}(t,x,t_0,y)$ is implicitly given by

3.3 $$\begin{aligned} y=x - (t-t_0){\,\widehat{\!p\!}\,\,}- (t-t_0)\Theta (t_0,t,x,{\,\widehat{\!p\!}\,\,}), \end{aligned}$$

where

3.4 $$\begin{aligned} \Theta (t_0,t,x,{\,\widehat{\!p\!}\,\,})^i= \int _{t_0}^t \frac{s-t_0}{t-t_0}\,{\Gamma }\big ( s, X_1(s,t,x,{\,\widehat{\!p\!}\,\,})\big )\cdot \Lambda \big ({\,\widehat{\!P\!}\,\,}_{\!\!1}(s,t,x,{\,\widehat{\!p\!}\,\,})\big )^i\, \mathrm{d}s. \end{aligned}$$

Here we used Taylor’s formula with integral remainder $f(t_0)=f(t)+f^\prime (t)(t_0-t)+\int _t^{t_0}(t_0-s)f^{\prime \prime }(s)\, \mathrm{d}s$. The first approximate geodesics in the previous section are independent of ${\,\widehat{\!p\!}\,\,}$ so then $\Theta$ is independent of ${\,\widehat{\!p\!}\,\,}$ and the above relation in fact gives ${\,\widehat{\!p\!}\,\,}_{X_2}$ explicitly.

#### Lifting of Physical Vector Fields to Vector Fields on Momentum Space Adapted to the Approximate Geodesics

For a given vector field *Z* on $\mathcal {M}$, let $\Phi ^Z_{\lambda } : \mathcal {M} \rightarrow \mathcal {M}$ denote the associated one parameter family of diffeomorphisms, so $\Phi ^Z_{0}=Id$ and so that

$$\begin{aligned} \frac{\mathrm{d} \Phi ^Z_{\lambda }(t,x)}{\mathrm{d}\lambda } = Z\vert _{\Phi ^Z_{\lambda }(t,x)}. \end{aligned}$$

Now the action of $\Phi ^Z_{\lambda }$ on (*t*, *x*) and $(\tau ,y)$ in $\mathcal {M}$ induces an action on $(t,x,{\,\widehat{\!p\!}\,\,})$ in $\mathcal {P}$, given by

$$\begin{aligned}&\overline{\Phi }^{Z,X_2}_{\lambda ,\tau }(t,x,{\,\widehat{\!p\!}\,\,}) := \left( \Phi ^Z_{\lambda }(t,x), {\,\widehat{\!p\!}\,\,}_{X_2}\left( \Phi ^Z_{\lambda }(t,x), \Phi ^Z_{\lambda }(\tau ,y) \right) \right) ,\\&\quad \text {where}\quad y=X_2(\tau ,t,x,{\,\widehat{\!p\!}\,\,}). \end{aligned}$$

For fixed $t_0$ we define the vector field $\overline{Z}$ by

$$\begin{aligned} \overline{Z}\vert _{(t,x,{\,\widehat{\!p\!}\,\,})} = \frac{\mathrm{d} \overline{\Phi }^{Z,X_2}_{\lambda ,\tau }(t,x,{\,\widehat{\!p\!}\,\,})}{\mathrm{d}\lambda } \bigg \vert _{\lambda = 0,\,\tau =t_0}. \end{aligned}$$

We have

3.5 $$\begin{aligned} \overline{Z}f(t,x,{\,\widehat{\!p\!}\,\,})= & {} \frac{\mathrm{d}}{\mathrm{d}\lambda } f\left( \Phi ^Z_{\lambda }(t,x), {\,\widehat{\!p\!}\,\,}_{X_2}\left( \Phi ^Z_{\lambda }(t,x), \Phi ^Z_{\lambda }(t_0,y) \right) \right) \bigg \vert _{\lambda = 0}\nonumber \\= & {} Z^\alpha \partial _{x^\alpha } f(t,x,{\,\widehat{\!p\!}\,\,})+\frac{\mathrm{d}}{\mathrm{d}\lambda } f\left( t,x, {\,\widehat{\!p\!}\,\,}(\lambda )\right) \bigg \vert _{\lambda = 0}, \end{aligned}$$

where

3.6 $$\begin{aligned} {\,\widehat{\!p\!}\,\,}(\lambda )={\,\widehat{\!p\!}\,\,}_{X_2}\left( \Phi ^Z_{\lambda }(t,x), \Phi ^Z_{\lambda }(t_0,y) \right) ,\quad \text {and}\quad y=X_2(t_0,t,x,{\,\widehat{\!p\!}\,\,}),\quad {\,\widehat{\!p\!}\,\,}(0)={\,\widehat{\!p\!}\,\,}. \end{aligned}$$

#### The Lifted Vector Fields Applied to the Initial Conditions of the Approximate Geodesics Parameterized by the Final Momentum Space

A computation shows that

3.7 $$\begin{aligned} \overline{Z}\vert _{(t,x,{\,\widehat{\!p\!}\,\,})} \left( X_2(t_0,t,x,{\,\widehat{\!p\!}\,\,})^i \right) = Z^i \vert _{(t_0,X_2(t_0))} - Z^0 \vert _{(t_0,X_2(t_0))} {\,\widehat{\!P\!}\,\,}_{\!2}(t_0,t,x,{\,\widehat{\!p\!}\,\,})^i, \end{aligned}$$

which as desired is bounded independently of $t>t_0+1$. In fact, if $y=X_2^i(t_0,t,x,{\,\widehat{\!p\!}\,\,})$ and $q={\,\widehat{\!P\!}\,\,}_2^i(t_0,t,x,{\,\widehat{\!p\!}\,\,})$ are bounded in the support of $f(t_0,y,q)$, equation (3.7) guarantees that $\vert \overline{Z}(X_2(t_0,t,x,{\,\widehat{\!p\!}\,\,})^i) \vert$ is bounded.

To see that (3.7) indeed holds, first note that the left hand side is the derivative of $X_2\big (t_0,\overline{\Phi }^{Z,X_2}_{\lambda ,t_0}(t,x,{\,\widehat{\!p\!}\,\,})\big )^i$ with respect to $\lambda$ at $\lambda =0$. Also the first term on the right hand side is the derivative of $\Phi ^Z_{\lambda }(t_0,y)^i$ at $\lambda =0$. The equality (3.7) follows from taking the derivative, with respect to $\lambda$ at $\lambda =0$ of both sides of the identity

3.8 $$\begin{aligned} X_2\big (\Phi ^Z_{\lambda }(t_0,y)^0,\overline{\Phi }^{Z,X_2}_{\lambda ,t_0}(t,x,{\,\widehat{\!p\!}\,\,})\big )^i =\Phi ^Z_{\lambda }(t_0,y)^i. \end{aligned}$$

The identity (3.8) is just two different ways of expressing that we apply the transformation $\Phi _\lambda$ to the initial point of the path $(t_0,y)$. The left hand side is that we apply the transformation to the approximate geodesic which will lead to the transformation of the initial point.

### Computation of the Lifted Vector Fields from Their Action on Initial Conditions for the Approximate Geodesics

In this section we will compute the vector fields from how they act of the initial conditions. We will use a slight modification of the formula for how they act on the initial conditions derived in the previous section and we will hence get a slightly modification of vector fields in the previous section. They are computationally slightly simpler to use than the vector fields of the previous section, though they are mildly singular for *t* close to $t_0$. It is therefore assumed throughout this section that $t\ge t_0+1$, and the vector fields will only be used in the proof of Theorem 1.3 under this assumption.

The vector fields can be derived by imposing that they have the form

3.9 $$\begin{aligned} \overline{Z}=Z^\mu \partial _{x^\mu }+\widetilde{Z}^i\partial _{{\,\widehat{\!p\!}\,\,}^i}, \end{aligned}$$

where $\widetilde{Z}^i$ are to be determined, and insisting that the relation

3.10 $$\begin{aligned} \overline{Z}\vert _{(t,x,{\,\widehat{\!p\!}\,\,})} \left( X_2(t_0,t,x,{\,\widehat{\!p\!}\,\,})^i \right) = Z^i \vert _{(t_0,X_2(t_0))} - Z^0 \vert _{(t_0,X_2(t_0))} {\,\widehat{\!p\!}\,\,}^i \end{aligned}$$

holds, instead of the relation (3.7) which involves ${\,\widehat{\!P\!}\,\,}_{\!\!2}(t_0,t,x,{\,\widehat{\!p\!}\,\,})^i$ instead of ${\,\widehat{\!p\!}\,\,}^i$ and is satisfied by the vector fields of the previous section. Equation (3.10) is in particular true for the Minkowski vector fields $\overline{Z}^M$.

We now make the further assumption that the first approximate geodesics $X_1$ and ${\,\widehat{\!P\!}\,\,}_{\!\!1}$ in (3.1) are independent of ${\,\widehat{\!p\!}\,\,}$ in which case by (3.3) the second approximation to the geodesics are given by

3.11 $$\begin{aligned} X_2(t_0,t,x,{\,\widehat{\!p\!}\,\,})=y=x - (t-t_0){\,\widehat{\!p\!}\,\,}- (t-t_0)\Theta (t_0,t,x), \end{aligned}$$

with $\Theta$ given by (3.4) now independent of ${\,\widehat{\!p\!}\,\,}$. Applying the expression (3.9) to (3.11) gives

3.12 $$\begin{aligned} \overline{Z}\big (X_2(t_0,t,x,{\,\widehat{\!p\!}\,\,})^i\big )=Z^i\vert _{(t,x)} - Z^0\vert _{(t,x)}{\,\widehat{\!p\!}\,\,}^i- Z\big ((t-t_0)\Theta (t_0,t,x)^i\big )-(t-t_0)\widetilde{Z}^i\vert _{(t,x,{\,\widehat{\!p\!}\,\,})}, \end{aligned}$$

and so $\widetilde{Z}^i$ is indeed determined if $\overline{Z}\big (X_2(t_0,t,x,{\,\widehat{\!p\!}\,\,})^i\big )$ is prescribed for each *i*. Substituting the definition (3.10) into (3.12) and solving for $\widetilde{Z}^i$ we obtain

3.13 $$\begin{aligned} \widetilde{Z}^i\vert _{(t,x,{\,\widehat{\!p\!}\,\,})}= & {} \frac{1}{t-t_0} \Big (Z^i\vert _{(t,x)}-Z^i \vert _{(t_0,X_2(t_0))}-\big (Z^0\vert _{(t,x)}-Z^0 \vert _{(t_0,X_2(t_0))} \big ){\,\widehat{\!p\!}\,\,}^i\nonumber \\&\quad -Z\big ((t-t_0)\Theta (t_0,t,x)^i\big )\Big ). \end{aligned}$$

The coefficients of the vector fields we are considering are linear functions

3.14 $$\begin{aligned} Z^\alpha =C^\alpha _\beta x^\beta , \end{aligned}$$

where $x^0=t$. Hence, by (3.11),

3.15 $$\begin{aligned} \frac{1}{t-t_0} \Big (Z^\alpha \vert _{(t,x)}-Z^\alpha \vert _{(t_0,X_2(t_0))}\Big ) =C^{\alpha }_\beta \frac{ x^\beta -y^\beta }{t-t_0} =C^{\alpha }_\beta ({\,\widehat{\!p\!}\,\,}^\beta +\Theta ^\beta ), \end{aligned}$$

where ${\,\widehat{\!p\!}\,\,}^0=1$ and we defined $\Theta ^0=0$. Hence

3.16 $$\begin{aligned} \widetilde{Z}^i=C^i_\beta ({\,\widehat{\!p\!}\,\,}^\beta +\Theta ^\beta )-C^0_\beta ({\,\widehat{\!p\!}\,\,}^\beta +\Theta ^\beta ){\,\widehat{\!p\!}\,\,}^i-(t-t_0)^{-1}Z\big ((t-t_0)\Theta (t_0,t,x)^i\big ). \end{aligned}$$

Substituting $X_1=sx/t$ and ${\,\widehat{\!P\!}\,\,}_{\! 1}=x/t$ into (3.4) we have

#### Lemma 3.1

If

3.17 $$\begin{aligned} \Theta (t_0,t,x)^i= \int _{t_0}^t \frac{s-t_0}{t-t_0}\,{\Gamma }\big ( s, sx/t\big )\cdot \Lambda \big (x/t\big )^i\, \mathrm{d}s, \end{aligned}$$

then

3.18 $$\begin{aligned} (t-t_0)^{-1} Z\big ((t-t_0) \Theta (t_0,t,x)^i\big ) = t\,{\Gamma }\big ( t, x\big )\cdot \Lambda \big (x/t\big )^i(Z^0 \!/t)+ \Theta _Z(t_0,t,x)^i, \end{aligned}$$

where, with $$(Z\Lambda )(x/t)=Z \big (\Lambda (x/t)\big )$$,

3.19 $$\begin{aligned} \Theta _Z(t_0,t,x)^i= & {} \int _{t_0}^t\frac{s-t_0}{t-t_0} \Big ((Z{\Gamma })\big ( s, sx/t\big )\cdot \Lambda \big (x/t\big )^i\nonumber \\&-(S\Gamma \big )\big ( s, sx/t\big )\cdot \Lambda \big (x/t\big )^i(Z^0 \!/t)\nonumber \\&+{\Gamma }\big ( s, sx/t\big )\cdot (Z\Lambda )\big (x/t\big )^i\Big )\, \mathrm{d}s. \end{aligned}$$

#### Proof

Changing variables gives

3.20 $$\begin{aligned} (t-t_0) \Theta (t_0,t,x)^i= & {} \int _{t_0}^t (s-t_0)\,{\Gamma }\big ( s, sx/t\big )\cdot \Lambda \big (x/t\big )^i\, \mathrm{d}s\nonumber \\= & {} \int _{t_0/t}^1(ts'-t_0) t\,{\Gamma }\big ( s't, s'x\big )\cdot \Lambda \big (x/t\big )^i\, \mathrm{d}s'. \end{aligned}$$

Hence

3.21 $$\begin{aligned} Z\big ((t-t_0) \Theta (t_0,t,x)^i\big )= & {} \int _{t_0}^t s\,{\Gamma }\big ( s, sx/t\big )\cdot \Lambda \big (x/t\big )^i(Z^0 \!/t)\, \mathrm{d}s\nonumber \\&+\int _{t_0}^t (s-t_0)\,{\Gamma }\big ( s, sx/t\big )\cdot \Lambda \big (x/t\big )^i (Z^0\! /t)\, \mathrm{d}s\nonumber \\&+\int _{t_0}^t (s-t_0)\,\Big ((Z{\Gamma })\big ( s, sx/t\big )\cdot \Lambda \big (x/t\big )^i\nonumber \\&+{\Gamma }\big ( s, sx/t\big )\cdot (Z\Lambda )\big (x/t\big )^i\Big )\, \mathrm{d}s. \end{aligned}$$

Integrating by parts we have

3.22 $$\begin{aligned}&\int _{t_0}^t \!\!\! s\,{\Gamma }\big ( s, sx/t\big )\cdot \Lambda \big (x/t\big )^i(Z^0 \!/t)\, \mathrm{d}s =(t-t_0)\,t\,{\Gamma }\big ( t, x\big )\cdot \Lambda \big (x/t\big )^i(Z^0 \!/t) \nonumber \\&\quad -\int _{t_0}^t\!\!\! (s-t_0) \big ( {\Gamma }+S\Gamma \big )\big ( s, sx/t\big )\cdot \Lambda \big (x/t\big )^i(Z^0 \!/t)\, \mathrm{d}s, \end{aligned}$$

and adding things up we get the lemma. $\quad \square$

#### Remark 3.2

We note $\Theta _Z$ is of exactly the same form of $\Theta$ and hence can be estimated in the same way, and moreover a vector field $Z'$ applied to $\Theta _Z$ will produce a $(\Theta _X)_{Z^\prime }$ of the same form.

One can further obtain bounds for $\overline{Z}$ applied to the initial conditions for ${\,\widehat{\!P\!}\,\,}$ as follows: we have

3.23 $$\begin{aligned} {\,\widehat{\!P\!}\,\,}_{\!2}(t_0,t,x,{\,\widehat{\!p\!}\,\,})^i={\,\widehat{\!p\!}\,\,}^i-\Psi (t_0,t,x)^i,\quad \text {where}\quad \Psi (t_0,t,x)^i= \int _{t_0}^t \,{\Gamma }\big ( s, sx/t\big )\cdot \Lambda \big (x/t\big )^i\, \mathrm{d}s, \end{aligned}$$

so

3.24 $$\begin{aligned} \overline{Z}\vert _{(t,x,{\,\widehat{\!p\!}\,\,})} \left( {\,\widehat{\!P\!}\,\,}_2(t_0,t,x,{\,\widehat{\!p\!}\,\,})^i \right) = \widetilde{Z}^i \vert _{(t,x,{\,\widehat{\!p\!}\,\,})} - Z^\mu \vert _{(t,x,{\,\widehat{\!p\!}\,\,})} \partial _{x^\mu } \Psi (t_0,t,x)^i \end{aligned}$$

which again is bounded, provided the components of *Z* grow at most like *t*.

#### The Rotation Vector Fields

Since the rotations satisfy $Z^0=0$ it easily follows from the above lemma that

$$\begin{aligned} \overline{\Omega }_{ij}=x^i\partial _{x^j}-x^j\partial _{x^i} +({\,\widehat{\!p\!}\,\,}^i +\Theta ^i)\partial _{{\,\widehat{\!p\!}\,\,}^j} -({\,\widehat{\!p\!}\,\,}^j +\Theta ^j)\partial _{{\,\widehat{\!p\!}\,\,}^i} -\Theta _{\Omega _{ij}}^\ell \partial _{{\,\widehat{\!p\!}\,\,}^\ell }, \end{aligned}$$

where $\Theta$ is given by (3.17) and

$$\begin{aligned} \Theta _{\Omega _{ij}}(t_0,t,x) {=}\int _{t_0}^t \frac{s-t_0}{t-t_0}\Big ((\Omega _{ij} {\Gamma })(s,sx/t)\cdot \Lambda (x/t) {+} {\Gamma }\big (s,s\frac{x}{t}\big )\cdot (\Omega _{ij}\Lambda )\big (\frac{x}{t}\big )\Big )\, \mathrm{d}s. \end{aligned}$$

#### The Scaling Vector Field

By the above lemma we have

3.25 $$\begin{aligned} \overline{S}=t\partial _t+x^i\partial _{x^i} + \big (\Theta ^i- t\,{\Gamma }\big ( t, x\big )\cdot \Lambda \big (x/t\big )^i\big )\partial _{{\,\widehat{\!p\!}\,\,}^i}. \end{aligned}$$

#### The Boost Vector Fields

By the above lemma we have

3.26 $$\begin{aligned} \overline{B}_i=x^i\partial _t+t\partial _{x^i}+\Big (\delta ^{ij}-({\,\widehat{\!p\!}\,\,}^i+\Theta ^i){\,\widehat{\!p\!}\,\,}^j -t\,{\Gamma }\big ( t, x\big )\cdot \Lambda \big (x/t\big )^j(x^i \!/t)-\Theta _{B_i}^j\Big )\partial _{{\,\widehat{\!p\!}\,\,}^j}, \end{aligned}$$

where

3.27 $$\begin{aligned} \Theta _{B_i}^j= & {} \int _{t_0}^t\frac{s-t_0}{t-t_0} \Big ((B_i{\Gamma })\big ( s, sx/t\big )\cdot \Lambda \big (x/t\big )^j-(S\Gamma \big )\big ( s, sx/t\big )\cdot \Lambda \big (x/t\big )^j(x^i\!/t)\nonumber \\&+{\Gamma }\big ( s, sx/t\big )\cdot (B_i\Lambda )\big (x/t\big )^j\Big )\, \mathrm{d}s. \end{aligned}$$

## Vector Fields Applied to Geodesics

Recall from Section 2 the definitions (2.13) of $X_2(s,t,x,{\,\widehat{\!p\!}\,\,})^i$ and ${\,\widehat{\!P\!}\,\,}_{\!\!2}(s,t,x,{\,\widehat{\!p\!}\,\,})^i$, and

$$\begin{aligned} \overline{X}(s,t,x,{\,\widehat{\!p\!}\,\,})^{i}&:= X(s,t,x,{\,\widehat{\!p\!}\,\,})^{i} - X_2(s,t,x,{\,\widehat{\!p\!}\,\,})^i,\\ \overline{P}(s,t,x,{\,\widehat{\!p\!}\,\,})^{i}&:= \frac{\mathrm{d}\overline{X}}{\mathrm{d}s}(s,t,x,{\,\widehat{\!p\!}\,\,}) = {\,\widehat{\!P\!}\,\,}(s,t,x,{\,\widehat{\!p\!}\,\,})^{i} - {\,\widehat{\!P\!}\,\,}_{\!\!2}(s,t,x,{\,\widehat{\!p\!}\,\,})^{i}. \end{aligned}$$

In this section estimates for combinations of appropriate vector fields, $\overline{Z}^I$ (see Sections 3 and 4.1 below), applied to the geodesics $X(t_0,t,x,{\,\widehat{\!p\!}\,\,})$ and ${\,\widehat{\!P\!}\,\,}(t_0,t,x,{\,\widehat{\!p\!}\,\,})$ of a fixed spacetime satisfying the pointwise bounds

4.1 $$\begin{aligned} \left| Z^I \Gamma ^{\alpha }_{\beta \gamma }(t',x') \right| \lesssim \frac{\varepsilon }{(t')^{1+a}} \quad \text {for } t_0 \le t' \le t, \text { } \vert x' \vert \le c t', \text { and } \vert I \vert \le \left\lfloor \frac{N}{2} \right\rfloor + 2, \end{aligned}$$

where $\frac{1}{2}< a < 1$, are obtained. The main results are Proposition 4.24 and Corollary 4.27, which will be used in Section 5 to estimate combinations of the spacetime vector fields, $Z^I$, applied to the components of the energy momentum tensor, $T^{\mu \nu }(t,x)$.

Proposition 4.24 and Corollary 4.27 are obtained by applying $\overline{Z}^I$ to the system (2.14)–(2.15) (see Propositions 4.23 and 4.26) to first estimate $\overline{Z}^I(\overline{X}(t_0)^k)$ and $\overline{Z}^I(\overline{P}(t_0)^k)$. Proposition 4.24 and Corollary 4.27 then follow from good estimates for $\overline{Z}^I(X_2(t_0)^k)$ and $\overline{Z}^I({\,\widehat{\!P\!}\,\,}_2(t_0)^k)$. The vector fields $\overline{Z}$ are defined so that such good estimates indeed hold (see Proposition 4.1 for the case $\vert I \vert = 1$, and Proposition 4.9 for general *I*).

In Section 4.1 the vector fields are recalled from Section 3. In Section 4.2 the basic idea of the remainder of the section is illustrated by considering only one rotation vector field $\overline{\Omega }_{ij}$ applied to the geodesics. In Section 4.3 schematic expressions for $\overline{Z}^I(X_2(t_0)^k)$, along with $\overline{Z}^I$ applied to $\frac{x^i}{t} - {\,\widehat{\!p\!}\,\,}^i$ and ${\,\widehat{\!p\!}\,\,}^i$ (estimates of which are also used in the proof of Propositions 4.23 and 4.26) are obtained. In Section 4.4 these schematic expressions are used to obtain estimates for $\overline{Z}^I(X_2(t_0)^k)$, and for $\overline{Z}^I$ applied to various other quantities. Since the commuted system (2.14)–(2.15) is integrated backwards from $s=t$, it is necessary to first estimate the “final conditions”, $\overline{Z}^I(\overline{X}(s,t,x,{\,\widehat{\!p\!}\,\,})^k)\vert _{s=t}$ and $\overline{Z}^I(\overline{P}(s,t,x,{\,\widehat{\!p\!}\,\,})^k)\vert _{s=t}$. Such estimates are obtained in Section 4.7 (see Propositions 4.21 and 4.22) using the results of Sections 4.5 and 4.6. In Section 4.8 lower order derivatives of $\overline{X}(s)^k$ and $\overline{P}(s)^k$ are estimated (Proposition 4.23) and in Section 4.9 higher order derivatives are estimated (Proposition 4.26).

In Sections 4.1–4.9 it will be assumed that $t\ge t_0+1$. Section 4.10 is concerned with the case $t_0 \le t \le t_0 + 1$. It will be assumed throughout this section that $\vert x \vert \le c t$, where $0<c<1$ (recall the discussion in Section 2.2).

### Vector Fields

The proof of the main result uses the following collection of vector fields, introduced in Section 3, which are schematically denoted $\overline{Z}$

$$\begin{aligned} \overline{Z} = \overline{\Omega }_{ij}, \overline{B}_i, \overline{S} \end{aligned}$$

for $i,j=1,2,3$, $i<j$, and reduce to the standard rotations, boosts and scaling vector field of Minkowski space when acting on spacetime functions. Recall the notation, for $i=1,2,3$,

$$\begin{aligned} {\,\widehat{\!\Gamma \!}\,\,}(t,x,{\,\widehat{\!p\!}\,\,})^i = {\,\widehat{\!p\!}\,\,}^i\Gamma _{\alpha \beta }^0(t,x){\,\widehat{\!p\!}\,\,}^\alpha {\,\widehat{\!p\!}\,\,}^\beta -\Gamma _{\alpha \beta }^i(t,x){\,\widehat{\!p\!}\,\,}^{\alpha }{\,\widehat{\!p\!}\,\,}^\beta , \end{aligned}$$

and

4.2 $$\begin{aligned} \Theta ^i(t,x) = \frac{1}{t-t_0} \int _{t_0}^t (s' - t_0) {\,\widehat{\!\Gamma \!}\,\,}^i \left( s',s'\frac{x}{t},\frac{x}{t} \right) \,\mathrm{d}s'. \end{aligned}$$

Recall that the vector fields $\overline{\Omega }_{ij}, \overline{B}_i, \overline{S}$ take the following form: first,

$$\begin{aligned} \overline{\Omega }_{ij}\vert _{(t,x,p)} = x^i \partial _{x^j} - x^j \partial _{x^i} + {\,\widehat{\!p\!}\,\,}^i \partial _{{\,\widehat{\!p\!}\,\,}^j} - {\,\widehat{\!p\!}\,\,}^j \partial _{{\,\widehat{\!p\!}\,\,}^i} + \mathring{\Omega }_{ij}^k \partial _{{\,\widehat{\!p\!}\,\,}^k}, \end{aligned}$$

where

4.3 $$\begin{aligned} \mathring{\Omega }_{ij}^k\vert _{(t,x,p)} =&\,\frac{1}{t-t_0} \Bigg [ \int _{t_0}^t (s'-t_0) \left( {\,\widehat{\!\Gamma \!}\,\,}^i \left( s', s' \frac{x}{t}, \frac{x}{t} \right) \delta _j^k - {\,\widehat{\!\Gamma \!}\,\,}^j \left( s', s' \frac{x}{t}, \frac{x}{t} \right) \delta _i^k \right) \,\mathrm{d}s' \end{aligned}$$

4.4 $$\begin{aligned}&- (x^i \partial _{x^j} - x^j \partial _{x^i}) \left( \int _{t_0}^t (s'-t_0) {\,\widehat{\!\Gamma \!}\,\,}^k \left( s', s' \frac{x}{t}, \frac{x}{t} \right) \, \mathrm{d}s' \right) \Bigg ] \nonumber \\ =&\, \Theta ^i(t,x) \delta _j^k - \Theta ^j(t,x) \delta _i^k - \Omega _{ij} \left( \Theta ^k(t,x) \right) \end{aligned}$$

for $$1 \le i < j \le 3$$. Second,

$$\begin{aligned} \overline{B}_i\vert _{(t,x,p)} = x^i \partial _t + t\partial _{x^i} + \left( \delta _i^j - {\,\widehat{\!p\!}\,\,}^i {\,\widehat{\!p\!}\,\,}^j \right) \partial _{{\,\widehat{\!p\!}\,\,}^j} + \mathring{B}_{i}^k \partial _{{\,\widehat{\!p\!}\,\,}^k}, \end{aligned}$$

where

4.5 $$\begin{aligned} \mathring{B}_{i}^k\vert _{(t,x,p)} =&\, - \frac{1}{t - t_0} \bigg [ \int _{t_0}^t (s' - t_0) {\,\widehat{\!\Gamma \!}\,\,}^i \left( s', s'\frac{x}{t}, \frac{x}{t} \right) \, \mathrm{d}s' {\,\widehat{\!p\!}\,\,}^k \nonumber \\&+ (x^i \partial _t + t \partial _{x^i}) \left( \int _{t_0}^t (s' - t_0) {\,\widehat{\!\Gamma \!}\,\,}^k \left( s', s'\frac{x}{t}, \frac{x}{t} \right) \,\mathrm{d}s' \right) \bigg ] \nonumber \\ =&\,- \Theta ^i(t,x) {\,\widehat{\!p\!}\,\,}^k - B_i \left( \Theta ^k(t,x) \right) - \frac{x^i}{t-t_0} \Theta ^k(t,x) \end{aligned}$$

for $$i=1,2,3$$. Finally,

$$\begin{aligned} \overline{S}\vert _{(t,x,p)} = t \partial _t + x^i \partial _{x^i} + \mathring{S}^k \partial _{{\,\widehat{\!p\!}\,\,}^k}, \end{aligned}$$

where

4.6 $$\begin{aligned} \mathring{S}^k\vert _{(t,x,p)}&= \frac{1}{t-t_0} \bigg [ \int _{t_0}^t (s' - t_0) {\,\widehat{\!\Gamma \!}\,\,}^k \left( s', s'\frac{x}{t}, \frac{x}{t} \right) \,\mathrm{d}s' \nonumber \\&\quad - (t \partial _t + x^i \partial _{x^i}) \left( \int _{t_0}^t (s' - t_0) {\,\widehat{\!\Gamma \!}\,\,}^k \left( s', s'\frac{x}{t}, \frac{x}{t} \right) \,\mathrm{d}s' \right) \bigg ] \nonumber \\&= \Theta ^k(t,x) - S \left( \Theta ^k(t,x) \right) - \frac{t}{t-t_0} \Theta ^k(t,x). \end{aligned}$$

By the bound (2.16), the maps $X_2(s,t,x,{\,\widehat{\!p\!}\,\,})^i$ are good approximations to the true geodesics, $X(s,t,x,{\,\widehat{\!p\!}\,\,})^i$. **Recall that the vector fields**$\overline{\Omega }_{ij}$, $\overline{B}_i$, $\overline{S}$**are defined so that, when applied to**$X_2(s,t,x,{\,\widehat{\!p\!}\,\,})^i$**the following hold at**$s=t_0$:

#### Proposition 4.1

For $(t,x,p) \in \mathrm {supp}(f)$, the vector fields $\overline{\Omega }_{ij}$, $\overline{B}_i$, $\overline{S}$ defined above satisfy that

4.7 $$\begin{aligned} \overline{\Omega }_{ij} \left( X_2(t_0,t,x,{\,\widehat{\!p\!}\,\,})^k \right)&= X_2(t_0,t,x,{\,\widehat{\!p\!}\,\,})^i \delta _j^k - X_2(t_0,t,x,{\,\widehat{\!p\!}\,\,})^j \delta _i^k, \end{aligned}$$

4.8 $$\begin{aligned} \overline{B}_i \left( X_2(t_0,t,x,{\,\widehat{\!p\!}\,\,})^k \right)&= t_0 \delta _i^k - X_2(t_0,t,x,{\,\widehat{\!p\!}\,\,})^i {\,\widehat{\!p\!}\,\,}^k, \end{aligned}$$

4.9 $$\begin{aligned} \overline{S} \left( X_2(t_0,t,x,{\,\widehat{\!p\!}\,\,})^k \right)&= X_2(t_0,t,x,{\,\widehat{\!p\!}\,\,})^k - t_0 {\,\widehat{\!p\!}\,\,}^k, \end{aligned}$$

for $$i,j,k = 1,2,3$$.

#### Proof

The proof is a straightforward computation. See also Section 3.2, where the above form of the vector fields $\overline{\Omega }_{ij}$, $\overline{B}_i$, $\overline{S}$ are derived by insisting that the result of this proposition is true. (Note that (4.7)–(4.9) are nothing other than equation (3.10) specialised to the case $\overline{Z} = \overline{\Omega }_{ij}, \overline{B}_i, \overline{S}$ respectively. See also equation (1.40) and the general discussion in Section 1.5.) $\quad \square$

#### Remark 4.2

Recall the Minkowski vector fields $\overline{\Omega }_{ij}^M$, $\overline{B}_{i}^M$, $\overline{S}^M$ from Section 1.5.1. Since, in Minkowski space, the maps $X(s,t,x,{\,\widehat{\!p\!}\,\,})^i$ are simply given by

$$\begin{aligned} X_M(s,t,x,{\,\widehat{\!p\!}\,\,})^i = x^i - (t-s){\,\widehat{\!p\!}\,\,}^i \end{aligned}$$

for $i=1,2,3$, it is easy to see that Proposition 4.1 in fact holds in Minkowski space with $t_0$ replaced by any *s*:

$$\begin{aligned} \overline{\Omega }_{ij}^M \left( X_M(s)^k \right)= & {} X_M(s)^i \delta _j^k - X_M(s)^j \delta _i^k, \quad \overline{B}_{i}^M \left( X_M(s)^k \right) = s \delta _i^k - X_M(s)^i {\,\widehat{\!p\!}\,\,}^k,\\ \overline{S}^M \left( X_M(s)^k \right)= & {} X_M(s)^k - s {\,\widehat{\!p\!}\,\,}^k. \end{aligned}$$

### Estimates for One Rotation Vector Field Applied to the Geodesics

Recall that the main goal of Section 4 is to control combinations of vector fields applied to the components of $X(t_0,t,x,{\,\widehat{\!p\!}\,\,})$ and ${\,\widehat{\!P\!}\,\,}(t_0,t,x,{\,\widehat{\!p\!}\,\,})$, that is to prove Proposition 4.24 and Corollary 4.27. Proposition 4.24 and Corollary 4.27 require introducing schematic notation and first obtaining preliminary results (see Sections 4.3–4.7). The main idea, however, is straightforward and can already be understood. In order to illustrate the idea, in this section it is shown how to estimate one rotation vector applied to the components of $X(t_0,t,x,{\,\widehat{\!p\!}\,\,})$ and ${\,\widehat{\!P\!}\,\,}(t_0,t,x,{\,\widehat{\!p\!}\,\,})$. This section is included for the purpose of exposition and the results are not directly used in the proof of Proposition 4.24 and Corollary 4.27 or elsewhere.

By Proposition 4.1, in order to control $\overline{\Omega }_{ij}$ applied to the components of $X(t_0,t,x,{\,\widehat{\!p\!}\,\,})$ and ${\,\widehat{\!P\!}\,\,}(t_0,t,x,{\,\widehat{\!p\!}\,\,})$ it suffices to control $\overline{\Omega }_{ij}$ applied to the components of $\overline{X}(t_0,t,x,{\,\widehat{\!p\!}\,\,})$ and $\overline{P}(t_0,t,x,{\,\widehat{\!p\!}\,\,})$ or, more generally, to show the following:

#### Proposition 4.3

Suppose $t\ge t_0 + 1$, $\vert x \vert \le c t$, $(t,x,{\,\widehat{\!p\!}\,\,}) \in \mathrm {supp}(f)$ and the bounds (4.1) hold with $N = 0$. Then, for $i,j,k=1,2,3$, $i\ne j$,

$$\begin{aligned} s^{2a - 1} \left| \overline{\Omega }_{ij} \left( \overline{X}(s,t,x,p)^k \right) \right| + s^{2a} \left| \overline{\Omega }_{ij} \left( \overline{P}(s,t,x,p)^k \right) \right| \le C \varepsilon \end{aligned}$$

for all $$t_0\le s \le t$$.

The proof of Proposition 4.3 relies on two facts. The first is the fact that, for $(t,x,{\,\widehat{\!p\!}\,\,}) \in \mathrm {supp}(f)$,

4.10 $$\begin{aligned} (\overline{\Omega }_{ij} \overline{X})(t,t,x,{\,\widehat{\!p\!}\,\,}) = (\overline{\Omega }_{ij} \overline{P})(t,t,x,{\,\widehat{\!p\!}\,\,}) = 0, \end{aligned}$$

which follows from the fact that $\overline{X}(t,t,x,{\,\widehat{\!p\!}\,\,}) = \overline{P}(t,t,x,{\,\widehat{\!p\!}\,\,}) = 0$, and that $\overline{\Omega }_{ij}$ does not involve $\partial _t$ derivatives. The analogue of (4.10) is not true, due to the presence of the $\partial _t$ derivatives, when $\overline{\Omega }_{ij}$ is replaced by $\overline{B}_{i}$ or $\overline{S}$. These “final conditions” for $\overline{B}_{i}$ and $\overline{S}$, and higher order combinations of all of the $\overline{Z}$ vector fields, applied to $\overline{X}$ and $\overline{P}$ are estimated in Section 4.7.

The second fact required for the proof of Proposition 4.3 is the following estimate for $\overline{\Omega }_{ij}$ applied to the right hand side of equation (2.15).

#### Proposition 4.4

Suppose $t\ge t_0 + 1$, $\vert x \vert \le c t$, $(t,x,{\,\widehat{\!p\!}\,\,}) \in \mathrm {supp}(f)$ and the bounds (4.1) hold with $N = 0$. Then

4.11 $$\begin{aligned}&\left| \, \overline{\Omega }_{ij} \left( \hat{\Gamma }^k (s,X(s),{\,\widehat{\!P\!}\,\,}(s)) - \hat{\Gamma }^k \left( s, s\frac{x}{t},\frac{x}{t} \right) \right) \right| \lesssim \frac{\varepsilon }{s^{1+2a}} + \frac{\varepsilon }{s^{1+2a}} \left| \, \overline{\Omega }_{ij} \left( \overline{X}(s) \right) \right| \nonumber \\&\quad + \frac{\varepsilon }{s^{1+a}} \left| \,\overline{\Omega }_{ij} \left( \overline{P}(s) \right) \right| \end{aligned}$$

for all $$t_0 \le s \le t$$.

#### Proof

Recall that

$$\begin{aligned}&\hat{\Gamma }^k (s,X(s),{\,\widehat{\!P\!}\,\,}(s)) - \hat{\Gamma }^k \left( s, s\frac{x}{t},\frac{x}{t} \right) \\&\quad = \Gamma ^0_{\alpha \beta }(s,X(s)) {\,\widehat{\!P\!}\,\,}^k {\,\widehat{\!P\!}\,\,}^{\alpha } {\,\widehat{\!P\!}\,\,}^{\beta } - \Gamma ^0_{\alpha \beta }\left( s,s \frac{x}{t} \right) \frac{x^k}{t} \frac{x^{\alpha }}{t} \frac{x^{\beta }}{t} \\&\qquad + \Gamma ^k_{\alpha \beta }\left( s,s \frac{x}{t} \right) \frac{x^{\alpha }}{t} \frac{x^{\beta }}{t} - \Gamma ^k_{\alpha \beta }(s,X(s)) {\,\widehat{\!P\!}\,\,}^{\alpha } {\,\widehat{\!P\!}\,\,}^{\beta }. \end{aligned}$$

Write

$$\begin{aligned}&\Gamma ^k_{\alpha \beta }(s,X(s)) {\,\widehat{\!P\!}\,\,}^{\alpha } {\,\widehat{\!P\!}\,\,}^{\beta } - \Gamma ^k_{\alpha \beta }\left( s,s \frac{x}{t} \right) \frac{x^{\alpha }}{t} \frac{x^{\beta }}{t} \\&\quad = \left( \Gamma ^k_{\alpha \beta }(s,X(s)) - \Gamma ^k_{\alpha \beta }\left( s,s \frac{x}{t} \right) \right) \frac{x^{\alpha }}{t} \frac{x^{\beta }}{t}\\&\qquad + \Gamma ^k_{\alpha \beta }(s,X(s)) \left( {\,\widehat{\!P\!}\,\,}^{\alpha } - \frac{x^{\alpha }}{t} \right) \frac{x^{\beta }}{t} + \Gamma ^k_{\alpha \beta }(s,X(s)) \frac{x^{\alpha }}{t} \left( {\,\widehat{\!P\!}\,\,}^{\beta } - \frac{x^{\beta }}{t} \right) \\&\qquad + \Gamma ^k_{\alpha \beta }(s,X(s)) \left( {\,\widehat{\!P\!}\,\,}^{\alpha } - \frac{x^{\alpha }}{t} \right) {\,\widehat{\!P\!}\,\,}^{\beta }, \end{aligned}$$

and note that

$$\begin{aligned}&\overline{\Omega }_{ij} \left( \Gamma ^k_{\alpha \beta }(s,X(s)) - \Gamma ^k_{\alpha \beta }\left( s,s \frac{x}{t} \right) \right) = \overline{\Omega }_{ij} \left( X(s)^l - s \frac{x^l}{t} \right) (\partial _{x^l} \Gamma ^k_{\alpha \beta }) (s,X(s))\\&\quad + \overline{\Omega }_{ij} \left( s \frac{x^l}{t} \right) \left( (\partial _{x^l} \Gamma ^k_{\alpha \beta })(s,X(s)) - (\partial _{x^l} \Gamma ^k_{\alpha \beta })\left( s,s \frac{x}{t} \right) \right) . \end{aligned}$$

The bound for the first term follows from writing $X(s)^l - s \frac{x^l}{t} = \overline{X}(s)^l + X_2(s)^l - s \frac{x^l}{t}$ and, using the definition (2.13) for $X_2$ and the definition of $\mathring{\Omega }^l_{ij}$ (see (4.3)),

$$\begin{aligned}&\overline{\Omega }_{ij} \left( X_2(s)^l - s \frac{x^l}{t} \right) = x^i \delta _j^l - x^j \delta _i^l - (t-s) \left( {\,\widehat{\!p\!}\,\,}^i \delta _j^l - {\,\widehat{\!p\!}\,\,}^j \delta _i^l \right) \\&\qquad - \frac{s}{t} \left( x^i \delta _j^l - x^j \delta _i^l \right) - (t-s) \mathring{\Omega }^l_{ij}\\&\qquad - \Omega _{ij} \left( \int _s^t (s'-s) {\,\widehat{\!\Gamma \!}\,\,}^l \left( s', s'\frac{x}{t}, \frac{x}{t} \right) \,\mathrm{d}s' \right) \\&\quad = x^i \delta _j^l - x^j \delta _i^l - \left( (t-t_0) - (s-t_0) \right) \left( {\,\widehat{\!p\!}\,\,}^i \delta _j^l - {\,\widehat{\!p\!}\,\,}^j \delta _i^l \right) \\&\qquad - \frac{s}{t} \left( x^i \delta _j^l - x^j \delta _i^l \right) - (t-t_0) \mathring{\Omega }^l_{ij} + (s-t_0) \mathring{\Omega }^l_{ij}\\&\qquad - \Omega _{ij} \left( \int _{t_0}^t (s'-t_0 + t_0) {\,\widehat{\!\Gamma \!}\,\,}^l \left( s', s'\frac{x}{t}, \frac{x}{t} \right) \, \mathrm{d}s' \right. \\&\qquad \left. - \int _{t_0}^s s' {\,\widehat{\!\Gamma \!}\,\,}^l \left( s', s'\frac{x}{t}, \frac{x}{t} \right) \, \mathrm{d}s' - s \int _s^t {\,\widehat{\!\Gamma \!}\,\,}^l \left( s', s'\frac{x}{t}, \frac{x}{t} \right) \,\mathrm{d}s' \right) \\&\quad = X_2(t_0)^i \delta _j^l - X_2(t_0)^j \delta _i^l - s \left( \left( \frac{x^i}{t} - {\,\widehat{\!p\!}\,\,}^i \right) \delta _j^l - \left( \frac{x^j}{t} - {\,\widehat{\!p\!}\,\,}^j \right) \delta _i^l \right) \\&\qquad + t_0 \left( {\,\widehat{\!p\!}\,\,}^i \delta _j^l - {\,\widehat{\!p\!}\,\,}^j \delta _i^l \right) + (s-t_0) \mathring{\Omega }^l_{ij}\\&\qquad - \Omega _{ij} \left( t_0 \int _{t_0}^t {\,\widehat{\!\Gamma \!}\,\,}^l \left( s', s'\frac{x}{t}, \frac{x}{t} \right) \,\mathrm{d}s' - \int _{t_0}^s s' {\,\widehat{\!\Gamma \!}\,\,}^l \left( s', s'\frac{x}{t}, \frac{x}{t} \right) \,\mathrm{d}s' \right. \\&\qquad \left. - s \int _s^t {\,\widehat{\!\Gamma \!}\,\,}^l \left( s', s'\frac{x}{t}, \frac{x}{t} \right) \, \mathrm{d}s' \right) . \end{aligned}$$

Using the assumptions (4.1) on $\Gamma$, the fact that

$$\begin{aligned} \left| \frac{x^i}{t} - {\,\widehat{\!p\!}\,\,}^i \right| \le \frac{C}{t^a} \end{aligned}$$

for $(t,x,{\,\widehat{\!p\!}\,\,}) \in \mathrm {supp}(f)$ (see Proposition 2.2), and the fact that $\vert \mathring{\Omega }^l_{ij}(t,x) \vert \le Ct^{-a}$, it follows that

$$\begin{aligned} \left| \,\overline{\Omega }_{ij} \left( X_2(s)^l - s \frac{x^l}{t} \right) \right| \le C s^{1-a}. \end{aligned}$$

For the second term, note that

$$\begin{aligned}&\left| \, (\partial _{x^l} \Gamma ^k_{\alpha \beta })(s,X(s)) - (\partial _{x^l} \Gamma ^k_{\alpha \beta })\left( s,s \frac{x}{t} \right) \right| \le \sup _{\vert z \vert \le c s+K} \left| \partial ^2 \Gamma ^k_{\alpha \beta }(s,z) \right| \left| X(s) - s \frac{x}{t} \right| \\&\quad \le \frac{C \varepsilon }{s^{3+a}} \left| X(s) - s \frac{x}{t} \right| , \end{aligned}$$

and, as above,

$$\begin{aligned} \left| X(s)^k - s \frac{x^k}{t} \right|\le & {} \left| \overline{X}(s)^k \right| {+} \left| X_2(s)^k {-} s \frac{x^k}{t} \right| {\le } \left| \overline{X}(s)^k \right| + \left| X_2(t_0)^k \right| + s \left| \frac{x^k}{t} - {\,\widehat{\!p\!}\,\,}^k \right| \\&+ t_0 \left| \int _{t_0}^t {\,\widehat{\!\Gamma \!}\,\,}^k \left( s', s'\frac{x}{t}, \frac{x}{t} \right) \,\mathrm{d}s' \right| + \left| \int _{t_0}^s s' {\,\widehat{\!\Gamma \!}\,\,}^k \left( s', s'\frac{x}{t}, \frac{x}{t} \right) \, \mathrm{d}s' \right| \\&+ s \left| \int _{s}^t {\,\widehat{\!\Gamma \!}\,\,}^k \left( s', s'\frac{x}{t}, \frac{x}{t} \right) \,\mathrm{d}s' \right| \le Cs^{1-a}. \end{aligned}$$

Hence,

$$\begin{aligned} \left| \, \overline{\Omega }_{ij} \left( \Gamma ^k_{\alpha \beta }(s,X(s)) - \Gamma ^k_{\alpha \beta }\left( s,s \frac{x}{t} \right) \right) \right| \lesssim \frac{\varepsilon }{s^{1+2a}} + \frac{\varepsilon }{s^{2+a}} \left| \,\overline{\Omega }_{ij} (\overline{X}(s)) \right| . \end{aligned}$$

Similarly, using the fact that

$$\begin{aligned} \overline{P}(s)^k = {\,\widehat{\!P\!}\,\,}^k(s) - {\,\widehat{\!p\!}\,\,}^k + \int _s^t {\,\widehat{\!\Gamma \!}\,\,}^k \left( s', s'\frac{x}{t}, \frac{x}{t} \right) \,\mathrm{d}s', \end{aligned}$$

it follows that

$$\begin{aligned} \left| {\,\widehat{\!P\!}\,\,}(s)^k - \frac{x^k}{t} \right|\lesssim & {} \left| \overline{P}(s)^k \right| + \left| \frac{x^k}{t} - {\,\widehat{\!p\!}\,\,}^k \right| \\&+ \int _s^t \left| \,{\,\widehat{\!\Gamma \!}\,\,}^k \left( s', s'\frac{x}{t}, \frac{x}{t} \right) \right| \,\mathrm{d}s' \lesssim s^{-a}, \quad \left| {\,\widehat{\!P\!}\,\,}(s)^k \right| \lesssim 1, \end{aligned}$$

and similarly,

$$\begin{aligned} \left| \,\overline{\Omega }_{ij} \left( {\,\widehat{\!P\!}\,\,}(s)^k - \frac{x^k}{t} \right) \right|\lesssim & {} \left| \, \overline{\Omega }_{ij} \left( \overline{P}(s)^k \right) \right| + s^{-a}, \\ \left| \, \overline{\Omega }_{ij} \left( {\,\widehat{\!P\!}\,\,}(s)^k \right) \right|\lesssim & {} \left| \, \overline{\Omega }_{ij} \left( \overline{P}(s)^k \right) \right| + 1. \end{aligned}$$

Hence,

$$\begin{aligned}&\left| \,\overline{\Omega }_{ij} \left( \Gamma ^k_{\alpha \beta }(s,X(s)) {\,\widehat{\!P\!}\,\,}^{\alpha } {\,\widehat{\!P\!}\,\,}^{\beta } - \Gamma ^k_{\alpha \beta }\left( s,s \frac{x}{t} \right) \frac{x^{\alpha }}{t} \frac{x^{\beta }}{t} \right) \right| \\&\quad \lesssim \frac{\varepsilon }{s^{1+2a}} + \frac{\varepsilon }{s^{1+2a}} \left| \,\overline{\Omega }_{ij} \left( \overline{X}(s) \right) \right| + \frac{\varepsilon }{s^{1+a}} \left| \, \overline{\Omega }_{ij} \left( \overline{P}(s) \right) \right| . \end{aligned}$$

In a similar manner it follows that

$$\begin{aligned}&\left| \,\overline{\Omega }_{ij} \left( \Gamma ^0_{\alpha \beta }(s,X(s)) {\,\widehat{\!P\!}\,\,}^k {\,\widehat{\!P\!}\,\,}^{\alpha } {\,\widehat{\!P\!}\,\,}^{\beta } - \Gamma ^0_{\alpha \beta }\left( s,s \frac{x}{t} \right) \frac{x^k}{t} \frac{x^{\alpha }}{t} \frac{x^{\beta }}{t} \right) \right| \\&\quad \lesssim \frac{\varepsilon }{s^{1+2a}} + \frac{\varepsilon }{s^{1+2a}} \left| \, \overline{\Omega }_{ij} \left( \overline{X}(s) \right) \right| + \frac{\varepsilon }{s^{1+a}} \left| \, \overline{\Omega }_{ij} \left( \overline{P}(s) \right) \right| , \end{aligned}$$

from which (4.11) then follows. $\quad \square$

A higher order analogue of Proposition 4.4, which includes also the boosts and scaling and is used in the proof of Proposition 4.24 and Corollary 4.27, is obtained in Section 4.5.

#### Proof of Proposition 4.3

The proof proceeds by applying $\overline{\Omega }_{ij}$ to the system (2.14)–(2.15). Applying $\overline{\Omega }_{ij}$ to the equation (2.14) and integrating backwards from $s=t$, using (4.10), that

$$\begin{aligned} \left| \, \overline{\Omega }_{ij} ( \overline{P}(s)^k) \right| \lesssim \frac{\varepsilon }{s^{2a}} + \int _s^t \frac{\varepsilon }{\tilde{s}^{1+2a}} \left| \, \overline{\Omega }_{ij} (\overline{X}(\tilde{s})) \right| + \frac{\varepsilon }{\tilde{s}^{1+a}} \left| \, \overline{\Omega }_{ij} (\overline{P}(\tilde{s})) \right| \,\mathrm{d}\tilde{s}, \end{aligned}$$

and, summing over $k=1,2,3$, the Grönwall inequality (see Lemma 4.25) gives

4.12 $$\begin{aligned} \left| \,\overline{\Omega }_{ij} ( \overline{P}(s)^k) \right| \lesssim \frac{\varepsilon }{s^{2a}} + \int _s^t \frac{\varepsilon }{\tilde{s}^{1+2a}} \left| \,\overline{\Omega }_{ij} (\overline{X}(\tilde{s})) \right| \,\mathrm{d}\tilde{s}. \end{aligned}$$

Inserting this bound into the equation (2.14) for $\overline{X}$, after applying $\overline{\Omega }_{ij}$, integrating backwards from $s=t$ and using (4.10) gives

$$\begin{aligned} \left| \,\overline{\Omega }_{ij} ( \overline{X}(s)^k) \right| \lesssim \varepsilon s^{1-2a} + \int _s^t \int _{s'}^t \frac{\varepsilon }{\tilde{s}^{1+2a}} \left| \,\overline{\Omega }_{ij} (\overline{X}(\tilde{s})) \right| \,\mathrm{d}\tilde{s} \mathrm{d}s', \end{aligned}$$

since $2a>1$. For any function $\lambda (\tilde{s})$,

$$\begin{aligned} \int _s^t \int _{s'}^t \frac{1}{\tilde{s}^{1+2a}} \lambda (\tilde{s}) \,\mathrm{d}\tilde{s} \mathrm{d}s' = \int _s^t \int _{s}^t \chi _{\{ s'\le \tilde{s} \}} \mathrm{d}s' \frac{1}{\tilde{s}^{1+2a}} \lambda (\tilde{s}) \,\mathrm{d}\tilde{s} = \int _s^t \frac{(\tilde{s} - s)}{\tilde{s}^{1+2a}} \lambda (\tilde{s}) \,\mathrm{d}\tilde{s}, \end{aligned}$$

where $\chi _A$ denotes the indicator function of the set *A*, and so

$$\begin{aligned} \left| \,\overline{\Omega }_{ij} ( \overline{X}(s)^k) \right| \lesssim \varepsilon s^{1-2a} + \int _s^t \frac{\varepsilon }{\tilde{s}^{2a}} \left| \,\overline{\Omega }_{ij} (\overline{X}(\tilde{s})) \right| \,\mathrm{d}\tilde{s}, \end{aligned}$$

and another application of the Grönwall inequality gives

$$\begin{aligned} \left| \,\overline{\Omega }_{ij} ( \overline{X}(s)^k) \right| \lesssim \varepsilon s^{1-2a}. \end{aligned}$$

The proof follows after inserting this bound back into (4.12). $\quad \square$

### Repeated Vector Fields Applied to the Initial Conditions for Approximations to Geodesics

Recall the discussion at the beginning of Section 4. In order to motivate the results of this section and the next section note that, after applying $\overline{Z}^I$ to the equation (2.15), the term

$$\begin{aligned}&\overline{Z}^I(X^k) (\partial _k {\,\widehat{\!\Gamma \!}\,\,}^i)(s,X,{\,\widehat{\!P\!}\,\,}) - \overline{Z}^I\left( \frac{sx^k}{t} \right) (\partial _k {\,\widehat{\!\Gamma \!}\,\,}^i) \left( s, \frac{sx}{t}, \frac{x}{t} \right) \\&\quad = \overline{Z}^I\left( X^k - \frac{sx^k}{t} \right) (\partial _k {\,\widehat{\!\Gamma \!}\,\,}^i)(s,X,{\,\widehat{\!P\!}\,\,}) \\&\qquad + \overline{Z}^I\left( \frac{sx^k}{t} \right) \left[ (\partial _k {\,\widehat{\!\Gamma \!}\,\,}^i)(s,X,{\,\widehat{\!P\!}\,\,}) - (\partial _k {\,\widehat{\!\Gamma \!}\,\,}^i) \left( s, \frac{sx}{t}, \frac{x}{t} \right) \right] , \end{aligned}$$

(amongst others) appears. Consider the first of these summands. Rewriting

$$\begin{aligned} \overline{Z}^I\left( X^k(s) - \frac{sx^k}{t} \right) = \overline{Z}^I\left( \overline{X}^k(s) \right) + \overline{Z}^I\left( X^k_2(s) - \frac{sx^k}{t} \right) , \end{aligned}$$

the first term $\overline{Z}^I(\overline{X}^k)$ is controlled, in the proofs of Propositions 4.23 and 4.26, by the Grönwall inequality. In Section 4.4, the term $\overline{Z}^I\left( X^k_2(s) - \frac{sx^k}{t} \right)$ is controlled by first rewriting

$$\begin{aligned} \overline{Z}^I \left( s\frac{x^i}{t} - X_2^i(s,t,x,{\,\widehat{\!p\!}\,\,}) \right)= & {} \overline{Z}^I \left( s\frac{x^i}{t} - X_2^i(s,t,x,{\,\widehat{\!p\!}\,\,}) \right. \\&\left. + X_2^i(t_0,t,x,{\,\widehat{\!p\!}\,\,}) \right) - \overline{Z}^I (X_2^i(t_0,t,x,{\,\widehat{\!p\!}\,\,})). \end{aligned}$$

The second term is computed schematically in Proposition 4.5 below, and is controlled in Section 4.4. For the first term, the fact that

4.13 $$\begin{aligned}&s\frac{x^i}{t} - X_2^i(s,t,x,{\,\widehat{\!p\!}\,\,}) + X_2^i(t_0,t,x,{\,\widehat{\!p\!}\,\,}) \nonumber \\&\quad = s \left( \frac{x^i}{t} - {\,\widehat{\!p\!}\,\,}^i \right) + t_0 {\,\widehat{\!p\!}\,\,}^i + \int _s^t (s' - s) {\,\widehat{\!\Gamma \!}\,\,}^k \left( s', s'\frac{x}{t}, \frac{x}{t} \right) \,\mathrm{d}s' \nonumber \\&\qquad - \int _{t_0}^t (s' - t_0) {\,\widehat{\!\Gamma \!}\,\,}^k \left( s', s'\frac{x}{t}, \frac{x}{t} \right) \,\mathrm{d}s' \nonumber \\&\quad = s \left( \frac{x^i}{t} - {\,\widehat{\!p\!}\,\,}^i \right) + t_0 {\,\widehat{\!p\!}\,\,}^i - \int _{t_0}^s (s' - t_0) {\,\widehat{\!\Gamma \!}\,\,}^k \left( s', s'\frac{x}{t}, \frac{x}{t} \right) \,\mathrm{d}s' \nonumber \\&\qquad - (s - t_0) \int _{s}^t {\,\widehat{\!\Gamma \!}\,\,}^k \left( s', s'\frac{x}{t}, \frac{x}{t} \right) \,\mathrm{d}s' \end{aligned}$$

is used, along with the schematic expressions of Propositions 4.6 and 4.7. The expression

4.14 $$\begin{aligned} \overline{Z}^I \left( \frac{x^i}{t} - {\,\widehat{\!P\!}\,\,}^i(s,t,x,p) \right) = - \overline{Z}^I \left( \overline{P}^i(s,t,x,p) \right) + \overline{Z}^I \left( \frac{x^i}{t} -\widehat{P}_2^i(s,t,x,p) \right) \end{aligned}$$

similarly appears after applying $\overline{Z}^I$ to equation (2.15), where

$$\begin{aligned} \widehat{P}_2^i(s,t,x,p)= {\,\widehat{\!p\!}\,\,}^i+\int _s^t {\,\widehat{\!\Gamma \!}\,\,}^i \left( s', s'\frac{x}{t}, \frac{x}{t} \right) \,\mathrm{d}s', \end{aligned}$$

and is therefore also estimated in Section 4.4.

The following generalises Proposition 4.1 to higher orders. Recall it is assumed that $t\ge t_0+1$. Section 4.10 is concerned with the case $t_0 \le t \le t_0 + 1$.

#### Proposition 4.5

For any multi index *I*, there exist smooth functions $\Lambda ^i_{I}$, $\Lambda ^i_{I,j}$ such that

$$\begin{aligned}&\overline{Z}^I \left( X_2(t_0)^i \right) \\&\quad = X_2(t_0)^j \Bigg ( \Lambda ^i_{I,j} \left( {\,\widehat{\!p\!}\,\,}\right) + \sum _{k=1}^{\vert I\vert -1} \sum _{\vert J_1\vert + \cdots + \vert J_k \vert \le \vert I \vert -k} \tilde{\Lambda }_{I,j,i_1,\ldots ,i_k}^{i,J_1,\ldots ,J_k} \left( {\,\widehat{\!p\!}\,\,},t,x \right) Z^{J_1} \left( \Theta ^{i_1} \right) \ldots Z^{J_k} \left( \Theta ^{i_k} \right) \Bigg )\\&\qquad + \Lambda ^i_{I} \left( {\,\widehat{\!p\!}\,\,}\right) + \sum _{k=1}^{\vert I\vert -1} \sum _{\vert J_1\vert + \cdots + \vert J_k \vert \le \vert I \vert -k} \tilde{\Lambda }_{I,i_1,\ldots ,i_k}^{i,J_1,\ldots ,J_k} \left( {\,\widehat{\!p\!}\,\,},t,x \right) Z^{J_1} \left( \Theta ^{i_1} \right) \ldots Z^{J_k} \left( \Theta ^{i_k} \right) \end{aligned}$$

for $i=1,2,3$, where $X_2(t_0)^i = X_2(t_0,t,x,p)^i$ and $\tilde{\Lambda }_{I,j,i_1,\ldots ,i_k}^{i,J_1,\ldots ,J_k}$ and $\tilde{\Lambda }_{I,i_1,\ldots ,i_k}^{i,J_1,\ldots ,J_k}$ satisfy

$$\begin{aligned} \tilde{\Lambda }_{I,j,i_1,\ldots ,i_k}^{i,J_1,\ldots ,J_k} \left( {\,\widehat{\!p\!}\,\,},t,x \right)= & {} \Lambda _{I,j,i_1,\ldots ,i_k}^{i,J_1,\ldots ,J_k} \left( {\,\widehat{\!p\!}\,\,}, \frac{x}{t-t_0}, \frac{t}{t-t_0} \right) , \\ \tilde{\Lambda }_{I,i_1,\ldots ,i_k}^{i,J_1,\ldots ,J_k} \left( {\,\widehat{\!p\!}\,\,},t,x \right)= & {} \Lambda _{I,i_1,\ldots ,i_k}^{i,J_1,\ldots ,J_k} \left( {\,\widehat{\!p\!}\,\,}, \frac{x}{t-t_0}, \frac{t}{t-t_0} \right) , \end{aligned}$$

for some smooth functions $\Lambda _{I,j,i_1,\ldots ,i_k}^{i,J_1,\ldots ,J_k}$ and $\Lambda _{I,i_1,\ldots ,i_k}^{i,J_1,\ldots ,J_k}$. (Here $\sum _{k=1}^0 := 0$.)

#### Proof

The result is clearly true for $\vert I \vert = 1$ by Proposition 4.1. The result for $\vert I \vert \ge 2$ then follows from a straightforward induction argument after noting that, for any multi index $J_j$,

$$\begin{aligned} \overline{Z}\left( Z^{J_j} \left( \Theta ^{i_j} \right) \right) = Z^{L} \left( \Theta ^{i_j} \right) , \end{aligned}$$

where $\vert L \vert = \vert J_j \vert + 1$, and also noting that

$$\begin{aligned} \overline{\Omega }_{ij} \left( \frac{t}{t-t_0} \right)&= 0, \overline{\Omega }_{ij} \left( \frac{x^k}{t-t_0} \right) = \frac{x^i}{t-t_0} \delta _j^k - \frac{x^j}{t-t_0} \delta _i^k,\\ \overline{B}_{i} \left( \frac{t}{t-t_0} \right)&= \frac{x^i}{t-t_0} - \frac{x^i}{t-t_0} \frac{t}{t-t_0}, \overline{B}_{i} \left( \frac{x^k}{t-t_0} \right) = \frac{t}{t-t_0} \delta _i^k - \frac{x^i}{t-t_0} \frac{x^k}{t-t_0},\\ \overline{S} \left( \frac{t}{t-t_0} \right)&= \frac{t}{t-t_0} - \left( \frac{t}{t-t_0} \right) ^2, \overline{S} \left( \frac{x^k}{t-t_0} \right) = \frac{x^k}{t-t_0} - \frac{x^k}{t-t_0} \frac{t}{t-t_0}, \end{aligned}$$

and

4.15 $$\begin{aligned} \overline{\Omega }_{ij} \left( {\,\widehat{\!p\!}\,\,}^k \right)&= \left( {\,\widehat{\!p\!}\,\,}^i + \Theta ^i \right) \delta _j^k - \left( {\,\widehat{\!p\!}\,\,}^j + \Theta ^j \right) \delta _i^k - \Omega _{ij} \left( \Theta ^k \right) , \end{aligned}$$

4.16 $$\begin{aligned} \overline{B}_{i} \left( {\,\widehat{\!p\!}\,\,}^k \right)&= \left( \delta _i^k - {\,\widehat{\!p\!}\,\,}^i {\,\widehat{\!p\!}\,\,}^k \right) - \Theta ^i {\,\widehat{\!p\!}\,\,}^k - \frac{x^i}{t-t_0} \Theta ^k - B_{i} \left( \Theta ^k \right) , \end{aligned}$$

4.17 $$\begin{aligned} \overline{S} \left( {\,\widehat{\!p\!}\,\,}^k \right)&= \Theta ^k - \frac{t}{t-t_0} \Theta ^k - S \left( \Theta ^k \right) , \end{aligned}$$

where the equalities (4.4), (4.5), (4.6) have been used. Hence, for any smooth function $\Lambda \left( {\,\widehat{\!p\!}\,\,}, \frac{x}{t-t_0}, \frac{t}{t-t_0} \right)$ and any $\overline{Z}= \overline{\Omega }_{ij}$, $\overline{B}_i$, *S*, there exist smooth functions $\tilde{\Lambda }$, $\tilde{\Lambda }_J$ such that

$$\begin{aligned}&\overline{Z}\left( \Lambda \left( {\,\widehat{\!p\!}\,\,}, \frac{x}{t-t_0}, \frac{t}{t-t_0} \right) \right) = \tilde{\Lambda } \left( {\,\widehat{\!p\!}\,\,}, \frac{x}{t-t_0}, \frac{t}{t-t_0} \right) \\&\quad + \sum _{\vert J \vert \le 1} \tilde{\Lambda }_J \left( {\,\widehat{\!p\!}\,\,}, \frac{x}{t-t_0}, \frac{t}{t-t_0} \right) Z^J(\Theta ), \end{aligned}$$

and the proof follows. $\quad \square$

Note that the arguments of the smooth functions $\Lambda ^i_{I}$, $\Lambda ^i_{I,j}$, $\Lambda _{I,j,i_1,\ldots ,i_k}^{i,J_1,\ldots ,J_k}$ and $\Lambda _{I,i_1,\ldots ,i_k}^{i,J_1,\ldots ,J_k}$ appearing in Proposition 4.5 are bounded in $\mathrm {supp}(f)$. Note also that the functions themselves also depend on $t_0$ but, since $t_0$ is considered fixed, this dependence is not made explicit. Things are similar for the functions appearing in Propositions 4.6 and 4.7 below.

#### Proposition 4.6

For any multi index *I*, there exist functions $\Lambda ^i_{I,j}$ such that

$$\begin{aligned}&\overline{Z}^I \left( \frac{x^i}{t} - {\,\widehat{\!p\!}\,\,}^i \right) = \left( \frac{x^j}{t} - {\,\widehat{\!p\!}\,\,}^j \right) \Lambda ^i_{I,j} \left( \frac{x}{t}, {\,\widehat{\!p\!}\,\,}\right) \\&\quad + \sum _{k=1}^{\vert I\vert } \sum _{\vert J_1\vert + \cdots + \vert J_k \vert \le \vert I \vert -k+1} \!\!\!\!\!\! \tilde{\Lambda }_{I,i_1,\ldots ,i_k}^{i,J_1,\ldots ,J_k} \left( {\,\widehat{\!p\!}\,\,},t,x \right) Z^{J_1} \left( \Theta ^{i_1} \right) \ldots Z^{J_k} \left( \Theta ^{i_k} \right) \end{aligned}$$

for $$i=1,2,3$$, where

$$\begin{aligned} \tilde{\Lambda }_{I,i_1,\ldots ,i_k}^{i,J_1,\ldots ,J_k} \left( {\,\widehat{\!p\!}\,\,},t,x \right) = \Lambda _{I,i_1,\ldots ,i_k}^{i,J_1,\ldots ,J_k} \left( {\,\widehat{\!p\!}\,\,}, \frac{x}{t}, \frac{x}{t-t_0}, \frac{t}{t-t_0} \right) \end{aligned}$$

for some smooth functions $\Lambda _{I,i_1,\ldots ,i_k}^{i,J_1,\ldots ,J_k}$.

#### Proof

Note that,

$$\begin{aligned} \overline{\Omega }_{ij} \left( \frac{x^k}{t} - {\,\widehat{\!p\!}\,\,}^k \right) =&\, \left( \frac{x^i}{t} - {\,\widehat{\!p\!}\,\,}^i \right) \delta _j^k - \left( \frac{x^j}{t} - {\,\widehat{\!p\!}\,\,}^j \right) \delta _i^k - \mathring{\Omega }_{ij}^k\\ \overline{B}_{i} \left( \frac{x^k}{t} - {\,\widehat{\!p\!}\,\,}^k \right) =&\, - \left( \frac{x^i}{t} - {\,\widehat{\!p\!}\,\,}^i \right) {\,\widehat{\!p\!}\,\,}^k - \frac{x^i}{t} \left( \frac{x^k}{t} - {\,\widehat{\!p\!}\,\,}^k \right) - \mathring{B}_i^k\\ \overline{S} \left( \frac{x^k}{t} - {\,\widehat{\!p\!}\,\,}^k \right) =&\,- \mathring{S}^k, \end{aligned}$$

and so, inserting the equalities (4.4), (4.5), (4.6), the result is clearly true for $\vert I \vert = 1$. The result for $\vert I \vert \ge 2$ follows from a straightforward induction, as in the proof of Proposition 4.5, now also using the fact that

4.18 $$\begin{aligned} \overline{\Omega }_{ij} \left( \frac{x^k}{t} \right) = \frac{x^i}{t} \delta _j^k - \frac{x^j}{t} \delta _i^k, \quad \overline{B}_{i} \left( \frac{x^k}{t} \right) = \delta _i^k - \frac{x^i}{t} \frac{x^k}{t}, \quad \overline{S} \left( \frac{x^k}{t} \right) = 0. \end{aligned}$$

$$\square $$

#### Proposition 4.7

For any multi index *I*, there exist smooth functions $\Lambda ^i_{I}$ such that

$$\begin{aligned} \overline{Z}^I \left( {\,\widehat{\!p\!}\,\,}^i \right) = \Lambda ^i_{I} \left( {\,\widehat{\!p\!}\,\,}\right)&+ \sum _{k=1}^{\vert I\vert } \sum _{\vert J_1\vert + \cdots + \vert J_k \vert \le \vert I \vert -k+1} \tilde{\Lambda }_{I,i_1,\ldots ,i_k}^{i,J_1,\ldots ,J_k} \left( {\,\widehat{\!p\!}\,\,},t,x \right) Z^{J_1} \left( \Theta ^{i_1} \right) \ldots Z^{J_k} \left( \Theta ^{i_k} \right) \end{aligned}$$

for $$i=1,2,3$$, where

$$\begin{aligned} \tilde{\Lambda }_{I,i_1,\ldots ,i_k}^{i,J_1,\ldots ,J_k} \left( {\,\widehat{\!p\!}\,\,},t,x \right) = \Lambda _{I,i_1,\ldots ,i_k}^{i,J_1,\ldots ,J_k} \left( {\,\widehat{\!p\!}\,\,}, \frac{x}{t-t_0}, \frac{t}{t-t_0} \right) \end{aligned}$$

for some smooth functions $\Lambda _{I,i_1,\ldots ,i_k}^{i,J_1,\ldots ,J_k}$.

#### Proof

The result for $\vert I \vert = 1$ clearly follows from the equalities (4.15), (4.16), (4.17). The proof for $\vert I \vert \ge 2$ follows from a straightforward induction argument, as in the proof of Proposition 4.5. $\quad \square$

### Preliminary Estimates for Repeated Vector Fields Applied to Approximations to Geodesics

#### Proposition 4.8

Suppose $t\ge t_0 + 1$, $\vert x \vert \le c t$ and the bounds (4.1) hold. Then, for $\vert I \vert \le N$,

$$\begin{aligned}&\left| Z^I \Theta (t,x) \right| \lesssim \sum _{\vert J\vert \le \vert I \vert -1} t \left| (Z^J \Gamma )(t,x) \right| \\&\quad + \sum _{\vert J\vert \le \vert I \vert } \frac{1}{t-t_0} \int _{t_0}^t (s'- t_0) \left| (Z^J \Gamma ) \left( s', s'\frac{x}{t} \right) \right| \,\mathrm{d}s', \end{aligned}$$

where $Z^I$ is a product of $\vert I \vert$ of the vector fields $\Omega _{ij}, B_i, S$. Moreover, if $\vert I \vert \le \left\lfloor \frac{N}{2} \right\rfloor + 2$, then

$$\begin{aligned} \left| Z^I \Theta (t,x) \right| \le \frac{C}{t^a}. \end{aligned}$$

#### Proof

Recall the definition (4.2) of $\Theta$. Note that

$$\begin{aligned} Z \left( \Theta ^i(t,x) \right)= & {} - \frac{Z(t)}{t-t_0} \Theta ^i(t,x) + Z(t) {\,\widehat{\!\Gamma \!}\,\,}^i \left( t,x,\frac{x}{t} \right) \\&+ \frac{1}{t-t_0} \int _{t_0}^t (s'-t_0) Z \left( {\,\widehat{\!\Gamma \!}\,\,}^i \left( s', s'\frac{x}{t}, \frac{x}{t} \right) \right) \,\mathrm{d}s', \end{aligned}$$

and that

$$\begin{aligned} \Omega _{ij} \left( \Gamma ^{\mu }_{\alpha \beta } \left( s, s\frac{x}{t} \right) \right)= & {} s \frac{x^i}{t} \left( \partial _{x^j} \Gamma ^{\mu }_{\alpha \beta } \right) \left( s, s\frac{x}{t} \right) - s \frac{x^j}{t} \left( \partial _{x^i} \Gamma ^{\mu }_{\alpha \beta } \right) \left( s, s\frac{x}{t} \right) \\= & {} \left( \Omega _{ij} \Gamma ^{\mu }_{\alpha \beta } \right) \left( s, s\frac{x}{t} \right) ,\\ B_i \left( \Gamma ^{\mu }_{\alpha \beta } \left( s, s\frac{x}{t} \right) \right)= & {} s \left( \partial _{x^i} \Gamma ^{\mu }_{\alpha \beta } \right) \left( s, s\frac{x}{t} \right) - s \frac{x^i}{t} \frac{x^k}{t} \left( \partial _{x^k} \Gamma ^{\mu }_{\alpha \beta } \right) \left( s, s\frac{x}{t} \right) \\= & {} \left( B_{i} \Gamma ^{\mu }_{\alpha \beta } \right) \left( s, s\frac{x}{t} \right) - \frac{x^i}{t} \left( S \Gamma ^{\mu }_{\alpha \beta } \right) \left( s, s\frac{x}{t} \right) , \end{aligned}$$

and

$$\begin{aligned} S \left( \Gamma ^{\mu }_{\alpha \beta } \left( s, s\frac{x}{t} \right) \right) = 0. \end{aligned}$$

Using the equalities (4.18), the result for $\vert I \vert = 1$ clearly follows from the $L^{\infty }$ bounds (4.1) for $\Gamma ^{\mu }_{\alpha \beta }$ (recall that, when restricted to spacetime functions, the $\overline{Z}$ vector fields are equal to the *Z* vector fields). The proof for $\vert I \vert \ge 2$ is a straightforward induction. $\quad \square$

#### Proposition 4.9

Supposing that $t\ge t_0 + 1$, $\vert x \vert \le c t$, $(t,x,{\,\widehat{\!p\!}\,\,}) \in \mathrm {supp}(f)$ and the bounds (4.1) hold, then, for $\vert I \vert \le N$,

4.19 $$\begin{aligned} \left| \overline{Z}^I \left( X_2(t_0)^i \right) \right| \lesssim&1 +\sum _{\vert J\vert \le \vert I \vert -1} t \left| (Z^J \Gamma )(t,x) \right| \nonumber \\&+\sum _{\vert J\vert \le \vert I \vert } \frac{1}{t-t_0} \int _{t_0}^t (s'- t_0) \left| (Z^J \Gamma ) \left( s', s'\frac{x}{t} \right) \right| \,\mathrm{d}s' \end{aligned}$$

4.20 $$\begin{aligned} \left| \overline{Z}^I \left( \frac{x^i}{t} - {\,\widehat{\!p\!}\,\,}^i \right) \right| \lesssim&\frac{1}{t^a} +\sum _{\vert J\vert \le \vert I \vert -1} t \left| (Z^J \Gamma )(t,x) \right| \nonumber \\&+\sum _{\vert J\vert \le \vert I \vert } \frac{1}{t-t_0} \int _{t_0}^t (s'- t_0) \left| (Z^J \Gamma ) \left( s', s'\frac{x}{t} \right) \right| \,\mathrm{d}s' \end{aligned}$$

4.21 $$\begin{aligned} \left| \overline{Z}^I \left( {\,\widehat{\!p\!}\,\,}^i \right) \right| \lesssim&1 +\sum _{\vert J\vert \le \vert I \vert -1} t \left| (Z^J \Gamma )(t,x) \right| \nonumber \\&+\sum _{\vert J\vert \le \vert I \vert } \frac{1}{t-t_0} \int _{t_0}^t (s'- t_0) \left| (Z^J \Gamma ) \left( s', s'\frac{x}{t} \right) \right| \,\mathrm{d}s' \end{aligned}$$

and

4.22 $$\begin{aligned}&\left| \overline{Z}^I \left( \int _{t_0}^s (s' - t_0) {\,\widehat{\!\Gamma \!}\,\,}^i \left( s', s'\frac{x}{t}, \frac{x}{t} \right) \, \mathrm{d}s' \right) \right| \nonumber \\ \lesssim&\ \sum _{\vert J\vert \le \vert I \vert } \int _{t_0}^s (s'- t_0) \left| (Z^J \Gamma ) \left( s', s'\frac{x}{t} \right) \right| \, \mathrm{d}s' \end{aligned}$$

4.23 $$\begin{aligned} \left| \overline{Z}^I \left( \int _{s}^t {\,\widehat{\!\Gamma \!}\,\,}^i \left( s', s'\frac{x}{t}, \frac{x}{t} \right) \, \mathrm{d}s' \right) \right| \lesssim&\ \sum _{\vert J\vert \le \vert I \vert -1} t \left| (Z^J\Gamma )(t,x)\right| \nonumber \\&+\sum _{\vert J\vert \le \vert I \vert } \int _{s}^t \left| (Z^J \Gamma ) \left( s', s'\frac{x}{t} \right) \right| \, \mathrm{d}s' \end{aligned}$$

for $i=1,2,3$ and for all $t_0 \le s \le t$. In particular, for $\vert I \vert \le \left\lfloor \frac{N}{2} \right\rfloor + 2$,

$$\begin{aligned} \left| \overline{Z}^I \left( X_2(t_0)^i \right) \right| + \left| \overline{Z}^I \left( {\,\widehat{\!p\!}\,\,}^i \right) \right| \le C, \quad \left| \overline{Z}^I \left( \frac{x^i}{t} - {\,\widehat{\!p\!}\,\,}^i \right) \right| \le \frac{C}{t^a} \end{aligned}$$

for $$i=1,2,3$$.

#### Proof

Consider first the bound (4.19). Proposition 4.5 implies that

$$\begin{aligned} \left| \overline{Z}^I \left( X_2(t_0)^i \right) \right| \lesssim 1 + \sum _{k=1}^{\vert I\vert -1} \sum _{\vert J_1\vert + \cdots + \vert J_k \vert \le \vert I \vert -k} \left| Z^{J_1} \left( \Theta ^{i_1} \right) \right| \ldots \left| Z^{J_k} \left( \Theta ^{i_k} \right) \right| , \end{aligned}$$

where the fact that $\vert X_2(t_0)^i \vert \lesssim 1$ has been used (see Corollary 2.4). If $\vert J_l \vert \le \left\lfloor \frac{N}{2} \right\rfloor +2$ then Proposition 4.8 and the bounds (4.1) imply that $\left| Z^{J_l} \left( \Theta ^{i_l} \right) \right| \lesssim 1$. The estimate (4.19) then follows from Proposition 4.8 after noting that, for all $\vert J_1 \vert + \cdots + \vert J_k \vert \le \vert I \vert$, there is at most one $1 \le j \le k$ such that $\vert J_j \vert > \left\lfloor \frac{N}{2} \right\rfloor +2$.

The estimate (4.20) follows similarly (using now Proposition 4.6 in place of Propositions 4.5 and 2.2 in place of Corollary 2.4), as does the estimate (4.21) (using Proposition 4.7 in place of Proposition 4.5). The bounds (4.22) and (4.23) can be shown as in the proof of Proposition 4.8. $\quad \square$

#### Corollary 4.10

Supposing that $t\ge t_0 + 1$, $\vert x \vert \le c t$, $(t,x,{\,\widehat{\!p\!}\,\,}) \in \mathrm {supp}(f)$ and the bounds (4.1) hold, then

4.24 $$\begin{aligned} \left| \overline{Z}^I \left( \frac{X_2^i(s,t,x,{\,\widehat{\!p\!}\,\,})}{s} \right) \right| \lesssim&1 + \sum _{\vert J \vert \le \vert I \vert -1} \!\!\!\!\! t \left| (Z^J \Gamma )(t,x) \right| \nonumber \\&+ \sum _{\vert J \vert \le \vert I \vert } \int _{t_0}^t \left| (Z^J \Gamma ) \left( s', s' \frac{x}{t} \right) \right| \frac{s'}{s'+s} \, \mathrm{d}s', \end{aligned}$$

and

4.25 $$\begin{aligned} \left| \overline{Z}^I \left( \frac{X_2^i(s,t,x,{\,\widehat{\!p\!}\,\,})}{s} - \frac{x^i}{t} \right) \right| \lesssim&\frac{1}{s^a} + \sum _{\vert J \vert \le \vert I \vert -1} \!\!\!\!\! t \left| (Z^J \Gamma )(t,x) \right| \nonumber \\&+ \sum _{\vert J \vert \le \vert I \vert } \int _{t_0}^t \left| (Z^J \Gamma ) \left( s', s' \frac{x}{t} \right) \right| \frac{s'}{s'+s}\, \mathrm{d}s' \end{aligned}$$

for all $t_0\le s \le t$, $i=1,2,3$. In particular, for $\vert I \vert \le \left\lfloor \frac{N}{2} \right\rfloor + 2$,

4.26 $$\begin{aligned} \left| \overline{Z}^I \left( \frac{X_2^i(s,t,x,{\,\widehat{\!p\!}\,\,})}{s} - \frac{x^i}{t} \right) \right| \lesssim \frac{1}{s^a}. \end{aligned}$$

Moreover,

4.27 $$\begin{aligned} \left| \overline{Z}^I \Big ( \frac{x^i}{t} - \widehat{P}_2^i(s,t,x,{\,\widehat{\!p\!}\,\,}) \Big ) \right|\lesssim & {} \frac{1}{t^a} + \sum _{\vert J\vert \le \vert I \vert -1}\!\!\!\!\! t \left| (Z^J \Gamma )(t,x) \right| \nonumber \\&+ \sum _{\vert J\vert \le \vert I \vert } \int _{t_0}^t \left| (Z^J \Gamma ) \left( s', s'\frac{x}{t} \right) \right| \frac{s'}{s'+s} \,\mathrm{d}s'. \end{aligned}$$

In particular, for $\vert I \vert \le \left\lfloor \frac{N}{2} \right\rfloor + 2$,

4.28 $$\begin{aligned} \left| \overline{Z}^I \left( \widehat{P}_2^i(s,t,x,{\,\widehat{\!p\!}\,\,}) - \frac{x^i}{t} \right) \right| \lesssim \frac{1}{s^a}. \end{aligned}$$

#### Proof

After writing

$$\begin{aligned} X_2^i(s,t,x,{\,\widehat{\!p\!}\,\,}) =&\, \ X_2^i(t_0,t,x,{\,\widehat{\!p\!}\,\,}) + \left( X_2^i(s,t,x,{\,\widehat{\!p\!}\,\,}) - X_2^i(t_0,t,x,{\,\widehat{\!p\!}\,\,}) \right) \\ =&\, \ X_2^i(t_0,t,x,{\,\widehat{\!p\!}\,\,}) + (s-t_0) {\,\widehat{\!p\!}\,\,}^i + (s-t_0) \int _s^t {\,\widehat{\!\Gamma \!}\,\,}^i \left( s', s' \frac{x}{t}, \frac{x}{t} \right) \, \mathrm{d}s' \\&+ \int _{t_0}^s (s' - t_0) {\,\widehat{\!\Gamma \!}\,\,}^i \left( s', s' \frac{x}{t}, \frac{x}{t} \right) \,\mathrm{d}s', \end{aligned}$$

Proposition 4.9 implies that

$$\begin{aligned} \left| \overline{Z}^I \left( X_2^i(s,t,x,{\,\widehat{\!p\!}\,\,}) \right) \right| \le&C s \Bigg [ 1 + \sum _{\vert J \vert \le \vert I \vert -1} t \left| (Z^J \Gamma )(t,x) \right| \\&+ \sum _{\vert J \vert \le \vert I \vert } \frac{1}{t-t_0} \int _{t_0}^t (s'-t_0) \left| (Z^J \Gamma ) \left( s', s' \frac{x}{t} \right) \right| \,\mathrm{d}s' \Bigg ]\\&+ Cs \sum _{\vert J \vert \le \vert I \vert } \int _{s}^t \left| (Z^J \Gamma ) \left( s', s' \frac{x}{t} \right) \right| \,\mathrm{d}s' \\&+ C \sum _{\vert J \vert \le \vert I \vert } \int _{t_0}^s (s'-t_0) \left| (Z^J \Gamma ) \left( s', s' \frac{x}{t} \right) \right| \,\mathrm{d}s'. \end{aligned}$$

The proof of the bound (4.24) follows after dividing by *s* and using the fact that

$$\begin{aligned} \int _{t_0}^t \frac{s'-t_0}{t-t_0} \left| (Z^J \Gamma ) \left( s', s' \frac{x}{t} \right) \right| \,\mathrm{d}s'\lesssim & {} \int _s^t \left| (Z^J \Gamma ) \left( s', s' \frac{x}{t} \right) \right| \,\mathrm{d}s' \\&+ \int _{t_0}^s \frac{s'}{s} \left| (Z^J \Gamma ) \left( s', s' \frac{x}{t} \right) \right| \,\mathrm{d}s', \end{aligned}$$

since $\frac{s'-t_0}{t-t_0} = \frac{s'}{s'+s}\frac{s' + s}{t-t_0} - \frac{t_0}{t-t_0}$ and $1 \lesssim \frac{s'}{s'+s} \lesssim 1$ if $s\le s'$, and $\frac{s'}{s} \lesssim \frac{s'}{s'+s} \lesssim \frac{s'}{s}$ if $s' \le s$ (recall also that $t \ge t_0+1$). The proof of (4.25) follows similarly by writing

$$\begin{aligned} \frac{X_2^i(s,t,x,{\,\widehat{\!p\!}\,\,})}{s} - \frac{x^i}{t} =&\, \left( {\,\widehat{\!p\!}\,\,}^i - \frac{x^i}{t} \right) - \frac{t_0}{s} {\,\widehat{\!p\!}\,\,}^i + \frac{X_2^i(t_0,t,x,{\,\widehat{\!p\!}\,\,})}{s}\\&+ \frac{(s-t_0)}{s} \int _s^t {\,\widehat{\!\Gamma \!}\,\,}^i \left( s', s' \frac{x}{t}, \frac{x}{t} \right) \,\mathrm{d}s' \\&+ \frac{1}{s} \int _{t_0}^s (s' - t_0) {\,\widehat{\!\Gamma \!}\,\,}^i \left( s', s' \frac{x}{t}, \frac{x}{t} \right) \,\mathrm{d}s', \end{aligned}$$

and using the bound (4.20). The bound (4.27) similarly follows from Proposition 4.9 after writing

$$\begin{aligned} \frac{x^i}{t} - {\,\widehat{\!P\!}\,\,}^i_2(s,t,x,{\,\widehat{\!p\!}\,\,}) = \frac{x^i}{t} - {\,\widehat{\!p\!}\,\,}^i - \int _s^t {\,\widehat{\!\Gamma \!}\,\,}^i \left( s', s'\frac{x}{t}, \frac{x}{t} \right) \,\mathrm{d}s'. \end{aligned}$$

The lower order estimates (4.26) and (4.28) follow from rewriting

$$\begin{aligned} \int _{t_0}^t \left| (Z^J \Gamma ) \left( s', s'\frac{x}{t} \right) \right| \frac{s'}{s'+s} \,\mathrm{d}s'\lesssim & {} \int _s^t \left| (Z^J \Gamma ) \left( s', s' \frac{x}{t} \right) \right| \,\mathrm{d}s' \\&+ \int _{t_0}^s \frac{s'}{s} \left| (Z^J \Gamma ) \left( s', s' \frac{x}{t} \right) \right| \mathrm{d}s', \end{aligned}$$

and using the pointwise bounds (4.1) for lower order derivatives of $\Gamma$. $\quad \square$

### Schematic Notation and Repeated Vector Fields Applied to Differences

To make long expressions more concise, the *s* dependence of many quantities is suppressed throughout this section.

The proofs of Propositions 4.23 and 4.26 below follow from applying vector fields to the system (2.14)–(2.15). It is therefore necessary to estimate vector fields applied to the difference

4.29 $$\begin{aligned}&\widehat{\Gamma }(X,\widehat{P})-\widehat{\Gamma }(X_1,\widehat{P}_1) = \Gamma (X)\cdot \Lambda (\widehat{P})-\Gamma (X_1)\cdot \Lambda (\widehat{P}_1) \nonumber \\&\quad = (\Gamma (X)-\Gamma (X_1))\cdot \Lambda (\widehat{P})+\Gamma (X_1)\cdot (\Lambda (\widehat{P})-\Lambda (\widehat{P}_1)), \end{aligned}$$

which appears on the right hand side of equation (2.15), where $X_1(s)=sx/t$ and $\widehat{P}_1= \frac{\mathrm{d}X_1}{\mathrm{d}s} = x/t$, and $\Lambda$ is defined in (2.4). Apart from controlling vector fields applied to $X_1$ and $\widehat{P}_1$ we also control vector fields applied to $X_2$ and $\widehat{P}_2$, and moreover the differences $X_1-X_2$ and $\widehat{P}_1-\widehat{P}_2$ decay, see (4.13) and (4.14) and the propositions to follow. Furthermore, because of the definition of $X_2$ and $\widehat{P}_2$ in terms of $X_1$ and $\widehat{P}_1$ the differences $\overline{X}=X-X_2$ and $\overline{P}=\widehat{P}-\widehat{P}_2$ are small, see Proposition 2.3. It may be tempting to write this as differences with $\widehat{\Gamma }$ evaluated at $(X_2,P_2)$. However we do not want to involve an estimate of $\Gamma$ applied to $X_2$ so instead we will first differentiate (4.29) and use the decomposition $X_1-X=X_1-X_2-\overline{X}$ to the factors that come out when we differentiated. The following result is straightforward to show:

#### Lemma 4.11

Given $Y_1,\dots ,Y_k,Y\in \mathbb {R}^3$ and $F:\mathbb {R}^3\rightarrow \mathbb {R}$, let $Y_1\cdots Y_k \cdot (\partial ^k F)(Y)$, denote the sum of $Y_1^{j_1}\cdots Y_k^{j_k} \cdot (\partial _{j_1} \cdots \partial _{j_k}F)(Y)$ over all components $1\le j_i\le 3$ for $i=1,\dots , k$. We have

4.30 $$\begin{aligned} \overline{Z}^J (F(X)-F(X_1))= & {} \sum _{\begin{array}{c} k<|J|/2,\, J_1+\dots +J_k=J,\, 1\le |J_1|\le \dots \le |J_k| \end{array}\!\!\!\!\!\!\!\!\!\!\!\!\!\!\!\!\!\!\!\!\!\!} c_{J_1\dots J_k}\,\,\,\, \overline{Z}^{J_1} X_1\nonumber \\&\quad \cdots \overline{Z}^{J_{k}}X_1\cdot \big ((\partial ^k F)(X)-(\partial ^k F)(X_1)\big )\nonumber \\&+ \sum _{\begin{array}{c} k<|J|/2,\, J_1+\dots +J_k=J,\, 1\le |J_1|\le \dots \le |J_k|\!\!\!\!\!\!\!\!\!\!\!\!\! \end{array}\!\!\!\!\!\!\!} c_{J_1\dots J_k}\sum _{1\le \ell \le k} \overline{Z}^{J_1} X\nonumber \\&\quad \cdots \overline{Z}^{J_{\ell -1}}X\,\, \overline{Z}^{J_\ell }(X-X_1) \,\, \overline{Z}^{J_{\ell +1}} X_1\cdots \overline{Z}^{J_{k}}X_1\cdot (\partial ^k F)(X),\nonumber \\&+ \sum _{\begin{array}{c} k\ge |J|/2,\, J_1+\dots +J_k=J,\, 1\le |J_1|\le \dots \le |J_k|\!\!\!\!\!\!\!\!\!\!\!\!\! \end{array} \!\!\!\!\!\!\!\!\!\!\!} c^\prime _{J_1\dots J_k}\,\,\,\, \overline{Z}^{J_1} X\nonumber \\&\quad \cdots \overline{Z}^{J_{k}}X\cdot \big ((\partial ^k F)(X)-(\partial ^k F)(X_1)\big )\nonumber \\&+ \sum _{\begin{array}{c} k\ge |J|/2,\, J_1+\dots +J_k=J,\, 1\le |J_1|\le \dots \le |J_k|\!\!\!\!\!\!\!\!\!\!\!\!\! \end{array}\!\!\!} c^\prime _{J_1\dots J_k}\sum _{1\le \ell \le k} \overline{Z}^{J_1} X_1\nonumber \\&\quad \cdots \overline{Z}^{J_{\ell -1}}X_1\,\, \overline{Z}^{J_\ell }(X-X_1) \,\, \overline{Z}^{J_{\ell +1}} X\cdots \overline{Z}^{J_{k}}X\cdot (\partial ^k F)(X_1),\nonumber \\ \end{aligned}$$

where the sums are over all possible partitions of the multi index *J* into nonempty sub indices $J_1$ to $J_k$.

#### Proof

First one differentiates to get a sum of terms of the form

$$\begin{aligned} \overline{Z}^{I_1} X \cdots \overline{Z}^{I_k} X \cdot (\partial ^k F)(X) -\overline{Z}^{I_1} X_1 \cdots \overline{Z}^{I_k} X_1 \cdot (\partial ^k F)(X_1), \end{aligned}$$

and then one makes a different decomposition depending on the size of *k*. Then one proceeds by organizing them in order so $|I_1|$ is smallest. If $k<|J|/2$, one writes

$$\begin{aligned}&\overline{Z}^{I_1} X \cdots \overline{Z}^{I_k} X \cdot (\partial ^k F)(X) -\overline{Z}^{I_1} X_1 \cdots \overline{Z}^{I_k} X_1 \cdot (\partial ^k F)(X_1)\\&\quad = \overline{Z}^{I_1} X \cdots \overline{Z}^{I_k} X \cdot ((\partial ^k F)(X)- (\partial ^k F)(X_1)) \\&\qquad +(\overline{Z}^{I_1} X \cdots \overline{Z}^{I_k} X -\overline{Z}^{I_1} X_1 \cdots \overline{Z}^{I_k} X_1) \cdot (\partial ^k F)(X_1), \end{aligned}$$

and replaces them one by one:

$$\begin{aligned}&\overline{Z}^{I_1} X \cdots \overline{Z}^{I_k} X = \overline{Z}^{I_1} X\cdots \overline{Z}^{I_k} (X-X_1)+ \overline{Z}^{I_1} X \cdots \overline{Z}^{I_k} X_1\\&\quad = \overline{Z}^{I_1} X\cdots \overline{Z}^{I_k} (X-X_1)+\overline{Z}^{I_1} X \cdots \overline{Z}^{I_{k-1}} (X-X_1)\overline{Z}^{I_k} X_1\\&\qquad +\overline{Z}^{I_1} X \cdots \overline{Z}^{I_{k-1}}X_1 \overline{Z}^{I_k} X_1=.... \end{aligned}$$

This produces the first two sums with $k<|J|/2$. For $k\ge |J|/2$ one simply does the same thing but with $X_1$ and *X* interchanged. $\quad \square$

Note that, for each term in the equality (4.30), $|J_i|\le |J|/2$ if $i\ne k$ and therefore, in the applications of Lemma 4.11 below, the factors $\overline{Z}^{J_i}\! X$ can be estimated by the their $L^\infty$ norms using induction by previous estimates. Given that $X_1$ is known the expression (4.30) can be thought of as linear in the unknown $X-X_1$. However when we prove $L^2$ estimates for high derivatives we also have to take into account how $Z^{J_k} X_1$ depends on high derivatives of $\Gamma$. Either $k\le |J|/2$ is small, in which case we can use $L^\infty$ estimates for $\partial ^k F$ and $\partial ^{k+1} F$, or $k\ge |J|/2$ is large, and as a result $|J_i|\le |J|/2$ are small for all *i*, and we can use $L^\infty$ estimates for all the other factors. Applying Lemma 4.11 we get the following estimates:

#### Lemma 4.12

Recall the function $\Lambda$ from (4.29). Suppose $t\ge t_0 + 1$, $\vert x \vert \le c t$, $(t,x,{\,\widehat{\!p\!}\,\,}) \in \mathrm {supp}(f)$ and suppose, for some $t_0 \le s \le t$, that $|\overline{Z}^{K} \widehat{P}(s,t,x,{\,\widehat{\!p\!}\,\,})| \le C$ for $|K|\le |L|/2$. Then

4.31 $$\begin{aligned} \big |\overline{Z}^L \big (\Lambda (\widehat{P}(s,t,x,{\,\widehat{\!p\!}\,\,}))-\Lambda (\widehat{P}_1(t,x))\big )\big |&\lesssim \sum _{|M|\le |L|} \big |\overline{Z}^{M} (\widehat{P}(s,t,x,{\,\widehat{\!p\!}\,\,})-\widehat{P}_1(t,x))\big | , \end{aligned}$$

4.32 $$\begin{aligned} \big |\overline{Z}^L \big (\Lambda (\widehat{P}_1(t,x))\big )\big |&\lesssim 1. \end{aligned}$$

#### Proof

The bound (4.31) follows from Lemma 4.11 applied to $\Lambda ({\,\widehat{\!P\!}\,\,})$, after noting that $\vert \overline{Z}^I ( {\,\widehat{\!P\!}\,\,}_1) \vert \lesssim 1$ for any *I*, using the form of the vector fields $\overline{Z}$ and the fact that ${\,\widehat{\!P\!}\,\,}_1(t,x) = \frac{x}{t}$. The term $(\partial ^k \Lambda )({\,\widehat{\!P\!}\,\,}) - (\partial ^k \Lambda )({\,\widehat{\!P\!}\,\,}_1)$ is estimated by

$$\begin{aligned} \left| (\partial ^k \Lambda )({\,\widehat{\!P\!}\,\,}) - (\partial ^k \Lambda )(\widehat{P}_1) \right| \lesssim \left| {\,\widehat{\!P\!}\,\,}- \widehat{P}_1 \right| , \end{aligned}$$

since $\Lambda$ is smooth. The bound (4.32) is even simpler. $\quad \square$

#### Lemma 4.13

Suppose $t\ge t_0 + 1$, $\vert x \vert \le c t$, $(t,x,{\,\widehat{\!p\!}\,\,}) \in \mathrm {supp}(f)$ and suppose, for some $t_0 \le s \le t$, that $|\overline{Z}^{K} X(s,t,x,{\,\widehat{\!p\!}\,\,})| \le Cs$ for $|K|\le |J|/2$. Then

$$\begin{aligned} \big |\overline{Z}^J \big (\Gamma (X)-\Gamma (X_1)\big )\big | \lesssim&\sum _{|K|\le |J|/2\!\!\!\!\!\!\!\!\!\!\!\!\!\!\!\!}\big |(Z^{K} \Gamma )(X)\big | \sum _{|M|\le |J|\!\!\!\!\!\!\!\!\!\!} \frac{|\overline{Z}^M (X-X_1)|}{s} \\&+ \sum _{|M|\le |J|\!\!\!\!\!\!\!\!}\big |(Z^{M} \Gamma )(X)-(Z^{M} \Gamma )(X_1)\big |\\&+ \sum _{|K|\le |J|/2\!\!\!\!\!\!\!\!\!\!\!\!\!\!\!} \frac{|\overline{Z}^{K} (X-X_1)|}{s}\sum _{|M|\le |J|\!\!\!\!\!\!\!\!\!}\big |(Z^{M} \Gamma )(X_1)\big |, \end{aligned}$$

and

$$\begin{aligned} \big |\overline{Z}^J\big (\Gamma (X_1)\big )\big | \lesssim \sum _{|M|\le |J|\!\!\!\!\!\!\!\!\!\!\!\!\!\!\!\!}\big |(Z^{M} \Gamma )(X_1)\big |, \end{aligned}$$

at $$(s,t,x,{\,\widehat{\!p\!}\,\,})$$.

#### Proof

The Lemma is again a straightforward application of Lemma 4.11, noting again that $\left| \frac{\overline{Z}^I X_1}{s} \right| \lesssim 1$ for any *I* since $X_1(s,t,x) = s \frac{x}{t}$. Note that

4.33 $$\begin{aligned} \partial ^k \Gamma (t,x)=t^{-k} \sum _{|K|\le k} A_K(x/t) (Z^K \Gamma )(t,x) \end{aligned}$$

for some homogeneous functions *A* that are smooth when $|x|/t\le c<1$, and hence,

4.34 $$\begin{aligned} s^k \left| (\partial ^k \Gamma )(X) - (\partial ^k \Gamma )(X_1) \right|\lesssim & {} \sum _{\vert K \vert \le k} \left| (Z^K \Gamma )(X) - (Z^K \Gamma )(X_1) \right| \nonumber \\&+ \frac{|X-X_1|}{s}\sum _{|K|\le k}\big |(Z^{K} \Gamma )(X_1)\big |. \end{aligned}$$

$$\square $$

In the application we will estimate low derivatives of $\Gamma$ with its $L^\infty$ norm so that the result only depends on the differences of functions evaluated at *X* and $X_1$ and functions evaluated at $X_1$ but not at *X*.

#### Lemma 4.14

Suppose $t\ge t_0 + 1$, $\vert x \vert \le c t$, $(t,x,{\,\widehat{\!p\!}\,\,}) \in \mathrm {supp}(f)$ and suppose, for some $t_0 \le s \le t$, that $|\overline{Z}^{K} X| \le Cs$ for $|K|\le |J|/2$. Then, at $(s,t,x,{\,\widehat{\!p\!}\,\,})$,

$$\begin{aligned} \big |\overline{Z}^J \big (\Gamma (X)-\Gamma (X_1)\big )\big | \lesssim&\sum _{|K|\le |J|/2\!\!\!\!\!\!\!\!\!\!\!\!\!\!\!} \big \Vert (Z^{K} \Gamma )(s,\cdot )\big \Vert _{L^{\infty }} \sum _{|M|\le |J|\!\!\!\!\!\!\!\!\!\!} \frac{|\overline{Z}^M (X-X_1)|}{s} \\&+\sum _{|M|\le |J|\!\!\!\!\!\!\!\!}\big |(Z^{M} \Gamma )(X)-(Z^{M} \Gamma )(X_1)\big |\\&+\sum _{|K|\le |J|/ 2\!\!\!\!\!\!\!\!\!\!\!\!\!\!\!} \frac{|\overline{Z}^{K} (X-X_1)|}{s} \sum _{|M|\le |J|\!\!\!\!\!\!\!\!\!}\big |(Z^{M} \Gamma )(X_1)\big |,\\ \big |\overline{Z}^J\big (\Gamma (X_1)\big )\big | \lesssim&\sum _{|M|\le |J|\!\!\!\!\!\!}\big |(Z^{M} \Gamma )(X_1)\big |. \end{aligned}$$

Instead of applying vector fields to the decomposition (4.29) we first apply vector fields and then apply this decomposition to the terms with more derivatives falling on $\Gamma$, but if more fall on $\Lambda$ we apply the decomposition with $\Lambda (\widehat{P})$ interchanged with $\Lambda (\widehat{P}_1)$ and $\Gamma (X)$ with $\Gamma (X_1)$:

$$\begin{aligned} \overline{Z}^I\big (\widehat{\Gamma }(X,\widehat{P})-\widehat{\Gamma }(X_1,\widehat{P}_1)\big )= & {} \sum _{J+L=I,\, |J|\ge |I|/2, |L|\le |I|/2} \!\!\!\!\! \overline{Z}^J(\Gamma (X)-\Gamma (X_1))\cdot \overline{Z}^L \Lambda (\widehat{P})\\&+\overline{Z}^J\Gamma (X_1)\cdot \overline{Z}^L(\Lambda (\widehat{P})- \Lambda (\widehat{P}_1))\\&+\sum _{J+L=I,\, |J|<|I|/2,|L|>|I|/2} \overline{Z}^J(\Gamma (X)-\Gamma (X_1))\cdot \overline{Z}^L \Lambda (\widehat{P}_1)\\&+\overline{Z}^J\Gamma (X)\cdot \overline{Z}^L(\Lambda (\widehat{P})-\Lambda (\widehat{P}_1)). \end{aligned}$$

Using this decomposition and the previous lemmas we obtain

#### Proposition 4.15

Suppose $t\ge t_0 + 1$, $\vert x \vert \le c t$, $(t,x,{\,\widehat{\!p\!}\,\,}) \in \mathrm {supp}(f)$ and suppose, for some $t_0 \le s \le t$, that $|\overline{Z}^{K} X|/s + |\overline{Z}^{K} \widehat{P}| \le C$ for $|K|\le |I|/2$. Then, at $(s,t,x,{\,\widehat{\!p\!}\,\,})$,

$$\begin{aligned}&\big |\overline{Z}^I\big (\widehat{\Gamma }(X,\widehat{P})-\widehat{\Gamma }(X_1,\widehat{P}_1)\big )\big |\\&\quad \lesssim \sum _{|K|\le |I|/2\!\!\!\!\!\!\!\!} \!\big \Vert (Z^{K} \Gamma )(s,\cdot )\big \Vert _{L^{\infty }} \sum _{|J|\le |I|\!\!\!\!\!\!}\Big ( \frac{|\overline{Z}^J \!(X - X_1)| }{s}+\big |\overline{Z}^{J} \!(\widehat{P} - \widehat{P}_1)\big |\Big )\\&\qquad +\sum _{|J|\le |I|\!\!\!\!\!\!\!\!}\Big (\big |(Z^{J} \Gamma )(X)-(Z^{J} \Gamma )(X_1)\big |\\&\qquad +\sum _{|K|\le |I|/2\!\!\!\!\!\!\!\!\!\!\!\!\!\!\!} \Big (\frac{|\overline{Z}^{K} \!(X-X_1)|}{s}+\big |\overline{Z}^{K}\! (\widehat{P}-\widehat{P}_1)\big |\Big ) \big |(Z^{J} \Gamma )(X_1)\big |\Big ), \end{aligned}$$

and

$$\begin{aligned} \big |\overline{Z}^J\big (\widehat{\Gamma }(X_1,P_1)\big )\big | \lesssim \sum _{|M|\le |J|\!\!\!\!\!\!\!\!\!\!}\big |(Z^{M} \Gamma )(X_1)\big |. \end{aligned}$$

#### Proof

The above decomposition and Lemma 4.12 give

$$\begin{aligned} \Big |\overline{Z}^I \big (\Gamma (X)\cdot \Lambda (\widehat{P})-\Gamma (X_1)\cdot \Lambda (\widehat{P}_1)\big )\Big | \lesssim&\sum _{|J|\le |I|\!\!\!\!\!\!} \big |\overline{Z}^J(\Gamma (X)-\Gamma (X_1))\big |\\&+ \big |\overline{Z}^J(\Gamma (X_1))| \sum _{|L|\le |J|/2\!\!\!\!\!\!\!\!\!\!} \big | \overline{Z}^L\big (\widehat{P}-\widehat{P}_1\big )\big |\\&+\sum _{|J|\le |I|/2\!\!\!} \big |\overline{Z}^J(\Gamma (X))| \sum _{|L|\le |I|\!\!\!\!\!\!\!\!} \big |\overline{Z}^{L} (\widehat{P}-\widehat{P}_1)\big | . \end{aligned}$$

The first bound then follows from Lemma 4.14. The proof of the second bound is straightforward. $\quad \square$

The following corollary of Proposition 4.15 is used at lower orders:

#### Corollary 4.16

Suppose $t\ge t_0 + 1$, $\vert x \vert \le c t$, $(t,x,{\,\widehat{\!p\!}\,\,}) \in \mathrm {supp}(f)$ and suppose, for some $t_0 \le s \le t$, that $|\overline{Z}^{K}\! X|/s+|\overline{Z}^{K} \!\widehat{P}|\le C$, for $|K|\le |I|/2$. Then, at $(s,t,x,{\,\widehat{\!p\!}\,\,})$,

$$\begin{aligned} \big |\overline{Z}^I\big (\widehat{\Gamma }(X,\widehat{P})-\widehat{\Gamma }(X_1,\widehat{P}_1)\big )\big |&\lesssim \sum _{|K|\le |I|+1\!\!\!\!\!\!\!\!\!\!\!\!\!}\big \Vert (Z^{K} \Gamma )(s,\cdot )\big \Vert _{L^{\infty }} \\&\quad \sum _{|J|\le |I|\!\!\!} \Big (\frac{|\overline{Z}^J (X\!-\!X_1)|\!}{s}+\big |\overline{Z}^{J} (\widehat{P}\!-\!\widehat{P}_1)\big | \Big ),\\ \big |\overline{Z}^I\big (\widehat{\Gamma }(X_1,\widehat{P}_1)\big )\big |&\lesssim \sum _{|K|\le |I|\!\!\!\!\!\!\!\!\!\!\!\!\!}\big \Vert (Z^{K} \Gamma )(s,\cdot )\big \Vert _{L^{\infty }} . \end{aligned}$$

#### Proof

The proof follows from Proposition 4.15 if we note that in the support of *X* and $X_1$,

4.35 $$\begin{aligned} \big |(Z^{J} \Gamma )(X)-(Z^{J} \Gamma )(X_1)\big |\lesssim & {} \Vert (\partial Z^{J} \Gamma )(s,\cdot )\Vert _{L^\infty } |X\!-\!X_1| \nonumber \\\lesssim & {} \sum _{|K|=|J|+1}\Vert (Z^{K} \Gamma )(s,\cdot )\Vert _{L^\infty } |X\!-\!X_1|/s . \end{aligned}$$

$$\square $$

### Parameter Derivatives of the Equations and Vector Fields Applied to Their Differences

The vector fields applied to the system (2.14)–(2.15) will be estimated by integrating from the final time *t* and in order to do this we need to control the final conditions for $\overline{Z}^I\overline{X}$ and $\overline{Z}^I\overline{P}$ at time *t*. Note that, for $i,j=1,2,3$, $(\partial _{x^i} X(s,t,x,{\,\widehat{\!p\!}\,\,})^j)\vert _{s=t} = \partial _{x^i}(X(t,t,x,{\,\widehat{\!p\!}\,\,}))$ (similarly for $\partial _{{\,\widehat{\!p\!}\,\,}^i}$ in place of $\partial _{x^i}$ and for $X_2, {\,\widehat{\!P\!}\,\,}, {\,\widehat{\!P\!}\,\,}_{\!\! 2}$ respectively in place of *X*) and so, if $\overline{Z}$ is a vector which does not involve $\partial _t$ derivatives, these final conditions vanish. Some of our vector fields, however, also involve $\partial _t$ derivatives. Therefore we need to estimate higher $\frac{\mathrm{d}}{\mathrm{d}s}$ derivatives of the system, which can be recast as spacetime derivatives. Recall that ${\,\widehat{\!X\!}\,\,}(s) = (s,X(s))$. We have

4.36 $$\begin{aligned} \frac{\mathrm{d}{\,\widehat{\!X\!}\,\,}}{\mathrm{d}s}={\,\widehat{\!P\!}\,\,},\quad \frac{\mathrm{d}{\,\widehat{\!P\!}\,\,}}{\mathrm{d}s}=\Gamma ({\,\widehat{\!X\!}\,\,})\cdot \Lambda ({\,\widehat{\!P\!}\,\,}). \end{aligned}$$

The structure of higher order $\frac{\mathrm{d}}{\mathrm{d}s}$ derivatives is very simple. Either the derivative falls on $\Lambda ({\,\widehat{\!P\!}\,\,})$, in which case we can substitute the second equation for $d{\,\widehat{\!P\!}\,\,}\!/\mathrm{d}s$ and get another factor of $\Gamma ({\,\widehat{\!X\!}\,\,})$, or the derivative falls $\Gamma ({\,\widehat{\!X\!}\,\,})$, which produces a derivative $\partial \Gamma$. Hence we get the system

4.37 $$\begin{aligned} \frac{\mathrm{d}\widehat{X}^{(k)}\!\!\!\!}{\mathrm{d}s}=\widehat{P}^{(k)},\quad \frac{\mathrm{d}\widehat{P}^{(k)\!\!\!\!}}{\mathrm{d}s}=\widehat{\Gamma }^{(k)}({\,\widehat{\!X\!}\,\,},{\,\widehat{\!P\!}\,\,}),\quad \text {where}\quad \widehat{X}^{(k)} =\frac{\mathrm{d}^k\widehat{X}}{\mathrm{d}s^k},\quad \widehat{P}^{(k)} =\frac{\mathrm{d}^k{\,\widehat{\!P\!}\,\,}}{\mathrm{d}s^k}, \end{aligned}$$

where

4.38 $$\begin{aligned} \widehat{\Gamma }^{(k)}({\,\widehat{\!X\!}\,\,},{\,\widehat{\!P\!}\,\,})= & {} {\Gamma }^{(k)}({\,\widehat{\!X\!}\,\,})\cdot \Lambda ^{(k)}({\,\widehat{\!P\!}\,\,})\nonumber \\:= & {} \sum _{\begin{array}{c} k_1+\dots +k_m+m=k+1,\\ 0\le k_1\le \dots \le k_m \le k \end{array}\!\!\!\!} \,\,(\partial ^{k_1}\Gamma )({\,\widehat{\!X\!}\,\,})\cdots (\partial ^{k_m}\Gamma )({\,\widehat{\!X\!}\,\,})\cdot \Lambda _{k_1,\dots ,k_m}({\,\widehat{\!P\!}\,\,}).\nonumber \\ \end{aligned}$$

Here $\partial ^k \Gamma$, denote the $\mathbb {R}^{4k}$ tensor with components sum of $\partial _{j_1} \cdots \partial _{j_k} \Gamma$ over all components $0\le j_i\le 3$ for $i=1,\dots , k$ and $\Lambda _{k_1,\dots ,k_m}({\,\widehat{\!P\!}\,\,})$ are polynomials in ${\,\widehat{\!P\!}\,\,}$ with values in $\mathbb {R}^{4k_1+\dots 4k_m}$. Here the first dot product is schematic notation to be interpreted as dot products of elements $\Gamma ^{(k)}$ and $\Lambda ^{(k)}$ in some larger dimensional space whose components corresponds to the terms in the sum. We now also want to take $\frac{\mathrm{d}}{\mathrm{d}s}$ derivatives of

4.39 $$\begin{aligned} \frac{\mathrm{d}\widehat{X}_2}{\mathrm{d}s}=\widehat{P}_2,\quad \frac{\mathrm{d}\widehat{P}_2}{\mathrm{d}s}=\Gamma (\widehat{X}_1)\cdot \Lambda (\widehat{P}_1 ),\quad \text {and}\quad \frac{\mathrm{d}\widehat{X}_1}{\mathrm{d}s}=\widehat{P}_1,\quad \frac{\mathrm{d}\widehat{P}_1}{\mathrm{d}s}=0. \end{aligned}$$

Then

4.40 $$\begin{aligned}&\frac{\mathrm{d}\overline{X}^{(k)}\!\!\!\!}{\mathrm{d}s}=\overline{P}^{(k)}\!\!\!\!,\quad \frac{\mathrm{d}\overline{P}^{(k)\!\!\!\!}}{\mathrm{d}s} =\widehat{\Gamma }^{(k)}({\,\widehat{\!X\!}\,\,}\!,{\,\widehat{\!P\!}\,\,})-\widehat{\Gamma }^{(k)}({\,\widehat{\!X\!}\,\,}_{\!\!1},{\,\widehat{\!P\!}\,\,}_{\!\!1}) +\widehat{\Gamma }^{(k,2)}({\,\widehat{\!X\!}\,\,}_{\!\!1},{\,\widehat{\!P\!}\,\,}_{\!\!1}),\nonumber \\&\quad \text {if}\quad \overline{X}^{(k)}\!\! =\frac{\mathrm{d}^k\overline{X}\!}{\mathrm{d}s^k},\quad \overline{P}^{(k)} \!\! =\frac{\mathrm{d}^k\overline{P}\!}{\mathrm{d}s^k}, \end{aligned}$$

where

4.41 $$\begin{aligned}&\widehat{\Gamma }^{(k,2)}({\,\widehat{\!X\!}\,\,},{\,\widehat{\!P\!}\,\,})\!={\Gamma }^{(k,2)}({\,\widehat{\!X\!}\,\,})\cdot \Lambda ^{(k)}({\,\widehat{\!P\!}\,\,})\nonumber \\&\quad =\sum _{\begin{array}{c} k_1+\cdot +k_m+m=k+1,\\ 0\le k_1\le \dots \le k_m \le k,\, m\ge 2 \end{array}\!\!\!\!} \,\,(\partial ^{k_1}\Gamma )({\,\widehat{\!X\!}\,\,})\cdots (\partial ^{k_m}\Gamma )({\,\widehat{\!X\!}\,\,})\cdot \Lambda _{k_1,\dots ,k_m}({\,\widehat{\!P\!}\,\,}). \end{aligned}$$

Here $\Gamma ^{(k)}$ satisfies the same estimates as $\partial ^k\Gamma$ whereas $\Gamma ^{(k,2)}$ is at least quadratic in $\Gamma$ (note $m\ge 2$ in the summation) and hence satisfies the same estimates as $\partial ^{k-1}\Gamma$ multiplied with $\Gamma$, which decays $s^{-a}$ faster than another derivative. We have

#### Lemma 4.17

Suppose that $t|Z^I \Gamma (t,x)|\lesssim 1$ for $|I|\le (|L|+k)/2$. Then

4.42 $$\begin{aligned} t^k |Z^L \Gamma ^{(k)}(t,x)|&\lesssim \sum _{|I|\le |L|+k} |Z^I \Gamma (t,x)|,\end{aligned}$$

4.43 $$\begin{aligned} t^k |Z^L \Gamma ^{(k,2)}(t,x)|&\lesssim \sum _{|I|\le (|L|+k)/2} t |Z^I \Gamma (t,x)| \sum _{|I|\le |L|+k} |Z^I \Gamma (t,x)|. \end{aligned}$$

Moreover,

4.44 $$\begin{aligned}&s^k\big |(Z^{L} \Gamma ^{(k)})(X)-(Z^{L} \Gamma ^{(k)})(X_1)\big | \lesssim \sum _{|J|\le |L|+k\!\!\!\!\!}\big |(Z^{J} \Gamma )(X)-(Z^{J} \Gamma )(X_1)\big |\nonumber \\&\quad +\frac{|X-X_1|}{s} \big |(Z^{J} \Gamma )(X_1)\big |. \end{aligned}$$

#### Proof

The proof of (4.44) follows from using (4.33)–(4.34). We first note that the components of $Z^J \partial ^k \Gamma$ are linear combinations of the components of $\partial ^k Z^K\Gamma$, for $|K|\le |J|$, since the commutator $[Z,\partial _\alpha ]$ is either 0 or $\partial _\beta$ for some $\beta$. Therefore it remains to estimate the difference $F_{k_1\dots k_m}^{J_1\dots J_m}({\,\widehat{\!X\!}\,\,})-F_{k_1\dots k_m}^{J_1\dots J_m}({\,\widehat{\!X\!}\,\,}_{\!\!1})$, where

$$\begin{aligned}&F_{k_1\dots k_m}^{J_1\dots J_m}(t,x)=(\partial ^{k_1}Z^{J_1}\Gamma )(t,x)\cdots (\partial ^{k_m}Z^{J_m}\Gamma )(t,x),\\&\quad \text {for } k_1+\dots +k_m\!+m=k+1,\quad |J_1|+\dots +|J_m|\le |L|. \end{aligned}$$

Using (4.33) we can write this as a linear combination of

$$\begin{aligned}&G^{I_1\dots I_m}(t,x)=t^{-k-1+m}A_{I_1\dots I_m} (x/t)(Z^{I_1}\Gamma )(t,x)\cdots (Z^{I_m}\Gamma )(t,x),\\&\quad |I_1|+\dots +|I_m|\le |L|+k+1-m. \end{aligned}$$

The difference $G^{I_1\dots I_m}({\,\widehat{\!X\!}\,\,}\!\!)-G^{I_1\dots I_m}({\,\widehat{\!X\!}\,\,}_{\!\!1}\!)$ can be estimated by the right hand side of (4.44) as for (4.34). $\quad \square$

We can now apply the estimates from the previous section with $\widehat{\Gamma }^{(k)}$ respectively $\widehat{\Gamma }^{(k,2)}$ in place of $\widehat{\Gamma }$. First we obtain the analogue of Proposition 4.15 as follows:

#### Proposition 4.18

Suppose that for some $k\!\ge \! 0$ and *s*, such that $t_0\!\le \!s\!\le \! t$, we have that $|\overline{Z}^{K}\! X|/s+|\overline{Z}^{K} \!\widehat{P}| \!\le \! C\!$, for $|K|\le |L|/2$, and $s|Z^I \Gamma (s,x)|\lesssim 1$, for $|I|\le (|L|+k)/2$. Then

$$\begin{aligned}&s^k \big |\overline{Z}^L\big (\widehat{\Gamma }^{(k)}(X,\widehat{P})-\widehat{\Gamma }^{(k)}(X_1,\widehat{P}_1)\big )\big |\\&\quad \lesssim \!\sum _{|K|\le (|L|+k)/2+1\!\!\!\!}\!\big \Vert (Z^{K} \Gamma )(s,\cdot )\big \Vert _{L^{\infty }} \!\!\sum _{|J|\le |L| \!\!\!\!\!}\Big ( \frac{|\overline{Z}^J \!(X\!\!-\!\!X_1)|\!}{s}+\big |\overline{Z}^{J} \!(\widehat{P}\!-\!\widehat{P}_1)\big |\Big )\\&\qquad +\sum _{|J|\le |L|+k\!\!}\Big (\big |(Z^{J} \Gamma )(X)-(Z^{J} \Gamma )(X_1)\big | \\&\qquad +\sum _{|K|\le |L|/2\!\!\!\!\!\!\!\!\!\!\!\!} \Big (\frac{|\overline{Z}^{K} \!(X-X_1)|}{s}+\big |\overline{Z}^{K}\! (\widehat{P}-\widehat{P}_1)\big |\Big ) \big |(Z^{J} \Gamma )(X_1)\big |\Big ), \end{aligned}$$

and

$$\begin{aligned}&s^k \big |\overline{Z}^L\big (\widehat{\Gamma }^{(k,2)}(X_1,P_1)\big )\big | \lesssim \sum _{|K|\le (|L|+k)/2\!\!}s\big \Vert (Z^{K} \Gamma )(s,\cdot )\big \Vert _{L^{\infty }}^2 \\&\quad + \sum _{|K|\le (|L|+k)/2\!\!\!}s\big \Vert (Z^{K} \Gamma )(s,\cdot )\big \Vert _{L^{\infty }} \sum _{|M|\le |L|+k\!\!\!\!\!\!}\big |(Z^{M} \Gamma )(X_1)\big |. \end{aligned}$$

#### Proof

By Proposition 4.15 applied to $\widehat{\Gamma }^{(k)}$ in place of $\widehat{\Gamma }$,

$$\begin{aligned}&s^k\big |\overline{Z}^L\big (\widehat{\Gamma }^{(k)}(X,\widehat{P})-\widehat{\Gamma }^{(k)}(X_1,\widehat{P}_1)\big )\big |\\&\quad \lesssim \!\sum _{|K|\le |L|/2\!\!\!}\!s^k\big \Vert (Z^{K} \Gamma ^{(k)})(s,\cdot )\big \Vert _{L^{\infty }} \!\!\sum _{|J|\le |L| \!\!\!\!\!}\Big ( \frac{|\overline{Z}^J \!(X\!\!-\!\!X_1)|\!}{s}+\big |\overline{Z}^{J} \!(\widehat{P}\!-\!\widehat{P}_1)\big |\Big )\\&\qquad +s^k\sum _{|J|\le |L|\!\!\!\!\!}\Big (\big |(Z^{J} \Gamma ^{(k)})(X)-(Z^{J} \Gamma ^{(k)})(X_1)\big | \\&\qquad +\sum _{|K|\le |L|/2\!\!\!\!\!\!\!\!\!\!\!\!} \Big (\frac{|\overline{Z}^{K} \!(X-X_1)|}{s}+\big |\overline{Z}^{K}\! (\widehat{P}-\widehat{P}_1)\big |\Big ) \big |(Z^{J} \Gamma ^{(k)})(X_1)\big |\Big ), \end{aligned}$$

and the first part of the proposition follows from Lemma 4.17. $\quad \square$

The following corollary of Proposition 4.18 is used at lower orders:

#### Corollary 4.19

Suppose that for some $k\!\ge \! 0$ and *s*, such that $t_0\!\le \! s\!\le \! t$, we have that $|\overline{Z}^{K}\! X|/s+|\overline{Z}^{K} \!\widehat{P}|\!\le \!C\!$, for $|K|\le |L|/2$, $|\overline{Z}^{M}\!X_1|/s+|\overline{Z}^{M}\!\widehat{P}_1|\le C$, for $|M|\le |L|$, and $s|(Z^I \Gamma )(s,x)|\lesssim 1$, for $|I|\le (|L|+k)/2$. Then

$$\begin{aligned} s^k \big |\overline{Z}^L\big (\widehat{\Gamma }^{(k)}(X,\widehat{P})-\widehat{\Gamma }^{(k)}(X_1,\widehat{P}_1)\big )\big |&\lesssim \sum _{|K|\le |L|+k+1\!\!\!\!\!\!}\big \Vert (Z^{K} \Gamma )(s,\cdot )\big \Vert _{L^{\infty }} \\&\quad \sum _{|J|\le |L|\!\!\!} \Big (\frac{|\overline{Z}^J (X\!-\!X_1)|\!}{s}+\big |\overline{Z}^{J} (\widehat{P}\!-\!\widehat{P}_1)\big | \Big ),\\ s^k\big |\overline{Z}^L\big (\widehat{\Gamma }^{(k,2)}(X_1,\widehat{P}_1)\big )\big |&\lesssim \sum _{|K|\le |L|+k\!\!\!}s\big \Vert (Z^{K} \Gamma )(s,\cdot )\big \Vert _{L^{\infty }}^2 . \end{aligned}$$

The proof of Corollary 4.19 follows from Proposition 4.18 as in the proof of Corollary 4.16.

### The Final Conditions

Note that, for any $Y(s,t,x,{\,\widehat{\!p\!}\,\,})$,

4.45 $$\begin{aligned}&\overline{Z}\left( Y(t,t,x,{\,\widehat{\!p\!}\,\,}) \right) = \overline{Z}(t) \frac{dY}{\mathrm{d}s}(t,t,x,{\,\widehat{\!p\!}\,\,}) + (\overline{Z}Y)(t,t,x,{\,\widehat{\!p\!}\,\,}), \nonumber \\&\quad \text {where}\quad (\overline{Z}Y)(t,t,x,{\,\widehat{\!p\!}\,\,}) = \overline{Z}\left( Y(s,t,x,{\,\widehat{\!p\!}\,\,}) \right) \vert _{s=t}. \end{aligned}$$

Repeated application (4.45) inductively implies that

4.46 $$\begin{aligned} \begin{aligned}&(\overline{Z}^I Y)(t,t,x,{\,\widehat{\!p\!}\,\,}) = \overline{Z}^I \left( Y(t,t,x,{\,\widehat{\!p\!}\,\,}) \right) \\&\quad +\!\!\!\sum _{\begin{array}{c} J_1 + \dots +J_{k+1} +J = I ,\, |J_i|\ge 1, \, k\ge 0 \end{array}\!\!\!\!\!\!\!\!\!\!\! \!\!\!\!\!\!\!\!\!\!\!\!\!\!\!\!\!\!\!\!\!\!\!\!\!\!\!\!\!\!\!\!\!\!\!\!\!\!\!} C_{I,J_1,\dots ,J_{k+1},J}\,\,\, \overline{Z}^{J_1\!}(t)\cdots \overline{Z}^{J_{k+1}\!}(t) \big (\overline{Z}^{J}Y^{(k+1)}\big )(t,t,x,{\,\widehat{\!p\!}\,\,}), \end{aligned} \end{aligned}$$

where $Y^{(k)}=d^k Y/\mathrm{d}s^k$ and $\overline{Z}^{J_i}(t)=Z^{J_i}(t)$ are constants times *t* or $x^j$, for some *j*. Applying (4.46) to $Y=\overline{X}$ and $Y=\overline{P}$ noting that $\overline{X}(t,t,x,{\,\widehat{\!p\!}\,\,})=\overline{P}(t,t,x,{\,\widehat{\!p\!}\,\,})=0$ gives

4.47 $$\begin{aligned} (\overline{Z}^I \overline{P})(t,t,x,{\,\widehat{\!p\!}\,\,})&=\!\!\!\sum _{\begin{array}{c} J_1 + \dots +J_{k+1} +L = I ,\, |J_i|\ge 1, \, k\ge 0 \end{array}\!\!\!\!\!\!\!\!\!\!\! \!\!\!\!\!\!\!\!\!\!\!\!\!\!\!\!\!\!\!\!\!\!\!\!\!\!\!\!\!\!\!} C_{I,J_1,\dots ,J_{k+1},L}\,\,\, \overline{Z}^{J_1\!}(t)\cdots \overline{Z}^{J_{k+1}\!}(t) \big (\overline{Z}^{L}\overline{P}^{(k+1)}\big )(t,t,x,{\,\widehat{\!p\!}\,\,}), \end{aligned}$$

4.48 $$\begin{aligned} (\overline{Z}^I \overline{X})(t,t,x,{\,\widehat{\!p\!}\,\,})&=\!\!\!\sum _{\begin{array}{c} J_1 + \dots +J_{k+2} +L = I ,\, |J_i|\ge 1, \, k\ge 0 \end{array}\!\!\!\!\!\!\!\!\!\!\! \!\!\!\!\!\!\!\!\!\!\!\!\!\!\!\!\!\!\!\!\!\!\!\!\!\!\!\!\!\!\!} C^\prime _{I,J_1,\dots ,J_{k+2},L}\,\,\, \overline{Z}^{J_1\!}(t)\cdots \overline{Z}^{J_{k+2}\!}(t) \big (\overline{Z}^{L}\overline{P}^{(k+1)}\big )(t,t,x,{\,\widehat{\!p\!}\,\,}), \end{aligned}$$

where for the proof of (4.48) we used that $\overline{X}^{(k+1)}=\overline{P}^{(k)}$ and for $k=0$ we also used (4.47). Hence

$$\begin{aligned} |(\overline{Z}^I \overline{P})(t,t,x,{\,\widehat{\!p\!}\,\,})|&\lesssim \sum _{|L|+k\le |I|-1,\, k\ge 0\!\!\!\!\!\!\!\!\!\!\!\!\!\!\!\!\!\!} t^{k+1} \big |\big (\overline{Z}^{L}\overline{P}^{(k+1)}\big )(t,t,x,{\,\widehat{\!p\!}\,\,})\big |,\\ |(\overline{Z}^I \overline{X})(t,t,x,{\,\widehat{\!p\!}\,\,})|&\lesssim \sum _{|L|+k\le |I|-2,\, k\ge 0\!\!\!\!\!\!\!\!\!\!\!\!\!\!\!\!\!\!} t^{k+2} \big |\big (\overline{Z}^{L}\overline{P}^{(k+1)}\big )(t,t,x,{\,\widehat{\!p\!}\,\,})\big |, \end{aligned}$$

and, by (4.40), we have

#### Lemma 4.20

With $\widehat{\Gamma }^{(k)}$ and $\widehat{\Gamma }^{(k,2)}$ as in (4.38) and (4.41) we have

$$\begin{aligned} |\overline{Z}^I \overline{P}| \lesssim&\sum _{|L|+k\le |I|-1,\, k\ge 0\!\!\!\!\!\!\!\!\!\!\!\!\!} t^{k+1} \big |\overline{Z}^{L} \big (\widehat{\Gamma }^{(k)}({\,\widehat{\!X\!}\,\,}\!,{\,\widehat{\!P\!}\,\,}) -\widehat{\Gamma }^{(k)}({\,\widehat{\!X\!}\,\,}_{\!\!1},{\,\widehat{\!P\!}\,\,}_{\!\!1})\big )\big | \\&+ t^{k+1} \big |\overline{Z}^{L} \big (\widehat{\Gamma }^{(k,2)}({\,\widehat{\!X\!}\,\,}_{\!\!1},{\,\widehat{\!P\!}\,\,}_{\!\!1})\big )\big |,\quad \text {at}\quad s=t,\\ |\overline{Z}^I \overline{X}| \lesssim&\sum _{|L|+k\le |I|-2,\, k\ge 0\!\!\!\!\!\!\!\!\!\!\!\!\!} t^{k+2} \big |\overline{Z}^{L} \big (\widehat{\Gamma }^{(k)}({\,\widehat{\!X\!}\,\,}\!,{\,\widehat{\!P\!}\,\,}) -\widehat{\Gamma }^{(k)}({\,\widehat{\!X\!}\,\,}_{\!\!1},{\,\widehat{\!P\!}\,\,}_{\!\!1})\big )\big | \\&+ t^{k+2} \big |\overline{Z}^{L} \big (\widehat{\Gamma }^{(k,2)}({\,\widehat{\!X\!}\,\,}_{\!\!1},{\,\widehat{\!P\!}\,\,}_{\!\!1})\big )\big |,\quad \text {at}\quad s=t, \end{aligned}$$

where everything is evaluated at $(s,t,x,{\,\widehat{\!p\!}\,\,})$ where $s=t$.

We can now apply the estimates from the previous section.

#### Proposition 4.21

Suppose that $t\ge t_0 + 1$, $\vert x \vert \le c t$, $(t,x,{\,\widehat{\!p\!}\,\,}) \in \mathrm {supp}(f)$ and the bounds (4.1) hold. Then, for $\vert I \vert \le N/2+2$,

$$\begin{aligned} \big \vert \overline{Z}^I \big ( \overline{P}^i(s,t,x,{\,\widehat{\!p\!}\,\,}) \big ) \big \vert _{s=t} \big \vert +t^{-1}\big \vert \overline{Z}^I \big ( \overline{X}^i(s,t,x,{\,\widehat{\!p\!}\,\,}) \big ) \big \vert _{s=t} \big \vert \lesssim \varepsilon t^{-2a}. \end{aligned}$$

#### Proof

We will use induction to prove this. Assuming that we have that $|I|< m\le N/2+1$, the assumptions of Corollary 4.19 hold at $s=t$, and writing $X-X_1=\overline{X}+X_2-X_1$ and ${\,\widehat{\!P\!}\,\,}-\widehat{P}_1=\overline{P}+\widehat{P}_2-\widehat{P}_1$ and using the estimates (4.26) and (4.28) for $X_2-X_1$ and $\widehat{P}_2-\widehat{P}_1$, respectively, we get, for $\vert L \vert + k \le m -1$,

$$\begin{aligned} t^{k+1}\big |\overline{Z}^L\!\big (\widehat{\Gamma }^{(k)}(X,\widehat{P})-\widehat{\Gamma }^{(k)}(X_1,\widehat{P}_1)\big )\big |&\lesssim \frac{\varepsilon }{t^{a}}\!\!\!\!\sum _{|J|\le |L|}\!\!\!\! \Big (\frac{|\overline{Z}^J\!\! (X\!-\!X_1)|\!}{t}+\big |\overline{Z}^{J} \! (\widehat{P}\!-\!\widehat{P}_1)\big |\Big )\\&\lesssim \frac{\varepsilon }{t^{2a}\!\!}+\frac{\varepsilon }{t^{a}}\!\!\!\!\sum _{|J|\le |L|}\!\!\!\! \Big (\frac{|\overline{Z}^J \!\overline{X}|\!}{s}+\big |\overline{Z}^{J}\! \overline{P}\big |\Big )\\ t^{k+1}\big |\overline{Z}^L\big (\widehat{\Gamma }^{(k,2)}(X_1,\widehat{P}_1)\big )\big |&\lesssim \frac{\varepsilon }{t^{2a}} . \end{aligned}$$

Assuming that the proposition is true for $|I|<m$ it now follows from this and previous lemma that it is also true for $|I|=m$. $\quad \square$

#### Proposition 4.22

Suppose $t\ge t_0 + 1$, $\vert x \vert \le c t$, $(t,x,{\,\widehat{\!p\!}\,\,}) \in \mathrm {supp}(f)$ and the bounds (4.1) hold. Then, for $|I|\le N$,

$$\begin{aligned} \left| \overline{Z}^J \left( \overline{P}^i(s,t,x,{\,\widehat{\!p\!}\,\,}) \right) \vert _{s=t} \right| \lesssim&\frac{\varepsilon }{t^{2a}} + \frac{1}{t^a}\sum _{\vert J \vert \le \vert I \vert -1} \bigg ( t \left| (Z^J \Gamma ) (t,x) \right| \\&+\int _{t_0}^t \frac{s' - t_0}{t-t_0} \left| (Z^J \Gamma ) \left( s', s'\frac{x}{t} \right) \right| \, \mathrm{d}s' \bigg ),\\ \left| \overline{Z}^I \left( \overline{X}^i(s,t,x,{\,\widehat{\!p\!}\,\,}) \right) \vert _{s=t} \right| \lesssim&\varepsilon t^{1-2a} + t^{1-a}\sum _{\vert J \vert \le \vert I \vert -1} \bigg ( t \left| (Z^J \Gamma ) (t,x) \right| \\&+ \int _{t_0}^t \frac{s' - t_0}{t-t_0} \left| (Z^J \Gamma ) \left( s', s'\frac{x}{t} \right) \right| \,\mathrm{d}s' \bigg ). \end{aligned}$$

#### Proof

We will use induction to prove this. Assuming that the proposition is true for $|I|< m\le N$, the assumptions of Proposition 4.18 hold at $s=t$ and so, for $\vert L \vert + k \le m -1$,

$$\begin{aligned} t^{k+1} \big |\overline{Z}^L\!\big (\widehat{\Gamma }^{(k)\!}(X,\!\widehat{P}) -\widehat{\Gamma }^{(k)\!}(X_{\!1},\!\widehat{P}_{1})\big )\big |\!\lesssim & {} \!\frac{1}{t^{a}}\!\!\sum _{|J|\le |L| \!\!\!\!\!\!} \Big (\varepsilon \frac{|\overline{Z}^J \!\!(X\!\!-\!\!X_{\!1})|\!}{t}+ \varepsilon \big |\overline{Z}^{J} \! \!(\widehat{P}-\widehat{P}_{1})\big |\!\\&+\frac{\varepsilon }{t^{a}\!} +\!\sum _{|J|\le |L|+k\!\!\!\!\!\!\!\!\!\!\!\!\!\!\!}t\big |(Z^{J} \Gamma )(X_{\!1})\big |\Big ) \end{aligned}$$

and

$$\begin{aligned} t^{k+1} \big |\overline{Z}^L\big (\widehat{\Gamma }^{(k,2)}(X_1,P_1)\big )\big | \lesssim \frac{\varepsilon }{t^{2a}} + \frac{1}{t^a} \sum _{|M|\le |L|+k\!\!\!\!\!}t\big |(Z^{M} \Gamma )(X_1)\big |, \end{aligned}$$

since $\frac{|\overline{Z}^I \!\!X_{\!1}|\!}{t}+|\overline{Z}^I \!\!\widehat{P}_{1}| \le C$ for any multi index *I*, using the form of the vector fields $\overline{Z}$. Hence, writing $X-X_1=\overline{X}+X_2-X_1$ and ${\,\widehat{\!P\!}\,\,}-\widehat{P}_1=\overline{P}+\widehat{P}_2-\widehat{P}_1$, we get

$$\begin{aligned}&t^{k+1} \big |\overline{Z}^L\!\big (\widehat{\Gamma }^{(k)\!}(X,\!\widehat{P}) -\widehat{\Gamma }^{(k)\!}(X_{\!1},\!\widehat{P}_{\!1})\big )\big |\! +t^{k+1} \big |\overline{Z}^L\big (\widehat{\Gamma }^{(k,2)}(X_1,P_1)\big )\big |\\&\quad \lesssim \!\frac{\varepsilon }{t^{a}}\sum _{|J|\le |L| } \Bigg (\frac{|\overline{Z}^J \overline{X}|\!}{t}+\big |\overline{Z}^{J}\overline{P}\big |\Bigg )\! + \!\frac{1}{t^{a}}\!\!\sum _{|J|\le |L| } \Bigg (\varepsilon \frac{|\overline{Z}^J \!\!(X_2\!-\!\!X_{\!1})|\!}{t}+\varepsilon \big |\overline{Z}^{J} (\widehat{P}_2\!-\widehat{P}_{\!1})\big |\!\\&\qquad +\frac{\varepsilon }{t^{a}\!} +\!\sum _{|J|\le |L|+k}t\big |(Z^{J} \Gamma )(X_{\!1})\big |\Bigg ). \end{aligned}$$

Using induction for the first sum and the estimates (4.25) and (4.27) for $X_2-X_1$ and $\widehat{P}_2-\widehat{P}_1$, respectively, the proposition follows. $\quad \square$

### $L^{\infty }$ Estimates for Lower Order Derivatives of Geodesics

The estimates in the previous sections easily lead to pointwise bounds for lower order derivatives of $\overline{X}(s,t,x,p)$ and $\overline{P}(s,t,x,p)$.

#### Proposition 4.23

Suppose $t\ge t_0 + 1$, $\vert x \vert \le c t$, $(t,x,{\,\widehat{\!p\!}\,\,}) \in \mathrm {supp}(f)$ and the bounds (4.1) hold. Then, for $i=1,2,3$,

$$\begin{aligned} s^{2a - 1} \left| \overline{Z}^I \left( \overline{X}(s,t,x,p)^i \right) \right| + s^{2a} \left| \overline{Z}^I \left( \overline{P}(s,t,x,p)^i \right) \right| \le C \varepsilon \end{aligned}$$

for all $t_0 \le s \le t$, for $\vert I \vert =0,1,\ldots ,\left\lfloor \frac{N}{2} \right\rfloor + 1$.

#### Proof

The proof proceeds by induction. Clearly the result is true when $\vert I \vert =0$ by Proposition 2.3. Assume the result is true for all $\vert I \vert \le k$, for some $k \le \left\lfloor \frac{N}{2} \right\rfloor$. Then *I* clearly satisfies the assumptions of Corollary 4.16 and so, by the equations (2.14), (2.15) and the pointwise bounds (4.1),

$$\begin{aligned} \Big \vert \frac{\mathrm{d}}{\mathrm{d}s} \overline{Z}^I \left( \overline{P}^i(s) \right) \Big \vert= & {} \left| \overline{Z}^I \left( {\,\widehat{\!\Gamma \!}\,\,}^i \left( s, X(s), {\,\widehat{\!P\!}\,\,}(s) \right) - {\,\widehat{\!\Gamma \!}\,\,}^i \left( s, s\frac{x}{t}, \frac{x}{t} \right) \right) \right| \\\lesssim & {} \frac{\varepsilon }{s^{1+a}} \sum _{\vert J \vert \le \vert I \vert } \Big (\frac{|\overline{Z}^J\! (X\!-\!X_1)|\!}{s}+\big |\overline{Z}^{J} \! (\widehat{P}\!-\!\widehat{P}_1)\big |\Big ), \end{aligned}$$

where we recall $X_1(s,t,x) = s \frac{x}{t}$ and $\widehat{P}_1(t,x) = \frac{x}{t}$. Writing $X-X_1=\overline{X}+X_2-X_1$ and ${\,\widehat{\!P\!}\,\,}-\widehat{P}_1=\overline{P}+\widehat{P}_2-\widehat{P}_1$ and using the estimates (4.26) and (4.28) for $X_2-X_1$ and $\widehat{P}_2-\widehat{P}_1$, respectively, gives

$$\begin{aligned} \Big \vert \frac{\mathrm{d}}{\mathrm{d}s} \overline{Z}^I \left( \overline{P}^i(s) \right) \Big \vert \lesssim \frac{\varepsilon }{s^{1+2a}} + \frac{\varepsilon }{s^{1+a}} \sum _{\vert J \vert \le \vert I \vert } \Big ( \frac{|\overline{Z}^J\! (\overline{X}(s))|\!}{s}+\big |\overline{Z}^{J} \! (\overline{P}(s))\big | \Big ). \end{aligned}$$

Integrating backwards from $s=t$ and using Proposition 4.21,

$$\begin{aligned} \left| \overline{Z}^I \left( \overline{P}^i(s) \right) \right| \lesssim \frac{\varepsilon }{s^{2a}} + \varepsilon \sum _{\vert J \vert \le \vert I \vert } \int _s^t \Big ( \frac{|\overline{Z}^J\! (\overline{X}(\tilde{s}))|\!}{\tilde{s}^{2+a}} + \frac{\big |\overline{Z}^{J} \! (\overline{P}(\tilde{s}))\big |}{\tilde{s}^{1+a}} \Big )\, \mathrm{d} \tilde{s}, \end{aligned}$$

and so, after summing over $i=1,2,3$ and *I*, the Grönwall inequality and Lemma 4.25, give that

$$\begin{aligned} \left| \overline{Z}^I \left( \overline{P}(s) \right) \right| \lesssim \frac{\varepsilon }{s^{2a}} + \varepsilon \sum _{\vert J \vert \le \vert I \vert } \int _s^t \frac{|\overline{Z}^J\! (\overline{X}(\tilde{s}))|\!}{\tilde{s}^{2+a}} \,\mathrm{d} \tilde{s}. \end{aligned}$$

The equation (2.14) and Proposition 4.21 then give, after integrating backwards from $s=t$ again,

$$\begin{aligned} \left| \overline{Z}^I \left( \overline{X}^i(s) \right) \right| \lesssim \varepsilon s^{1-2a} + \varepsilon \sum _{\vert J \vert \le \vert I \vert } \int _s^t \frac{|\overline{Z}^J\! (\overline{X}(\tilde{s}))|\!}{\tilde{s}^{1+a}} \,\mathrm{d}\tilde{s}, \end{aligned}$$

where the fact that, for any function $\lambda (s)$,

$$\begin{aligned} \int _s^t \int _{s'}^t \lambda (\tilde{s}) \,\mathrm{d}\tilde{s} \mathrm{d}s' = \int _s^t \int _s^t \chi _{\{ s'\le \tilde{s}\}} \,\mathrm{d}s' \lambda (\tilde{s}) \,\mathrm{d}\tilde{s} = \int _s^t (\tilde{s} - s) \lambda (\tilde{s}) \,\mathrm{d}\tilde{s}, \end{aligned}$$

has been used (here $\chi _{\{ s'\le \tilde{s}\}}$ is the indicator function of the interval $[s',\tilde{s}]$). Another application of the Grönwall inequality, after summing over $i=1,2,3$ and *I*, completes the proof. $\quad \square$

Corollary 4.10 and Proposition 4.23 immediately yield the following sharp pointwise bounds:

#### Proposition 4.24

Suppose $t\ge t_0 + 1$, $\vert x \vert \le c t$, $(t,x,{\,\widehat{\!p\!}\,\,}) \in \mathrm {supp}(f)$ and the bounds (4.1) hold. Then, for $i=1,2,3$,

$$\begin{aligned} s^{-1}\big \vert \overline{Z}^I \big ( X(s,t,x,p)^i \big ) \big \vert + \big \vert \overline{Z}^I \big ( {\,\widehat{\!P\!}\,\,}(s,t,x,p)^i \big ) \big \vert \le C \end{aligned}$$

for all $t_0\le s \le t$, for $\vert I \vert =0,1,2,\ldots ,\left\lfloor \frac{N}{2} \right\rfloor + 1$.

The following form of the Grönwall inequality was used in the proof of Proposition 4.23 above, and will be used in the proof of Proposition 4.26 below:

#### Lemma 4.25

For continuous functions $v,a,b: [t_0 ,t] \rightarrow \mathbb {R}$, if

$$\begin{aligned} v(s) \le \int _s^t a(s') v(s') \,\mathrm{d}s' + b(s) \end{aligned}$$

for $$s\in [t_0,t]$$, then

$$\begin{aligned} v(s) \le b(s) + \int _s^t a(s') b(s') e^{\int _s^{s'} a(s'') \,\mathrm{d}s''} \,\mathrm{d}s'. \end{aligned}$$

### Higher Order Estimates for Derivatives of Geodesics

The main result of this section is

#### Proposition 4.26

Suppose that $t\ge t_0 + 1$, $\vert x \vert \le c t$, $(t,x,{\,\widehat{\!p\!}\,\,}) \in \mathrm {supp}(f)$ and the bounds (4.1) hold. Then, for $i=1,2,3$,

4.49 $$\begin{aligned} \left| \overline{Z}^I \left( \overline{X}(s,t,x,{\,\widehat{\!p\!}\,\,})^i \right) \right| \lesssim&\, \varepsilon s^{1-2a} + t^{2-a} \sum _{\vert J \vert \le |I|-1} \left| (Z^J \Gamma )(t,x) \right| \nonumber \\&+ \sum _{\vert J \vert \le |I| } \int _{t_0}^t (s')^{1-a}\left| (Z^J \Gamma ) \left( s', s' \frac{x}{t} \right) \right| \, \mathrm{d}s' \nonumber \\&+ \sum _{\vert J \vert \le \vert I \vert } \int _s^t s' \left| (Z^J \Gamma ) \left( s', s'\frac{x}{t} \right) - (Z^J \Gamma ) \left( s', X(s') \right) \right| \,\mathrm{d}s' \end{aligned}$$

and

4.50 $$\begin{aligned} \left| \overline{Z}^I \left( \overline{P}(s,t,x,{\,\widehat{\!p\!}\,\,})^i \right) \right| \lesssim&\, \varepsilon s^{-2a} + \frac{t}{s^{a}} \sum _{\vert J \vert \le |I|-1} \left| (Z^J \Gamma )(t,x) \right| \nonumber \\&+\sum _{\vert J \vert \le |I| } \int _{t_0}^t (s')^{-a}\left| (Z^J \Gamma ) \left( s', s' \frac{x}{t} \right) \right| \, \mathrm{d}s'\nonumber \\&+ \sum _{\vert J \vert \le \vert I \vert } \int _s^t \left| (Z^J \Gamma ) \big ( s', s'\frac{x}{t} \big ) - (Z^J \Gamma ) \left( s', X(s') \right) \right| \, \mathrm{d}s' \end{aligned}$$

for all $$t_0 \le s \le t$$, $$\vert I \vert \le N$$.

#### Proof

Let *I* be a multi index with $\vert I \vert \le N$. Using the equation (2.15) and Proposition 4.15,

$$\begin{aligned} \Big \vert \frac{\mathrm{d} \overline{Z}^I \big ( \overline{P}^i(s) \big )}{\mathrm{d}s} \Big \vert \lesssim&\sum _{\vert K \vert \le \left\lfloor \frac{\vert I \vert }{2} \right\rfloor +1} \Vert (Z^K\Gamma )(s,\cdot ) \Vert _{L^{\infty }} \sum _{\vert J \vert \le \vert I \vert } \Big ( \frac{\vert \overline{Z}^J(X-X_1) \vert }{s} + \vert \overline{Z}^J({\,\widehat{\!P\!}\,\,}-{\,\widehat{\!P\!}\,\,}_{\!\!1}) \vert \Big )\\&+ \sum _{\vert J \vert \le \vert I \vert } \left| (Z^J\Gamma )\big (s,s \frac{x}{t} \big ) \right| \sum _{\vert K \vert \le \left\lfloor \frac{\vert I \vert }{2} \right\rfloor +1} \Big ( \frac{\vert \overline{Z}^K(X-X_1) \vert }{s} + \vert \overline{Z}^K({\,\widehat{\!P\!}\,\,}-{\,\widehat{\!P\!}\,\,}_{\!\!1}) \vert \Big )\\&+ \sum _{\vert J \vert \le \vert I \vert } \Big \vert (Z^J \Gamma ) \big ( s, s\frac{x}{t} \big ) - (Z^J \Gamma ) \left( s, X(s) \right) \Big \vert . \end{aligned}$$

Writing

$$\begin{aligned} \frac{X(s)-X_1(s)}{s} = \frac{\overline{X}(s)}{s} + \frac{X_2(s)}{s} - \frac{x}{t}, \quad {\,\widehat{\!P\!}\,\,}(s) - {\,\widehat{\!P\!}\,\,}_{\!\!1}(s) = \overline{P}(s) + {\,\widehat{\!P\!}\,\,}_2(s) - \frac{x}{t}, \end{aligned}$$

and using Corollary 4.10, Proposition 4.23 and the pointwise bounds (4.1) for $\Gamma$ gives

$$\begin{aligned} \Big \vert \frac{\mathrm{d} \overline{Z}^I \big ( \overline{P}^i(s) \big )}{\mathrm{d}s} \Big \vert \lesssim \varepsilon \sum _{\vert J \vert \le \vert I \vert } \Big ( \frac{\vert \overline{Z}^J\left( \overline{X}(s)\right) \vert }{s^{2+a}} + \frac{\vert \overline{Z}^J\left( \overline{P}(s)\right) \vert }{s^{1+a}} \Big ) + F_{\vert I \vert }(s,t,x,{\,\widehat{\!p\!}\,\,}), \end{aligned}$$

where

$$\begin{aligned} F_{\vert I \vert }(s,t,x,{\,\widehat{\!p\!}\,\,}) =&\, \frac{\varepsilon }{s^{1+2a}} + \frac{t}{s^{1+a}} \sum _{\vert J \vert \le |I|-1} \left| (Z^J \Gamma )(t,x) \right| + \frac{1}{s^a} \sum _{\vert J \vert \le \vert I \vert } \left| (Z^J\Gamma )\left( s,s \frac{x}{t} \right) \right| \\&+ \frac{1}{s^{1+a}} \sum _{\vert J \vert \le \vert I \vert } \int _{t_0}^t \left| (Z^J\Gamma )\left( s',s' \frac{x}{t} \right) \right| \frac{s'}{s'+s} \,\mathrm{d}s' \\&+ \sum _{\vert J \vert \le \vert I \vert } \left| (Z^J \Gamma ) \left( s, s\frac{x}{t} \right) - (Z^J \Gamma ) \left( s, X(s) \right) \right| . \end{aligned}$$

Integrating backwards from $s=t$ gives

$$\begin{aligned} \left| \overline{Z}^I \big ( \overline{P}^i(s) \big ) \right| \lesssim&\left| \overline{Z}^I \big ( \overline{P}^i(s) \big ) \vert _{s=t} \right| + \int _s^t \varepsilon \sum _{\vert J \vert \le \vert I \vert } \Big ( \frac{\vert \overline{Z}^J\left( \overline{X}(\tilde{s})\right) \vert }{\tilde{s}^{2+a}} + \frac{\vert \overline{Z}^J\left( \overline{P}(\tilde{s})\right) \vert }{\tilde{s}^{1+a}} \Big ) \\&+ F_{\vert I \vert }(\tilde{s},t,x,{\,\widehat{\!p\!}\,\,}) \,\mathrm{d}\tilde{s}. \end{aligned}$$

Summing over $i=1,2,3$ and *I*, the Grönwall inequality, Lemma 4.25, gives

$$\begin{aligned} \left| \overline{Z}^I \big ( \overline{P}^i(s) \big ) \right| \lesssim \left| \overline{Z}^I \big ( \overline{P}^i(s) \big ) \vert _{s=t} \right| + \int _s^t \varepsilon \sum _{\vert J \vert \le \vert I \vert } \frac{\vert \overline{Z}^J\left( \overline{X}(\tilde{s})\right) \vert }{\tilde{s}^{2+a}} + F_{\vert I \vert }(\tilde{s},t,x,{\,\widehat{\!p\!}\,\,})\,\mathrm{d}\tilde{s}. \end{aligned}$$

Integrating backwards from $s=t$ again, the equation (2.14) implies

$$\begin{aligned} \left| \overline{Z}^I\big ( \overline{X}^i(s) \big ) \right| \lesssim&\left| \overline{Z}^I \big ( \overline{X}^i(s) \big ) \vert _{s=t} \right| + (t-s) \left| \overline{Z}^I \big ( \overline{P}^i(s) \big ) \vert _{s=t} \right| \\&+ \int _s^t \varepsilon \sum _{\vert J \vert \le \vert I \vert } \frac{\vert \overline{Z}^J\left( \overline{X}(\tilde{s})\right) \vert }{\tilde{s}^{1+a}} + \tilde{s} F_{\vert I \vert }(\tilde{s},t,x,{\,\widehat{\!p\!}\,\,})\,\mathrm{d}\tilde{s}, \end{aligned}$$

where the fact that, for any function $\lambda (s)$,

$$\begin{aligned} \int _s^t \int _{\tilde{s}}^t \lambda (s') \,\mathrm{d}s'\mathrm{d}\tilde{s} = \int _s^t \int _s^t \chi _{\{ \tilde{s} \le s' \}}\,\mathrm{d}\tilde{s} \lambda (s') \mathrm{d}s' = \int _s^t (s'-s) \lambda (s')\, \mathrm{d}s', \end{aligned}$$

has been used. Another application of the Grönwall inequality 4.25 gives

$$\begin{aligned} \left| \overline{Z}^I \big ( \overline{X}^i(s) \big ) \right| \lesssim \left| \overline{Z}^I \big ( \overline{X}^i(s) \big ) \vert _{s=t} \right| + (t-s) \left| \overline{Z}^I \big ( \overline{P}^i(s) \big ) \vert _{s=t} \right| + \int _s^t \tilde{s} F_{\vert I \vert }(\tilde{s},t,x,{\,\widehat{\!p\!}\,\,})\,\mathrm{d}\tilde{s}. \end{aligned}$$

The bound (4.49) follows from Proposition 4.22, along with the fact that

$$\begin{aligned}&\int _s^t \frac{1}{\tilde{s}^{a}} \int _{t_0}^t \left| (Z^J\Gamma )\left( s',s' \frac{x}{t} \right) \right| \frac{s'}{s'+\tilde{s}} \mathrm{d}s' \,\mathrm{d}\tilde{s}\\&\quad \lesssim \int _s^t \frac{1}{\tilde{s}^{a}} \int _{\tilde{s}}^t \left| (Z^J\Gamma )\left( s',s' \frac{x}{t} \right) \right| \mathrm{d}s' + \frac{1}{\tilde{s}^{1+a}} \int _{t_0}^{\tilde{s}} s' \left| (Z^J\Gamma )\left( s',s' \frac{x}{t} \right) \right| \, \mathrm{d}s' \,\mathrm{d}\tilde{s}, \end{aligned}$$

and, for any nonnegative function $\lambda (s)$,

$$\begin{aligned}&\int _s^t \frac{1}{\tilde{s}^{1+a}} \int _{t_0}^{\tilde{s}} s' \lambda (s') \,\mathrm{d}s' \mathrm{d}\tilde{s}\\&\quad \lesssim \int _{t_0}^t \int _{t_0}^t \frac{1}{\tilde{s}^{1+a}} \chi _{\{ s'\le \tilde{s} \}}\,\mathrm{d}\tilde{s} s' \lambda (s') \mathrm{d}s' \lesssim \int _{t_0}^t (s')^{1-a} \lambda (s')\, \mathrm{d}s', \end{aligned}$$

and

$$\begin{aligned} \int _s^t \frac{1}{\tilde{s}^{a}} \int _{\tilde{s}}^t \lambda (s') \,\mathrm{d}s' \,\mathrm{d}\tilde{s} \lesssim \int _{s}^t \int _{s}^t \frac{1}{\tilde{s}^{a}} \chi _{\{ \tilde{s} \le s' \}}\,\mathrm{d}\tilde{s} \lambda (s') \,\mathrm{d}s' \lesssim \int _{s}^t (s')^{1-a} \lambda (s')\, \mathrm{d}s'. \end{aligned}$$

The bound (4.50) follows similarly. $\quad \square$

#### Corollary 4.27

Suppose $t\ge t_0 + 1$, $\vert x \vert \le c t$, $(t,x,{\,\widehat{\!p\!}\,\,}) \in \mathrm {supp}(f)$ and the bounds (4.1) hold. Then, for $i=1,2,3$,

$$\begin{aligned}&\left| \overline{Z}^I \left( X(t_0,t,x,{\,\widehat{\!p\!}\,\,})^i \right) \right| + \left| \overline{Z}^I \left( P(t_0,t,x,{\,\widehat{\!p\!}\,\,})^i \right) \right| \\&\quad \le C \bigg ( 1 + \sum _{\vert J \vert \le \vert I \vert -1} t^{2-a} \vert (Z^J \Gamma )(t,x) \vert \\&\qquad + \sum _{\vert J \vert \le \vert I \vert } \int _{t_0}^t (s')^{1-a} \left| (Z^J \Gamma ) \left( s', s'\frac{x}{t} \right) \right| \\&\qquad + s' \left| (Z^J \Gamma ) \left( s', s'\frac{x}{t} \right) - (Z^J \Gamma ) \left( s', X(s') \right) \right| \,\mathrm{d}s' \bigg ). \end{aligned}$$

#### Proof

The corollary is an immediate consequence of Propositions 4.9 and 4.26.

$$\square $$

### Spacetime Derivatives and Small Time

Since the vector fields $\overline{Z}$ become singular at time $t=t_0$, in this section the spacetime $\partial _t$ and $\partial _{x^i}$ derivatives of $X(s,t,x,{\,\widehat{\!p\!}\,\,})$ and ${\,\widehat{\!P\!}\,\,}(s,t,x,{\,\widehat{\!p\!}\,\,})$ are estimated for $t_0 \le t \le t_0 +1$. Since the results of this section are local in time they are much simpler than those in previous sections. In particular, it is not necessary to subtract the approximations $X_2$, ${\,\widehat{\!P\!}\,\,}_2$ from *X* and ${\,\widehat{\!P\!}\,\,}$ respectively. Note that $\partial$ always denotes the spacetime gradient $\partial = (\partial _t,\partial _{x^1}, \partial _{x^2}, \partial _{x^3})$. When applied to functions on $\mathcal {P}$ the derivatives are, as usual, taken with respect to the $(t,x,{\,\widehat{\!p\!}\,\,})$ coordinate system.

It is first necessary to estimate derivatives of the equations (1.17).

#### Proposition 4.28

Let *I* be a multi index and suppose $\vert \partial ^K X \vert + \vert \partial ^K {\,\widehat{\!P\!}\,\,}\vert \le C$ for all $\vert K \vert \le \frac{\vert I \vert }{2}$. Then,

$$\begin{aligned} \left| \partial ^I \left( {\,\widehat{\!\Gamma \!}\,\,}\left( s, X(s), {\,\widehat{\!P\!}\,\,}(s) \right) \right) \right|\lesssim & {} \sum _{\vert K \vert \le \left\lfloor \frac{\vert I \vert }{2} \right\rfloor + 1} \!\!\!\!\!\Vert \partial ^K \Gamma (s,\cdot ) \Vert _{L^{\infty }} \sum _{\vert J \vert \le \vert I \vert } \left( \vert \partial ^J X(s) \vert + \vert \partial ^J {\,\widehat{\!P\!}\,\,}(s) \vert \right) \\&+ \sum _{\vert J \vert \le \vert I \vert } \left| \partial ^J \Gamma (s,X(s)) \right| . \end{aligned}$$

#### Proof

Recall that ${\,\widehat{\!\Gamma \!}\,\,}(X,{\,\widehat{\!P\!}\,\,}) = \Gamma (X) \cdot \Lambda ({\,\widehat{\!P\!}\,\,})$ (the *s* dependence is omitted for brevity). There exist constants $c_{J_1\ldots J_k I_K}$, $\widetilde{c}_{J_1\ldots J_k I_K}$ such that

$$\begin{aligned} \partial ^I (\Gamma (X)) = \sum _{k=1}^{\vert I \vert } \sum _{\begin{array}{c} \vert J_1 \vert + \ldots + \vert J_k \vert \le \vert I \vert \\ \vert I_k \vert = k \end{array}} c_{J_1\ldots J_k I_K} \partial ^{J_1} X \ldots \partial ^{J_k} X \cdot (\partial ^{I_k} \Gamma )(X), \end{aligned}$$

and

$$\begin{aligned} \partial ^I (\Lambda ({\,\widehat{\!P\!}\,\,}))= \sum _{k=1}^{\vert I \vert } \sum _{\begin{array}{c} \vert J_1 \vert + \cdots + \vert J_k \vert \le \vert I \vert \\ \vert I_k \vert = k \end{array}} \widetilde{c}_{J_1\ldots J_k I_K} \partial ^{J_1} {\,\widehat{\!P\!}\,\,}\ldots \partial ^{J_k} {\,\widehat{\!P\!}\,\,}\cdot (\partial ^{I_k} \Lambda )({\,\widehat{\!P\!}\,\,}), \end{aligned}$$

and so, by the assumed lower order bounds for *X* and ${\,\widehat{\!P\!}\,\,}$,

$$\begin{aligned} \left| \partial ^I (\Gamma (X)) \right|\lesssim & {} \sum _{\vert K \vert \le \left\lfloor \frac{\vert I \vert }{2} \right\rfloor + 1} \!\!\!\!\!\Vert \partial ^K \Gamma (s,\cdot ) \Vert _{L^{\infty }} \sum _{\vert J \vert \le \vert I \vert } \vert \partial ^J X(s) \vert \\&+ \sum _{\vert J \vert \le \vert I \vert } \left| \partial ^J \Gamma (s,X(s)) \right| , \quad \left| \partial ^I (\Lambda ({\,\widehat{\!P\!}\,\,})) \right| \lesssim \sum _{\vert J \vert \le \vert I \vert } \vert \partial ^J {\,\widehat{\!P\!}\,\,}(s) \vert , \end{aligned}$$

from which the result follows. $\quad \square$

In order to use the system (1.17) to estimate $\partial ^I X(s,t,x,{\,\widehat{\!p\!}\,\,})$ and $\partial ^I {\,\widehat{\!P\!}\,\,}(s,t,x,{\,\widehat{\!p\!}\,\,})$, it is also necessary to estimate the final conditions (note that this is completely straightforward unless $\partial ^I$ contains $\partial _t$ derivatives).

#### Proposition 4.29

Let *I* be a multi index with $\vert I \vert \ge 1$ and suppose $\vert \partial ^J \Gamma (t,x) \vert \le C$ for all $\vert J \vert \le \left\lfloor \frac{\vert I \vert }{2} \right\rfloor + 1$. Then

$$\begin{aligned} \left| (\partial ^I {\,\widehat{\!P\!}\,\,})(t,t,x,{\,\widehat{\!p\!}\,\,}) \right|\lesssim & {} \sum _{\vert J \vert \le \vert I \vert - 1} \left| (\partial ^J \Gamma )(t,x) \right| , \quad \left| (\partial ^I X)(t,t,x,{\,\widehat{\!p\!}\,\,}) \right| \lesssim 1 \\&+ \sum _{\vert J \vert \le \vert I \vert - 1} \left| (\partial ^J \Gamma )(t,x) \right| . \end{aligned}$$

#### Proof

Recall the notation ${\,\widehat{\!P\!}\,\,}^{(k)}$ and ${\,\widehat{\!\Gamma \!}\,\,}^{(k)}$ from Section 4.6. By the formula (4.46) it follows that

$$\begin{aligned} (\partial ^I {\,\widehat{\!P\!}\,\,})(t,t,x,{\,\widehat{\!p\!}\,\,})&= \sum _{\begin{array}{c} J_1+ \cdots + J_{k+1} +L=I\\ \vert J_i \vert \ge 1, k \ge 0 \end{array}} C_{IJ_1\ldots J_{k+1},L} \partial ^{J_1} t \ldots \partial ^{J_{k+1}}t \Big ( \partial ^L {\,\widehat{\!P\!}\,\,}^{(k+1)} \Big ) (t,t,x,{\,\widehat{\!p\!}\,\,}),\\ (\partial ^I X)(t,t,x,{\,\widehat{\!p\!}\,\,})&= \partial ^I x + \sum _{\begin{array}{c} J_1+ \cdots + J_{k+2} +L=I\\ \vert J_i \vert \ge 1, k \ge 0 \end{array}} C_{IJ_1\ldots J_{k+2},L}' \partial ^{J_1} t \ldots \partial ^{J_{k+2}}t \Big ( \partial ^L {\,\widehat{\!P\!}\,\,}^{(k+2)} \Big ) (t,t,x,{\,\widehat{\!p\!}\,\,}) \end{aligned}$$

for some constants $C_{IJ_1\ldots J_{k+1},L}, C_{IJ_1\ldots J_{k+2},L}'$, where the proof of the second uses the first and the fact that $\frac{\mathrm{d} X^{(k)}}{\mathrm{d}s} = {\,\widehat{\!P\!}\,\,}^{(k)}$. Hence,

$$\begin{aligned} \left| (\partial ^I {\,\widehat{\!P\!}\,\,})(t,t,x,{\,\widehat{\!p\!}\,\,}) \right|&\lesssim \sum _{\begin{array}{c} \vert L\vert + k \le \vert I \vert - 1,\,\,\, k \ge 0 \end{array}} \Big \vert \Big ( \partial ^L {\,\widehat{\!P\!}\,\,}^{(k+1)} \Big ) (t,t,x,{\,\widehat{\!p\!}\,\,}) \Big \vert ,\\ \left| (\partial ^I X)(t,t,x,{\,\widehat{\!p\!}\,\,}) \right|&\lesssim 1 + \sum _{\begin{array}{c} \vert L\vert + k \le \vert I \vert - 2,\,\,\, k \ge 0 \end{array}} \Big \vert \Big ( \partial ^L {\,\widehat{\!P\!}\,\,}^{(k+1)} \Big ) (t,t,x,{\,\widehat{\!p\!}\,\,}) \Big \vert . \end{aligned}$$

The proof follows by noting that

$$\begin{aligned}&\Big \vert \Big ( \partial ^L {\,\widehat{\!P\!}\,\,}^{(k+1)} \Big ) (t,t,x,{\,\widehat{\!p\!}\,\,}) \Big \vert \\&\quad = \Big \vert \partial ^L \Big ( {\,\widehat{\!\Gamma \!}\,\,}^{(k)}(s,X(s),{\,\widehat{\!P\!}\,\,}(s)) \Big ) \Big \vert _{s=t} \Big \vert \lesssim \sum _{\vert I \vert \le \vert L \vert +k} \left| (\partial ^I \Gamma )(t,x) \right| , \end{aligned}$$

by an appropriate version of Lemma 4.17. $\quad \square$

#### Proposition 4.30

Suppose $t_0 \le t \le t_0+1$, and $\vert \partial ^J \Gamma (t',x)\vert \le C$ for all $t_0 \le t'\le t$ and $\vert x \vert \le ct'$ and $\vert J \vert \le \left\lfloor \frac{N}{2} \right\rfloor + 2$. Then, for $\vert I \vert \le \left\lfloor \frac{N}{2} \right\rfloor + 2$,

$$\begin{aligned} \left| \partial ^I X(s,t,x,{\,\widehat{\!p\!}\,\,}) \right| + \left| \partial ^I {\,\widehat{\!P\!}\,\,}(s,t,x,{\,\widehat{\!p\!}\,\,}) \right| \le C \end{aligned}$$

for all $$t_0 \le s \le t$$.

#### Proof

Proposition 4.28 and the equation (1.17) imply

$$\begin{aligned} \Big \vert \frac{\mathrm{d} \partial ^I {\,\widehat{\!P\!}\,\,}^i}{\mathrm{d}s}(s) \Big \vert \lesssim 1 + \sum _{\vert J \vert \le \vert I \vert } \left( \left| \partial ^J X(s) \right| + \left| \partial ^J {\,\widehat{\!P\!}\,\,}(s) \right| \right) . \end{aligned}$$

Integrating backwards from $s=t$, by Proposition 4.29,

$$\begin{aligned} \left| \partial ^I {\,\widehat{\!P\!}\,\,}^i(s) \right| \lesssim 1 + \sum _{\vert J \vert \le \vert I \vert } \int _s^t \left( \left| \partial ^J X(s') \right| + \left| \partial ^J {\,\widehat{\!P\!}\,\,}(s') \right| \right) \,\mathrm{d}s'. \end{aligned}$$

Summing over *I*, the Grönwall inequality, Lemma 4.25, gives

$$\begin{aligned} \left| \partial ^I {\,\widehat{\!P\!}\,\,}^i(s) \right| \lesssim 1 + \sum _{\vert J \vert \le \vert I \vert } \int _s^t \left| \partial ^J X(s') \right| \,\mathrm{d}s'. \end{aligned}$$

The result follows by integrating from $s=t$ again and repeating. $\quad \square$

#### Proposition 4.31

Suppose $t_0 \le t \le t_0 +1$ and $\vert \partial ^J \Gamma (t',x)\vert \le C$ for all $t_0 \le t'\le t$ and $\vert x \vert \le ct'$ and $\vert J \vert \le \left\lfloor \frac{N}{2} \right\rfloor + 2$. Then, for $\vert I \vert \le N$,

$$\begin{aligned} \left| \partial ^I X(s,t,x,{\,\widehat{\!p\!}\,\,}) \right| + \left| \partial ^I {\,\widehat{\!P\!}\,\,}(s,t,x,{\,\widehat{\!p\!}\,\,}) \right|\lesssim & {} 1 + \sum _{\vert J\vert \le \vert I \vert -1} \left| \partial ^J \Gamma (t,x) \right| \\&+ \sum _{\vert J\vert \le \vert I \vert } \int _s^t \left| \partial ^J \Gamma (s',X(s')) \right| \, \mathrm{d}s' \end{aligned}$$

for all $$t_0 \le s \le t$$.

#### Proof

Proposition 4.28, the equation (1.17) and Proposition 4.30 now imply

$$\begin{aligned} \Big \vert \frac{\mathrm{d} \partial ^I {\,\widehat{\!P\!}\,\,}^i}{\mathrm{d}s}(s) \Big \vert \lesssim 1 + \sum _{\vert J \vert \le \vert I \vert } \left( \left| \partial ^J X(s) \right| + \left| \partial ^J {\,\widehat{\!P\!}\,\,}(s) \right| + \left| \partial ^J \Gamma (s,X(s)) \right| \right) , \end{aligned}$$

and so Proposition 4.29 implies

$$\begin{aligned} \left| \partial ^I {\,\widehat{\!P\!}\,\,}(s) \right|\lesssim & {} 1 + \sum _{\vert J \vert \le \vert I \vert -1} \left| \partial ^J \Gamma (t,x) \right| \\&+ \sum _{\vert J \vert \le \vert I \vert } \int _s^t \left( \left| \partial ^J X(s') \right| + \left| \partial ^J {\,\widehat{\!P\!}\,\,}(s') \right| + \left| \partial ^J \Gamma (s',X(s')) \right| \right) \, \mathrm{d}s'. \end{aligned}$$

The proof then proceeds exactly as in Proposition 4.30. $\quad \square$

## Estimates for Components of the Energy Momentum Tensor

In this section a proof of Theorem 1.3 is given. Recall the discussion in Section 2.2. In order to use the results of Section 4 it will again be assumed throughout most of this section that $t\ge t_0+1$, the bounds (4.1) hold, and $\pi \left( \mathrm {supp}(f) \right) \subset \{ \vert x \vert \le c t \}$, where $\pi : \mathcal {P}\rightarrow \mathcal {M}$ is the natural projection. It is shown how Theorem 1.3 then follows in Section 5.4.

### Derivatives of Components of the Energy Momentum Tensor in Terms of Derivatives of *f*

Recall

$$\begin{aligned} T^{\mu \nu }(t,x) = \int f(t,x,{\,\widehat{\!p\!}\,\,}) p^{\mu } p^{\nu } \frac{\sqrt{-\det g}}{p^0}\,\mathrm{d}p^1 \mathrm{d}p^2 \mathrm{d}p^3. \end{aligned}$$

The main result of this section is Proposition 5.4, which uses the bounds on $\overline{Z}^I X$ and $\overline{Z}^I {\,\widehat{\!P\!}\,\,}$ of Corollary 4.27 to give bounds on $Z^I T^{\mu \nu }$. In order to prove the bounds for $Z^I T^{\mu \nu }$, it is convenient to first rewrite the above integral in terms of the ${\,\widehat{\!p\!}\,\,}^i$ variables.

#### Proposition 5.1

There exists a non-zero function $\Lambda$, smooth provided $\vert {\,\widehat{\!p\!}\,\,}^i \vert \le c <1$ for $i=1,2,3$, such that

$$\begin{aligned} \det \left( \frac{\partial p^i}{\partial {\,\widehat{\!p\!}\,\,}^j} \right) (t,x,{\,\widehat{\!p\!}\,\,}) = \Lambda ({\,\widehat{\!p\!}\,\,}, h(t,x)). \end{aligned}$$

#### Proof

Define ${\,\widehat{\!p\!}\,\,}^0 = 1$ and note that, since,

$$\begin{aligned} g_{\alpha \beta } p^{\alpha } p^{\beta } = -1, \end{aligned}$$

it follows that

$$\begin{aligned} 2 g_{\alpha \beta } p^{\alpha } \frac{\partial p^{\beta }}{\partial p^j} = 0, \end{aligned}$$

and hence,

$$\begin{aligned} \frac{\partial p^0}{\partial p^j} = - \frac{g_{\alpha j} p^{\alpha }}{g_{\beta 0} p^{\beta }} = - \frac{g_{\alpha j} {\,\widehat{\!p\!}\,\,}^{\alpha }}{g_{\beta 0} {\,\widehat{\!p\!}\,\,}^{\beta }}. \end{aligned}$$

Now, since $${\,\widehat{\!p\!}\,\,}^i = \frac{p^i}{p^0}$$,

$$\begin{aligned} \frac{\partial {\,\widehat{\!p\!}\,\,}^i}{\partial p^j} = \frac{1}{p^0} \left( \delta ^i_j - {\,\widehat{\!p\!}\,\,}^i \frac{\partial p^0}{\partial p^j} \right) = \frac{1}{p^0} \Big ( \delta ^i_j + {\,\widehat{\!p\!}\,\,}^i \frac{g_{\alpha j} {\,\widehat{\!p\!}\,\,}^{\alpha }}{g_{\beta 0} {\,\widehat{\!p\!}\,\,}^{\beta }} \Big ). \end{aligned}$$

The proof follows by writing $g_{\alpha \beta } = m_{\alpha \beta } + h_{\alpha \beta }$, noting that

$$\begin{aligned} g_{\alpha \beta } p^{\alpha } p^{\beta } = -1 \Rightarrow g_{\alpha \beta } {\,\widehat{\!p\!}\,\,}^{\alpha } {\,\widehat{\!p\!}\,\,}^{\beta } = - \frac{1}{(p^0)^2} \Rightarrow p^0 = \sqrt{- \frac{1}{g_{\alpha \beta } {\,\widehat{\!p\!}\,\,}^{\alpha } {\,\widehat{\!p\!}\,\,}^{\beta }}}, \end{aligned}$$

and using the fact that $\det (A^{-1}) = ( \det A)^{-1}$ for any matrix *A*. $\quad \square$

In Minkowski space, that is when $h=0$, it is straightforward to compute

$$\begin{aligned} \det \left( \frac{\partial p^i}{\partial {\,\widehat{\!p\!}\,\,}_M^j} \right) = (p^0_M)^5 = \left( 1+ (p^1)^2 + (p^2)^2 + (p^3)^2 \right) ^{\frac{5}{2}}, \end{aligned}$$

where ${\,\widehat{\!p\!}\,\,}_M^j = \frac{p^j}{p^0_M}$, and $p^0_M$ is defined by the relation $m_{\alpha \beta } p^{\alpha } p^{\beta } = -1$. It then follows that

$$\begin{aligned}&\Big \vert \det \left( \frac{\partial p^i}{\partial {\,\widehat{\!p\!}\,\,}^j} \right) - \left( 1+ (p^1)^2 + (p^2)^2 + (p^3)^2 \right) ^{\frac{5}{2}} \Big \vert = \Big \vert \det \left( \frac{\partial p^i}{\partial {\,\widehat{\!p\!}\,\,}^j} \right) - \det \left( \frac{\partial p^i}{\partial {\,\widehat{\!p\!}\,\,}_M^j} \right) \Big \vert \\&\quad = \left| \Lambda ({\,\widehat{\!p\!}\,\,}, h(t,x)) - \Lambda ({\,\widehat{\!p\!}\,\,},0) \right| \le \sup \vert \partial \Lambda \vert \vert h(t,x) \vert \le C \varepsilon , \end{aligned}$$

since $\vert {\,\widehat{\!p\!}\,\,}^i \vert \le c <1$, and hence the change of variables $(t,x,p) \mapsto (t,x,{\,\widehat{\!p\!}\,\,})$ is well defined if $\varepsilon$ is sufficiently small. Moreover, recalling that

$$\begin{aligned} p^0 = \sqrt{- \frac{1}{g_{\alpha \beta } {\,\widehat{\!p\!}\,\,}^{\alpha } {\,\widehat{\!p\!}\,\,}^{\beta }}}, \end{aligned}$$

it follows that, for each $\mu , \nu = 0,1,2,3$,

$$\begin{aligned} p^{\mu } p^{\nu } \frac{\sqrt{-\det g}}{p^0} \det \left( \frac{\partial p^i}{\partial {\,\widehat{\!p\!}\,\,}^j} \right) (t,x,{\,\widehat{\!p\!}\,\,}) = \Lambda ^{\mu \nu }({\,\widehat{\!p\!}\,\,}, h(t,x)) \end{aligned}$$

for some functions $\Lambda ^{\mu \nu }$, smooth when $\vert {\,\widehat{\!p\!}\,\,}\vert \le c <1$, and so

5.1 $$\begin{aligned} T^{\mu \nu }(t,x) = \int f(t,x,{\,\widehat{\!p\!}\,\,}) \Lambda ^{\mu \nu }({\,\widehat{\!p\!}\,\,},h(t,x)) \,\mathrm{d}{\,\widehat{\!p\!}\,\,}^1 \,\mathrm{d}{\,\widehat{\!p\!}\,\,}^2 \,\mathrm{d}{\,\widehat{\!p\!}\,\,}^3. \end{aligned}$$

For each vector field $\overline{Z}$, recall the corresponding functions $\mathring{Z}^k(t,x,{\,\widehat{\!p\!}\,\,})$ defined in Section 4.1. Note that, for each $\overline{Z}$, the $\mathring{Z}^k$ have the form

$$\begin{aligned} \mathring{Z}^k(t,x,{\,\widehat{\!p\!}\,\,}) = \mathring{Z}^k_{1,l}(t,x) {\,\widehat{\!p\!}\,\,}^l + \mathring{Z}^k_2 (t,x) \end{aligned}$$

for some functions $\mathring{Z}^k_{1,l}(t,x), \mathring{Z}^k_{2}(t,x)$. Explicitly,

$$\begin{aligned} \mathring{\Omega }_{ij, 1,l}^k \equiv 0, \quad \mathring{\Omega }_{ij,2}^k(t,x) = \Theta ^i(t,x) \delta _j^k - \Theta ^j(t,x) \delta _i^k - \Omega _{ij} \left( \Theta ^k(t,x) \right) ,\\ \mathring{B}_{i,1,l}^k(t,x) = - \Theta ^i(t,x) \delta ^k_l \quad \mathring{B}_{i,2}^k(t,x) = - B_i \left( \Theta ^k(t,x) \right) - \frac{x^i}{t-t_0} \Theta ^k(t,x), \end{aligned}$$

and

$$\begin{aligned} \mathring{S}_{1,l}^k \equiv 0, \quad \mathring{S}_2^k (t,x) = \Theta ^k(t,x) - S \left( \Theta ^k(t,x) \right) - \frac{t}{t-t_0} \Theta ^k(t,x). \end{aligned}$$

This notation will be used below.

#### Proposition 5.2

For $\mu ,\nu =0,1,2,3$ and any multi index *I*, there exist smooth functions $\Lambda ^{\mu \nu }_{I_j}$, $\Lambda ^{\mu \nu }_{J_j,l_j}$, $\Lambda ^{\alpha _j \beta _j, \mu \nu }_{J_1}$, $\widetilde{\Lambda }^{\mu \nu }_{L_j}$ such that

$$\begin{aligned} Z^I T^{\mu \nu } (t,x) =&\sum _{\vert I_1 \vert + \vert I_2 \vert \le \vert I \vert } \int \overline{Z}^{I_1} \left( f(t,x,{\,\widehat{\!p\!}\,\,}) \right) \Lambda ^{\mu \nu }_{I_1}({\,\widehat{\!p\!}\,\,},h)\\&\qquad \times \sum _{k=0}^{\vert I_2 \vert }\,\,\, \sum _{ \begin{array}{c} \vert J_1 \vert + \cdots + \vert J_k\vert \le \vert I_2 \vert , \,\,\, \vert J_i \vert \ge 1 \end{array}}\\&\qquad \sum _{ \begin{array}{c} \vert L_1 \vert + \cdots + \vert L_k\vert \le \vert I_2 \vert - 1, \,\,\, \vert L_i \vert \ge 1 \end{array}} \left( \overline{Z}^{J_1} ({\,\widehat{\!p\!}\,\,}^{l_1}) \Lambda ^{\mu \nu }_{J_1,l_1}({\,\widehat{\!p\!}\,\,},h) \right. \\&\qquad \left. + \overline{Z}^{J_1}(h_{\alpha _1 \beta _1}) \Lambda ^{\alpha _1 \beta _1, \mu \nu }_{J_1} ({\,\widehat{\!p\!}\,\,}, h) \right. \\&\qquad \left. + \overline{Z}^{L_1}(\mathring{Z}^m_{1,m}) \widetilde{\Lambda }^{\mu \nu }_{L_1}({\,\widehat{\!p\!}\,\,},h) \right) \\&\qquad \times \ldots \times \left( \overline{Z}^{J_k} ({\,\widehat{\!p\!}\,\,}^{l_k}) \Lambda ^{\mu \nu }_{J_k,l_k}({\,\widehat{\!p\!}\,\,},h) + \overline{Z}^{J_k}(h_{\alpha _k \beta _k}) \Lambda ^{\alpha _k \beta _k, \mu \nu }_{J_k} ({\,\widehat{\!p\!}\,\,}, h) \right. \\&\qquad \left. + \overline{Z}^{L_k}(\mathring{Z}^m_{1,m}) \widetilde{\Lambda }^{\mu \nu }_{L_k}({\,\widehat{\!p\!}\,\,},h) \right) \,\mathrm{d}{\,\widehat{\!p\!}\,\,}, \end{aligned}$$

where $Z^I$ is a product of $\vert I \vert$ of the vector fields $\Omega _{ij}, B_i, S$.

#### Proof

Recall that the components of the energy momentum tensor take the form (5.1). Note that

$$\begin{aligned} \overline{Z}\left( \Lambda ^{\mu \nu } ({\,\widehat{\!p\!}\,\,},h(t,x)) \right)= & {} \overline{Z}({\,\widehat{\!p\!}\,\,}^l) (\partial _{{\,\widehat{\!p\!}\,\,}^l} \Lambda ^{\mu \nu })({\,\widehat{\!p\!}\,\,}, h(t,x)) \\&+ \sum _{\alpha \beta } (Zh_{\alpha \beta })(t,x) (\partial _{h_{\alpha \beta }} \Lambda ^{\mu \nu })({\,\widehat{\!p\!}\,\,},h(t,x)), \end{aligned}$$

and, for $$Z = \Omega _{ij}, B_i, S$$,

$$\begin{aligned} Z \Big ( \int \eta (t,x,{\,\widehat{\!p\!}\,\,}) \,\mathrm{d}{\,\widehat{\!p\!}\,\,}\Big )= & {} \int \left( \overline{Z}- \mathring{Z}^k \partial _{{\,\widehat{\!p\!}\,\,}^k} \right) \eta (t,x,{\,\widehat{\!p\!}\,\,}) \,\mathrm{d}{\,\widehat{\!p\!}\,\,}\\= & {} \int \overline{Z}\left( \eta (t,x,{\,\widehat{\!p\!}\,\,}) \right) + (\partial _{{\,\widehat{\!p\!}\,\,}^k} \mathring{Z}^k)(t,x) \eta (t,x,{\,\widehat{\!p\!}\,\,})\,\mathrm{d}{\,\widehat{\!p\!}\,\,}, \end{aligned}$$

for any function $\eta (t,x,{\,\widehat{\!p\!}\,\,})$ and

$$\begin{aligned} (\partial _{{\,\widehat{\!p\!}\,\,}^k} \mathring{Z}^k)(t,x) = \mathring{Z}^k_{1,k}(t,x). \end{aligned}$$

Therefore, for $\vert I \vert =1$ and $Z = \Omega _{ij}, B_i, S$,

$$\begin{aligned} Z T^{\mu \nu } (t,x)= & {} \int \left( \overline{Z}\left( f(t,x,{\,\widehat{\!p\!}\,\,}) \right) + \mathring{Z}^m_{1,m}(t,x) f(t,x,{\,\widehat{\!p\!}\,\,}) \right) \Lambda ^{\mu \nu }({\,\widehat{\!p\!}\,\,}, h(t,x))\\&+ f(t,x,{\,\widehat{\!p\!}\,\,}) \left( \overline{Z}({\,\widehat{\!p\!}\,\,}^l) (\partial _{{\,\widehat{\!p\!}\,\,}^l} \Lambda ^{\mu \nu })({\,\widehat{\!p\!}\,\,}, h(t,x)) \right. \\&\left. + (Zh_{\alpha \beta })(t,x) (\partial _{h_{\alpha \beta }} \Lambda ^{\mu \nu })({\,\widehat{\!p\!}\,\,},h(t,x)) \right) \,\mathrm{d}{\,\widehat{\!p\!}\,\,}, \end{aligned}$$

and the proof follows from the fact that $\partial _{{\,\widehat{\!p\!}\,\,}^l} \Lambda ^{\mu \nu }$ and $\partial _{h_{\alpha \beta }} \Lambda ^{\mu \nu }$ are smooth functions of ${\,\widehat{\!p\!}\,\,}$ and *h*. The proof for $\vert I \vert \ge 1$ follows from a straightforward induction argument.

$$\square $$

#### Proposition 5.3

For any multi index *I*, there exist constants $C_{I,k,J,L}$ such that

$$\begin{aligned} \overline{Z}^{I} \left( f(t,x,{\,\widehat{\!p\!}\,\,}) \right) =&\,\sum _{k+m=1}^{\vert I\vert } \sum _{ \begin{array}{c} \vert J_1\vert + \cdots + \vert J_k \vert \\ + \vert L_1\vert + \cdots + \vert L_m \vert \le \vert I\vert ,\\ \vert J_i \vert \ge 1, \quad \vert L_i \vert \ge 1 \end{array}} \overline{Z}^{J_1} \big ( X(t_0)^{i_1} \big ) \ldots \overline{Z}^{J_k} \big ( X(t_0)^{i_k} \big )\\&\qquad \overline{Z}^{L_1} \big ( {\,\widehat{\!P\!}\,\,}(t_0)^{l_1} \big ) \ldots \overline{Z}^{L_m} \big ( {\,\widehat{\!P\!}\,\,}(t_0)^{l_m} \big )\\&\quad \times C_{I,k,J,L} \left( \partial _{x^{i_1}} \ldots \partial _{x^{i_k}} \partial _{{\,\widehat{\!p\!}\,\,}^{l_1}} \ldots \partial _{{\,\widehat{\!p\!}\,\,}^{l_m}} f \right) (t_0,X(t_0),{\,\widehat{\!P\!}\,\,}(t_0)). \end{aligned}$$

#### Proof

Using the Vlasov equation to write

$$\begin{aligned} f(t,x,{\,\widehat{\!p\!}\,\,}) = f(t_0,X(t_0),{\,\widehat{\!P\!}\,\,}(t_0)), \end{aligned}$$

it follows that

$$\begin{aligned} \overline{Z}\left( f(t,x,{\,\widehat{\!p\!}\,\,}) \right)= & {} \overline{Z}\left( X(t_0)^i \right) (\partial _{x^i} f)(t_0,X(t_0),{\,\widehat{\!P\!}\,\,}(t_0)) \\&+ \overline{Z}\left( X(t_0)^l \right) (\partial _{{\,\widehat{\!p\!}\,\,}^l} f)(t_0,X(t_0),{\,\widehat{\!P\!}\,\,}(t_0)). \end{aligned}$$

The proof for $\vert I \vert \ge 2$ follows from a straightforward induction argument. $\quad \square$

#### Proposition 5.4

Suppose $t\ge t_0 +1$, $\vert x \vert \le c t$, and the bounds (4.1) hold. Then, for each $\mu ,\nu = 0,1,2,3$ and any multi index *I* with $\vert I \vert \le N$ and $Z^I$ equal to a product of $\vert I \vert$ of the vector fields $\Omega _{ij}, B_i, S$,

$$\begin{aligned} \left| Z^I T^{\mu \nu }(t,x) \right|\le & {} C \!\!\!\!\!\sum _{n_1+n_2 \le \vert I \vert }\! \int \left| (\partial ^{n_1}_x \partial ^{n_2}_{{\,\widehat{\!p\!}\,\,}} f)(t_0,X(t_0),{\,\widehat{\!P\!}\,\,}(t_0)) \right| \,\mathrm{d}{\,\widehat{\!p\!}\,\,}\\&\quad + C \!\!\!\!\!\!\!\!\!\!\sum _{n_1+n_2 \le \left\lfloor \frac{\vert I \vert }{2} \right\rfloor + 1}\!\!\!\!\!\! \int \left| (\partial ^{n_1}_x \partial ^{n_2}_{{\,\widehat{\!p\!}\,\,}} f)(t_0,X(t_0),{\,\widehat{\!P\!}\,\,}(t_0)) \right| \times \\&\quad \times \bigg ( \sum _{\vert J \vert \le \vert I \vert -1}\!\!\!\!\! t^{2-a} \vert (Z^J \Gamma )(t,x) \vert + \sum _{\vert J \vert \le \vert I \vert } \int _{t_0}^t s^{1-a} \left| (Z^J \Gamma ) \left( s, s\frac{x}{t} \right) \right| \\&\quad + s \left| (Z^J \Gamma ) \left( s, s\frac{x}{t} \right) - (Z^J \Gamma ) \left( s, X(s) \right) \right| \, \mathrm{d}s \bigg ) \,\mathrm{d}{\,\widehat{\!p\!}\,\,}. \end{aligned}$$

#### Proof

Recall the schematic expression for $Z^I T^{\mu \nu }$ of Proposition 5.2. Consider multi indices $I_1$, $I_2$ such that $\vert I_1 \vert + \vert I_2 \vert \le \vert I \vert$, and suppose first that $\vert I_1 \vert \ge \big \lfloor \frac{\vert I \vert }{2} \big \rfloor + 1$. It must then be the case that $\vert I_2 \vert \le \big \lfloor \frac{\vert I \vert }{2} \big \rfloor + 1 \le \big \lfloor \frac{N}{2} \big \rfloor + 1$. If $1 \le \vert J_i \vert \le \vert I_2 \vert$, then clearly

$$\begin{aligned} \big \vert \overline{Z}^{J_i} \left( h_{\alpha _i \beta _i} \right) (t,x) \big \vert \le C, \end{aligned}$$

and Proposition 4.9 implies that

$$\begin{aligned} \big \vert \overline{Z}^{J_i} \big ( {\,\widehat{\!p\!}\,\,}^i \big ) \big \vert \le C. \end{aligned}$$

If $1 \le \vert L_i \vert \le \vert I_2 \vert - 1$, then, since

$$\begin{aligned} \mathring{B}_{i,1,k}^k (t,x) = - 3 \Theta ^i(t,x), \quad \mathring{\Omega }_{ij,1,k}^k (t,x) = 0, \quad \mathring{S}_{1,k}^k = 0, \end{aligned}$$

Proposition 4.8 implies

$$\begin{aligned} \left| \overline{Z}^{L_i} \left( \mathring{Z}_{1,k}^k \right) \right| \le C \end{aligned}$$

for each $\mathring{Z}_{1,k}^k$. Let now $k,m,J_1,\ldots ,J_k, L_1,\ldots ,L_m$ be such that $1 \le k+m \le \vert I_1 \vert$, $\vert J_1 \vert + \cdots + \vert J_k \vert + \vert L_1 \vert + \ldots + \vert L_m\vert \le \vert I_1 \vert$, $\vert J_i \vert \ge 1$, $\vert L_i \vert \ge 1$. If $k + m \ge \big \lfloor \frac{\vert I_1 \vert }{2} \big \rfloor + 2$ then it must be the case that $\vert J_i \vert \le \big \lfloor \frac{\vert I_1 \vert }{2} \big \rfloor + 1 \le \big \lfloor \frac{\vert N \vert }{2} \big \rfloor + 1$ for $i=1,\ldots ,k$, and $\vert L_i \vert \le \big \lfloor \frac{\vert I_1 \vert }{2} \big \rfloor + 1 \le \big \lfloor \frac{\vert N \vert }{2} \big \rfloor + 1$ for $i=1,\ldots ,m$. Proposition 4.24 then implies

$$\begin{aligned}&\Big \vert \overline{Z}^{J_1} \left( X(t_0)^{i_1} \right) \ldots \overline{Z}^{J_k} \left( X(t_0)^{i_k} \right) \overline{Z}^{L_1} \big ( {\,\widehat{\!P\!}\,\,}(t_0)^{l_1} \big ) \ldots \overline{Z}^{L_m} \big ( {\,\widehat{\!P\!}\,\,}(t_0)^{l_m} \big )\\&\qquad \times \left( \partial _{x^{i_1}} \ldots \partial _{x^{i_k}} \partial _{{\,\widehat{\!p\!}\,\,}^{l_1}} \ldots \partial _{{\,\widehat{\!p\!}\,\,}^{l_m}} f \right) (t_0,X(t_0),{\,\widehat{\!P\!}\,\,}(t_0)) \Big \vert \\&\quad \le C \sum _{n_1+n_2 \le \vert I_1 \vert } \left| \partial _{x}^{n_1} \partial _{{\,\widehat{\!p\!}\,\,}}^{n_2} f (t_0,X(t_0),{\,\widehat{\!P\!}\,\,}(t_0)) \right| . \end{aligned}$$

Similarly, if $k+m \le \big \lfloor \frac{\vert I_1 \vert }{2} \big \rfloor + 1$, there can be at most one *i* such that either $\vert J_i \vert \ge \big \lfloor \frac{\vert I_1 \vert }{2} \big \rfloor + 2$ or $\vert L_i \vert \ge \big \lfloor \frac{\vert I_1 \vert }{2} \big \rfloor + 2$, so Proposition 4.24 and Corollary 4.27 imply

$$\begin{aligned}&\Big \vert \overline{Z}^{J_1} \left( X(t_0)^{i_1} \right) \ldots \overline{Z}^{J_k} \left( X(t_0)^{i_k} \right) \overline{Z}^{L_1} \big ( {\,\widehat{\!P\!}\,\,}(t_0)^{l_1} \big ) \ldots \overline{Z}^{L_m} \big ( {\,\widehat{\!P\!}\,\,}(t_0)^{l_m} \big )\\&\qquad \times \left( \partial _{x^{i_1}} \ldots \partial _{x^{i_k}} \partial _{{\,\widehat{\!p\!}\,\,}^{l_1}} \ldots \partial _{{\,\widehat{\!p\!}\,\,}^{l_m}} f \right) (t_0,X(t_0),{\,\widehat{\!P\!}\,\,}(t_0)) \Big \vert \\&\quad \le C \sum _{n_1+n_2 \le \vert I_1 \vert } \left| \partial _{x}^{n_1} \partial _{{\,\widehat{\!p\!}\,\,}}^{n_2} f (t_0,X(t_0),{\,\widehat{\!P\!}\,\,}(t_0)) \right| \bigg ( 1 + \sum _{\vert J \vert \le \vert I \vert -1} t^{2-a} \vert (Z^J \Gamma )(t,x) \vert \\&\qquad + \sum _{\vert J \vert \le \vert I \vert } \int _{t_0}^t (s')^{1-a} \left| (Z^J \Gamma ) \left( s', s'\frac{x}{t} \right) \right| + s' \left| (Z^J \Gamma ) \left( s', s'\frac{x}{t} \right) - (Z^J \Gamma ) \left( s', X(s') \right) \right| \,\mathrm{d}s' \bigg ). \end{aligned}$$

It is then clear that

$$\begin{aligned}&\Bigg \vert \int \overline{Z}^{I_1} \left( f(t,x,{\,\widehat{\!p\!}\,\,}) \right) \\&\quad \times \sum _{k=0}^{\vert I_2 \vert }\,\,\,\,\, \sum _{ \begin{array}{c} \vert J_1 \vert + \cdots + \vert J_k\vert \le \vert I_2 \vert ,\,\,\, \vert J_i \vert \ge 1 \end{array}}\,\,\,\,\, \sum _{ \begin{array}{c} \vert L_1 \vert + \ldots + \vert L_k\vert \le \vert I_2 \vert - 1, \,\,\, \vert L_i \vert \ge 1 \end{array}}\\&\qquad \left( \overline{Z}^{J_1} ({\,\widehat{\!p\!}\,\,}^{l_1}) + \overline{Z}^{J_1}(h_{\alpha _1 \beta _1}) \Lambda ^{l_1, \alpha _1 \beta _1} ({\,\widehat{\!p\!}\,\,}, h) + \overline{Z}^{L_1}(\mathring{Z}^m_{1,m}) \Lambda ^{l_1} ({\,\widehat{\!p\!}\,\,}, h) \right) \\&\qquad \times \ldots \times \left( \overline{Z}^{J_k} ({\,\widehat{\!p\!}\,\,}^{l_k}) + \overline{Z}^{J_k}(h_{\alpha _k \beta _k}) \Lambda ^{l_k, \alpha _k \beta _k} ({\,\widehat{\!p\!}\,\,}, h) \right. \\&\qquad \left. + \overline{Z}^{L_k}(\mathring{Z}^m_{1,m}) \Lambda ^{l_k} ({\,\widehat{\!p\!}\,\,}, h) \right) \Lambda ({\,\widehat{\!p\!}\,\,},h)\,\mathrm{d}{\,\widehat{\!p\!}\,\,}\Bigg \vert \\&\quad \le C \sum _{n_1+n_2 \le \vert I \vert } \int \left| (\partial ^{n_1}_x \partial ^{n_2}_{{\,\widehat{\!p\!}\,\,}} f)(t_0,X(t_0),{\,\widehat{\!P\!}\,\,}(t_0)) \right| \,\mathrm{d}{\,\widehat{\!p\!}\,\,}\\&\qquad + C \sum _{n_1+n_2 \le \left\lfloor \frac{\vert I \vert }{2} \right\rfloor + 1} \int \left| (\partial ^{n_1}_x \partial ^{n_2}_{{\,\widehat{\!p\!}\,\,}} f)(t_0,X(t_0),{\,\widehat{\!P\!}\,\,}(t_0)) \right| \times \\&\qquad \times \Big ( \sum _{\vert J \vert \le \vert I \vert -1} t^{2-a} \vert (Z^J \Gamma )(t,x) \vert + \sum _{\vert J \vert \le \vert I \vert } \int _{t_0}^t s^{1-a} \left| (Z^J \Gamma ) \left( s, s\frac{x}{t} \right) \right| \\&\qquad + s \left| (Z^J \Gamma ) \left( s, s\frac{x}{t} \right) - (Z^J \Gamma ) \left( s, X(s) \right) \right| \,\mathrm{d}s \Big ) \,\mathrm{d}{\,\widehat{\!p\!}\,\,}. \end{aligned}$$

Suppose now that $\vert I_2 \vert \ge \big \lfloor \frac{\vert I \vert }{2} \big \rfloor + 1$. It must then be the case that $\vert I_1 \vert \le \big \lfloor \frac{\vert I \vert }{2} \big \rfloor + 1 \le \big \lfloor \frac{\vert N \vert }{2} \big \rfloor + 1$. Proposition 5.3 then implies that

$$\begin{aligned} \left| \overline{Z}^{I_1} \left( f(t,x,{\,\widehat{\!p\!}\,\,}) \right) \right| \le C \sum _{n_1+n_2 \le \left\lfloor \frac{\vert I \vert }{2} \right\rfloor + 1} \left| (\partial ^{n_1}_x \partial ^{n_2}_{{\,\widehat{\!p\!}\,\,}} f)(t_0,X(t_0),{\,\widehat{\!P\!}\,\,}(t_0)) \right| . \end{aligned}$$

If $\vert J_i \vert \le \vert I_2\vert$ and $\vert L_i \vert \le \vert I_2 \vert -1$, then Propositions 4.8 and 4.9 imply

$$\begin{aligned}&\left| Z^{J_i} \big ( {\,\widehat{\!p\!}\,\,}^k \big ) \right| + \left| Z^{J_i} \big ( h_{\alpha _k \beta _k} \big ) \right| + \left| Z^{J_i} \big ( \mathring{Z}_{1,k}^k \big ) \right| \\&\quad \le C \Big ( 1 +\!\!\!\! \sum _{\vert J \vert \le \vert I_2 \vert -1}\!\!\!\! t \left| \left( Z^J \Gamma \right) (t,x) \right| \\&\qquad +\sum _{\vert J \vert \le \vert I_2 \vert } \int _{t_0}^t \left| \left( Z^J \Gamma \right) \left( s',s'\frac{x}{t} \right) \right| \,\mathrm{d}s' \Big ). \end{aligned}$$

Hence,

$$\begin{aligned}&\Bigg \vert \int \overline{Z}^{I_1} \left( f(t,x,{\,\widehat{\!p\!}\,\,}) \right) \times \sum _{k=0}^{\vert I_2 \vert }\,\,\,\,\, \sum _{ \begin{array}{c} \vert J_1 \vert + \cdots + \vert J_k\vert \le \vert I_2 \vert ,\,\,\, \vert J_i \vert \ge 1 \end{array}}\,\,\,\,\, \sum _{ \begin{array}{c} \vert L_1 \vert + \ldots + \vert L_k\vert \le \vert I_2 \vert - 1,\,\,\, \vert L_i \vert \ge 1 \end{array}}\\&\qquad \left( \overline{Z}^{J_1} ({\,\widehat{\!p\!}\,\,}^{l_1}) + \overline{Z}^{J_1}(h_{\alpha _1 \beta _1}) \Lambda ^{l_1, \alpha _1 \beta _1} ({\,\widehat{\!p\!}\,\,}, h) + \overline{Z}^{L_1}(\mathring{Z}^m_{1,m}) \Lambda ^{l_1} ({\,\widehat{\!p\!}\,\,}, h) \right) \\&\qquad \times \ldots \times \left( \overline{Z}^{J_k} ({\,\widehat{\!p\!}\,\,}^{l_k}) + \overline{Z}^{J_k}(h_{\alpha _k \beta _k}) \Lambda ^{l_k, \alpha _k \beta _k} ({\,\widehat{\!p\!}\,\,}, h) \right. \\&\qquad \left. +\overline{Z}^{L_k}(\mathring{Z}^m_{1,m}) \Lambda ^{l_k} ({\,\widehat{\!p\!}\,\,}, h) \right) \Lambda ({\,\widehat{\!p\!}\,\,},h)\,\mathrm{d}{\,\widehat{\!p\!}\,\,}\Bigg \vert \\&\quad \le C\!\!\!\!\!\!\!\!\!\!\! \sum _{n_1+n_2 \le \left\lfloor \frac{\vert I \vert }{2} \right\rfloor + 1} \!\!\!\int \left| (\partial ^{n_1}_x \partial ^{n_2}_{{\,\widehat{\!p\!}\,\,}} f)(t_0,X(t_0),{\,\widehat{\!P\!}\,\,}(t_0)) \right| \Big ( 1 +\!\!\!\!\! \sum _{\vert J \vert \le \vert I_2 \vert -1}\!\!\!\!\! t \left| \left( Z^J \Gamma \right) (t,x) \right| \\&\qquad + \sum _{\vert J \vert \le \vert I_2 \vert } \!\!\int _{t_0}^t\!\! \left| \left( Z^J \Gamma \right) \left( s',s'\frac{x}{t} \right) \right| \mathrm{d}s' \Big ) \,\mathrm{d}{\,\widehat{\!p\!}\,\,}. \end{aligned}$$

The proof then follows from Proposition 5.2. $\quad \square$

### Determinants and Changes of Variables

In the proof of Theorem 1.3, the change of variables $(t,x,{\,\widehat{\!p\!}\,\,}) \mapsto (t,x,y)$ will be used, where

$$\begin{aligned} y^i(t,x,{\,\widehat{\!p\!}\,\,}) = X(t_0,t,x,{\,\widehat{\!p\!}\,\,})^i \end{aligned}$$

for $i=1,2,3$, along with several other changes of variables; see the proof of Propositions 5.8 and 5.9. A first step towards controlling the determinants of these changes is contained in the following:

#### Lemma 5.5

Suppose $t\ge t_0$, $\vert x \vert \le ct$, and $(t,x,{\,\widehat{\!p\!}\,\,}) \in \mathrm {supp}(f)$. Then, if the assumption (4.1) holds and $\varepsilon$ is sufficiently small,

5.2 $$\begin{aligned} \left| \frac{\partial X^i}{\partial {\,\widehat{\!p\!}\,\,}^j}(s,t,x,{\,\widehat{\!p\!}\,\,}) + (t-s) \delta ^i_j \right| \lesssim \frac{\varepsilon t}{s^a}, \qquad \left| \frac{\partial {\,\widehat{\!P\!}\,\,}^i}{\partial {\,\widehat{\!p\!}\,\,}^j}(s,t,x,{\,\widehat{\!p\!}\,\,}) - \delta ^i_j \right| \lesssim \frac{\varepsilon t}{s^{1+a}}, \end{aligned}$$

and

5.3 $$\begin{aligned} \left| \frac{\partial X^i}{\partial x^j}(s,t,x,{\,\widehat{\!p\!}\,\,}) - \delta ^i_j \right| \lesssim \frac{\varepsilon }{s^a}, \qquad \left| \frac{\partial {\,\widehat{\!P\!}\,\,}^i}{\partial x^j}(s,t,x,{\,\widehat{\!p\!}\,\,}) \right| \lesssim \frac{\varepsilon }{s^{1+a}} \end{aligned}$$

for all $t_0 \le s \le t$, and $i,j=1,2,3$.

#### Proof

From the equations (1.17) and the estimates

$$\begin{aligned} s^{1+a} \left| \Gamma ^{\alpha }_{\beta \gamma }(s,X(s)) \right| + s^{2+a} \left| \partial _k \Gamma ^{\alpha }_{\beta \gamma }(s,X(s)) \right| \lesssim \varepsilon , \end{aligned}$$

it follows that

$$\begin{aligned}&\left| \frac{\mathrm{d}}{\mathrm{d}s} \frac{\partial X^i}{\partial {\,\widehat{\!p\!}\,\,}^j} \right| \le \left| \frac{\partial {\,\widehat{\!P\!}\,\,}^i}{\partial {\,\widehat{\!p\!}\,\,}^j} \right| , \qquad \\&\left| \frac{\mathrm{d}}{\mathrm{d}s} \frac{\partial {\,\widehat{\!P\!}\,\,}^i}{\partial {\,\widehat{\!p\!}\,\,}^j}(s) \right| \lesssim \varepsilon \sum _{k,l=1}^3 \left( \frac{1}{s^{2+a}} \left| \frac{\partial X^k}{\partial {\,\widehat{\!p\!}\,\,}^l}(s) \right| + \frac{1}{s^{1+a}} \left| \frac{\partial {\,\widehat{\!P\!}\,\,}^k}{\partial {\,\widehat{\!p\!}\,\,}^l}(s) \right| \right) . \end{aligned}$$

Integrating the second inequality backwards from $s=t$ and using the fact that $\frac{\partial {\,\widehat{\!P\!}\,\,}^k}{\partial {\,\widehat{\!p\!}\,\,}^l}(t,t,x,{\,\widehat{\!p\!}\,\,}) = \delta ^i_j$ gives

$$\begin{aligned} \left| \frac{\partial {\,\widehat{\!P\!}\,\,}^i}{\partial {\,\widehat{\!p\!}\,\,}^j}(s,t,x,{\,\widehat{\!p\!}\,\,}) - \delta ^i_j \right|&\lesssim \varepsilon \sum _{k,l=1}^3 \int _s^t \frac{1}{\tilde{s}^{2+a}} \left| \frac{\partial X^k}{\partial {\,\widehat{\!p\!}\,\,}^l}(\tilde{s}) \right| + \frac{1}{\tilde{s}^{1+a}} \left| \frac{\partial {\,\widehat{\!P\!}\,\,}^k}{\partial {\,\widehat{\!p\!}\,\,}^l}(\tilde{s}) \right| \,\mathrm{d}\tilde{s}\\&\lesssim \frac{\varepsilon }{s^a} + \varepsilon \sum _{k,l=1}^3 \int _s^t \frac{1}{\tilde{s}^{2+a}} \left| \frac{\partial X^k}{\partial {\,\widehat{\!p\!}\,\,}^l}(\tilde{s}) \right| \\&\quad + \frac{1}{\tilde{s}^{1+a}} \left| \frac{\partial {\,\widehat{\!P\!}\,\,}^k}{\partial {\,\widehat{\!p\!}\,\,}^l}(\tilde{s}) - \delta ^k_l \right| \,\mathrm{d}\tilde{s}. \end{aligned}$$

Dividing by $s^{1+a}$, integrating again backwards from $s=t$, and summing over *i*, *j* gives

$$\begin{aligned}&\sum _{k,l=1}^3 \int _s^t \frac{1}{(s')^{1+a}} \left| \frac{\partial {\,\widehat{\!P\!}\,\,}^k}{\partial {\,\widehat{\!p\!}\,\,}^l}(s') - \delta ^k_l \right| d s'\\&\quad \lesssim \int _s^t \frac{1}{(s')^{1+a}} \left( \frac{\varepsilon }{(s')^a} + \varepsilon \sum _{k,l=1}^3 \int _{s'}^t \frac{1}{\tilde{s}^{2+a}} \left| \frac{\partial X^k}{\partial {\,\widehat{\!p\!}\,\,}^l}(\tilde{s}) \right| + \frac{1}{\tilde{s}^{1+a}} \left| \frac{\partial {\,\widehat{\!P\!}\,\,}^k}{\partial {\,\widehat{\!p\!}\,\,}^l}(\tilde{s}) - \delta ^k_l \right| \,\mathrm{d}\tilde{s} \right) \mathrm{d}s'\\&\quad \lesssim \left( \frac{\varepsilon }{s^a} + \varepsilon \sum _{k,l=1}^3 \int _{s}^t \frac{1}{\tilde{s}^{2+a}} \left| \frac{\partial X^k}{\partial {\,\widehat{\!p\!}\,\,}^l}(\tilde{s}) \right| + \frac{1}{\tilde{s}^{1+a}} \left| \frac{\partial {\,\widehat{\!P\!}\,\,}^k}{\partial {\,\widehat{\!p\!}\,\,}^l}(\tilde{s}) - \delta ^k_l \right| \,\mathrm{d}\tilde{s} \right) \int _s^t \frac{1}{(s')^{1+a}} \mathrm{d}s'\\&\quad \lesssim \frac{\varepsilon }{s^a} + \varepsilon \sum _{k,l=1}^3 \int _{s}^t \frac{1}{\tilde{s}^{2+a}} \left| \frac{\partial X^k}{\partial {\,\widehat{\!p\!}\,\,}^l}(\tilde{s}) \right| + \frac{1}{\tilde{s}^{1+a}} \left| \frac{\partial {\,\widehat{\!P\!}\,\,}^k}{\partial {\,\widehat{\!p\!}\,\,}^l}(\tilde{s}) - \delta ^k_l \right| \,\mathrm{d}\tilde{s}. \end{aligned}$$

Taking $\varepsilon$ sufficiently small then gives

$$\begin{aligned} \sum _{k,l=1}^3 \int _s^t \frac{1}{\tilde{s}^{1+a}} \left| \frac{\partial {\,\widehat{\!P\!}\,\,}^k}{\partial {\,\widehat{\!p\!}\,\,}^l}(\tilde{s}) - \delta ^k_l \right| \,\mathrm{d}\tilde{s} \lesssim \frac{\varepsilon }{s^a} + \varepsilon \sum _{k,l=1}^3 \int _s^t \frac{1}{\tilde{s}^{2+a}} \left| \frac{\partial X^k}{\partial {\,\widehat{\!p\!}\,\,}^l}(\tilde{s}) \right| \,\mathrm{d}\tilde{s}. \end{aligned}$$

Inserting back into the above bound gives

5.4 $$\begin{aligned} \left| \frac{\partial {\,\widehat{\!P\!}\,\,}^i}{\partial {\,\widehat{\!p\!}\,\,}^j}(s,t,x,{\,\widehat{\!p\!}\,\,}) - \delta ^i_j \right| \lesssim \frac{\varepsilon }{s^a} + \varepsilon \sum _{k,l=1}^3 \int _s^t \frac{1}{\tilde{s}^{2+a}} \left| \frac{\partial X^k}{\partial {\,\widehat{\!p\!}\,\,}^l}(\tilde{s}) \right| \,\mathrm{d}\tilde{s}. \end{aligned}$$

Integrating this bound backwards from $s=t$, and using the fact that $\frac{\partial X^i}{\partial {\,\widehat{\!p\!}\,\,}^j}(t,t,x,{\,\widehat{\!p\!}\,\,}) = 0$, gives

$$\begin{aligned} \left| \frac{\partial X^i}{\partial {\,\widehat{\!p\!}\,\,}^j}(s,t,x,{\,\widehat{\!p\!}\,\,}) + (t-s) \delta ^i_j \right|&\lesssim \varepsilon t^{1-a} + \varepsilon \sum _{k,l=1}^3 \int _s^t \frac{1}{\tilde{s}^{1+a}} \left| \frac{\partial X^k}{\partial {\,\widehat{\!p\!}\,\,}^l}(\tilde{s}) \right| \,\mathrm{d}\tilde{s}\\&\lesssim \frac{\varepsilon t}{s^a} + \varepsilon \sum _{k,l=1}^3 \int _s^t \frac{1}{\tilde{s}^{1+a}} \left| \frac{\partial X^k}{\partial {\,\widehat{\!p\!}\,\,}^l}(\tilde{s}) + (t-s) \delta ^k_l \right| \,\mathrm{d}\tilde{s}, \end{aligned}$$

where the fact that, for any function $\lambda (s)$,

$$\begin{aligned} \int _s^t \int _{s'}^t \lambda (\tilde{s}) \,\mathrm{d}\tilde{s} \mathrm{d}s' = \int _s^t \int _s^t \chi _{\{ s'\le \tilde{s}\}} \mathrm{d}s' \lambda (\tilde{s})\,\mathrm{d}\tilde{s} = \int _s^t (\tilde{s} - s) \lambda (\tilde{s})\,\mathrm{d}\tilde{s} \end{aligned}$$

has been used (here $\chi _{\{ s'\le \tilde{s}\}}$ is the indicator function of the interval $[s',\tilde{s}]$). Again, dividing by $s^{1+a}$, integrating backwards from $s=t$, summing over *i*, *j* and taking $\varepsilon$ small gives

$$\begin{aligned} \sum _{k,l=1}^3 \int _s^t \frac{1}{\tilde{s}^{1+a}} \left| \frac{\partial X^k}{\partial {\,\widehat{\!p\!}\,\,}^l}(\tilde{s}) + (t-s) \delta ^k_l \right| \,\mathrm{d}\tilde{s} \lesssim \frac{\varepsilon t}{s^{2a}}. \end{aligned}$$

Inserting back into the above bound then gives

$$\begin{aligned} \left| \frac{\partial X^i}{\partial {\,\widehat{\!p\!}\,\,}^j}(s,t,x,{\,\widehat{\!p\!}\,\,}) + (t-s) \delta ^i_j \right| \lesssim \frac{\varepsilon t}{s^a}, \end{aligned}$$

and inserting this into (5.4) gives the second bound of (5.2).

In a similar manner, it is straightforward to show that

$$\begin{aligned} \left| \frac{\mathrm{d}}{\mathrm{d}s} \frac{\partial {\,\widehat{\!P\!}\,\,}^i}{\partial x^j}(s) \right| \lesssim \varepsilon \sum _{k,l=1}^3 \left( \frac{1}{s^{2+a}} \left| \frac{\partial X^k}{\partial x^l}(s) \right| + \frac{1}{s^{1+a}} \left| \frac{\partial {\,\widehat{\!P\!}\,\,}^k}{\partial x^l}(s) \right| \right) , \end{aligned}$$

and, using the final conditions $\frac{\partial {\,\widehat{\!P\!}\,\,}^i}{\partial x^j}(t,t,x,{\,\widehat{\!p\!}\,\,}) = 0$, $\frac{\partial X^i}{\partial x^j}(t,t,x,{\,\widehat{\!p\!}\,\,}) = \delta ^i_j$, that (5.3) holds. $\quad \square$

The properties of these changes are collected in the following proposition:

#### Proposition 5.6

For fixed *t*, *x* with $t \ge t_0 +1$, $\vert x \vert \le c t$, if the assumptions (4.1) hold and $\varepsilon$ is sufficiently small then, for ${\,\widehat{\!p\!}\,\,}$ such that $(t,x,{\,\widehat{\!p\!}\,\,}) \in \mathrm {supp}(f)$, the change of variables ${\,\widehat{\!p\!}\,\,}\mapsto y:= X(t_0,t,x,{\,\widehat{\!p\!}\,\,})$ satisfies

5.5 $$\begin{aligned} \left| \det \left( \frac{\partial {\,\widehat{\!p\!}\,\,}^i}{\partial y^j} \right) \right| \le \frac{C}{t^3}. \end{aligned}$$

Define

$$\begin{aligned} z_1^i(s,t,x,y):= & {} X(s,t,x,{\,\widehat{\!p\!}\,\,}(t,x,y))^i, \\ z_2^i(\sigma ,s,t,x,y):= & {} \sigma s \frac{x^i}{t} + (1-\sigma ) X(s,t,x,{\,\widehat{\!p\!}\,\,}(t,x,y))^i \end{aligned}$$

for $i=1,2,3$. If $t \ge t_0 +1$ and $t_0 \le s \le t_0+\frac{1}{2}$, then the change of variables $(x,y) \mapsto (x,z_1(s,t,x,y))$ satisfies

5.6 $$\begin{aligned} \left| \det \left( \frac{\partial z_1^i(s,t)}{\partial y^j} \right) ^{-1} \right| \le C. \end{aligned}$$

If $t_0+\frac{1}{2} \le s \le t$ then the change of variables $(x,y) \mapsto (z_1(s,t,x,y),y)$ satisfies

5.7 $$\begin{aligned} \left| \det \left( \frac{\partial z_1^i(s,t)}{\partial x^j} \right) ^{-1} \right| \le C \left( \frac{t}{s} \right) ^3. \end{aligned}$$

Finally, if $t_0+\frac{1}{2}\le s \le t$ and $0 \le \sigma \le 1$, then the change of variables $(x,y) \mapsto (z_2(\sigma ,s,t,x,y),y)$ satisfies

5.8 $$\begin{aligned} \left| \det \left( \frac{\partial z_2^i(\sigma ,s,t)}{\partial x^j} \right) ^{-1} \right| \le C \left( \frac{t}{s} \right) ^3. \end{aligned}$$

Moreover, for $t\ge t_0$, the determinant of the 6 by 6 matrix $\kappa$ satisfies

5.9 $$\begin{aligned} \left| \det \kappa (t_0,t,x,{\,\widehat{\!p\!}\,\,}) - 1 \right| \lesssim \varepsilon , \qquad \text {where } \kappa = \begin{pmatrix} \frac{\partial X}{\partial x} &{} \frac{\partial X}{\partial {\,\widehat{\!p\!}\,\,}} \\ \frac{\partial {\,\widehat{\!P\!}\,\,}}{\partial x} &{} \frac{\partial {\,\widehat{\!P\!}\,\,}}{\partial {\,\widehat{\!p\!}\,\,}} \end{pmatrix}. \end{aligned}$$

#### Proof

Setting $s=t_0$ in (5.2), it follows that

5.10 $$\begin{aligned} \left| \frac{\partial y^i}{\partial {\,\widehat{\!p\!}\,\,}^j} + (t-t_0) \delta ^i_j \right| \lesssim \varepsilon t, \end{aligned}$$

and, if $\varepsilon$ is sufficiently small,

5.11 $$\begin{aligned} \left| \det \left( \frac{\partial y^i}{\partial {\,\widehat{\!p\!}\,\,}^j} \right) + (t-t_0)^3 \right| \lesssim \varepsilon t^3. \end{aligned}$$

Since $t\ge t_0 +1$, the bound (5.5) follows.

For the remaining bounds of the proposition, it is necessary to consider ${\,\widehat{\!p\!}\,\,}^i$ as a function of *t*, *x*, *y* (using the above bound and the Inverse Function Theorem) and estimate $\frac{\partial {\,\widehat{\!p\!}\,\,}^i}{\partial x^j}$ and $\frac{\partial {\,\widehat{\!p\!}\,\,}^i}{\partial y^j}$. Clearly the matrix $\left( \frac{\partial {\,\widehat{\!p\!}\,\,}^i}{\partial y^j}\right)$ is the inverse of the matrix $\left( \frac{\partial y^i}{\partial {\,\widehat{\!p\!}\,\,}^j}\right)$, and hence it follows from (5.10) that

$$\begin{aligned} \left| \frac{\partial {\,\widehat{\!p\!}\,\,}^i}{\partial y^j} + \frac{\delta ^i_j}{t-t_0} \right| \lesssim \frac{\varepsilon }{t}. \end{aligned}$$

Also,

$$\begin{aligned} 0 = \frac{\partial {\,\widehat{\!p\!}\,\,}^i(t,x,y(t,x,{\,\widehat{\!p\!}\,\,}))}{\partial x^j} = \frac{\partial {\,\widehat{\!p\!}\,\,}^i}{\partial x^j}(t,x,y) + \frac{\partial {\,\widehat{\!p\!}\,\,}^i}{\partial y^k}(t,x,y) \frac{\partial y^k}{\partial x^j}, \end{aligned}$$

so

$$\begin{aligned} \frac{\partial {\,\widehat{\!p\!}\,\,}^i}{\partial x^j} = - \frac{\partial {\,\widehat{\!p\!}\,\,}^i}{\partial y^k} \frac{\partial y^k}{\partial x^j}. \end{aligned}$$

Setting $s=t_0$ in (5.3) gives

$$\begin{aligned} \left| \frac{\partial y^i}{\partial x^j} - \delta ^i_j \right| \lesssim \varepsilon , \end{aligned}$$

and so, since

$$\begin{aligned} \frac{\partial {\,\widehat{\!p\!}\,\,}^i}{\partial x^j} - \frac{\delta ^i_j}{t-t_0}= & {} - \left( \frac{\partial {\,\widehat{\!p\!}\,\,}^i}{\partial y^k} + \frac{\delta _k^i}{t-t_0} \right) \left( \frac{\partial y^k}{\partial x^j} - \delta ^k_j \right) \\&+ \frac{1}{t-t_0} \left( \frac{\partial y^i}{\partial x^j} - \delta ^i_j \right) - \left( \frac{\partial {\,\widehat{\!p\!}\,\,}^i}{\partial y^j} + \frac{\delta ^i_j}{t-t_0} \right) , \end{aligned}$$

it follows that

$$\begin{aligned} \left| \frac{\partial {\,\widehat{\!p\!}\,\,}^i}{\partial x^j} - \frac{\delta ^i_j}{t-t_0} \right| \lesssim \frac{\varepsilon }{t}, \end{aligned}$$

since $$t \ge t_0 +1$$.

Now,

$$\begin{aligned} \frac{\partial z_1^i}{\partial y^j} = \frac{\partial X^i(s,t,x,{\,\widehat{\!p\!}\,\,}(t,x,y))}{\partial y^j} = \frac{\partial X^i}{\partial {\,\widehat{\!p\!}\,\,}^k} \frac{\partial {\,\widehat{\!p\!}\,\,}^k}{\partial y^j}, \end{aligned}$$

and hence, inserting the above bounds,

$$\begin{aligned}&\left| \frac{\partial z_1^i}{\partial y^j} - \frac{t-s}{t-t_0} \delta ^i_j \right| \\&\quad = \left| \left( \frac{\partial X^i}{\partial {\,\widehat{\!p\!}\,\,}^k} + (t-s) \delta ^i_k \right) \left( \frac{\partial {\,\widehat{\!p\!}\,\,}^k}{\partial y^j} + \frac{\delta ^k_j}{t-t_0} \right) - (t-s) \left( \frac{\partial {\,\widehat{\!p\!}\,\,}^i}{\partial y^j} + \frac{\delta ^i_j}{t-t_0} \right) \right. \\&\qquad \left. -\frac{1}{t-t_0} \left( \frac{\partial X^i}{\partial {\,\widehat{\!p\!}\,\,}^j} + (t-s) \delta ^i_j \right) \right| \lesssim \varepsilon . \end{aligned}$$

It follows that

$$\begin{aligned} \left| \det \left( \frac{\partial z_1^i}{\partial y^j} \right) - \left( \frac{t-s}{t-t_0} \right) ^3 \right| \lesssim \varepsilon , \end{aligned}$$

and, if $\varepsilon$ is sufficiently small, the bound (5.6) follows for $t_0 \le s \le t_0 + \frac{1}{2}$. Similarly,

$$\begin{aligned} \frac{\partial z_1^i}{\partial x^j} = \frac{\partial X^i(s,t,x,{\,\widehat{\!p\!}\,\,}(t,x,y))}{\partial x^j} = \frac{\partial X^i}{\partial x^j} + \frac{\partial X^i}{\partial {\,\widehat{\!p\!}\,\,}^k} \frac{\partial {\,\widehat{\!p\!}\,\,}^k}{\partial x^j}, \end{aligned}$$

and

$$\begin{aligned}&\left| \frac{\partial z_1^i}{\partial x^j} - \frac{s-t_0}{t-t_0} \delta ^i_j \right| \\&\quad = \Bigg \vert \frac{\partial X^i}{\partial x^j} - \delta ^i_j + \left( \frac{\partial X^i}{\partial {\,\widehat{\!p\!}\,\,}^k} + (t-s) \delta ^i_k \right) \bigg ( \frac{\partial {\,\widehat{\!p\!}\,\,}^k}{\partial x^j} - \frac{\delta ^k_j}{t-t_0} \bigg ) \\&\qquad - (t-s) \left( \frac{\partial {\,\widehat{\!p\!}\,\,}^i}{\partial x^j} - \frac{\delta ^i_j}{t-t_0} \right) +\frac{1}{t-t_0} \left( \frac{\partial X^i}{\partial {\,\widehat{\!p\!}\,\,}^j} + (t-s) \delta ^i_j \right) \Bigg \vert \\&\quad \lesssim \varepsilon , \end{aligned}$$

which, if $\varepsilon$ is suitably small, implies

$$\begin{aligned} \left| \det \left( \frac{\partial z_1^i}{\partial x^j} \right) - \left( \frac{s-t_0}{t-t_0} \right) ^3 \right| \lesssim \varepsilon , \end{aligned}$$

and the bound (5.7) follows.

Finally,

$$\begin{aligned} \frac{\partial z_1^i}{\partial x^j} = \frac{\sigma s}{t} \delta ^i_j + (1 - \sigma ) \frac{\partial z_1^i}{\partial x^j}, \end{aligned}$$

and so

$$\begin{aligned} \left| \frac{\partial z_2^i}{\partial x^j} - \left( \sigma \frac{s}{t} + (1-\sigma ) \frac{s-t_0}{t-t_0} \right) \delta ^i_j \right| \lesssim \varepsilon , \end{aligned}$$

from which the bound (5.8) follows.

We now prove the bound (5.9). For $t_0 \le s \le t$ denote $W(s) = \left( X(s,t,x,{\,\widehat{\!p\!}\,\,}), {\,\widehat{\!P\!}\,\,}(s,t,x,{\,\widehat{\!p\!}\,\,}) \right)$ and $w = (x,{\,\widehat{\!p\!}\,\,})$, so that $\kappa = \frac{\partial W}{\partial w}$. Now

$$\begin{aligned}&\frac{\mathrm{d}}{\mathrm{d}s} W(s) =F\big (s,W(s)\big ),\\&\quad \text {where}\quad F\big (s,W\big )=\Big ({\,\widehat{\!P\!}\,\,}, \big ( {\,\widehat{\!P\!}\,\,}^i {\,\widehat{\!P\!}\,\,}^{\alpha } {\,\widehat{\!P\!}\,\,}^{\beta } \Gamma _{\alpha \beta }^0 (s,X) - {\,\widehat{\!P\!}\,\,}^{\alpha } {\,\widehat{\!P\!}\,\,}^{\beta } \Gamma _{\alpha \beta }^i (s,X) \big )\Big ), \end{aligned}$$

With $M=\partial W/\partial w$ we have

5.12 $$\begin{aligned}&\frac{\mathrm{d}}{\mathrm{d}s} \det M(s) = \text {tr}\left( M^{-1} \frac{dM}{\mathrm{d}s} (s) \right) \cdot \det M(s),\nonumber \\&\quad \text {where}\quad \text {tr}\left( M^{-1} \frac{dM}{\mathrm{d}s}\right) =\frac{\partial w^j}{\partial W^i}\frac{\partial F^i}{\partial w^j}=\frac{\partial F^i}{\partial W^i} . \end{aligned}$$

We have

$$\begin{aligned} \frac{\partial F^i}{\partial W^i} = 3 {\,\widehat{\!P\!}\,\,}^{\alpha } {\,\widehat{\!P\!}\,\,}^{\beta } \Gamma _{\alpha \beta }^0 (s,X) + 2 {\,\widehat{\!P\!}\,\,}^{i} {\,\widehat{\!P\!}\,\,}^{\beta } \Gamma _{i \beta }^0 (s,X) - 2 {\,\widehat{\!P\!}\,\,}^{\beta } \Gamma _{i \beta }^i (s,X), \end{aligned}$$

and so,

$$\begin{aligned} \left| \frac{\mathrm{d}}{\mathrm{d}s} \det \left( \frac{\partial W}{\partial w}(s) \right) \right| \le \frac{C\varepsilon }{s^{1+a}} \left| \det \left( \frac{\partial W}{\partial w}(s) \right) \right| . \end{aligned}$$

The bound (5.9) then follows from the Grönwall inequality. $\quad \square$

### $L^1$ and $L^2$ Estimates of Components of the Energy Momentum Tensor

The main part of the proof of Theorem 1.3 is contained in Propositions 5.8 and 5.9 below. The following Lemma will be used:

#### Lemma 5.7

Suppose $\pi (\mathrm {supp}(f_0)) \subset \{\vert x \vert \le K \}$, $t\ge t_0+1$ and the assumptions (4.1) hold. Then

$$\begin{aligned} \int \chi _{\mathrm {supp}(f)}(t,x,{\,\widehat{\!p\!}\,\,})\,\mathrm{d}{\,\widehat{\!p\!}\,\,}\lesssim \frac{1}{t^3}, \end{aligned}$$

where $\chi _{\mathrm {supp}(f)}(t,x,{\,\widehat{\!p\!}\,\,})$ is the characteristic function of $\mathrm {supp}(f)$.

#### Proof

Since *f* solves the Vlasov equation, $\chi _{\mathrm {supp}(f)}(t,x,{\,\widehat{\!p\!}\,\,}) = \chi _{\mathrm {supp}(f)}(t_0,X(t_0),{\,\widehat{\!P\!}\,\,}(t_0))$ and

$$\begin{aligned}&\int \chi _{\mathrm {supp}(f)}(t_0,X(t_0),{\,\widehat{\!P\!}\,\,}(t_0))\,\mathrm{d}{\,\widehat{\!p\!}\,\,}\\&\quad \le \frac{C}{t^3} \int \chi _{\mathrm {supp}(f)}(t_0,y,{\,\widehat{\!P\!}\,\,}(t_0,t,x,{\,\widehat{\!p\!}\,\,}(t,x,y)))\,\mathrm{d}y \le \frac{CK}{t^3}, \end{aligned}$$

where the change of variables ${\,\widehat{\!p\!}\,\,}\mapsto y= X(t_0)$ and the bound (5.5) have been used. $\quad \square$

#### Proposition 5.8

Suppose $\pi (\mathrm {supp}(f)) \subset \{\vert x \vert \le ct\}$ and consider $t\ge t_0+1$. If the assumptions (4.1) hold and $\varepsilon$ is sufficiently small then, for any multi index *I* with $\vert I \vert \le N-1$ and each $\mu , \nu = 0,1,2,3$,

$$\begin{aligned} \Vert Z^I T^{\mu \nu } (t,\cdot ) \Vert _{L^{2}} \le&\frac{C\mathcal {V}_{\vert I \vert }}{t^{\frac{3}{2}}} + C \mathbb {D}_{\left\lfloor \frac{\vert I \vert }{2} \right\rfloor +1} \left( \sum _{\vert J \vert \le \vert I \vert -1} \frac{\Vert ( Z^J \Gamma )(t,\cdot ) \Vert _{L^2}}{t^{1+a}} \right. \\&\left. + \sum _{\vert J \vert \le \vert I \vert + 1} \frac{1}{t^{\frac{3}{2}}} \int _{t_0}^t \frac{\Vert ( Z^J \Gamma )(s,\cdot ) \Vert _{L^2}}{s^{\frac{1}{2}+a}} \mathrm{d}s \right) ,\\ \Vert Z^I T^{\mu \nu } (t,\cdot ) \Vert _{L^{1}} \le&C\mathcal {V}_{\vert I \vert } + C \mathbb {D}_{\left\lfloor \frac{\vert I \vert }{2} \right\rfloor +1} \left( \sum _{\vert J \vert \le \vert I \vert -1} t^{\frac{1}{2} - a} \Vert ( Z^J \Gamma )(t,\cdot ) \Vert _{L^2} \right. \\&\left. + \sum _{\vert J \vert \le \vert I \vert + 1} \int _{t_0}^t \frac{\Vert ( Z^J \Gamma )(s,\cdot ) \Vert _{L^2}}{s^{\frac{1}{2}+a}} \,\mathrm{d}s \right) , \end{aligned}$$

where $Z^I$ is a product of $\vert I \vert$ of the vector fields $\Omega _{ij}, B_i, S$.

#### Proof

Given any function *F*(*t*, *x*) it follows from Proposition 5.4 that

$$\begin{aligned}&\Vert Z^I T^{\mu \nu } (t,\cdot ) F(t,\cdot ) \Vert _{L^1} \\&\quad \le C \sum _{n_1+n_2 \le \vert I \vert } \int _{\vert x \vert \le c t +K} \int \left| (\partial ^{n_1}_x \partial ^{n_2}_{{\,\widehat{\!p\!}\,\,}} f)(t_0,X(t_0),{\,\widehat{\!P\!}\,\,}(t_0)) \right| \,\mathrm{d}{\,\widehat{\!p\!}\,\,}\vert F(t,x) \vert \, \mathrm{d}x\\&\qquad + C \sum _{n_1+n_2 \le \left\lfloor \frac{\vert I \vert }{2} \right\rfloor + 1} \int _{\vert x \vert \le ct + K} \int \left| (\partial ^{n_1}_x \partial ^{n_2}_{{\,\widehat{\!p\!}\,\,}} f)(t_0,X(t_0),{\,\widehat{\!P\!}\,\,}(t_0)) \right| \\&\qquad \Bigg ( \sum _{\vert J \vert \le \vert I \vert -1} t^{2-a} \vert (Z^J \Gamma )(t,x) \vert \\&\qquad + \sum _{\vert J \vert \le \vert I \vert } \int _{t_0}^t s^{1-a} \left| (Z^J \Gamma ) \left( s, s\frac{x}{t} \right) \right| + s \left| (Z^J \Gamma ) \left( s, s\frac{x}{t} \right) \right. \\&\qquad \left. - (Z^J \Gamma ) \left( s, X(s) \right) \right| \, \mathrm{d}s \Bigg ) \,\mathrm{d}{\,\widehat{\!p\!}\,\,}\vert F(t,x) \vert \, \mathrm{d}x. \end{aligned}$$

Given $n_1+n_2 \le \vert I \vert$, it follows from Lemma 5.7 and the bound (5.9) that

$$\begin{aligned}&\int _{\vert x \vert \le c t +K} \int \left| (\partial ^{n_1}_x \partial ^{n_2}_{{\,\widehat{\!p\!}\,\,}} f)(t_0,X(t_0),{\,\widehat{\!P\!}\,\,}(t_0)) \right| \,\mathrm{d}{\,\widehat{\!p\!}\,\,}\vert F(t,x) \vert \mathrm{d}x\\&\quad \le \int \left( \int \chi _{\mathrm {supp}(f)}(t,x,{\,\widehat{\!p\!}\,\,}) \,\mathrm{d}{\,\widehat{\!p\!}\,\,}\right) ^{\frac{1}{2}} \\&\qquad \left( \int \left| (\partial ^{n_1}_x \partial ^{n_2}_{{\,\widehat{\!p\!}\,\,}} f)(t_0,X(t_0),{\,\widehat{\!P\!}\,\,}(t_0)) \right| ^2 \,\mathrm{d}{\,\widehat{\!p\!}\,\,}\right) ^{\frac{1}{2}} \vert F(t,x) \vert \mathrm{d}x\\&\quad \le \frac{C}{t^{\frac{3}{2}}} \left( \int \int \left| (\partial ^{n_1}_x \partial ^{n_2}_{{\,\widehat{\!p\!}\,\,}} f)(t_0,X(t_0),{\,\widehat{\!P\!}\,\,}(t_0)) \right| ^2 \,\mathrm{d}{\,\widehat{\!p\!}\,\,}\mathrm{d}x \right) ^{\frac{1}{2}} \Vert F(t,\cdot ) \Vert _{L^2} \\&\quad \le \frac{C \mathcal {V}_{n_1+n_2}}{t^{\frac{3}{2}}} \Vert F(t,\cdot ) \Vert _{L^2}. \end{aligned}$$

Similarly, for any multi index *J*,

$$\begin{aligned}&\int _{\vert x \vert \le c t +K} \int \left| (\partial ^{n_1}_x \partial ^{n_2}_{{\,\widehat{\!p\!}\,\,}} f)(t_0,X(t_0),{\,\widehat{\!P\!}\,\,}(t_0)) \right| \,\mathrm{d}{\,\widehat{\!p\!}\,\,}t^{2-a} \left| (Z^J \Gamma )(t,x) \right| \vert F(t,x) \vert \,\mathrm{d}x\\&\quad \le \int _{\vert x \vert \le c t +K} \int _{\vert y \vert \le K} \left| (\partial ^{n_1}_x \partial ^{n_2}_{{\,\widehat{\!p\!}\,\,}} f) (t_0,y,{\,\widehat{\!P\!}\,\,}(t_0,t,x,{\,\widehat{\!p\!}\,\,}(t,x,y))) \right| \\&\qquad \left| \det \left( \frac{\partial {\,\widehat{\!p\!}\,\,}^i}{\partial y^j} \right) \right| \,\mathrm{d}y t^{2-a} \left| (Z^J \Gamma )(t,x) \right| \vert F(t,x) \vert \mathrm{d}x\\&\quad \le \frac{C \mathbb {D}_{n_1+n_2}}{t^3} t^{2-a} \int _{\vert x \vert \le ct + K} \left| (Z^J \Gamma )(t,x) \right| \vert F(t,x) \vert \mathrm{d}x \\&\quad \le \frac{C \mathbb {D}_{n_1+n_2}}{t^{1+a}} \Vert (Z^J \Gamma )(t,\cdot ) \Vert _{L^2} \Vert F(t,\cdot ) \Vert _{L^2}, \end{aligned}$$

using the bound (5.5). Now, for the third term,

$$\begin{aligned}&\int _{\vert x \vert \le c t +K} \int \left| (\partial ^{n_1}_x \partial ^{n_2}_{{\,\widehat{\!p\!}\,\,}} f)(t_0,X(t_0),{\,\widehat{\!P\!}\,\,}(t_0)) \right| \,\mathrm{d}{\,\widehat{\!p\!}\,\,}\int _{t_0}^t s^{1-a} \left| (Z^J \Gamma ) \left( s, s\frac{x}{t} \right) \right| \,\mathrm{d}s \vert F(t,x) \vert \,\mathrm{d}x\\&\quad \le \frac{C \mathbb {D}_{n_1+n_2}}{t^3} \int _{t_0}^t s^{1-a} \int _{\vert y \vert \le K} \int _{\vert x \vert \le c t +K} \left| (Z^J \Gamma ) \left( s, s\frac{x}{t} \right) \right| \vert F(t,x) \vert \,\mathrm{d}x \mathrm{d}y \mathrm{d}s\\&\quad \le \frac{C \mathbb {D}_{n_1+n_2}}{t^3} \int _{t_0}^t s^{1-a} \left( \int _{\vert x \vert \le c t +K} \left| (Z^J \Gamma ) \left( s, s\frac{x}{t} \right) \right| ^2 \,\mathrm{d}x \right) ^{\frac{1}{2}} \Vert F(t,\cdot ) \Vert _{L^2} \,\mathrm{d}s\\&\quad \le \frac{C \mathbb {D}_{n_1+n_2}}{t^{\frac{3}{2}}} \int _{t_0}^t \frac{ \left\| (Z^J \Gamma ) \left( s, \cdot \right) \right\| _{L^2} }{ s^{\frac{1}{2}+a} }\, \mathrm{d}s \Vert F(t,\cdot ) \Vert _{L^2}, \end{aligned}$$

where the change of variables $x^i \mapsto z^i := s \frac{x^i}{t}$ has been used, along with the fact that $\det \left( \frac{\partial x^i}{\partial z^j} \right) = \left( \frac{t}{s} \right) ^3$.

For the final term, first write,

$$\begin{aligned}&\sum _{\vert J \vert \le \vert I\vert } \int _{t_0}^t s \left| (Z^J \Gamma ) \left( s, s\frac{x}{t} \right) - (Z^J \Gamma ) \left( s, X(s) \right) \right| \,\mathrm{d}s\\&\quad \le \sum _{\vert J \vert \le \vert I\vert } \int _{t_0}^{t_0+\frac{1}{2}}\!\!\!\! s \left| (Z^J \Gamma ) \left( s, s\frac{x}{t} \right) \right| + s \left| (Z^J \Gamma ) \left( s, X(s) \right) \right| \,\mathrm{d}s \\&\qquad + \sum _{\vert J \vert \le \vert I\vert } \int _{t_0+\frac{1}{2}}^t\!\!\!\! s \left| (Z^J \Gamma ) \left( s, s\frac{x}{t} \right) - (Z^J \Gamma ) \left( s, X(s) \right) \right| \,\mathrm{d}s. \end{aligned}$$

As above,

$$\begin{aligned}&\int _{\vert x \vert \le c t +K} \int \left| (\partial ^{n_1}_x \partial ^{n_2}_{{\,\widehat{\!p\!}\,\,}} f)(t_0,X(t_0),{\,\widehat{\!P\!}\,\,}(t_0)) \right| \,\mathrm{d}{\,\widehat{\!p\!}\,\,}\int _{t_0}^{t_0+\frac{1}{2}} s \left| (Z^J \Gamma ) \left( s, s\frac{x}{t} \right) \right| \mathrm{d}s \vert F(t,x) \vert \,\mathrm{d}x\\&\quad \le \frac{C \mathbb {D}_{n_1+n_2}}{t^{\frac{3}{2}}} \int _{t_0}^{t_0+\frac{1}{2}} \left\| (Z^J \Gamma ) \left( s, \cdot \right) \right\| _{L^2} \mathrm{d}s \Vert F(t,\cdot ) \Vert _{L^2} \\&\quad \le \frac{C \mathbb {D}_{n_1+n_2}}{t^{\frac{3}{2}}} \int _{t_0}^t \frac{ \left\| (Z^J \Gamma ) \left( s, \cdot \right) \right\| _{L^2} }{ s^{\frac{1}{2}+a} } \,\mathrm{d}s \Vert F(t,\cdot ) \Vert _{L^2}, \end{aligned}$$

and, estimating the term with $X(s,t,x,{\,\widehat{\!p\!}\,\,})$ slightly differently,

$$\begin{aligned}&\int _{\vert x \vert \le c t +K} \int \left| (\partial ^{n_1}_x \partial ^{n_2}_{{\,\widehat{\!p\!}\,\,}} f)(t_0,X(t_0),{\,\widehat{\!P\!}\,\,}(t_0)) \right| \int _{t_0}^{t_0+\frac{1}{2}} s \left| (Z^J \Gamma ) \left( s, X(s) \right) \right| \,\mathrm{d}s \mathrm{d}{\,\widehat{\!p\!}\,\,}\vert F(t,x) \vert \,\mathrm{d}x\\&\quad \le \frac{C \mathbb {D}_{n_1+n_2}}{t^3} \int _{t_0}^{t_0+\frac{1}{2}} \int _{\vert x \vert \le c t +K} \vert F(t,x) \vert \int _{\vert y \vert \le K} \left| (Z^J \Gamma ) \left( s, X(s) \right) \right| \,\mathrm{d}y \mathrm{d}x \mathrm{d}s\\&\quad \le \frac{C \mathbb {D}_{n_1+n_2}}{t^3} \int _{t_0}^{t_0+\frac{1}{2}} \int _{\vert x \vert \le c t +K} \vert F(t,x) \vert \left( \int _{\vert y \vert \le K} \left| (Z^J \Gamma ) \left( s, X(s) \right) \right| ^2 \mathrm{d}y \right) ^{\frac{1}{2}} \mathrm{d}x \mathrm{d}s\\&\quad \le \frac{C \mathbb {D}_{n_1+n_2}}{t^{\frac{3}{2}}} \int _{t_0}^{t_0+\frac{1}{2}} \left\| (Z^J \Gamma ) \left( s, \cdot \right) \right\| _{L^2} \,\mathrm{d}s \Vert F(t,\cdot ) \Vert _{L^2} \\&\quad \le \frac{C \mathbb {D}_{n_1+n_2}}{t^{\frac{3}{2}}} \int _{t_0}^{t} \frac{\left\| (Z^J \Gamma ) \left( s, \cdot \right) \right\| _{L^2}}{s^{\frac{1}{2}+a}} \,\mathrm{d}s \Vert F(t,\cdot ) \Vert _{L^2}, \end{aligned}$$

where now the change of variables $y^i \mapsto z_1^i := X(s,t,x,{\,\widehat{\!p\!}\,\,}(t,x,y))^i$ has been used, together with Proposition 5.6, which guarantees that $\left| \det \left( \frac{\partial y^i}{\partial z_1^j} \right) \right| \le C$ when $t_0\le s \le t_0+\frac{1}{2}$. Note that this term was estimated slightly differently since the change of variables $x^i \mapsto X(s,t,x,{\,\widehat{\!p\!}\,\,}(t,x,y))^i$ breaks down as $s\rightarrow t_0$, since then $X(s,t,x,{\,\widehat{\!p\!}\,\,}(t,x,y))\rightarrow y$.

Finally, write

$$\begin{aligned}&Z^J \Gamma \left( s, s\frac{x}{t} \right) - Z^J \Gamma \left( s, X(s) \right) \\&\quad = \int _0^1 \frac{\mathrm{d}}{d\sigma } \left( Z^J \Gamma \left( s, \sigma s\frac{x}{t} + (1-\sigma ) X(s) \right) \right) d\sigma \\&\quad = \left( s \frac{x^l}{t} - X(s)^l \right) \int _0^1 \left( \partial _{x^l} Z^J \Gamma \right) \left( s, \sigma s\frac{x}{t} + (1-\sigma ) X(s) \right) d\sigma . \end{aligned}$$

Since,

$$\begin{aligned} \left| s \frac{x^l}{t} - X(s)^l \right| \le \left| s \frac{x^l}{t} - X_2(s,t,x,{\,\widehat{\!p\!}\,\,})^l \right| + \left| \overline{X}(s,t,x,{\,\widehat{\!p\!}\,\,})^l \right| \le Cs^{1-a}, \end{aligned}$$

by Proposition 2.3 and the bound (4.26) with $I=0$, it follows that

$$\begin{aligned}&\sum _{\vert J \vert \le \vert I \vert } s \left| Z^J \Gamma \left( s, s\frac{x}{t} \right) - Z^J \Gamma \left( s, X(s) \right) \right| \\&\quad \le C \sum _{\vert J \vert \le \vert I \vert } s^{1-a} \int _0^1 \left| s \left( \partial Z^J \Gamma \right) \left( s, \sigma s\frac{x}{t} + (1-\sigma ) X(s) \right) \right| d\sigma \\&\quad \le C \sum _{\vert J \vert \le \vert I \vert + 1} s^{1-a} \int _0^1 \left| \left( Z^J \Gamma \right) \left( s, \sigma s\frac{x}{t} + (1-\sigma ) X(s) \right) \right| d\sigma . \end{aligned}$$

Hence,

$$\begin{aligned}&\int _{\vert x \vert \le ct + K} \int \left| (\partial ^{n_1}_x \partial ^{n_2}_{{\,\widehat{\!p\!}\,\,}} f)(t_0,X(t_0),{\,\widehat{\!P\!}\,\,}(t_0)) \right| \\&\qquad \times \sum _{\vert J \vert \le \vert I \vert } \int _{t_0+ \frac{1}{2}}^t s \left| (Z^J \Gamma ) \left( s, s\frac{x}{t} \right) - (Z^J \Gamma ) \left( s, X(s) \right) \right| \, \mathrm{d}s \mathrm{d}{\,\widehat{\!p\!}\,\,}\vert F(t,x) \vert \,\mathrm{d}x\\&\quad \le \frac{C\mathbb {D}_{n_1+n_2}}{t^3} \sum _{\vert J \vert \le \vert I \vert + 1} \int _{\vert x \vert \le ct + K} \int _{\vert y \vert \le K} \int _{t_0+\frac{1}{2}}^t s^{1-a} \int _0^1 \\&\qquad \left| \left( Z^J \Gamma \right) \left( s, \sigma s\frac{x}{t} + (1-\sigma ) X(s) \right) \right| \mathrm{d}\sigma \,\mathrm{d}s \mathrm{d}y \vert F(t,x) \vert \mathrm{d}x\\&\quad \le \frac{C\mathbb {D}_{n_1+n_2}}{t^3}\!\!\!\!\! \sum _{\vert J \vert \le \vert I \vert + 1} \int _{t_0+\frac{1}{2}}^t\!\!\!\!\!\! s^{1-a} \int _0^1 \int _{\vert y \vert \le K} \\&\qquad \Big ( \int _{\vert x \vert \le ct + K} \left| \left( Z^J \Gamma \right) \left( s, \sigma s\frac{x}{t} + (1-\sigma ) X(s) \right) \right| ^2\!\! \mathrm{d}x \Big )^{\frac{1}{2}} \mathrm{d}y \mathrm{d}\sigma \mathrm{d}s\, \Vert F(t,\cdot ) \Vert _{L^2}\\&\quad \le \frac{C\mathbb {D}_{n_1+n_2}}{t^{\frac{3}{2}}} \sum _{\vert J \vert \le \vert I \vert + 1} \int _{t_0}^t \frac{\left\| \left( Z^J \Gamma \right) \left( s, \cdot \right) \right\| _{L^2}}{s^{\frac{1}{2} + a}} \mathrm{d}s \Vert F(t,\cdot ) \Vert _{L^2}, \end{aligned}$$

where the change of variables $x^i \mapsto z_2^i := \sigma s \frac{x^i}{t} + (1-\sigma )X(s)^i$ has been used, along with the fact that $\left| \det \left( \frac{\partial x^i}{\partial z_2^j} \right) \right| \le C \left( \frac{t}{s} \right) ^3$ when $0 \le \sigma \le 1$, $t_0 + \frac{1}{2} \le s \le t$, by Proposition 5.6.

It follows that, for any *F*(*t*, *x*),

$$\begin{aligned}&\Vert \left( Z^I T^{\mu \nu } \right) (t,\cdot ) F(t, \cdot ) \Vert _{L^{1}} \le \frac{C\mathcal {V}_{\vert I \vert } \Vert F(t,\cdot ) \Vert _{L^2}}{t^{\frac{3}{2}}}\\&\quad + C \mathbb {D}_{\left\lfloor \frac{\vert I \vert }{2} \right\rfloor +1} \Vert F(t,\cdot ) \Vert _{L^2} \left( \sum _{\vert J \vert \le \vert I \vert -1} \frac{\Vert ( Z^J \Gamma )(t,\cdot ) \Vert _{L^2}}{t^{1+a}} \right. \\&\quad \left. + \sum _{\vert J \vert \le \vert I \vert + 1} \frac{1}{t^{\frac{3}{2}}} \int _{t_0}^t \frac{\Vert ( Z^J \Gamma )(s,\cdot ) \Vert _{L^2}}{s^{\frac{1}{2}+a}} \mathrm{d}s \right) . \end{aligned}$$

The $L^2$ estimate follows by setting $F(t,x) = Z^I T^{\mu \nu }(t,x)$ and dividing by $\Vert Z^I T^{\mu \nu } (t,\cdot ) \Vert _{L^2}$. The $L^1$ estimate follows by setting $F(t,x) = \chi _{\{ \vert x \vert \le c t + K \}}$, and using the fact that $\mathrm {supp}(T^{\mu \nu }) \subset \{ \vert x \vert \le c t + K \}$, and $\Vert \chi _{\{ \vert x \vert \le c t + K \}} \Vert _{L^2} \le C t^{\frac{3}{2}}$. $\quad \square$

#### Proposition 5.9

Suppose $\pi (\mathrm {supp}(f)) \subset \{\vert x \vert \le ct\}$ and consider $t\ge t_0+1$. If the assumptions (4.1) hold, and $\varepsilon$ is sufficiently small, then for any multi index *I* with $\vert I \vert \le N$ and each $\mu ,\nu = 0,1,2,3$,

$$\begin{aligned} \Vert \left( Z^I T^{\mu \nu } \right) (t,\cdot ) \Vert _{L^{2}} \le&\frac{C\mathcal {V}_{\vert I \vert }}{t^{\frac{3}{2}}} + C \mathbb {D}_{\left\lfloor \frac{\vert I \vert }{2} \right\rfloor +1} \left( \sum _{\vert J \vert \le \vert I \vert -1} \frac{\Vert ( Z^J \Gamma )(t,\cdot ) \Vert _{L^2}}{t^{1+a}}\right. \\&\left. + \sum _{\vert J \vert \le \vert I \vert } \frac{1}{t^{\frac{3}{2}}} \int _{t_0}^t \frac{\Vert ( Z^J \Gamma )(s,\cdot ) \Vert _{L^2}}{s^{\frac{1}{2}}} \,\mathrm{d}s \right) , \end{aligned}$$

where $Z^I$ is a product of $\vert I \vert$ of the vector fields $\Omega _{ij}, B_i, S$.

#### Proof

The proof is very similar to that of Proposition 5.8. Recall that, for any *F*(*t*, *x*),

$$\begin{aligned}&\Vert \left( Z^I T^{\mu \nu } \right) (t,\cdot ) F(t, \cdot ) \Vert _{L^{1}} \le \frac{C\mathcal {V}_{\vert I \vert } \Vert F(t,\cdot ) \Vert _{L^2}}{t^{\frac{3}{2}}}\\&\quad + C \mathbb {D}_{\left\lfloor \frac{\vert I \vert }{2} \right\rfloor +1} \Vert F(t,\cdot ) \Vert _{L^2} \left( \sum _{\vert J \vert \le \vert I \vert -1} \frac{\Vert ( Z^J \Gamma )(t,\cdot ) \Vert _{L^2}}{t^{1+a}} \right. \\&\left. \quad + \sum _{\vert J \vert \le \vert I \vert } \frac{1}{t^{\frac{3}{2}}} \int _{t_0}^t \frac{\Vert ( Z^J \Gamma )(s,\cdot ) \Vert _{L^2}}{s^{\frac{1}{2}+a}} \,\mathrm{d}s \right) \\&\quad + C \sum _{n_1+n_2 \le \left\lfloor \frac{\vert I \vert }{2} \right\rfloor +1} \sum _{\vert J \vert \le \vert I \vert } \int _{\vert x \vert \le ct + K} \int \left| (\partial ^{n_1}_x \partial ^{n_2}_{{\,\widehat{\!p\!}\,\,}} f)(t_0,X(t_0),{\,\widehat{\!P\!}\,\,}(t_0)) \right| \\&\quad \times \int _{t_0+\frac{1}{2}}^t s \left| (Z^J \Gamma ) \left( s, s\frac{x}{t} \right) - (Z^J \Gamma ) \left( s, X(s) \right) \right| \mathrm{d}s \,\mathrm{d}{\,\widehat{\!p\!}\,\,}\vert F(t,x) \vert \, \mathrm{d}x. \end{aligned}$$

It is only the final term which is estimated differently. In Proposition 5.8 an extra derivative of $\Gamma$ was used to exploit the cancellation in $(Z^J \Gamma ) \left( s, s\frac{x}{t} \right) - (Z^J \Gamma ) \left( s, X(s) \right)$. Now, at the top order, these terms are estimated individually:

$$\begin{aligned}&\sum _{\vert J \vert \le \vert I \vert } \int _{t_0+\frac{1}{2}}^t s \left| (Z^J \Gamma ) \left( s, s\frac{x}{t} \right) - (Z^J \Gamma ) \left( s, X(s) \right) \right| \,\mathrm{d}s \\&\quad \le C\!\!\! \sum _{\vert J \vert \le \vert I \vert } \int _{t_0+\frac{1}{2}}^t s \left| (Z^J \Gamma ) \left( s, s\frac{x}{t} \right) \right| + s \left| (Z^J \Gamma ) \left( s, X(s) \right) \right| \, \mathrm{d}s. \end{aligned}$$

The first term is estimated exactly as in Proposition 5.8 (note that the *s* power is now slightly worse) to give

$$\begin{aligned}&\int _{\vert x \vert \le ct + K} \int \left| (\partial ^{n_1}_x \partial ^{n_2}_{{\,\widehat{\!p\!}\,\,}} f)(t_0,X(t_0),{\,\widehat{\!P\!}\,\,}(t_0)) \right| \\&\int _{t_0+\frac{1}{2}}^t s \left| (Z^J \Gamma ) \left( s, s\frac{x}{t} \right) \right| \,\mathrm{d}s \,\mathrm{d}{\,\widehat{\!p\!}\,\,}\vert F(t,x) \vert \mathrm{d}x\\&\quad \le \frac{C\mathbb {D}_{n_1+n_2}}{t^{{3}/{2}}} \Vert F(t,\cdot ) \Vert _{L^2} \int _{t_0}^t \frac{\Vert (Z^J \Gamma )(s,\cdot )\Vert _{L^2}}{s^{{1}/{2}}} \,\mathrm{d}s. \end{aligned}$$

The second term is estimated similarly:

$$\begin{aligned}&\int _{\vert x \vert \le ct + K} \int \left| (\partial ^{n_1}_x \partial ^{n_2}_{{\,\widehat{\!p\!}\,\,}} f)(t_0,X(t_0),{\,\widehat{\!P\!}\,\,}(t_0)) \right| \int _{t_0+\frac{1}{2}}^t s \left| (Z^J \Gamma ) \left( s, X(s) \right) \right| \,\mathrm{d}s \mathrm{d}{\,\widehat{\!p\!}\,\,}\vert F(t,x) \vert \mathrm{d}x\\&\quad \le \frac{C\mathbb {D}_{n_1+n_2}}{t^3} \int _{t_0+\frac{1}{2}}^t s \int _{\vert y \vert \le K} \Big ( \int _{\vert x \vert \le ct +K} \left| (Z^J \Gamma ) \left( s, X(s) \right) \right| ^2 \mathrm{d}x \Big )^{\frac{1}{2}} \mathrm{d}y \mathrm{d}s \Vert F(t,\cdot ) \Vert _{L^2}\\&\quad \le \frac{C\mathbb {D}_{n_1+n_2}}{t^{{3}/{2}}} \Vert F(t,\cdot ) \Vert _{L^2} \int _{t_0}^t \frac{\Vert (Z^J \Gamma )(s,\cdot )\Vert _{L^2}}{s^{\frac{1}{2}}}\, \mathrm{d}s, \end{aligned}$$

where the change of variables $x^i \mapsto z_1^i := X(s,t,x,{\,\widehat{\!p\!}\,\,}(t,x,y))^i$ has now been used, along with the fact that $\vert \det ( {\partial x^i}/{\partial z_1^j} ) \vert \le C ( {t}/{s} )^3$ for $t_0 + \frac{1}{2} \le s \le t$, by Proposition 5.6.

It follows that

$$\begin{aligned}&\Vert \left( Z^I T^{\mu \nu } \right) (t,\cdot ) F(t, \cdot ) \Vert _{L^{1}} \le \frac{C\mathcal {V}_{\vert I \vert } \Vert F(t,\cdot ) \Vert _{L^2}}{t^{\frac{3}{2}}}\\&\quad + C \mathbb {D}_{\left\lfloor \frac{\vert I \vert }{2} \right\rfloor +1} \Vert F(t,\cdot ) \Vert _{L^2} \Big ( \sum _{\vert J \vert \le \vert I \vert -1} \frac{\Vert ( Z^J \Gamma )(t,\cdot ) \Vert _{L^2}}{t^{1+a}} \\&\qquad + \sum _{\vert J \vert \le \vert I \vert } \frac{1}{t^{\frac{3}{2}}} \int _{t_0}^t \frac{\Vert ( Z^J \Gamma )(s,\cdot ) \Vert _{L^2}}{s^{\frac{1}{2}}} \,\mathrm{d}s \Big ). \end{aligned}$$

The proof then follows by setting $F(t,x) = Z^IT^{\mu \nu }(t,x)$. $\quad \square$

### Proof of Theorem 1.3

First note that Propositions 5.8 and 5.9 can be extended to include $t_0 \le t \le t_0 +1$ as follows:

#### Proposition 5.10

Suppose $\pi \left( \mathrm {supp}(f) \right) \subset \{ \vert x \vert \le c t\}$ and consider $t_0 \le t \le t_0 +1$. If the assumptions (4.1) hold and $\varepsilon$ is sufficiently small then, for any multi index *I* with $\vert I \vert \le N$,

$$\begin{aligned} \Vert \partial ^I T^{\mu \nu }( t,\cdot ) \Vert _{L^2} + \Vert \partial ^I T^{\mu \nu }( t,\cdot ) \Vert _{L^1}\lesssim & {} \mathcal {V}_{\vert I \vert } + \mathbb {D}_{\left\lfloor \frac{\vert I \vert }{2} \right\rfloor + 1} \Big ( \sum _{\vert J \vert \le \vert I \vert -1} \Vert \partial ^J \Gamma (t,\cdot ) \Vert _{L^2} \\&+ \sum _{\vert J \vert \le \vert I \vert } \int _{t_0}^t \Vert \partial ^J \Gamma (s,\cdot ) \Vert _{L^2} \,\mathrm{d}s \Big ). \end{aligned}$$

#### Proof

By Propositions 4.30 and 4.31, it follows from an appropriate version of Proposition 5.4 that

$$\begin{aligned} \left| \partial ^I T^{\mu \nu } (t,x) \right| \lesssim&\sum _{n_1+n_2 \le \vert I \vert } \int \left| (\partial _x^{n_1} \partial _{{\,\widehat{\!p\!}\,\,}}^{n_2} f)(t_0,X(t_0),{\,\widehat{\!P\!}\,\,}(t_0)) \right| \,\mathrm{d}{\,\widehat{\!p\!}\,\,}\\&+ \sum _{n_1+n_2 \le \left\lfloor \frac{\vert I \vert }{2} \right\rfloor + 1} \int \left| (\partial _x^{n_1} \partial _{{\,\widehat{\!p\!}\,\,}}^{n_2} f)(t_0,X(t_0),{\,\widehat{\!P\!}\,\,}(t_0)) \right| \\&\Big ( \sum _{\vert J \vert \le \vert I \vert -1} \left| (\partial ^J \Gamma )(t,x) \right| + \sum _{\vert J \vert \le \vert I \vert } \int _{t_0}^t \left| (\partial ^J \Gamma )(s,X(s)) \right| \, \mathrm{d}s \Big ) \,\mathrm{d}{\,\widehat{\!p\!}\,\,}\\ \lesssim&\sum _{n_1+n_2 \le \vert I \vert } \int \left| (\partial _x^{n_1} \partial _{{\,\widehat{\!p\!}\,\,}}^{n_2} f)(t_0,X(t_0),{\,\widehat{\!P\!}\,\,}(t_0)) \right| \,\mathrm{d}{\,\widehat{\!p\!}\,\,}\\&+ \mathbb {D}_{\left\lfloor \frac{\vert I \vert }{2} \right\rfloor + 1} \Big ( \sum _{\vert J \vert \le \vert I \vert -1} \left| (\partial ^J \Gamma )(t,x) \right| \\&+ \sum _{\vert J \vert \le \vert I \vert } \int \int _{t_0}^t \left| (\partial ^J \Gamma )(s,X(s)) \right| \, \mathrm{d}s \mathrm{d}{\,\widehat{\!p\!}\,\,}\Big ). \end{aligned}$$

For any function *F*(*t*, *x*),

$$\begin{aligned} \int \int \left| (\partial _x^{n_1} \partial _{{\,\widehat{\!p\!}\,\,}}^{n_2} f)(t_0,X(t_0),{\,\widehat{\!P\!}\,\,}(t_0)) \right| \,\mathrm{d}{\,\widehat{\!p\!}\,\,}\vert F(t,x) \vert \,\mathrm{d}x \lesssim \mathcal {V}_{n_1+n_2} \Vert F(t,\cdot ) \Vert _{L^2}, \end{aligned}$$

by (5.9) (as in the proof of Proposition 5.8), and

$$\begin{aligned}&\int \vert F(t,x) \vert \int \int _{t_0}^t \left| (\partial ^J \Gamma )(s,X(s)) \right| \, \mathrm{d}s\, \mathrm{d} {\,\widehat{\!p\!}\,\,}\mathrm{d}x \\&\quad \le \int _{t_0}^t \int \left( \int \left| (\partial ^J \Gamma )(s,X(s)) \right| ^2 \,\mathrm{d}x \right) ^{\frac{1}{2}} \Vert F(t,\cdot )\Vert _{L^2} \,\mathrm{d}{\,\widehat{\!p\!}\,\,}\mathrm{d}s\\&\quad \lesssim \int _{t_0}^t \Vert (\partial ^J \Gamma )(s,\cdot ) \Vert _{L^2} \,\mathrm{d}s \Vert F(t,\cdot )\Vert _{L^2}, \end{aligned}$$

where the change of variables $x \mapsto X(s,t,x,{\,\widehat{\!p\!}\,\,})$ and the bound (5.3) have been used. Clearly then

$$\begin{aligned}&\int \left| \partial ^I T^{\mu \nu } (t,x) \right| \vert F(t,x) \vert \,\mathrm{d}x \lesssim \mathcal {V}_{\vert I \vert } \Vert F(t,\cdot ) \Vert _{L^2}\\&\qquad + \mathbb {D}_{\left\lfloor \frac{\vert I \vert }{2} \right\rfloor + 1} \int \vert F(t,x) \vert \Big ( \sum _{\vert J \vert \le \vert I \vert -1} \left| (\partial ^J \Gamma )(t,x) \right| \\&\qquad + \sum _{\vert J \vert \le \vert I \vert } \int \int _{t_0}^t \left| (\partial ^J \Gamma )(s,X(s)) \right| \mathrm{d}s\,\mathrm{d}{\,\widehat{\!p\!}\,\,}\Big ) \mathrm{d}x\\&\quad \lesssim \Big [ \mathcal {V}_{\vert I \vert } + \mathbb {D}_{\left\lfloor \frac{\vert I \vert }{2} \right\rfloor + 1} \Big ( \sum _{\vert J \vert \le \vert I \vert -1} \left\| (\partial ^J \Gamma )(t,\cdot ) \right\| _{L^2} \\&\qquad + \sum _{\vert J \vert \le \vert I \vert } \int _{t_0}^t \left\| (\partial ^J \Gamma )(s,\cdot ) \right\| \,\mathrm{d}s \Big ) \Big ] \Vert F(t,\cdot ) \Vert _{L^2}. \end{aligned}$$

The $L^2$ bound then follows by setting $F = \partial ^I T^{\mu \nu }$, and the $L^1$ bound follows by setting $F(t,x) = \chi _{\{ \vert x \vert \le c t + K \}}$, and using the fact that $\mathrm {supp}(T^{\mu \nu }) \subset \{ \vert x \vert \le c t + K \}$, and $\Vert \chi _{\{ \vert x \vert \le c t + K \}} \Vert _{L^2} \le C t^{\frac{3}{2}}$. $\quad \square$

Since, for any function *F*(*t*, *x*) and any multi index *I*, the vector fields *Z* satisfy

$$\begin{aligned} \sum _{\vert J \vert \le \vert I \vert } \vert \partial ^I F(t,x) \vert \lesssim \vert Z^I F(t,x) \vert \lesssim \sum _{\vert J \vert \le \vert I \vert } \vert \partial ^I F(t,x) \vert , \quad \text {for } t_0 \le t \le t_0 + 1, \vert x \vert \le c t, \end{aligned}$$

it is clear that Propositions 5.8 and 5.9 in fact hold for $t \ge t_0$. Moreover, it is clear from (2.12) that

$$\begin{aligned} \Vert \partial ^I Z^J T^{\mu \nu }(t,\cdot ) \Vert \lesssim \sum _{\vert K \vert \le \vert I \vert + \vert J \vert } \Vert Z^K T^{\mu \nu }(t,\cdot ) \Vert , \qquad \text {for } t \ge t_0, \end{aligned}$$

where $Z^J$ is a product of $\vert J \vert$ of the vector fields $\Omega _{ij}, B_i, S$, for $\Vert \cdot \Vert = \Vert \cdot \Vert _{L^1}$ or $\Vert \cdot \Vert _{L^2}$ since $\mathrm {supp}(T^{\mu \nu }) \subset \{ \vert x \vert \le ct\}$, and so spacetime derivatives $\partial ^I$ can be included in Propositions 5.8 and 5.9.

Suppose now that the assumptions of Theorem 1.3 hold. It follows from Proposition 2.1 that the support of *f* satisfies $\pi (\mathrm {supp}(f)) \subset \{ \vert x \vert \le ct + K\}$ and so, letting $\tilde{t} = t_0 + t$ where $t_0 = \frac{K}{c}$, it follows from Proposition 5.8, Proposition 5.9 and the above comments that the bounds of Theorem 1.3 hold with *t* replaced by $\tilde{t}$ and the vector fields *Z* replaced by $\tilde{Z}$ for $\tilde{t} \ge t_0$, where the vector fields $\tilde{Z}$ are as in Section 2.2. The proof of Theorem 1.3 then follows from noting that

$$\begin{aligned} \tilde{\Omega }_{ij} = \Omega _{ij}, \qquad \tilde{B}_i = B_i + t_0 \partial _{x^i}, \qquad \tilde{S} = S + t_0 \partial _t, \end{aligned}$$

and so

$$\begin{aligned} \partial ^I Z^J = \sum _{\vert I'\vert + \vert J'\vert \le \vert I \vert + \vert J \vert } C_{I'J''} \partial ^{I'} \tilde{Z}^{J'} \end{aligned}$$

for some constants $C_{I'J'}$.

## The Einstein Equations

The main results of this section are the following:

### Proposition 6.1

Suppose that $N\ge 4$, $\varepsilon \le 1$. Consider a solution of the reduced Einstein equations (1.11) for $t<T_*$ such that, with $E_N$ and $\gamma$ as in (1.14), the weak decay estimates

6.1 $$\begin{aligned} E_N(t)^{1/2} \le C_N \varepsilon (1+t)^{\delta }, \qquad \sum _{\vert J \vert \le N-1} \Vert Z^J \widehat{T}(t,\cdot ) \Vert _{L^1} \le C_N \varepsilon ,\qquad \text {and}\quad M\le \varepsilon \end{aligned}$$

hold for all $t\in [0,T_*]$ for some $\delta$ such that

6.2 $$\begin{aligned} 0<8\delta< \gamma < 1-8\delta , \qquad M\le \varepsilon . \end{aligned}$$

Then for some constant $C_N^\prime$ depending only on $C_N$, the weak decay estimates

6.3 $$\begin{aligned} |Z^I h^1(t,x)|\le \frac{C_N^\prime \varepsilon (1+t)^{2\delta }}{(1+t+r) (1+q_+)^{\gamma }}, \qquad |I|\le N-3, \end{aligned}$$

where $q_\pm =\max \{\pm q,0\}$ and $q=r-t$, hold for all $t\in [0,T_*]$.

Note that the inverse of the metric $g_{\mu \nu }$ can be expressed as

6.4 $$\begin{aligned} g^{\mu \nu } = m^{\mu \nu }+H^{\mu \nu }, \quad \text {and}\quad H^{\mu \nu }=H_0^{\mu \nu }+H_1^{\mu \nu },\quad \text {where}\quad H_0^{\mu \nu } = -{\chi }\big (\tfrac{r}{1+t}\big )\tfrac{M}{r}\delta ^{\mu \nu }, \end{aligned}$$

where $\chi$ is as in (1.13). Then $m^{\mu \nu }\!+H_0^{\mu \nu }\!-h^{1\mu \nu }$, where $h^{1\mu \nu }=m^{\mu \alpha }m^{\nu \beta }h^1_{\alpha \beta }$, is an approximate inverse to $g_{\mu \nu }\!=m_{\mu \nu }\!+h^0_{\mu \nu }\!+h^1_{\mu \nu }$ up to $O(h^2)$, where $h^0$ is as in (1.13). Hence $H_1^{\mu \nu }\! = -h^{1\, \mu \nu }\!\! +O(h^2)$. Therefore $H_1$ will satisfy the same estimates as $h_1$.

We have the following strong decay estimates from the wave coordinate condition for certain tangential components expressed in the null frame:

### Proposition 6.2

Suppose the conditions of Proposition 6.1 hold. Let $\mathcal {N}=\{L,\underline{L},S_1,S_2\}$ be the null frame defined by (1.28). The modified Lie derivative $\widehat{{\mathcal {L}}}_Z$ defined by (6.73) satisfies

6.5 $$\begin{aligned} |\partial _q (\widehat{{\mathcal {L}}}_Z^I{H}_1)_{LT}| +|\partial _q \,/{\!\!\!\!\text {tr}}\,\widehat{{\mathcal {L}}}_Z^I {H}_1 |&\le C_N^{\prime \prime } \varepsilon (1+t+r)^{-2+2\delta }(1\!+q_+)^{-\gamma }, \end{aligned}$$

6.6 $$\begin{aligned} |(\widehat{{\mathcal {L}}}_Z^I{H}_1)_{L T}| +|\,/{\!\!\!\!\text {tr}}\,\widehat{{\mathcal {L}}}_Z^I {H}_1 |&\le C_N^{\prime \prime } \varepsilon (1\!+t\!+r)^{-1-\gamma +2\delta } (1\!+q_-)^{\gamma } \end{aligned}$$

for $\vert I \vert \le N-4$, where $\,/{\!\!\!\!\text {tr}}\,H_1=H_{1S_1 S_1}+H_{1S_2 S_2}$ and $T\in \mathcal {T}=\{L,S_1,S_2\}$, the subset that spans the tangent space of the outgoing light cones. Here the constant $C_N^\prime$ depends only on $C_N^\prime$ in (6.3) and on *N*.

### Proposition 6.3

Suppose that $N\ge 5$ and the weak decay estimates (6.3) hold for some $8\delta \le \gamma \le 1-8\delta$, $M\le \varepsilon \le 1$ and that there is a constant $0<c<1$ such that

6.7 $$\begin{aligned} \mathrm {supp}\, {\,\widehat{\!T\!}\,\,}(t,x)\subset \{(t,x);\,|x|\le K+ct\}, \qquad c<1. \end{aligned}$$

Then the following strong decay estimates hold: for any $-1\le \gamma ^\prime <\gamma -2\delta$, and $|I|=k\le N-5$ there are constants $c_k$ such that

6.8 $$\begin{aligned} \big | \,\partial Z^I h^1\big |\le c_k \varepsilon (1+t)^{c_k\varepsilon } (1+t+r)^{-1}(1+q_+)^{-1-\gamma ^\prime }. \end{aligned}$$

In addition we have the following estimates for certain tangential components expressed in a null frame:

6.9 $$\begin{aligned} \big | \,\partial h^1_{TU}\big |\le c_0\varepsilon (1+t+r)^{-1}(1+q_+)^{-1-\gamma ^\prime },\qquad T\in \mathcal {T},\quad U\in \mathcal {N}. \end{aligned}$$

Here all constants depend only on $C_N^\prime$ in (6.3), on *N* and on *c*, *K* in (6.7).

### Theorem 6.4

Suppose that $N\ge 9$ and the decay and support conditions (6.2)–(6.9) hold. Then there is a $\varepsilon _N>0$ and constants $C_N^{\prime \prime \prime }$, $d_1,\ldots ,d_N$, depending only on *N*, $C_N$, *c*, *K* and a lower positive bound for $\min {(\gamma ,1-\gamma )}$, such that for all $t\in [0,T_*]$ and $\varepsilon <\varepsilon _N$,

$$\begin{aligned} Q_k(t)\le & {} 8 Q_k(0) + M_k M + C^{\prime \prime \prime }_N\varepsilon \!\int _0^t \frac{Q_{k}(\tau )}{1+\tau } + \frac{Q_{k-1}(\tau )}{(1+\tau )^{1-d_k\varepsilon }} \,\mathrm{d}\tau \\&+ M_k \sum _{|I|\le k} \int _0^t \Vert Z^I \widehat{T} (\tau ,\cdot ) \Vert _{L^{2}} \,\mathrm{d}\tau , \end{aligned}$$

where $Q_k(t) := \sup _{0\le \tau \le t} E_k(\tau )^{1/2}$ and $Q_{-1}(0) \equiv 0$, and $M_1,\ldots ,M_k$ are universal constants.

In the proof of Theorem 1.2 in Section 7, Proposition 6.1 will first be appealed to for the coupled Einstein–Vlasov system (1.2), (1.3), (1.11). As a consequence the assumptions of Proposition 2.1 will be satisfied, which in turn will ensure that the assumptions of Proposition 6.3 and hence of Theorem 6.4 are satisfied.

### Weak $L^\infty$ Decay Estimates

Here we assume the weak energy bounds (6.1) and prove that this implies certain decay estimates.

#### The Weak Decay Estimates for the Metric

##### Lemma 6.5

*(The Klainerman–Sobolev inequalities with weights)* Let $\phi$ be a real valued function and let *w* be as in (1.14). Then

$$\begin{aligned} | \phi (t,x)|w^{1/2}\le \frac{C \sum _{|I|\le 3} \Vert w^{1/2} Z^I \phi (t,\cdot )\Vert _{L^2} }{(1\!+\!t\!+\!r)(1\!+\!|t-r|)^{1/2}}. \end{aligned}$$

For a proof of this, see Proposition 14.1 in [37]. Using this we get

##### Proposition 6.6

Suppose that the weak energy bounds (6.1) hold. Then

6.10 $$\begin{aligned} |\partial Z^I h^1(t,x)|\le {\left\{ \begin{array}{ll} C\varepsilon (1+t+r)^{-1} (1+|r-t|)^{-1-\gamma }(1+t)^\delta , \qquad r>t\\ C\varepsilon (1+t+r)^{-1}(1+|r-t|)^{-1/2}(1+t)^\delta ,\qquad r<t \end{array}\right. },\qquad |I|\le N-3. \end{aligned}$$

Furthermore,

6.11 $$\begin{aligned} |Z^I h^1(t,x)|\le {\left\{ \begin{array}{ll} C\varepsilon (1+t+r)^{-1} (1+|r-t|)^{-\gamma }(1+t)^\delta , \qquad r>t\\ C\varepsilon (1+t+r)^{-1}(1+|r-t|)^{1/2}(1+t)^\delta ,\qquad r<t \end{array}\right. },\qquad |I|\le N-3. \end{aligned}$$

The same estimates hold for $H_1$ in place of $h^1$, and for *h* or *H* in place of $h_1$ if $\gamma$ is replaced by $\delta$.

##### Proof

(6.11) follows from integrating (6.10) in the $r-t$ direction from data, see Corollary 9.4 in [37]. $\quad \square$

That $H_1$ satisfy the same estimates as $h^1$ follows from the discussion after (6.4). That *h* and respectively, *H*, only satisfy these estimates with $\gamma$ replaced with $\delta$ follows from the fact that $h^0$ and $H_0$, given by (1.13) and (6.4), respectively, only satisfy these estimates.

#### The Improved Weak Decay Estimates for the Metric

To get improved decay estimates in the interior we will use Hörmander’s $L^1$–$L^\infty$ estimates for the fundamental solution of $\square$, see Theorem 3.5 in [31].

##### Lemma 6.7

Suppose that *u* is a solution of $\square u=F$ (that is the flat Minkowski wave operator) with vanishing data $u\big |_{t=0}=\partial _t u\big |_{t=0}=0$. Then

6.12 $$\begin{aligned} |u(t,x)|(1+t+|x|) \le C\sum _{|I|\le 2}\int _0^t\int _{\mathbf {R}^3}\frac{|Z^I F(s,y)|}{1+s+|y|}\, \mathrm{d}y \mathrm{d}s. \end{aligned}$$

Also, for the linear homogenous solution, we have, by Lemma 10 in [31],

##### Lemma 6.8

If *v* is the solution of $\square v=0$, with data $v\big |_{t=0}=v_0$ and $\partial _t v\big |_{t=0}=v_1$, then for any $\gamma >0$,

6.13 $$\begin{aligned} (1+t)|v(t,x)| \le C\sup _x \big ( (1+|x|)^{2+\gamma } ( |v_1(x)|+|\partial v_0(x)|) +(1+ |x|)^{1+\gamma }|v_0(x)|\big ). \end{aligned}$$

For the proof below we will also use the following version of Hardy’s inequality, see Corollary 13.3 in [37]:

##### Lemma 6.9

For any $-1\le a\le 1$ and any $\phi \in C^\infty _0({{\mathbb {R}}}^3)$,

6.14 $$\begin{aligned} \int \frac{|\phi |^2}{(1+|t-r|)^2}\,\frac{ w\, \mathrm{d}x}{(1+t+r)^{1-a}} \lesssim \int |\partial \phi |^2\, \frac{ w\, \mathrm{d}x}{(1+t+r)^{1-a}}. \end{aligned}$$

On the other hand we can also estimate derivatives in terms of the vector fields as follows:

##### Lemma 6.10

Let $\partial _q=(\partial _r-\partial _t)/2$ and let ${\bar{\partial }}_\mu =\partial _\mu -L_\mu \partial _q$ be the projection of $\partial _\mu$ onto the tangent space of the outgoing light cones. Then

6.15 $$\begin{aligned} |\partial \phi (t,x)|\lesssim |\partial _q \phi (t,x)|+|{\bar{\partial }}\phi (t,x)| \end{aligned}$$

and

6.16 $$\begin{aligned} (1+|t-r|)|\partial \phi (t,x)|+(1+t+r)|\overline{\partial }\phi (t,x)|\lesssim \sum _{|I|=1} |Z^I \phi (t,x)|. \end{aligned}$$

##### Proposition 6.11

Suppose that $N\ge 4$ and the weak energy bounds (6.1) hold for a solution of Einstein–Vlasov in wave coordinates. Then

6.17 $$\begin{aligned} |Z^I h^1(t,x)|\le \frac{C\varepsilon (1+t)^{2\delta }}{(1+t+r) (1+q_+)^{\gamma }}, \qquad |I|\le N-3, \end{aligned}$$

where $q_\pm =\max \{\pm q,0\}$ and $q=r-t$. Moreover,

6.18 $$\begin{aligned}&(1+|q|)\big |\partial Z^I h^1(t,x)\big |+(1+t+r)\big |\overline{\partial } Z^I h^1(t,x)\big |\nonumber \\&\quad \le \frac{C\varepsilon (1+t)^{2\delta }}{(1+t+r)(1+q_+)^{\gamma }},\qquad |I|\le N-4. \end{aligned}$$

The same estimates hold for $H_1$ in place of $h^1$, and *h* or *H* in place of $h^1$ if $\gamma$ is replaced by $2\delta$.

##### Proof

First (6.18) is a consequence of (6.17) using (6.16) so it only remains to prove (6.17) for $r<t$. Let $h^1_{\mu \nu }=v_{\mu \nu }+u_{\mu \nu }+\phi _{\mu \nu }$ where,

6.19 $$\begin{aligned} \square v_{\mu \nu }=0,\qquad v_{\mu \nu }\big |_{t=0}=h^1_{\mu \nu }\big |_{t=0},\qquad \partial _t v_{\mu \nu }\big |_{t=0}=\partial _t h^1_{\mu \nu }\big |_{t=0}. \end{aligned}$$

and

6.20 $$\begin{aligned} \square u_{\mu \nu }&= -H^{\alpha \beta }\partial _\alpha \partial _\beta h_{\mu \nu } +F_{\mu \nu }(h)(\partial h,\partial h)-\square h_{\mu \nu }^0,\qquad&u_{\mu \nu }\big |_{t=0}=\partial _t u_{\mu \nu }\big |_{t=0}=0, \end{aligned}$$

6.21 $$\begin{aligned} \square \phi _{\mu \nu }&=\widehat{T}_{\mu \nu },\qquad&\phi _{\mu \nu }\big |_{t=0}=\partial _t \phi _{\mu \nu }\big |_{t=0}=0. \end{aligned}$$

We will prove (6.17) for $r<t$ separately for each of $v,u,\phi$. For *v* and *u* the proof follows the proof in section 16 of [37]. The estimate for the homogeneous linear part *v* follows from using Lemma 6.8 with the estimate for $v_0$ and $v_1$ obtained from (6.10)–(6.11) when $t=0$.

Then using the $L^\infty$ bounds (6.10)–(6.11) for a small number of vector fields we have

6.22 $$\begin{aligned} |Z^I F_{\mu \nu }(h)(\partial h,\partial h)|\le C\!\!\!\sum _{|J|+|K|\le |I|\!\!\!\!\!\!}|\partial Z^J h|\, |\partial Z^K h| +C\!\!\! \sum _{|J|+|K|\le |I|} \frac{|Z^J h|}{1+|q|}\, |\partial Z^K h|, \end{aligned}$$

and since $$H^{\alpha \beta }=-h_{\alpha \beta }+O(h^2)$$,

6.23 $$\begin{aligned} \big |Z^I\big ( H^{\alpha \beta }\partial _\alpha \partial _\beta h_{\mu \nu }\big )\big |\le C\!\!\! \sum _{|J|+|K|\le |I|+1,\, |J|\le |I|} \frac{|Z^J h|}{1+|q|}\, |\partial Z^K h|. \end{aligned}$$

Now

6.24 $$\begin{aligned} \int |\partial Z^J h|\, |\partial Z^K h|(s,y)\, \mathrm{d}y \le \sum _{|I|\le N}\Vert \partial Z^I h(s,\cdot )\Vert _{L^2}^2\le C\varepsilon ^2(1+s)^{2\delta }, \end{aligned}$$

since

6.25 $$\begin{aligned} \int |\partial Z^I h^0(s,y)|^2\, \mathrm{d}y \le C M^2 \int _0^\infty \frac{r^2\, dr}{(1+|t+r|)^4}\le C^\prime M^2. \end{aligned}$$

We write $h=h^0+h^1$ and estimate

6.26 $$\begin{aligned} \int \frac{|Z^J h^0(t,x)|^2}{(1+|t-r|)^2}\,\mathrm{d}x \le CM^2 \int _0^\infty \frac{r^2\, dr}{(1+|t+r|)^2(1+|t-r|)^2}\le C^\prime M^2, \end{aligned}$$

and by Lemma 6.9,

6.27 $$\begin{aligned} \int \frac{|Z^J h^1(t,x)|^2}{(1+|t-r|)^2}\,\mathrm{d}x \le C\int |\partial Z^J h^1(t,x)|^2 w\, \mathrm{d}x\le C\varepsilon ^2 (1+t)^{2\delta }. \end{aligned}$$

Hence,

6.28 $$\begin{aligned} \int \frac{|Z^J h|}{1+|q|}\, |\partial Z^K h|(s,y)\, \mathrm{d}y \le C\varepsilon ^2 (1+t)^{2\delta }. \end{aligned}$$

Finally,

6.29 $$\begin{aligned} |\square h_{\mu \nu }^0| =\Big |\frac{M\delta _{\mu \nu }}{r}(\partial _t+\partial _r)(\partial _t-\partial _r)\big (\chi (\tfrac{r}{1+t})\big )\Big | \le \frac{CM H(r<3t/4)}{(1+t+r)^3}, \end{aligned}$$

where $H(r<3t/4)=1$ when $r<3t/4$ and 0 otherwise. Hence

6.30 $$\begin{aligned} \Vert \, \square h^0(t,\cdot )\Vert _{L^1}\le M. \end{aligned}$$

It now follows from Lemma 6.7 that

6.31 $$\begin{aligned} |u_{\mu \nu }(t,x)|(1+t+|x|)\le \int _0^t \frac{(\varepsilon ^2 +M)\, \mathrm{d}s}{(1+s)^{1-2\delta }}\le C\varepsilon (1+s)^{2\delta }, \end{aligned}$$

which proves (6.17) for $r<t$ and also for *u*. It remains to prove the estimate for $\phi$ as well, but this also follows from Lemma 6.7:

6.32 $$\begin{aligned} |\phi _{\mu \nu }(t,x)|(1+t+|x|)\le \int _0^t \frac{C\Vert \widehat{T}(s,\cdot )\Vert _{L^1}\, \mathrm{d}s}{1+s}\le \int _0^t \frac{C\varepsilon \, \mathrm{d}s}{(1+s)^{1-\delta }}\le C\varepsilon (1+s)^{\delta }. \end{aligned}$$

$$\square $$

#### The Support and Weak Decay of Matter

The following Sobolev inequality will be used to obtain pointwise bounds for ${\,\widehat{\!T\!}\,\,}$ from the assumptions (6.1):

##### Lemma 6.12

If $\mathrm {supp}\,\phi \subset \{(t,x);\, |x|\le K+ct\}$ for some $K\ge 0$ and $0<c<1$, then

$$\begin{aligned} | \phi (t,x)|\lesssim (1+t+r)^{-3} {\sum }_{|I| = 3} \Vert Z^I \phi (t,\cdot )\Vert _{L^1}. \end{aligned}$$

##### Proof

The proof proceeds by noting that, with $c' = (1+c)/2$, $\mathrm {supp}\,\phi (t,\cdot ) \subset \{(t,x);\, |x|\le c't\}$ for $t\ge 2K/(1-c)$. The inequality for $t \le 2K/(1-c)$ follows from the standard Sobolev inequality,

$$\begin{aligned} \vert \phi (t,x) \vert \lesssim \sum _{\vert I \vert = 3} \vert \partial ^I \phi (t,\cdot ) \vert . \end{aligned}$$

The proof for $t \ge 2K/(1-c)$ follows from the identity (2.12). $\quad \square$

Lemma 6.12, together with assumption (6.7) on the support of ${\,\widehat{\!T\!}\,\,}$ and the weak energy bounds (6.1), gives

6.33 $$\begin{aligned} |Z^I T(t,x)|\lesssim \varepsilon (1+t)^{-3}, \qquad |I|\le N-4. \end{aligned}$$

### The Sharp Decay Estimates for the First Order Derivatives

Throughout the rest of this section we will assume that the weak decay estimates (6.17)–(6.18) hold for some $0<8\delta<\gamma <1-8\delta$, $M\le \varepsilon \le 1$, along with the support condition (6.7) for $\widehat{T}$. However we will not use the weak energy bounds (6.1) any further.

#### The Sharp Decay Estimates for First Order Derivative of Certain Components from the Wave Coordinate Condition

By (2.20 in [34] the wave coordinate condition can be written as

6.34 $$\begin{aligned} \partial _\mu \widehat{H}^{\mu \nu \!}\! = W^{\nu }(h,\partial h)\qquad \text {where}\quad \widehat{H}^{\mu \nu } = H^{\mu \nu }\!-m^{\mu \nu } \text {tr}_m H_{\!}/2,\quad \text {tr}_m H\!=m_{\alpha \beta } H^{\alpha \beta } \end{aligned}$$

and $$|W(h,\partial h)|\lesssim |h|\, |\partial h |$$. Moreover,

6.35 $$\begin{aligned} \partial _\mu \widehat{H}_1^{\mu \nu \!}\! = W^{\nu }(h,\partial h)-\partial _\mu \widehat{H}_0^{\mu \nu \!},\quad \text {where}\quad \partial _\mu \widehat{H}_0^{\mu \nu \!}=2{\chi }^{\,\prime }\big (\tfrac{r}{1+t}\big ) M(1\!+t)^{-2}\delta ^{0\nu }. \end{aligned}$$

We first express the divergence in a null frame as follows:

##### Lemma 6.13

Let $\partial _q=(\partial _r\!-\partial _t)/2$ and $\partial _s=(\partial _r\!+\partial _t)/2$. Then for any tensor $k^{\mu \nu }$,

6.36 $$\begin{aligned} \partial _q \big (L_\mu U_\nu k^{\mu \nu }\big )\!=L_\mu U_\nu \partial _q k^{\mu \nu }\!= \underline{L}_{\,\mu } U_\nu \partial _s k^{\mu \nu } -A_\mu U_\nu \partial _A k^{\mu \nu }+U_\nu \,\partial _\mu k^{\mu \nu }\!\!, \qquad U\in \mathcal {N}. \end{aligned}$$

##### Proof

The proof follows expressing the divergence in a null frame $\partial _\mu F^\mu \!=L_\mu \partial _q F^\mu -\underline{L}_{\,\mu } \partial _s F^\mu \!+A_\mu \partial _A F^\mu \!$; for when $\partial _q$ and $\partial _s$ commute with the frame, see Lemma 1 in [34]. $\quad \square$

Using this, we get

##### Lemma 6.14

We have

6.37 $$\begin{aligned} |\,\partial _q {H}_{LT}| +|\,\partial _q \,/{\!\!\!\!\text {tr}}\,H|&\lesssim |\overline{\partial } H| + |h|\, |\partial h|, \end{aligned}$$

6.38 $$\begin{aligned} |\,\partial _q {H}_{1LT}| +|\,\partial _q \,/{\!\!\!\!\text {tr}}\,H_1 |&\lesssim |\overline{\partial } H_1| + |h|\, |\partial h|+M \big |\chi ^\prime \big (\tfrac{r}{t+1}\big )\big | (1+t+r)^{-2}, \end{aligned}$$

where $\chi ^\prime (s)$, is a function supported when $1/4\le s\le 1/2$. Moreover (6.37) also holds for *h* in place of *H*.

##### Proof

It follows from the previous lemmas that

$$\begin{aligned} |\partial _q \widehat{H}_{1\,LU}|\lesssim |\overline{\partial } H_1|+|h|\,|\partial h| +M\big |\chi ^\prime \big (\tfrac{r}{t+1}\big )\big |(1+t+r)^{-2}. \end{aligned}$$

Picking $U=T$ and, respectively, $U=\underline{L}$, gives (6.38). $\quad \square$

##### Proposition 6.15

With $H_{1UV}=H_1^{\mu \nu }U_\mu V_\nu$ and $\,/{\!\!\!\!\text {tr}}\,H_1=\delta ^{AB} H_{1AB}$ we have, for $T=\{L,S_1,S_2\}$

6.39 $$\begin{aligned} |\,\partial _q {H}_{1LT}| +|\,\partial _q \,/{\!\!\!\!\text {tr}}\,H_1 |&\lesssim \varepsilon (1+t+r)^{-2+2\delta }(1\!+q_+)^{-\gamma }, \end{aligned}$$

6.40 $$\begin{aligned} |{H}_{1LT}| \!+|\,/{\!\!\!\!\text {tr}}\,{H}_{1}|&\lesssim \varepsilon (1\!+t\!+r)^{-1-\gamma +2\delta }\!\! + \varepsilon (1\!+t)^{-2+2\delta }(1\!+q_-) \nonumber \\&\lesssim \varepsilon (1\!+t\!+r)^{-1-\gamma +2\delta } (1\!+q_-)^{\gamma }. \end{aligned}$$

The same estimates hold for *H* in place of $H_1$ if $\gamma$ is replaced by $2\delta$,

##### Proof

The proof follows as in the proof of Proposition 13 in [34]. (6.40) follows from integrating (6.39) in the $t-r$ direction from initial data. When $|t-r|>t/8$ the estimates follow from Proposition 6.11 so we may assume that $|t-r|<t/8$. It then follows from Lemma 6.14 and Proposition 6.11 that

6.41 $$\begin{aligned} |\partial _q {H}_{1LT}| +|\partial _q \,/{\!\!\!\!\text {tr}}\,H_1 |\lesssim & {} |\overline{\partial } H_1|+|h|\,|\partial h| \lesssim \varepsilon (1+t+r)^{-2+2\delta }(1\!+q_+)^{-\gamma }\nonumber \\&+\varepsilon ^2(1+t+r)^{-2+2\delta }(1\!+q_+)^{-1-2\delta }. \end{aligned}$$

$$\square $$

#### The Leading Order Behaviour of the Inhomogeneous Term Towards Null Infinity

The inhomogeneous term in Einstein’s equations can be written as

6.42 $$\begin{aligned} F_{\mu \nu }&= P(\partial _{\mu } h,\partial _{\nu } h) + Q_{\mu \nu }(\partial h,\partial h)+G_{\mu \nu }(h)(\partial h,\partial h),\nonumber \\ P(h, k)&= \frac{1}{2} m^{\alpha \alpha ^\prime }m^{\beta \beta ^\prime } \ h_{\alpha \beta }\, k_{\alpha ^\prime \beta ^\prime } -\frac{1}{4} m^{\alpha \alpha ^\prime } h_{\alpha \alpha ^\prime } \, m^{\beta \beta ^\prime } h_{\beta \beta ^\prime }, \end{aligned}$$

where $G_{\mu \nu }(h)(\partial h,\partial h)$ is cubic:

$$\begin{aligned} | \,G_{\mu \nu }(h)(\partial h,\partial h)| \lesssim |h|\, |\partial h|^2, \end{aligned}$$

and $Q_{\mu \nu }(\partial h,\partial h)$ satisfy the standard null condition, and hence

6.43 $$\begin{aligned} |Q(\partial h,\partial k)|\lesssim |\overline{\partial } h|\,|\partial k|+|\partial h|\, |\overline{\partial } k|. \end{aligned}$$

The main term $P(\partial _{\mu } h,\partial _{\nu } h)$ can be further analyzed by first noting that

6.44 $$\begin{aligned} \big | P(\partial _\mu h,\partial _\nu k)- L_{\mu }L_\nu P(\partial _q h,\partial _q k)\big |\lesssim |\overline{\partial } h| \,|\partial k| +|\partial h|\,|\overline{\partial } k|, \end{aligned}$$

which follows from expressing $\partial _\mu$ in a null frame: $\partial _\mu =L_\mu \partial _q -\underline{L}_{\,\mu } \partial _s + A_\mu \partial _A$. Expressing $P(h,k)=P_{{\mathcal {N}}}(h,k)$ in a null frame we have

6.45 $$\begin{aligned} P_\mathcal {N\,}(h,k)= & {} -\frac{1}{8}\big ({h}_{LL} {k}_{\underline{L}\underline{L}} +{h}_{\underline{L}\underline{L}} {k}_{{L}{L}}\big ) -\frac{1}{4}\delta ^{CD}\delta ^{C^\prime D^\prime }\big (2{h}_{CC^\prime }{k}_{DD^\prime }- {h}_{CD} {k}_{C^\prime D^\prime }\big )\nonumber \\&+\frac{1}{4}\delta ^{CD}\big (2{h}_{C L}{k}_{D\underline{L}} +2{h}_{C \underline{L}}{k}_{D{L}}- {h}_{CD} {k}_{L\underline{L}}-{h}_{L\underline{L}} {k}_{CD} \big ). \end{aligned}$$

Taking into account the wave coordinate condition this reduces in leading order to $P_{\!\mathcal {N}}(\partial _q h,\partial _q h)\sim P_{\!\mathcal {S}}(\partial _q h,\partial _q h)$, where

6.46 $$\begin{aligned}&P_{\mathcal {S}} (D,E)= -\widehat{D}_{AB}\, \widehat{E}^{AB}\!/2,\quad A,B\in \mathcal {S},\nonumber \\&\quad \text {where} \quad \widehat{D}_{AB}=D_{AB}-\delta _{AB}\,/{\!\!\!\!\text {tr}}\,D/2,\quad \,/{\!\!\!\!\text {tr}}\,D=\delta ^{AB}D_{AB}. \end{aligned}$$

In fact, by (6.45) we have

6.47 $$\begin{aligned}&\big |P_{{\mathcal {N}}}(h,k)-P_{{\mathcal {S}}}(h,k)|\big |\lesssim \big (|h\,|_{L\mathcal T}+|\,/{\!\!\!\!\text {tr}}\,h|\big )|k| +|h\,|\big (|k\,|_{L\mathcal T}+|\,/{\!\!\!\!\text {tr}}\,k_{}|\big ),\nonumber \\&\quad \text {where}\quad |h|_{L\mathcal T}=|h_{LL}|+|h_{LS_1}|+|h_{LS_2}|. \end{aligned}$$

Also, using (6.37) and that fact the $H=-h+O(h^2)$, we get

6.48 $$\begin{aligned} \big | P_{{\mathcal {N}}}(\partial _q h,\partial _q h)-P_{{\mathcal {S}}}(\partial _q h,\partial _q h)\big | \lesssim (|\overline{\partial } h|+|h||\partial h|) |\partial h|. \end{aligned}$$

Summing up, we have shown

##### Lemma 6.16

Let

6.49 $$\begin{aligned} {/\!\!\!\!} P_{\mu \nu }(\partial h,\partial k)=\overline{\chi }\big (\tfrac{\langle \,r-t\,\rangle }{t+r}\big ) L_{\mu }L_\nu P_\mathcal {S\,}(\partial _q h,\partial _q h), \end{aligned}$$

where $\overline{\chi }_{\!}\!\in \! C_0^\infty \!\!$ satisfies $\overline{\chi }(s)_{\!} = 0$, when $|s|\!\ge \!3_{\!}/4$ and $\overline{\chi }(s)_{\!} = 1$, when $|s|\!\le \! 1_{\!}/2$. Here $\langle q\rangle =(1+|q|^2)^{1/2}$. Then

6.50 $$\begin{aligned} \big |F_{\mu \nu }(h)(\partial h,\partial h)- {/\!\!\!\!} P_{\mu \nu }(\partial h,\partial h) \big | \lesssim |\overline{\partial } h| |\partial h|+| h| |\partial h|^2\!\!, \qquad \text {when}\quad \tfrac{\langle \,r-t\,\rangle }{t+r}\le 1/2. \end{aligned}$$

Using (6.18)–(6.17) and (6.11)–(6.10), we obtain

##### Lemma 6.17

With notation as in the previous lemma we have that

6.51 $$\begin{aligned} \big |F_{\mu \nu }(h)(\partial h,\partial h)- {/\!\!\!\!} P_{\mu \nu }(\partial h,\partial h) \big | \lesssim \frac{\varepsilon ^2}{(1\!+t\!+r)^{3-4\delta }(1\!+|q|)(1\!+q_+)^{4\delta }}. \end{aligned}$$

#### The Leading Order of the Geometric Wave Operator Towards Null Infinity

Expanding in a null frame as in the proof of Lemma 6.13 and using (6.16), we get

##### Lemma 6.18

We have

6.52 $$\begin{aligned} \big |k^{\alpha \beta }\partial _\alpha \partial _\beta \phi \big |\lesssim \Big (\frac{| k_{LL}| }{1+|q|} + \frac{|k| }{1+t+r}\Big )\sum _{|K|\le 1}|\partial Z^K \phi |. \end{aligned}$$

As a consequence, we get

##### Lemma 6.19

We have

6.53 $$\begin{aligned} \big |(\widetilde{\square }_g -\square _0)\phi \big |\lesssim \frac{\varepsilon (1+q_+)^{-\gamma }}{(1+t+r)^{1+\gamma -2\delta }(1+|q|)^{1-\gamma }}\sum _{|K|\le 1}|\partial Z^K \phi | , \end{aligned}$$

where the asymptotic Schwarzschild wave operator is given by

6.54 $$\begin{aligned} \square _{\,0}=\big (m^{\alpha \beta }+H_0^{\alpha \beta }\big )\partial _\alpha \partial _\beta , \quad \text {where}\quad H_0^{\alpha \beta }=-\tfrac{M}{r}{\chi }\big (\tfrac{r}{1+t}\big )\, \delta ^{\alpha \beta }. \end{aligned}$$

##### Proof

We apply (6.52) to $H_1^{\alpha \beta }\partial _\alpha \partial _\beta \phi$ using (6.40) and (6.17) to get

$$\begin{aligned}&\big |H_1^{\alpha \beta }\partial _\alpha \partial _\beta \phi \big |\lesssim \Big (\frac{| H_{1\,LL}| }{1+|q|} + \frac{|H_1| }{1+t+r}\Big )\sum _{|K|\le 1}|\partial Z^K \phi | \\&\quad \lesssim \frac{C\varepsilon }{(1+t+r)^{1+\gamma -2\delta }(1+|q|)^{1-\gamma }(1+q_+)^\gamma }\sum _{|K|\le 1}|\partial Z^K \phi |. \end{aligned}$$

$$\square $$

In spherical coordinates, (6.54) takes the form

6.55 $$\begin{aligned} \square _0\phi= & {} \Big (-\partial _t^2+\triangle _x -\tfrac{M}{r}{\chi }\big (\tfrac{r}{1+t}\big )\big (\partial _t^2+\triangle _x\big )\Big )\phi \nonumber \\= & {} \frac{1}{r}\Big (-\partial _t^2+\partial _r^2-\tfrac{M}{r}{\chi }\big (\tfrac{r}{1+t}\big )(\partial _t^2+\partial _r^2)\Big )(r\phi )\nonumber \\&+\Big (1-\tfrac{M}{r}{\chi }\big (\tfrac{r}{1+t}\big )\Big ) \frac{1}{r^2}\triangle _\omega \phi . \end{aligned}$$

#### The Leading Order of the Metric Towards Space Like Infinity

Following [37], we have defined $h^0_{\alpha \beta }=-H_0^{\alpha \beta }$ to be a function that picks up the leading behavior of the initial data at space like infinity:

6.56 $$\begin{aligned} h^0_{\mu \nu }=\tfrac{M}{r}{\chi }\big (\tfrac{r}{1+t}\big ). \delta _{\mu \nu }. \end{aligned}$$

It would, however, perhaps have been more natural to define it to be a solution of the homogeneous wave $\square h^0=0$ (or even better $\square _0 h^0=0$.) with data coinciding with this function at time 0 in which case $h^0_{\mu \nu }=\chi (r-t) \tfrac{M}{r}\delta _{\mu \nu }$ which is equal to (6.56) in the exterior when $r\ge t+1$. We therefore think of (6.56) as an approximate solution to the homogeneous wave equation. By (6.55), we have

6.57 $$\begin{aligned} \square _0 h^0_{\mu \nu }= & {} \frac{1}{r}\Big (-\partial _t^2+\partial _r^2 -\tfrac{M}{r}{\chi }\big (\tfrac{r}{1+t}\big )(\partial _t^2+\partial _r^2)\Big )\chi \big (\tfrac{r}{t+1}\big )\nonumber \\ M\delta _{\mu \nu }= & {} \frac{M\delta _{\mu \nu }}{(1+t)^3} \Big (\chi _1^\prime \big (\tfrac{r}{t+1}\big )+\tfrac{1}{t+1} \chi _2^\prime \big (\tfrac{r}{t+1}\big )\Big ), \end{aligned}$$

for some functions $\chi _i^\prime (s)$ that vanish when $s\ge 1/2$ or $s\le 1/4$; this, in particular, means that in the exterior and in the wave zone it is a solution of the wave operator $\square _0$. Moreover,

6.58 $$\begin{aligned} (\widetilde{\square }_g -\square _0)h^0_{\mu \nu }= H_1^{\alpha \beta }\partial _\alpha \partial _\beta h^0_{\mu \nu }=\frac{M\delta _{\mu \nu }}{(1+t+r)^3} H_1^{\alpha \beta } \chi _{\alpha \beta }\big (\tfrac{r}{t+1},\omega \big ) \end{aligned}$$

for some smooth function $\chi _{\alpha \beta }(s,\omega )$ supported when $s\ge 1/4$. Hence

6.59 $$\begin{aligned} \big |(\widetilde{\square }_g-\square _0) h^0_{\mu \nu }\big |\lesssim \frac{\varepsilon M}{(1+t+r)^{4-2\delta }(1+q_+)^\gamma }. \end{aligned}$$

Summing up, we have proved

##### Proposition 6.20

(Asymptotic Approximate Einstein’s equations.) When $|x|\ge ct$ we have

6.60 $$\begin{aligned} \big |\,\square _{\,0}\, h^1_{\mu \nu }- {/\!\!\!\!}P_{\mu \nu }(\partial h,\partial h)\big | \lesssim \frac{\varepsilon ^2}{(1\!+t\!+r)^{2+\gamma -4\delta } (1\!+|q|)^{2-\gamma }(1\!+q_+)^{4\delta }}. \end{aligned}$$

#### The Sharp Decay Estimates for First Order Derivatives from the Wave Equation

We will now derive sharp estimates for the first order derivatives. Following [31, 37], we have

##### Lemma 6.21

Let $D_t=\{(t,x);\, \big |t-|x|\big |\le c_0 t \}$, for some constant $0<c_0<1$ and let $\overline{w}(q)>0$ be an increasing positive weight $\overline{w}^{\,\prime }(q)\ge 0$. Then

6.61 $$\begin{aligned}&(1+t+|x|) \,|\partial \phi _{UV}(t,x)\, \overline{w}(q)| \lesssim \!\sup _{0\le \tau \le t} \sum _{|I|\le 1}\Vert \,Z^I\! \phi (\tau ,\cdot )\, \overline{w}\Vert _{L^\infty }\nonumber \\&\quad + \int _0^t\Big ( (1+\tau )\Vert \,(\square _0 \phi )_{UV}(\tau ,\cdot )\, \overline{w}\Vert _{L^\infty (D_\tau )} \nonumber \\&\quad +\sum _{|I|\le 2} (1+\tau )^{-1} \Vert Z^I \phi (\tau ,\cdot )\, \overline{w}\Vert _{L^\infty (D_\tau )}\Big )\,\mathrm{d}\tau . \end{aligned}$$

##### Proof

Since $\square \phi =-r^{-1}(\partial _t^2-\partial _r^2)(r\phi )+r^{-2}\triangle _\omega \phi$, where $\triangle _\omega =\sum \Omega _{ij}^2$ and $|Z U|\le C$, for $U\in \{ A,B, L,\underline{L}\}$, it follows that

6.62 $$\begin{aligned} \big | \square _0 {\phi }_{UV} -U^\mu V^\nu \square _0 \phi _{\mu \nu }\big | \le {r}^{-2}\,\, {\sum }_{|J|\le 1} \,\,|Z^{J} \phi |. \end{aligned}$$

Using (6.55), we get

$$\begin{aligned} \square _0\phi =\frac{1}{r}\Big (4\partial _s\partial _q -2\tfrac{M}{r}{\chi }\big (\tfrac{r}{1+t}\big )(\partial _q^2+\partial _s^2)\Big )(r\phi ) +\Big (1-\tfrac{M}{r}{\chi }\big (\tfrac{r}{1+t}\big )\Big ) \frac{1}{r^2}\triangle _\omega \phi , \end{aligned}$$

where $\partial _q=(\partial _r\!-\partial _t)/2$ and $\partial _s=(\partial _r\!+\partial _t)/2$. Hence,

6.63 $$\begin{aligned} \Big |\big (4\partial _s -\tfrac{2M}{r}\partial _q\big )\partial _q (r\phi ) -r \square _0\phi \Big | \lesssim {r}^{-1}\,\, {\sum }_{|J|\le 2} \,\,|Z^{J} \phi |, \end{aligned}$$

so with $$s=t+r$$,

6.64 $$\begin{aligned} \Big |\big (\partial _s -\tfrac{M}{s}\partial _q\big )\partial _q (r \phi _{UV})\Big | \lesssim r\big |(\square _0 \phi )_{UV}\big |+ (t+r)^{-1}\,\, {\sum }_{|J|\le 2} \,\,|Z^{J} \phi |, \qquad |t-r|\le c_0 t. \end{aligned}$$

Integrating this along the flow lines of the vector field $\big (\partial _s -\tfrac{M}{s}\partial _q\big )$ from the boundary of $D=\cup _{\tau \ge 0}D_\tau$ to any point inside *D*, using that $\overline{w}$ is decreasing along the flow lines, gives that, for any $(t,x)\in D$,

6.65 $$\begin{aligned}&|\partial _q(r \phi _{UV}(t,x))\, \overline{w}| \lesssim \!\sup _{0\le \tau \le t} \sum _{|I|\le 1}\Vert \,Z^I \phi (\tau ,\cdot )\, \overline{w}\Vert \nonumber \\&\quad + \int _0^t\Big ( (1+\tau )\Vert \,(\square _0 \phi )_{UV}(\tau ,\cdot )\, \overline{w}\Vert _{L^\infty (D_\tau )} \nonumber \\&\quad +\sum _{|I|\le 2} (1+\tau )^{-1} \Vert Z^I \phi (\tau ,\cdot )\, \overline{w}\Vert _{L^\infty (D_\tau )}\Big )\,\mathrm{d}\tau . \end{aligned}$$

The lemma now follows from (6.15), since it is trivially true when $|r-t|\ge c_0 t$ by (6.16). $\quad \square$

From Lemma 6.21 and the estimate (6.17), we get

##### Lemma 6.22

Let $D_t=\{(t,x);\, \big |t-|x|\big |\le c_0 t \}$ for some constant $0<c_0<1$ and $\overline{w}(q)=(1+q_+)^{1+\gamma ^\prime }$ where $-1\le \gamma ^\prime <\gamma -2\delta$. Then

6.66 $$\begin{aligned} (1+t+|x|) \,|\partial h^1_{UV}(t,x)\, \overline{w}(q)| \lesssim \varepsilon + \int _0^t (1+\tau )\Vert \,(\square _0 h^1)_{UV}(\tau ,\cdot )\, \overline{w}\Vert _{L^\infty (D_\tau )} \,\mathrm{d}\tau . \end{aligned}$$

Using Lemma 6.22 and Proposition 6.20, we obtain

##### Proposition 6.23

If the weak energy bounds and initial bounds hold then we have, for any $0\le \gamma ^\prime <\gamma -4\delta$,

6.67 $$\begin{aligned} (1+t+r) (1+q_+)^{1+\gamma ^\prime }\big | \partial h^1_{TU}\big |&\lesssim \varepsilon , \end{aligned}$$

6.68 $$\begin{aligned} (1+t+r) (1+q_+)^{1+\gamma ^\prime }\big | \partial h^1\big |&\lesssim \varepsilon (1+\varepsilon \ln {(2+t)})\lesssim \varepsilon (1+t)^\varepsilon . \end{aligned}$$

The same estimates hold for *h* in place of $h^1$ if $\gamma ^\prime =0$.

##### Proof

We want to apply Lemma 6.22 to the decomposition in Proposition 6.20. To prove (6.67) we note that ${/\!\!\!\!}P_{TU}=0$. Moreover ${\,\widehat{\!T\!}\,\,}=0$ in $D_t$, for $t \ge 2K/(1-c)$, if we pick $c_0$ so small that $c_0\le 1-c'$, where $c' = (1+c)/2$. Also Lemma 6.12 implies that ${\,\widehat{\!T\!}\,\,}(t,x)$ is uniformly bounded for $0 \le t \le 2K/(1-c)$. From the preceeding lemmas it follows that all the terms in the right hand side of (6.61) are bounded independently of *t* by a constant times $\varepsilon$ when $(U,V)=(U,T)$ and this proves (6.67). To prove (6.68) we note that the only new term is ${/\!\!\!\!}P_{\mu \nu }$, which is controlled by

6.69 $$\begin{aligned} |{/\!\!\!\!}P(\partial h,\partial h)|\le |\partial h_{TS}|^2\lesssim \varepsilon ^2 (1+t+r)^{-2} (1+q_+)^{-2-2\gamma ^\prime } \end{aligned}$$

by the first part and multiplying by $(1+t)$ and integrating gives a logarithm. $\quad \square$

### The Commutators and Lie Derivatives

We will use Lie derivatives which will simplify the commutators very much by removing the lower order terms. It was first observed in [34] that one can get bounds from the wave coordinate condition for Lie derivatives. Here we take it further and observe that the Lie derivative unlike vector fields preserve the geometric null structure of not only the wave coordinate condition, but also of the nonlinear inhomogeneous terms of Einstein’s equations and the commutators with the geometric wave operator.

#### Modified Lie Derivatives Applied to the Equations

The Lie derivative applied to a (*r*, *s*) tensor *K* is defined by

6.70 $$\begin{aligned} {{\mathcal {L}}}_Z K^{\alpha _1\dots \alpha _r}_{\beta _1\dots \beta _s}= & {} Z K^{\alpha _1\dots \alpha _r}_{\beta _1\dots \beta _s} -\partial _\gamma Z^{\alpha _1} K^{\gamma \dots \alpha _r}_{\beta _1\dots \beta _s}-\cdots -\partial _\gamma Z^{\alpha _r} K^{\alpha _1\dots \gamma }_{\beta _1\dots \beta _s} \nonumber \\&+\partial _{\beta _1} Z^{\gamma } K^{\alpha _1\dots \alpha _r}_{\gamma \dots \beta _s}+\cdots +\partial _{\beta _s} Z^{\gamma } K^{\alpha _1\dots \alpha _r}_{\beta _1\dots \gamma }. \end{aligned}$$

Recall that the Lie derivative satisfies the Leibniz rule. For the case of our vector fields, $\partial _\gamma Z^\beta$ are constant, which results in the following commutation properties:

##### Proposition 6.24

If *K* is an (r,s) tensor then, with respect to the coordinate system $\{x^{\mu }\}$, the vector fields $Z = \partial _{x^{\mu }}, \Omega _{ij}, B_i, S$ satisfy

6.71 $$\begin{aligned} {{\mathcal {L}}}_Z\partial _{\mu _1}\!\cdots \partial _{\mu _k} K^{\alpha _1\dots \alpha _r}_{\beta _1\dots \beta _s} = \partial _{\mu _1}\!\cdots \partial _{\mu _k} {{\mathcal {L}}}_Z K^{\alpha _1\dots \alpha _r}_{\beta _1\dots \beta _s}, \end{aligned}$$

and

6.72 $$\begin{aligned} {{\mathcal {L}}}_Z \partial _\mu K^{\mu \dots \alpha _r}_{\beta _1\dots \beta _s} = \partial _\mu {{\mathcal {L}}}_Z K^{\mu \dots \alpha _r}_{\beta _1\dots \beta _s}. \end{aligned}$$

##### Proof

From the definition (6.70),

$$\begin{aligned} {{\mathcal {L}}}_Z\partial _{\mu _1}\!\cdots \partial _{\mu _k} K^{\alpha _1\dots \alpha _r}_{\beta _1\dots \beta _s} =&\, Z \left( \partial _{\mu _1}\!\cdots \partial _{\mu _k} K^{\alpha _1\dots \alpha _r}_{\beta _1\dots \beta _s} \right) \\&+ \partial _{\mu _1} Z^{\gamma } \partial _{\gamma }\!\cdots \partial _{\mu _k} K^{\alpha _1\dots \alpha _r}_{\beta _1\dots \beta _s} + \cdots + \partial _{\mu _k} Z^{\gamma } \partial _{\mu _1}\!\cdots \partial _{\gamma } K^{\alpha _1\dots \alpha _r}_{\beta _1\dots \beta _s}\\&- \partial _{\gamma } Z^{\alpha _1} \partial _{\mu _1}\!\cdots \partial _{\mu _k} K^{\gamma \dots \alpha _r}_{\beta _1\dots \beta _s} - \ldots - \partial _{\gamma } Z^{\alpha _r} \partial _{\mu _1}\!\cdots \partial _{\mu _k} K^{\alpha _1 \dots \gamma }_{\beta _1\dots \beta _s}\\&+\partial _{\beta _1} Z^{\gamma } \partial _{\mu _1}\!\cdots \partial _{\mu _k} K^{\alpha _1\dots \alpha _r}_{\gamma \dots \beta _s} + \cdots + \partial _{\beta _s} Z^{\gamma } \partial _{\mu _1}\!\cdots \partial _{\mu _k} K^{\alpha _1\dots \alpha _r}_{\beta _1 \dots \gamma }, \end{aligned}$$

and

$$\begin{aligned}&\partial _{\mu _1}\!\cdots \partial _{\mu _k} {{\mathcal {L}}}_Z K^{\alpha _1\dots \alpha _r}_{\beta _1\dots \beta _s} \\&\quad = \partial _{\mu _1}\!\cdots \partial _{\mu _k} \bigg [ Z^{\gamma } \partial _{\gamma } \left( K^{\alpha _1\dots \alpha _r}_{\beta _1\dots \beta _s} \right) - \partial _{\gamma } Z^{\alpha _1} K^{\gamma \dots \alpha _r}_{\beta _1\dots \beta _s} - \ldots - \partial _{\gamma } Z^{\alpha _r} K^{\alpha _1 \dots \gamma }_{\beta _1\dots \beta _s}\\&\qquad +\partial _{\beta _1} Z^{\gamma } K^{\alpha _1 \dots \alpha _r}_{\gamma \dots \beta _s} + \cdots + \partial _{\beta _s} Z^{\gamma } K^{\alpha _1 \dots \alpha _r}_{\beta _1\dots \gamma } \bigg ]. \end{aligned}$$

The equality (6.71) follows directly since $\partial _{x^{\alpha }} Z^{\beta }$ is constant for each of the vector fields *Z*. The equality (6.72) follows directly from (6.71). $\quad \square$

Since, for an (*r*, *s*) tensor *K*, the quantity $\partial _{\mu _1}\!\cdots \partial _{\mu _k} K^{\alpha _1\dots \alpha _r}_{\beta _1\dots \beta _s}$ appearing in (6.71) (similar quantities also appear below) is not a geometric object, its Lie derivative is defined formally in the $\{x^{\mu }\}$ coordinate system, using the coordinate expression (6.70). Alternatively, one could note that, in the $\{x^{\mu }\}$ coordinate system,

$$\begin{aligned} \partial _{\mu _1}\!\cdots \partial _{\mu _k} K^{\alpha _1\dots \alpha _r}_{\beta _1\dots \beta _s} = D_{\mu _1}\!\cdots D_{\mu _k} K^{\alpha _1\dots \alpha _r}_{\beta _1\dots \beta _s}, \end{aligned}$$

where *D* denotes the connection of the Minkowski metric, since the Christoffel symbols of *D* with respect to the Cartesian coordinate system $\{ x^{\mu }\}$ vanish, $D_{\partial _{x^{\alpha }}} \partial _{x^{\beta }} = 0$. One could then give a geometric proof of Proposition 6.24 using the fact that the curvature tensor of *D* vanishes and $D^2 Z = 0$ for each of the vector fields *Z*.

Let the modified Lie derivative be defined by

6.73 $$\begin{aligned} \widehat{{\mathcal {L}}}_Z K^{\alpha _1\dots \alpha _r}_{\beta _1\dots \beta _s} ={{\mathcal {L}}}_Z K^{\alpha _1\dots \alpha _r}_{\beta _1\dots \beta _s} +\tfrac{r-s}{4}(\partial _\gamma Z^\gamma )K^{\alpha _1\dots \alpha _r}_{\beta _1\dots \beta _s}. \end{aligned}$$

With this definition $\widehat{{\mathcal {L}}}_Z m^{\alpha \beta }=0$ and $\widehat{{\mathcal {L}}}_Z m_{\alpha \beta }=0$ for the vector fields in our collection, as the modified Lie derivative is defined so it commutes with contractions with the Minkowski metric. Let $h_{\alpha \beta }$ and $k_{\alpha \beta }$ be (0, 2) tensors and let $S_{\mu \nu }(\partial h,\partial k)$ be a (0, 2) tensor which is a quadratic form in the (0, 3) tensors $\partial h$ and $\partial k$ with two contractions with the Minkowski metric (in particular $P(\partial _\mu h,\partial _\nu h)$ or $Q_{\mu \nu }(\partial h,\partial k)$). Then

6.74 $$\begin{aligned} {{\mathcal {L}}}_Z\,\big ( S_{\mu \nu }(\partial h,\partial k)\big ) =S_{\mu \nu }(\partial \widehat{{\mathcal {L}}}_Z h,k)+S_{\mu \nu }(\partial h,\partial \widehat{{\mathcal {L}}}_Z k). \end{aligned}$$

Moreover,

6.75 $$\begin{aligned} {{\mathcal {L}}}_Z \, \big (g^{\alpha \beta } \partial _\alpha \partial _\beta h_{\mu \nu }\big ) =\big (\widehat{{\mathcal {L}}}_Z g^{\alpha \beta } \big )\partial _\alpha \partial _\beta h_{\mu \nu } + g^{\alpha \beta } \partial _\alpha \partial _\beta \widehat{{\mathcal {L}}}_Z h_{\mu \nu }. \end{aligned}$$

Let ${{\mathcal {L}}}_Z^I$ be a product of |*I*| Lie derivatives with respect to |*I*| vector fields *Z*. It follows that

6.76 $$\begin{aligned} \widetilde{\square }_g \widehat{{\mathcal {L}}}_Z^I h_{\mu \nu } =\big [ \widetilde{\square }_g \widehat{{\mathcal {L}}}_Z^I -{{\mathcal {L}}}_Z^I \widetilde{\square }_g\big ]h_{\mu \nu } +{{\mathcal {L}}}_Z^I F_{\mu \nu }(H)(\partial h,\partial h)+{{\mathcal {L}}}_Z^I T_{\mu \nu }, \end{aligned}$$

where

6.77 $$\begin{aligned} \big [ \widetilde{\square }_g \widehat{{\mathcal {L}}}_Z^I -{{\mathcal {L}}}_Z^I \widetilde{\square }_g\big ]\phi _{\mu \nu } =-\!\!\!\!\!\sum _{J+K=I,\,|K|<|I|}\!\!\!\!\! \widehat{{\mathcal {L}}}_Z^J H^{\alpha \beta } \,\partial _\alpha \partial _\beta \widehat{{\mathcal {L}}}_Z^K \phi _{\mu \nu }, \end{aligned}$$

and

6.78 $$\begin{aligned} {{\mathcal {L}}}_Z^I F_{\mu \nu }(H)(\partial h,\partial h)= & {} \sum _{J+K=I}\!\! P\big (\partial _\mu \widehat{{\mathcal {L}}}_Z^J h,\partial _\nu \widehat{{\mathcal {L}}}_Z^K h\big )\nonumber \\&+\sum _{J+K=I}\!\!Q_{\mu \nu }\big (\partial \widehat{{\mathcal {L}}}_Z^J h,\partial \widehat{{\mathcal {L}}}_Z^K h\big )\nonumber \\&+{{\mathcal {L}}}_Z^I G_{\mu \nu }(H)(\partial h,\partial h), \end{aligned}$$

where

6.79 $$\begin{aligned} \big | {{\mathcal {L}}}_Z^I G_{\mu \nu }(H)(\partial h,\partial h)\big |\lesssim \sum _{I_1+I_{2}+\dots I_{k}=I,\, k\ge 3 } |\widehat{{\mathcal {L}}}_Z^{I_3} H| \cdots |\widehat{{\mathcal {L}}}_Z^{I_k} H| |\partial \widehat{{\mathcal {L}}}_Z^{I_1} h|\, |\partial \widehat{{\mathcal {L}}}_Z^{I_2} h|, \end{aligned}$$

that is at least one factor of $|\widehat{{\mathcal {L}}}_Z^{I_k} H|$. Finally, from the wave coordinate condition

6.80 $$\begin{aligned} \partial _\mu \widehat{{\mathcal {L}}}_Z \widehat{H}^{\mu \nu }=\big (\widehat{{\mathcal {L}}}_Z +\tfrac{\partial _\gamma Z^\gamma }{2}\big )\partial _\mu \widehat{H}^{\mu \nu } =\big (\widehat{{\mathcal {L}}}_Z +\tfrac{\partial _\gamma Z^\gamma }{2}\big ) W^{\nu }(H,\partial h), \end{aligned}$$

it follows that

6.81 $$\begin{aligned} \big | \partial _\mu \widehat{{\mathcal {L}}}_Z^I \widehat{H}^{\mu \nu } \big |\lesssim \sum _{I_1+\cdots + I_k,\, k\ge 2} \big | \widehat{{\mathcal {L}}}_Z^{I_2} {H}\big |\cdots \big | \widehat{{\mathcal {L}}}_Z^{I_k} {H}\big |\, \big |\partial \widehat{{\mathcal {L}}}_Z^{I_1} H\big |, \end{aligned}$$

where

6.82 $$\begin{aligned} \widehat{{\mathcal {L}}}_Z^{I}\widehat{H}{}^{\mu \nu } = \widehat{{\mathcal {L}}}_Z^{I}H^{\mu \nu }\!-m^{\mu \nu } \text {tr}_m \widehat{{\mathcal {L}}}_Z^{I}H_{\!}/2,\qquad \text {tr}\widehat{{\mathcal {L}}}_Z^{I}H\!=m_{\alpha \beta } \widehat{{\mathcal {L}}}_Z^{I}H^{\alpha \beta }\!\!. \end{aligned}$$

We have

6.83 $$\begin{aligned} \sum _{|I|\le k} |Z^I K|\lesssim \sum _{|I|\le k}|\widehat{{\mathcal {L}}}_Z^I K|\lesssim \sum _{|I|\le k} |Z^I K|, \end{aligned}$$

since the Lie derivative just adds lower order terms.

#### Estimates from the Wave Coordinate Condition

It follows from Lemma 6.13 and (6.81), and the fact that $\big | \widehat{{\mathcal {L}}}_Z^{I_k} {h}\big |\lesssim 1$ for small $|I_k|$, that we have

##### Lemma 6.25

6.84 $$\begin{aligned} |\partial _q \widehat{{\mathcal {L}}}_Z^I{H}|_{L{\mathcal {T}}} +|\partial _q \,/{\!\!\!\!\text {tr}}\,\widehat{{\mathcal {L}}}_Z^I {H} |\lesssim&|\overline{\partial } \widehat{{\mathcal {L}}}_Z^I H| + \sum _{|J|+|K|\le |I|} \big | \widehat{{\mathcal {L}}}_Z^{J} {h}\big | \big |\partial \widehat{{\mathcal {L}}}_Z^{K} h\big | \end{aligned}$$

6.85 $$\begin{aligned} |\partial _q \widehat{{\mathcal {L}}}_Z^I{H}_1|_{L{\mathcal {T}}} +|\partial _q \,/{\!\!\!\!\text {tr}}\,\widehat{{\mathcal {L}}}_Z^I {H} |\lesssim&|\overline{\partial } \widehat{{\mathcal {L}}}_Z^I H_1| \nonumber \\&+ \sum _{|J|+|K|\le |I|} \big | \widehat{{\mathcal {L}}}_Z^{J} {h}\big | \big |\partial \widehat{{\mathcal {L}}}_Z^{K} h\big |+\big |\chi ^\prime \big (\tfrac{r}{t+1}\big )\big | \frac{M}{(1+t+r)^2}, \end{aligned}$$

where $\chi ^\prime (s)$, is a function supported when $1/4\le s\le 1/2$. Moreover, (6.84) also holds for *h* in place of *H*.

#### $L^\infty$ estimates from the Wave Coordinate Condition

For low derivatives, (6.85) leads to the following:

##### Proposition 6.26

For $|I|\le N-4$, we have

6.86 $$\begin{aligned} |\partial _q \widehat{{\mathcal {L}}}_Z^I{H}_1|_{L{\mathcal {T}}} +|\partial _q \,/{\!\!\!\!\text {tr}}\,\widehat{{\mathcal {L}}}_Z^I {H}_1 |&\lesssim \varepsilon (1+t+r)^{-2+2\delta }(1\!+q_+)^{-\gamma }, \end{aligned}$$

6.87 $$\begin{aligned} |\widehat{{\mathcal {L}}}_Z^I{H}_1|_{L{\mathcal {T}}} +|\,/{\!\!\!\!\text {tr}}\,\widehat{{\mathcal {L}}}_Z^I {H}_1 |&\lesssim \varepsilon (1\!+t\!+r)^{-1-\gamma +2\delta }\!\! + \varepsilon (1\!+t)^{-2+2\delta }(1\!+q_-) \nonumber \\&\lesssim \varepsilon (1\!+t\!+r)^{-1-\gamma +2\delta } (1\!+q_-)^{\gamma }. \end{aligned}$$

The same estimates hold for *H* in place of $H_1$ if $\gamma$ is replaced by $2\delta$.

The proof is the same as for Proposition 6.15.

#### Estimates for the Inhomogeneous Term

First using the fact that $\big | \widehat{{\mathcal {L}}}_Z^{I_k} {h}\big |\lesssim 1$ for small $|I_k|$

6.88 $$\begin{aligned} \big | {{\mathcal {L}}}_Z^I G_{\mu \nu }(h)(\partial h,\partial h)\big |\lesssim \sum _{|I_1|+|I_{2}|+|I_{3}|\le |I|,\, } |\widehat{{\mathcal {L}}}_Z^{I_3} H| |\partial \widehat{{\mathcal {L}}}_Z^{I_1} h|\, |\partial \widehat{{\mathcal {L}}}_Z^{I_2} h|. \end{aligned}$$

Secondly, for any term satisfying classical null condition by (6.43), we have

6.89 $$\begin{aligned} \Big |\sum _{J+K=I}\!\!Q_{\mu \nu }\big (\partial \widehat{{\mathcal {L}}}_Z^J h,\partial \widehat{{\mathcal {L}}}_Z^K h\big )\Big |\lesssim \sum _{|J|+|K|\le |I|}\!\! |\overline{\partial } \widehat{{\mathcal {L}}}_Z^J h|\, |{\partial } \widehat{{\mathcal {L}}}_Z^K h|. \end{aligned}$$

Moreover by (6.47) and (6.44), we have, with ${/\!\!\!\!}P_{\mu \nu }$ as in (6.49),

$$\begin{aligned}&\sum _{J+K=I}\!\!\!\big |P\big (\partial _\mu \widehat{{\mathcal {L}}}_Z^J h,\partial _\nu \widehat{{\mathcal {L}}}_Z^K h\big )\big |\lesssim \sum _{|J|+|K|\le |I|\!\!\!\!\!\!\!\!\!\!\!}\big | {/\!\!\!\!}P_{\mu \nu }\big (\partial \widehat{{\mathcal {L}}}_Z^J h,\partial \widehat{{\mathcal {L}}}_Z^K h\big )\big |\\&\quad +\sum _{|J|+|K|\le |I|\!\!\!\!\!\!\!\!\!\!\!} \big (|\partial _q \widehat{{\mathcal {L}}}_Z^J{h}|_{L{{\mathcal {T}}}} +|\partial _q \,/{\!\!\!\!\text {tr}}\,\widehat{{\mathcal {L}}}_Z^J {h} |\big )\, |\partial \widehat{{\mathcal {L}}}_Z^K h|. \end{aligned}$$

Summing up, we have the estimate

6.90 $$\begin{aligned}&\big |{{\mathcal {L}}}_Z^I F_{\mu \nu }(h)(\partial h,\partial h)\big |\nonumber \\&\quad \lesssim \sum _{|J|+|K|\le |I|}\!\! \!\!\!\!\!\!\big | {/\!\!\!\!}P_{\mu \nu }\big (\partial \widehat{{\mathcal {L}}}_Z^J h, \widehat{{\mathcal {L}}}_Z^K h\big )\big | +\sum _{|J|+|K|\le |I|}\!\! |\overline{\partial } \widehat{{\mathcal {L}}}_Z^J h|\, |{\partial } \widehat{{\mathcal {L}}}_Z^K h|\nonumber \\&\qquad +\sum _{|I_1|+|I_{2}|+|I_{3}|\le |I|,\, } |\widehat{{\mathcal {L}}}_Z^{I_3} h| |\partial \widehat{{\mathcal {L}}}_Z^{I_1} h|\, |\partial \widehat{{\mathcal {L}}}_Z^{I_2} h|. \end{aligned}$$

Dividing up into low and high derivatives, we get

6.91 $$\begin{aligned}&\big |{{\mathcal {L}}}_Z^I F_{\mu \nu }(h)(\partial h,\partial h)\big |\nonumber \\&\quad \lesssim \big (|\partial h|_{\mathcal {S}S}+|\overline{\partial } h|+|h|\,|\partial h|)\!\!\!\sum _{|J|\le |I|}\!\! \!\!|\partial \widehat{{\mathcal {L}}}_Z^J h| +|\partial h|\!\!\! \sum _{|J|\le |I|}\!\! |\overline{\partial } \widehat{{\mathcal {L}}}_Z^J h|\, +|\partial h|^2\!\!\! \sum _{|J|\le |I|}\!\! |\widehat{{\mathcal {L}}}_Z^J h| \nonumber \\&\qquad +\sum _{|K|\le |I|/2\!\!\!\!\!\!\!\!\!\!\!\!\!\!\!\!\!}|\partial \widehat{{\mathcal {L}}}_Z^K h|\sum _{|J|\le |I|-1\!\!\!\!\!\!\!\!\!\!\!} |\partial \widehat{{\mathcal {L}}}_Z^J h|\nonumber \\&\quad \lesssim \frac{\varepsilon (1\!+\!q_+)^{-1}}{1+t+r}\!\! \sum _{|J|\le |I|}\!\! \!\!|\partial \widehat{{\mathcal {L}}}_Z^J h| +\frac{\varepsilon (1\!+\!t)^{2\delta }(1\!+\!q_+)^{-2\delta }\!\!\!}{(1+t+r)(1+|q|)} \!\sum _{|J|\le |I|}\!\! |\overline{\partial } \widehat{{\mathcal {L}}}_Z^J h|\nonumber \\&\qquad +\frac{\varepsilon ^2(1\!+\!t)^{4\delta }(1\!+\!q_+)^{-4\delta }\!\!\!}{(1+t+r)^2(1+|q|)^2} \!\!\!\sum _{|J|\le |I|}\!\! |\widehat{{\mathcal {L}}}_Z^J h|\nonumber \\&\qquad +\sum _{|K|\le |I|/2\!\!\!\!\!\!\!\!\!\!\!\!\!\!\!\!\!}|\partial \widehat{{\mathcal {L}}}_Z^K h|\sum _{|J|\le |I|-1\!\!\!\!\!\!\!\!\!\!\!} |\partial \widehat{{\mathcal {L}}}_Z^J h|. \end{aligned}$$

#### Estimates of the Wave Operator Applied to $h^0$.

By (6.58), we have

6.92 $$\begin{aligned} \big |{{\mathcal {L}}}_Z^I\, \big (\widetilde{\square }_g -\square _0\big )h^0_{\mu \nu }\big | \lesssim \frac{M}{(1+t+r)^3}\sum _{|J|\le |I|} \big |\widehat{{\mathcal {L}}}_Z^J H_1|, \end{aligned}$$

and by (6.57), we have

6.93 $$\begin{aligned} \big | {{\mathcal {L}}}_Z^I\, \square _0 h^0_{\mu \nu }\big | \lesssim \frac{M}{(1+t+r)^3} \chi ^\prime \big (\tfrac{r}{t+1}\big ), \end{aligned}$$

where $\chi ^\prime (s)$ is supported in $1/4\le s\le 1$.

#### Estimates of the Wave Commutator Term

By (6.52), we have

$$\begin{aligned}&\big |\big [ \widetilde{\square }_g \widehat{{\mathcal {L}}}_Z^I -{{\mathcal {L}}}_Z^I\widetilde{\square }_g\big ]\phi _{\mu \nu }\big |\lesssim \sum _{J+K=I,\,|K|<|I|\!\!\!\!\! \!\!\!\!\!\!\!\!\!\!\!\!\!\!\!\!\!\!} \big |\widehat{{\mathcal {L}}}_Z^J H^{\alpha \beta }\partial _\alpha \partial _\beta \widehat{{\mathcal {L}}}_Z^K \phi _{\mu \nu }\big | \\&\quad \lesssim \sum _{\begin{array}{c} |J|+|K|-1\le |I|,\\ 1\le |K|\le |I| \end{array}}\!\!\!\!\! \bigg (\frac{\big |(\widehat{{\mathcal {L}}}_Z^J H)_{LL}\big |}{1+|q|}+\frac{\big |\widehat{{\mathcal {L}}}_Z^J H\big |}{1+t+r}\bigg ) \,\big |\partial \widehat{{\mathcal {L}}}_Z^K \phi _{\mu \nu }\big |. \end{aligned}$$

Writing $H=H_0+H_1$, this can be divided up in the commutator with $\square _0=\square +H_0^{\alpha \beta }\partial _\alpha \partial _\beta$ and with $\widetilde{\square }_g-\square _0=H_1^{\alpha \beta }\partial _\alpha \partial _\beta$. Since $H_0\sim r^{-1}$, we have

6.94 $$\begin{aligned} \big |\big [ \square _0 {\widehat{{\mathcal {L}}}}_Z^I -{{\mathcal {L}}}_Z^I\square _0\big ]\phi _{\mu \nu }\big |\lesssim & {} \sum _{|J|\le |I|}\!\!\!\! \bigg (\frac{\big |({\widehat{{\mathcal {L}}}}_Z^J H_0)_{LL}\big |\!}{1+|q|}+\frac{\big |{\widehat{{\mathcal {L}}}}_Z^J H_0\big |}{1+t+r}\bigg ) \!\!\!\sum _{|K|\le |I|}\!\!\!\!\big |\partial {\widehat{{\mathcal {L}}}}_Z^K \phi _{\mu \nu }\big |\nonumber \\\lesssim & {} \frac{M(1\!+\!|q|)^{-1}}{1\!+t+r}\!\sum _{|K|\le |I|\!\!\!\!\!\!}\!\! \,\big |\partial \widehat{{\mathcal {L}}}_Z^K \phi _{\mu \nu }\big |. \end{aligned}$$

Similarly, by (6.87) and (6.17),

$$\begin{aligned}&\sum _{|J|\le |I|/2+1\!\!\!\!\! \!\!\!\!\!\!\!\!}\big (\frac{\big |(\widehat{{\mathcal {L}}}_Z^J H_{\!1})_{LL}\big |\!}{1+|q|}+\frac{\big |\widehat{{\mathcal {L}}}_Z^J H_{\!1}\big |}{1+t+r}\big ) \!\!\!\sum _{|K|\le |I|}\!\!\!\!\big |\partial \widehat{{\mathcal {L}}}_Z^K \phi _{\mu \nu }\big |\\&\quad \lesssim \frac{\varepsilon (1+q_+)^{-\gamma }}{(1+t+r)^{1+\gamma -2\delta }(1+|q|)^{1-\gamma }} \!\sum _{|K|\le |I|\!\!\!\!\!\!}\!\! \,\big |\partial \widehat{{\mathcal {L}}}_Z^K \phi _{\mu \nu }\big |, \end{aligned}$$

and we conclude that

6.95 $$\begin{aligned}&\big |\big [ (\widetilde{\square }_g \!-\square _0)\widehat{{\mathcal {L}}}_Z^I -{{\mathcal {L}}}_Z^I (\widetilde{\square }_g \!-\square _0) \big ]\phi _{\mu \nu }\big |\nonumber \\&\quad \lesssim \frac{\varepsilon (1+q_+)^{-\gamma }}{(1\!+\!t\!+\!r)^{1+\gamma -2\delta }(1\!+\!|q|)^{1-\gamma }} \!\sum _{|K|\le |I|\!\!\!\!\!\!}\!\! \,\big |\partial \widehat{{\mathcal {L}}}_Z^K \phi _{\mu \nu }\big |\!\nonumber \\&\qquad +\!\sum _{|K|\le |I|/2\!\!\!\!\!\!\!\!\!\!\!\!\!}\!\!\big |\partial \widehat{{\mathcal {L}}}_Z^K \phi _{\mu \nu }\big | \sum _{|J|\le |I| \!\!\!\!\!\!} \bigg (\!\frac{\big |(\widehat{{\mathcal {L}}}_Z^J H_1)_{LL}\big |\!}{1+|q|}+\frac{\big |\widehat{{\mathcal {L}}}_Z^J H_1\big |\!}{1+t}\bigg ) . \end{aligned}$$

### The Sharp $L^\infty$ Decay Estimates for Higher Order Low Derivatives

As in section 10 of [37], using the methods in Section 6.2 we can also inductively prove sharp decay estimates for higher order low derivatives. As we have already proven the higher order weak decay estimates in Proposition 6.11 and the higher order sharp decay estimates for components we control with the wave coordinate condition in Proposition 6.26, it only remains to generalize Proposition 6.23 to higher order. In order to do that we will, as before, rely on the crucial Lemma 6.21 to control transversal derivatives in terms of tangential derivatives, which we control by Proposition 6.11, and $\square _0$ close to the light cone $|t-r|<1-c$. It therefore only remains to get control of $\square _0 \widehat{{\mathcal {L}}}_Z^I h^1_{\mu \nu }$ close to the light cone $|t-r|<(1-c)t$, where $0<c<1$. When $|t-r|<(1-c)t$, that we have, by (6.57) and (6.58),

6.96 $$\begin{aligned} {{\mathcal {L}}}_Z^I \square _g h^1_{\mu \nu } ={{\mathcal {L}}}_Z^I F_{\mu \nu } -{{\mathcal {L}}}_Z^I (\square _g -\square _0)h^0_{\mu \nu },\qquad |t-r|<(1-c)t, \end{aligned}$$

where $\widehat{{\mathcal {L}}}_Z^I (\square _g -\square _0)h^0_{\mu \nu }$ is controlled by (6.92) using Proposition 6.11 as follows:

6.97 $$\begin{aligned} \big |{{\mathcal {L}}}_Z^I\, \big (\widetilde{\square }_g -\square _0\big )h^0_{\mu \nu }\big | \lesssim \frac{\varepsilon M}{(1+t+r)^{3-2\delta }(1+q_+)^{\gamma }}, \end{aligned}$$

and by (6.91) as

6.98 $$\begin{aligned}&\big |{{\mathcal {L}}}_Z^I F_{\mu \nu }(H)(\partial h,\partial h)\big |\nonumber \\&\quad \lesssim \frac{\varepsilon (1\!+\!q_+)^{-1}}{1+t+r}\!\! \!\!\sum _{|J|\le |I|}\!\! \!\!|\partial \widehat{{\mathcal {L}}}_Z^J h| +\frac{\varepsilon ^2(1\!+\!q_+)^{-4\delta }\!\!\!}{(1+t+r)^{3-4\delta }(1+|q|)}\nonumber \\&+\frac{\varepsilon ^3(1\!+\!q_+)^{-6\delta }\!\!\!}{(1+t+r)^{3-6\delta }(1+|q|)^3}\nonumber \\&\qquad +\sum _{|K|\le |I|/2\!\!\!\!\!\!\!\!\!\!\!\!\!\!\!\!\!}|\partial \widehat{{\mathcal {L}}}_Z^K h|\sum _{|J|\le |I|-1\!\!\!\!\!\!\!\!\!\!\!} |\partial \widehat{{\mathcal {L}}}_Z^J h|. \end{aligned}$$

It remains to estimate the difference $\widetilde{\square }_g-\square _0$ and the commutators

$$\begin{aligned} \square _0 \widehat{{\mathcal {L}}}_Z^I h^1_{\mu \nu }= & {} {{\mathcal {L}}}_Z^I \square _0 h^1_{\mu \nu } +[ \square _0\widehat{{\mathcal {L}}}_Z^I-{{\mathcal {L}}}_Z^I\square _0] h^1_{\mu \nu } \\= & {} {{\mathcal {L}}}_Z^I \widetilde{\square }_g h^1_{\mu \nu } -{{\mathcal {L}}}_Z^I (\widetilde{\square }_g-\square _0) h^1_{\mu \nu } +[ \square _0\widehat{{\mathcal {L}}}_Z^I-{{\mathcal {L}}}_Z^I\square _0] h^1_{\mu \nu }. \end{aligned}$$

By (6.94)

6.99 $$\begin{aligned} \big |\big [ \square _0 \widehat{{\mathcal {L}}}_Z^I- {{\mathcal {L}}}_Z^I \square _0 \big ]h^1_{\mu \nu }\big | \lesssim \frac{\varepsilon (1\!+\!|q|)^{-1}}{1\!+t+r}\!\sum _{|K|\le |I|\!\!\!\!\!\!}\!\! \,\big |\partial \widehat{{\mathcal {L}}}_Z^K h^1_{\mu \nu }\big |, \end{aligned}$$

and by,

6.100 $$\begin{aligned} \big |\big [ (\widetilde{\square }_g \!-\square _0)\widehat{{\mathcal {L}}}_Z^I -{{\mathcal {L}}}_Z^I (\widetilde{\square }_g \!-\square _0) \big ]h^1_{\mu \nu }\big |\lesssim \frac{\varepsilon (1+q_+)^{-\gamma }}{(1\!+\!t\!+\!r)^{1+\gamma -2\delta }(1\!+\!|q|)^{1-\gamma }} \!\sum _{|K|\le |I|\!\!\!\!\!\!}\!\! \,\big |\partial \widehat{{\mathcal {L}}}_Z^K h^1_{\mu \nu }\big |. \end{aligned}$$

Since ${{\mathcal {L}}}_Z^I (\widetilde{\square }_g-\square _0) h^1_{\mu \nu } -\big [ (\widetilde{\square }_g \!-\square _0)\widehat{{\mathcal {L}}}_Z^I-{{\mathcal {L}}}_Z^I (\widetilde{\square }_g \!-\square _0) \big ]h^1_{\mu \nu } = (\widetilde{\square }_g-\square _0) \widehat{{\mathcal {L}}}_Z^Ih^1_{\mu \nu }$, which can be estimated in the same way, we obtain

6.101 $$\begin{aligned} \big |{{\mathcal {L}}}_Z^I( \widetilde{\square }_g \!-\square _0)h^1_{\mu \nu }\big |\lesssim & {} \frac{\varepsilon (1+q_+)^{-\gamma }}{(1\!+\!t\!+\!r)^{1+\gamma -2\delta }(1\!+\!|q|)^{1-\gamma }} \!\sum _{|K|\le |I|+1\!\!\!\!\!\!}\!\! \,\big |\partial \widehat{{\mathcal {L}}}_Z^K h^1_{\mu \nu }\big |\nonumber \\\lesssim & {} \frac{\varepsilon ^2(1+q_+)^{-2\gamma }}{(1\!+\!t\!+\!r)^{2+\gamma -4\delta }(1\!+\!|q|)^{2-\gamma }}. \end{aligned}$$

Summing up,

6.102 $$\begin{aligned} \big | \square _0 \widehat{{\mathcal {L}}}_Z^I h^1_{\mu \nu }\big |\lesssim & {} \frac{\varepsilon (1\!+\!q_+)^{-1}}{1+t+r}\!\! \!\!\sum _{|J|\le |I|}\!\! \!\!|\partial \widehat{{\mathcal {L}}}_Z^J h| +\sum _{|K|\le |I|/2\!\!\!\!\!\!\!\!\!\!\!\!\!\!\!\!\!}|\partial \widehat{{\mathcal {L}}}_Z^K h|\sum _{|J|\le |I|-1\!\!\!\!\!\!\!\!\!\!\!} |\partial \widehat{{\mathcal {L}}}_Z^J h| \nonumber \\&+\frac{\varepsilon ^2(1\!+\!q_+)^{-4\delta }\!\!\!}{(1+t+r)^{3-4\delta }(1+|q|)} +\frac{\varepsilon ^2(1+q_+)^{-2\gamma }}{(1\!+\!t\!+\!r)^{2+\gamma -4\delta }(1\!+\!|q|)^{2-\gamma }}.\nonumber \\ \end{aligned}$$

From Lemma 6.21 and the estimate (6.17), we get

#### Lemma 6.27

Let $D_t=\{(t,x);\, \big |t-|x|\big |\le c_0 t \}$ for some constant $0<c_0<1$ and $\overline{w}(q)=(1+q_+)^{1+\gamma ^\prime }$ where $-1\le \gamma ^\prime <\gamma -2\delta$. Then

6.103 $$\begin{aligned} (1+t+|x|) \,|\partial \widehat{{\mathcal {L}}}_Z^I h^1(t,x)\, \overline{w}(q)| \lesssim \varepsilon + \int _0^t (1+\tau )\Vert \,\square _0 \widehat{{\mathcal {L}}}_Z^I h^1(\tau ,\cdot )\, \overline{w}\Vert _{L^\infty (D_\tau )} \,\mathrm{d}\tau . \end{aligned}$$

#### Proposition 6.28

If the weak energy bounds and initial bounds hold, then we have, for any $0\le \gamma ^\prime <\gamma -2\delta$ and $|I|=k\le N-5$, that there are constants $c_k$ such that

6.104 $$\begin{aligned} \big | \partial \widehat{{\mathcal {L}}}_Z^I h^1\big |\le c_k \varepsilon (1+t)^{c_k\varepsilon } (1+t+r)^{-1}(1+q_+)^{-1-\gamma ^\prime }. \end{aligned}$$

The same estimates hold for *h* in place of $h^1$ if $\gamma ^\prime =0$.

#### Proof

Let $N_k(t)=(1+t) \sum _{|I|\le k}\Vert \widehat{{\mathcal {L}}}_Z^I h^1(t,\cdot )\overline{w}\Vert _{L^\infty (D_t)}$. We will prove (6.104) by induction, noting that it is true for $k=0$ by (6.68). Then, by Lemma 6.27, we have, for $k\ge 1$,

6.105 $$\begin{aligned} N_k(t)\lesssim \varepsilon +\int _0^t \frac{\varepsilon }{1+\tau } N_k(\tau ) \,\mathrm{d}\tau +\int _0^t \frac{1}{1+\tau } N_{k-1}(\tau )^2 \,\mathrm{d}\tau , \end{aligned}$$

where the bounds (6.33) have been used. By the induction hypothesis, $N_k(\tau )^2 \lesssim \varepsilon ^2 c_{k-1}^2 , (1+\tau )^{2c_{k-1}\varepsilon }$, so for some $c_k\ge 4c_{k-1}$,

6.106 $$\begin{aligned} N_k(t)\le c_k\int _0^t \frac{\varepsilon }{1+\tau } N_k(\tau ) \,\mathrm{d}\tau +c_k\, \varepsilon (1+t)^{2c_{k-1}\varepsilon } . \end{aligned}$$

Using Grönwall’s lemma with *G* denoting the integral we get $G'(t)\le \varepsilon (1+t)^{-1}c_k\big ( G(t)+\varepsilon (1+t)^{2c_{k-1}\varepsilon }\big )$ and multiplying with the integrating factor we get $\big ( G(t) (1+t)^{-c_k\varepsilon }\big )^\prime \le c_k\varepsilon ^2 (1+t)^{2 c_{k-1}\varepsilon -c_k\varepsilon -1}$. Assuming that $c_k \ge 4c_{k-1}$, we get $G(t)\le c_k \varepsilon (1+t)^{c_k\varepsilon }$, and hence $N_k(t)\le c_k^\prime \varepsilon (1+t)^{c_k\varepsilon }$. $\quad \square$

### The Energy Estimate

#### The Basic Energy Estimate for the Wave Equation

In [37] (see Proposition 6.2 there), the following energy estimate was proven:

##### Lemma 6.29

Let $\phi$ be a solution of the wave equation $\widetilde{\square }_g \phi =F$, with the metric *g* such that, for $H^{\alpha \beta }=g^{\alpha \beta }-m^{\alpha \beta }$,

6.107 $$\begin{aligned}&(1+|q|)^{-1} |H|_{LL} +|\partial H|_{LL}+|\overline{\partial } H|\le C\varepsilon ' (1+t)^{-1},\nonumber \\&(1+|q|)^{-1}\,|H|+ |\partial H|\le C\varepsilon ' (1+t)^{-\frac{1}{2}} (1+|q|)^{-\frac{1}{2}}(1+q_-)^{-\mu } \end{aligned}$$

for some $\mu >0$. Set

$$\begin{aligned} w={\left\{ \begin{array}{ll} (1+|r-t|)^{1+2\gamma },\,\,\, &{}r>t\\ 1+(1+|r-t|)^{-2\mu },\quad &{}r\le t\end{array}\right. }\qquad \text {and}\qquad w^\prime ={\left\{ \begin{array}{ll} (1+2\gamma )(1+|r-t|)^{2\gamma },\,\,\, &{}r>t\\ 2\mu (1+|r-t|)^{-1-2\mu },\quad &{}r\le t.\end{array}\right. } \end{aligned}$$

Then, for any $0<\gamma \leqq 1$ and $0<\varepsilon '\le \gamma /C_1$, we have

6.108 $$\begin{aligned}&\int _{\Sigma _{t}}\!\! |\partial \phi |^{2}\,w \mathrm{d}x + \!\int _{0}^{t}\!\! \int _{\Sigma _{\tau }} \!\!|{\bar{\partial }}\phi |^{2}\,w^{\prime } \,\mathrm{d}x \mathrm{d}\tau \le 8\!\int _{\Sigma _{0}}\!\! |\partial \phi |^{2}\,w \,\mathrm{d}x\nonumber \\&\quad + \!\int _0^t\!\!\frac{C\varepsilon }{1+\tau }\int _{\Sigma _{\tau }} \!\! |\partial \phi |^{2} \, w \,\mathrm{d}x \, \mathrm{d}\tau \nonumber \\&\quad +16 \int _0^t\Big (\int _{\Sigma _{\tau }}\!\!\! |F|^2 w\mathrm{d}x\Big )^{\!1/2}\Big (\int _{\Sigma _{\tau }} \!\!\! |\partial \phi |^2 w \mathrm{d}x\Big )^{\!1/2}\! \,\mathrm{d}\tau . \end{aligned}$$

#### The Lowest Order Energy Estimate for Einstein’s Equations

Let

6.109 $$\begin{aligned} E_k(t)=\sum _{|I|\le k}\int _{\Sigma _t}|\partial Z^I h^1|^2 w \,\mathrm{d}x\qquad \text {and}\qquad S_k(t)=\sum _{|I|\le k}\int _0^t\int _{\Sigma _t}|\partial Z^I h^1|^2 w^\prime \, \mathrm{d}x \,\mathrm{d}\tau . \end{aligned}$$

By Lemma 6.29, we have

6.110 $$\begin{aligned} E_0(t)+S_0(t)\le 8 E_0(0)+\int _0^t \frac{\varepsilon }{1+\tau }E_0(\tau )+\Vert F(\tau ,\cdot ) w^{1/2}\Vert _{L^2}E_0(\tau )^{1/2} \,\mathrm{d}\tau , \end{aligned}$$

where $F=\big |F_{\mu \nu }(h)(\partial h,\partial h)+\widehat{T}_{\mu \nu }-\widetilde{\square }_g h^0_{\mu \nu }\big |$, where with $\overline{h}=h_{TS}$, $T,S\in \mathcal {S}$, we have

6.111 $$\begin{aligned}&|F_{\mu \nu }|\lesssim |{/\!\!\!\!}P_{\mu \nu } (\partial h,\partial h)|+|\overline{\partial } h|\, |\partial h| +|h|\, |\partial h|^2\lesssim \big (|\partial \overline{h}|+|\overline{\partial } h|+|h|\, |\partial h|\big )|\partial h| \nonumber \\&\quad \lesssim \frac{\varepsilon |\partial h|}{(1+t+r)(1+q_+)}. \end{aligned}$$

Writing $h=h^0+h^1$, we see that it is enough to estimate

6.112 $$\begin{aligned} F^j=\frac{\varepsilon |\partial h^j|}{(1+t+r)(1+q_+)} \end{aligned}$$

for $j=0,1$. We have

6.113 $$\begin{aligned} \Vert F^{1}(t,\cdot ) w^{1/2}\Vert _{L^2}\lesssim \varepsilon (1+t)^{-1}\Vert \partial h^1(t,\cdot ) w^{1/2}\Vert _{L^2}=\varepsilon (1+t)^{-1}E_0(t)^{1/2} \end{aligned}$$

and $$F^0\lesssim M\varepsilon (1+t+r)^{-3}(1+q_+)^{-1}$$, so

6.114 $$\begin{aligned} \Vert F^0(t,\cdot )w^{1/2}\Vert _{L^2} \lesssim \varepsilon M\Big (\int \frac{(1+q_+)^{2\gamma -1}}{(1+t+r)^6}\, r^2 dr\Big )^{1/2} \lesssim \frac{M\varepsilon }{(1+t)^{2-\gamma }}. \end{aligned}$$

As far as the energy estimate is concerned, one could have picked $h^0_{\mu \nu }$ to satisfy $\widetilde{\square }_g h^0_{\mu \nu }=0$ and wouldn’t have to do anything further. However, since we didn’t do this, we will estimate using (6.58) and (6.57) to set

6.115 $$\begin{aligned} \big | \widetilde{\square }_g h^0_{\mu \nu }\big |\le & {} \big |[\widetilde{\square }_g-\square _0]h^0_{\mu \nu }\big | +\big |[\square _0 -\square ]h^0_{\mu \nu }|+\big |\square h^0_{\mu \nu }|\le \frac{C_0 |H_1| M}{(1+t+r)^3}\nonumber \\&+\frac{M^2\chi ^\prime \big (\tfrac{r}{t+1}\big )}{(1+t+r)^4} +\frac{M\chi ^\prime \big (\tfrac{r}{t+1}\big )}{(1+t+r)^3}, \end{aligned}$$

and hence, using Hardy’s inequality,

6.116 $$\begin{aligned} \Vert \widetilde{\square }_g h^0_{\mu \nu }(t,\cdot ) w^{1/2}\Vert _{L^2}\le CM (1+t)^{-2} \Vert \partial H_1(t,\cdot ) w^{1/2}\Vert _{L^2}+ M_0 M(1+t)^{-3/2}, \end{aligned}$$

where $M_0$ is a universal constant.

Hence,

6.117 $$\begin{aligned} E_0(t)\le & {} 8 E_0(0) +C'\varepsilon \int _0^t \frac{E_0(\tau )}{1+\tau }\,\mathrm{d}\tau \nonumber \\&+C'\varepsilon \int _0^t\!\!\frac{ME_0(\tau )^{1/2}}{(1+t)^{2-\gamma }}\,\mathrm{d}\tau + 16M_0\int _0^t\frac{ME_0(\tau )^{1/2}}{(1+\tau )^{3/2}} \,\mathrm{d}\tau \nonumber \\&+16\int _0^t \Vert \widehat{T} (\tau ,\cdot ) \Vert _{L^{2}}E_0(\tau )^{1/2}\,\mathrm{d}\tau \end{aligned}$$

for some univeral constant $M_0$.

### Higher Order $L^2$ Energy Estimates

For this section we have to make the following smallness assumption on $\varepsilon$:

6.118 $$\begin{aligned} c_{k'}\varepsilon \le \delta , \end{aligned}$$

where $c_{k'}$ are the constants in Proposition 6.28.

#### $L^2$ Estimate of the Inhomogeneous Term

It follows from (6.91) that, with $k=|I|$ and $k^\prime =[k/2]+1$, we have

6.119 $$\begin{aligned} \big |{{\mathcal {L}}}_Z^I F_{\mu \nu }(h)(\partial h,\partial h)\big | \lesssim F_1^{k0}+F_1^{k1}+F_2^{k0}+F_2^{k1}+F_3^{k0}+F_3^{k1}+F_4^{k0}+F_4^{k1}, \end{aligned}$$

where

6.120 $$\begin{aligned} F_1^{kj}= & {} \frac{\varepsilon \!\!\!\!}{(1+t+r)(1+q_+)}\! \sum _{|J|\le k}\!\! |\partial \widehat{{\mathcal {L}}}_Z^J h^j|,\nonumber \\ F_2^{kj}= & {} \frac{c_{k^\prime } \varepsilon (1+t)^{c_{k^\prime }\varepsilon } }{ (1+t+r)(1+q_+)}\sum _{|J|\le k-1}\!\! \!\!|\partial \widehat{{\mathcal {L}}}_Z^J h^j| \end{aligned}$$

6.121 $$\begin{aligned} F_3^{kj}= & {} \frac{\varepsilon ^2(1+t)^{4\delta }(1+q_+)^{-4\delta }\!\!\!}{(1+t+r)^2(1+|q|)^2} \sum _{|J|\le |I|}\!\! |\widehat{{\mathcal {L}}}_Z^J h^j|,\nonumber \\ F_4^{kj}= & {} \frac{\varepsilon (1+t)^{2\delta }(1+q_+)^{-2\delta }\!\!\!}{(1+t+r)(1+|q|)}\sum _{|J|\le |I|}\!\! |\overline{\partial } \widehat{{\mathcal {L}}}_Z^J h^j|. \end{aligned}$$

For $i=1,2$, we have

6.122 $$\begin{aligned} \Big (\int |F_1^{k1}|^2 w\, \mathrm{d}x\Big )^{1/2}\lesssim & {} \frac{\varepsilon }{1+t} E_{k}(t)^{1/2} ,\qquad \Big (\int |F_2^{k1}|^2\, w\, \mathrm{d}x\Big )^{1/2} \nonumber \\\le & {} \frac{c_{k^\prime }\varepsilon (1+t)^{c_{k^\prime } \varepsilon } }{1+t} E_{k-1}(t)^{1/2} . \end{aligned}$$

We have

6.123 $$\begin{aligned} |F_1^{k0}|\lesssim \frac{\varepsilon M\!\!\!\!}{(1+t+r)^{3}(1+q_+)}, \qquad |F_2^{k0}|\lesssim \frac{\varepsilon M(1+t)^{c_{k^\prime }\varepsilon }}{(1+t+r)^{3}(1+q_+)}, \end{aligned}$$

and hence,

6.124 $$\begin{aligned} \Big (\int |F_1^{k0}|^2 w\, \mathrm{d}x\Big )^{1/2} \lesssim \frac{\varepsilon M}{(1+t)^{2-\gamma }} ,\qquad \Big (\int |F_2^{k0}|^2\, w\, \mathrm{d}x\Big )^{1/2} \le \frac{c_{k^\prime }\varepsilon M }{(1+t)^{2-\gamma -c_{k'}\varepsilon }} . \end{aligned}$$

For $i=3$ we will use Hardy’s inequality (Lemma 6.9), but first we divide it up into two terms for $j=0,1$:

6.125 $$\begin{aligned} |F_3^{k0}|\lesssim \frac{\varepsilon ^2 M (1+t)^{4\delta }(1+q_+)^{-4\delta }\!\!\!}{(1+t+r)^3(1+|q|)^2}, \end{aligned}$$

and hence

6.126 $$\begin{aligned} \Big (\int |F_3^{k0}|^2\, w\, \mathrm{d}x \Big )^{1/2} \lesssim \frac{\varepsilon ^2 M}{(1+t)^{2-4\delta } }. \end{aligned}$$

By Hardy’s inequality,

6.127 $$\begin{aligned} \Big (\int |F_3^{k1}|^2\, w\, \mathrm{d}x \Big )^{1/2}\lesssim & {} \sum _{|J|\le |I|}\!\! \frac{\varepsilon }{(1+t)^{2-4\delta } }\Big ( \int \frac{|\widehat{{\mathcal {L}}}_Z^J h^1|^2}{(1+|q|)^2} w\mathrm{d}x \Big )^{1/2}\nonumber \\\lesssim & {} \frac{\varepsilon }{(1+t)^{2-4\delta } } E_k(t)^{1/2}. \end{aligned}$$

Moreover,

6.128 $$\begin{aligned} |F_4^{k0}|\lesssim \frac{\varepsilon M (1+t)^{2\delta }(1+q_+)^{-2\delta }\!\!\!}{(1+t+r)^3(1+|q|)}, \end{aligned}$$

and hence,

6.129 $$\begin{aligned} \Big (\int |F_4^{k0}|^2\, w\, \mathrm{d}x \Big )^{1/2} \lesssim \frac{\varepsilon M}{(1+t)^{3-2\gamma } }. \end{aligned}$$

The last term $F_4^{k1}$ will be estimated differently in terms of the space–time integral. We have

$$\begin{aligned} \int |F_4^{k1}|^2 w\, \mathrm{d}x\lesssim & {} \int \frac{\varepsilon ^2(1+t)^{4\delta }(1+q_+)^{-4\delta }\!\!\!}{(1+t+r)^2(1+|q|)^2}\sum _{|J|\le |I|}\!\! |\overline{\partial } \widehat{{\mathcal {L}}}_Z^J h^1|^2\, w \mathrm{d}x\\\lesssim & {} \frac{\varepsilon ^2}{(1+t)^{2-4\delta }} \int \sum _{|J|\le |I|}\!\! |\overline{\partial } \widehat{{\mathcal {L}}}_Z^J h^1|^2\, w^\prime \mathrm{d}x. \end{aligned}$$

It follows that

$$\begin{aligned} \int \Big (\int |F_4^{k1}|^2 w\, \mathrm{d}x\Big )^{1/2} E_k(\tau )^{1/2} \,\mathrm{d}\tau\le & {} C \big (\varepsilon S_k(t)\big )^{1/2}\Big (\int _0^t \frac{\varepsilon E_k(\tau )\,\mathrm{d}\tau }{(1+\tau )^{2-4\delta }} \Big )^{1/2} \\\le & {} \varepsilon S_k(t) +C^2\int _0^t \frac{\varepsilon E_k(\tau )\,\mathrm{d}\tau }{(1+\tau )^{2-4\delta }} . \end{aligned}$$

Summing up and using that $\delta \le 1/4$, we have

6.130 $$\begin{aligned} \int _0^t \!\!\int \!\!\big |{{\mathcal {L}}}_Z^I F_{\mu \nu }\big | \,\big |\partial {{\mathcal {L}}}_Z^I h^1\!\big | w\, \mathrm{d}x \,\mathrm{d}\tau\le & {} C^{\prime }\!\!\int _0^t\!\!\!\Big ( \frac{\varepsilon E_{k}(\tau )^{1/2}\!\!\!\!\!}{1+\tau } + \frac{c_{k^\prime }\varepsilon (1\!+\!\tau )^{ c_{k^\prime } \varepsilon }\!\!\!\!\!\!}{1+\tau } E_{k-1}(\tau )^{1/2}\nonumber \\&+\frac{\varepsilon M}{(1+t)^{2-\gamma -c_{k'}\varepsilon }}\Big )E_k(\tau )^{1/2} \,\mathrm{d}\tau +\varepsilon S_k(t).\nonumber \\ \end{aligned}$$

#### Equivalence of Norms

The inhomogeneous terms contain factors of $\partial \widehat{{\mathcal {L}}}_Z^I h$ which we estimate by writing $h=h_0+h^1$, and estimate the $L^2$ norm factors with $h^1$ in terms of the energy of $h^1$ whereas the $L^2$ norms of $h^0$ can be estimated directly. The commutator terms will in addition contain factors of $\widehat{{\mathcal {L}}}_Z^I H$, where we can also write $H=H_0+H_1$ and estimate the factors with $H_0$ directly since it is explicit, and for the factors with $\widehat{{\mathcal {L}}}_Z^I H_1$ we first use Hardy’s inequality to estimate them in terms of $\partial \widehat{{\mathcal {L}}}_Z^I H_1$. However $H_1$ is only approximately equal to $-h^1$. We have that $H=-h+K(h)$, where $K(h)=O(h^2)$ and hence $H_1=-h^1+K(h)-h^0-H_0$. Differentiating, we see that to conclude that the norms of $H_1$ are approximately bounded by those of $h^1$ we have to estimate factors of the form $\widehat{{\mathcal {L}}}_Z^J h\,\, \partial \widehat{{\mathcal {L}}}_Z^K h$, with $|J|+|K|\le |I|$, in $L^2$ with respect to the measure *w*. Again this can be estimated by writing $h=h^0+h^1$ and estimating the factors with $h^1$ in terms of the energy (after possibly using Hardy’s inequality) and estimating the explicit factors with $h^0$ directly. The conclusion of this process is that

6.131 $$\begin{aligned} \Vert \partial \widehat{{\mathcal {L}}}_Z^I K(h)(t,\cdot )\,\, w^{1/2}\Vert _{L^2}\lesssim \varepsilon \big (M+E_k(t)^{1/2}\big ),\quad \text {if}\quad |I|\le k. \end{aligned}$$

Hence, since $h^0=-H_0$, it follows that

6.132 $$\begin{aligned} {\sum }_{|I|\le k} \Vert \partial \widehat{{\mathcal {L}}}_Z^I H_1(t,\cdot )\,\, w^{1/2}\Vert _{L^2}\lesssim \varepsilon M +E_k(t)^{1/2}, \end{aligned}$$

and similarly for the space–time integrals of tangential components.

#### $L^2$ Estimate of the Wave Operator Applied to $h^0$

By (6.92), using Hardy’s inequality we have

6.133 $$\begin{aligned} \Vert \big ({{\mathcal {L}}}_Z^I\, \big (\widetilde{\square }_g -\square _0\big )h^0\big )(t,\cdot ) w^{1/2} \Vert _{L^2} \lesssim \frac{M}{(1+t)^{2}} E_k(t)^{1/2}, \end{aligned}$$

and by (6.93) we have

6.134 $$\begin{aligned} \sum _{|I|\le k}\Vert \big ({{\mathcal {L}}}_Z^I\, \square _0 h^0\big )(t,\cdot ) w^{1/2}\Vert _{L^2}^2 \lesssim M_k^2 \frac{M^2}{(1+t)^{3}} \end{aligned}$$

for some universal constant $M_k$.

#### $L^2$ Estimates of the Wave Commutator

It remains to estimate the commutator, which, by (6.94) and (6.95), is bounded by

$$\begin{aligned} \big |\big [ \widetilde{\square }_g \widehat{{\mathcal {L}}}_Z^I -{{\mathcal {L}}}_Z^I \widetilde{\square }_g \big ]h^1_{\mu \nu }\big |\le & {} \big |\big [ \square _0 \widehat{{\mathcal {L}}}_Z^I -{{\mathcal {L}}}_Z^I\square _0\big ]h^1_{\mu \nu }\big | \\&+\big |\big [ (\widetilde{\square }_g -\square _0)\widehat{{\mathcal {L}}}_Z^I- {{\mathcal {L}}}_Z^I(\widetilde{\square }_g -\square _0)\big ]h^1_{\mu \nu }\big | \\\lesssim & {} F_5^k + F_6^k+F_7^k, \end{aligned}$$

where

6.135 $$\begin{aligned} F_5^k=\frac{\varepsilon }{1\!+t}\!\sum _{|K|\le |I|\!\!\!\!\!\!}\!\! \,\big |\partial \widehat{{\mathcal {L}}}_Z^K h^1_{\mu \nu }\big |\! \end{aligned}$$

and

6.136 $$\begin{aligned} F_6^{k}= & {} \frac{\varepsilon (1+q_+)^{-\gamma }}{(1+t+r)^{2-2\delta }} \!\! \sum _{|J|\le |I|}\!\!\!\frac{\big |\widehat{{\mathcal {L}}}_Z^J H_1\big |}{1+|q|},\nonumber \\ F_7^{k}= & {} \frac{\varepsilon (1+t)^{2\delta }(1+q_+)^{-\gamma }}{(1+t+r)(1+|q|)} \!\! \sum _{|J|\le |I|}\!\!\!\frac{\big |(\widehat{{\mathcal {L}}}_Z^J H_1)_{LL}\big |}{1+|q|}\, \chi (\tfrac{r}{t+1}), \end{aligned}$$

since

$$\begin{aligned}&\sum _{|J|\le |I|,\, |K|\le |I|/2+1\!\!\!\!\! \!\!\!\!\! } \bigg (\frac{\big |(\widehat{{\mathcal {L}}}_Z^J H_1)_{LL}\big |\!\!}{1+|q|}+\frac{\big |\widehat{{\mathcal {L}}}_Z^J H_1\big |\!}{1+t}\bigg ) \,\big |\partial \widehat{{\mathcal {L}}}_Z^K h^1_{\mu \nu }\big | \\&\quad \lesssim \frac{\varepsilon (1+t)^{2\delta }(1+q_+)^{-\gamma }\!\!\!\!}{(1+t+r)(1+|q|)} \sum _{|J|\le |I|}\!\!\!\bigg (\frac{\big |(\widehat{{\mathcal {L}}}_Z^J H_1)_{LL}\big |}{1+|q|}+\frac{\big |\widehat{{\mathcal {L}}}_Z^J H_1\big |}{1+t+r}\bigg ). \end{aligned}$$

We have

6.137 $$\begin{aligned} \Big (\int |F_5^{k}|^2\, w\, \mathrm{d}x \Big )^{1/2}\lesssim \frac{\varepsilon }{1+t } E_k(\tau )^{1/2}. \end{aligned}$$

By Hardy’s inequality,

6.138 $$\begin{aligned} \Big (\int |F_6^{k}|^2\, w\, \mathrm{d}x \Big )^{1/2} \lesssim \sum _{|J|\le |I|}\!\! \frac{\varepsilon }{(1+t)^{2-2\delta } }\Big ( \int \frac{|\widehat{{\mathcal {L}}}_Z^J h^1|^2}{(1+|q|)^2} w\mathrm{d}x \Big )^{1/2}\lesssim \frac{\varepsilon }{(1+t)^{2-2\delta } } E_k(\tau )^{1/2}. \end{aligned}$$

Dealing with the last term $F_7^{k}$ requires the following slight generalization of Hardy’s inequality Corollary 13.3 in [37]:

##### Corollary 6.30

Let $\gamma >0$ and $\mu >0$. Then, for any $-1\le a\le 1$ and any $\phi \in C^\infty _0({{\mathbb {R}}}^3)$, if, in addition, $a<2\min {(\gamma , \mu )}$, we have

6.139 $$\begin{aligned} \int \frac{|\phi |^2}{(1+|q|)^2}\,\frac{(1+|q|)^{-a} \, }{(1+t+|q|)^{1-a}} \frac{w\, \mathrm{d}x}{(1+q_-)^{2\mu }}\lesssim \int {|\partial \phi |^2}\min \, (w', \frac{w}{(1+t+|q|)^{1-a}}) \, \mathrm{d}x. \end{aligned}$$

The last term $F_7^{k}$ will be estimated differently in terms of the space–time integral. By Hardy’s inequality and (6.85), we have

6.140 $$\begin{aligned} \int |F_7^{k}|^2 w\, \mathrm{d}x\lesssim & {} \frac{\varepsilon ^2}{1+\tau } \sum _{|J|\le |I|} \int \frac{(1+q_+)^{-2\gamma }\!\!\!}{(1+\tau +r)^{1-4\delta }(1+|q|)^2}\frac{| (\widehat{{\mathcal {L}}}_Z^J H_1)_{LL}|^2}{(1+|q|)^2}\, w \,\mathrm{d}x\nonumber \\\lesssim & {} \frac{\varepsilon ^2}{1+\tau } \sum _{|J|\le |I|} \int |\partial (\widehat{{\mathcal {L}}}_Z^J H_1)_{LL}|^2\, \min {\Big ( w^\prime ,\frac{w}{(1+\tau +|q|)^{1-4\delta }}\Big )} \mathrm{d}x \nonumber \\\lesssim & {} \!\frac{\varepsilon ^2}{1\!+\!\tau }\Big ( \sum _{|J|\le |I|}\int |\overline{\partial } \widehat{{\mathcal {L}}}_Z^J H_1|^2w^\prime \mathrm{d}x\nonumber \\&+\!\!\!\!\!\!\! \!\!\sum _{|J|+|K|\le |I|} \int \!\big | \widehat{{\mathcal {L}}}_Z^{J} {h}\big |^2 \big |\partial \widehat{{\mathcal {L}}}_Z^{K} h\big |^2 \, \frac{w\, \mathrm{d}x }{(1\!+\tau \!+\!r)^{1-4\delta }} +\int _{|x|\le 3\tau \!/4} \!\!\!\!\!\! \frac{M^2 w^\prime \mathrm{d}x \, \!\!\!}{\,\,\,\,\,(1\!+\!\tau )^4}\,\Big )\nonumber \\\lesssim & {} \frac{\varepsilon ^2}{1\!+\!\tau }\!\!\sum _{|J|\le |I|}\int \! |\overline{\partial } \widehat{{\mathcal {L}}}_Z^J H_1|^2w^\prime \, \mathrm{d}x \nonumber \\&+ \frac{\varepsilon ^4}{1\!+\!\tau }\!\!\sum _{|J|\le |I|} \int \!\! \frac{(1+q_+)^{-4\delta }}{(1\!+\!\tau \!+\!r)^{3-8\delta } } \Big (\big |\partial \widehat{{\mathcal {L}}}_Z^{J} h\big |^2\! \nonumber \\&+\frac{| \widehat{{\mathcal {L}}}_Z^{J} h|^2}{(1+|q|)^2}\Big ) w\, \mathrm{d}x +\frac{\varepsilon ^2 M^2}{(1\!+\!\tau )^{3+2\mu }}. \end{aligned}$$

Here we again write $h=h^0+h^1$. We have

6.141 $$\begin{aligned} \sum _{|J|\le |I|} \int \!\! \frac{(1+q_+)^{-4\delta }}{(1\!+\!\tau \!+\!r)^{3-8\delta } } \Big (\big |\partial \widehat{{\mathcal {L}}}_Z^{J} h^0\big |^2\! +\frac{| \widehat{{\mathcal {L}}}_Z^{J} h^0|^2}{(1+|q|)^2}\Big ) w\, \mathrm{d}x \lesssim \frac{M^2}{(1+\tau )^{3-2\gamma -4\delta } }, \end{aligned}$$

and by Hardy’s inequality again,

6.142 $$\begin{aligned}&\sum _{|J|\le |I|} \int \!\! \frac{(1+q_+)^{-4\delta }}{(1\!+\!\tau )^{3-8\delta } } \Big (\big |\partial \widehat{{\mathcal {L}}}_Z^{J} h^1\big |^2\! +\frac{| \widehat{{\mathcal {L}}}_Z^{J} h^1|^2}{(1+|q|)^2}\Big ) w\, \mathrm{d}x \end{aligned}$$

6.143 $$\begin{aligned}&\lesssim \frac{1}{(1\!+\!\tau )^{3-8\delta } } \sum _{|J|\le |I|} \int \!\! \big |\partial \widehat{{\mathcal {L}}}_Z^{J} h^1\big |^2\! w\, \mathrm{d}x. \end{aligned}$$

Hence,

$$\begin{aligned} \int |F_7^{k}|^2 w\, \mathrm{d}x\lesssim & {} \frac{\varepsilon ^2}{1\!+\!\tau }\!\!\sum _{|J|\le |I|}\int \! |\overline{\partial } \widehat{{\mathcal {L}}}_Z^J h^1|^2w^\prime \, \mathrm{d}x + \frac{\varepsilon ^2}{(1\!+\!\tau )^3 } \Big ( \sum _{|J|\le |I|}\varepsilon ^2 \int \!\! \big |\partial \widehat{{\mathcal {L}}}_Z^{J} h^1\big |^2\! w\, \mathrm{d}x+M^2\Big ) \\&+\frac{\varepsilon ^4 M^2}{(1\!+\!\tau )^{4-2\gamma -4\delta }}. \end{aligned}$$

It follows that

$$\begin{aligned}&\int \Big (\int |F_7^{k}|^2 w\, \mathrm{d}x\Big )^{1/2} E_k(\tau )^{1/2} \,\mathrm{d}\tau \\&\quad \le C\big (\varepsilon S_k(t)\big )^{1/2}\Big (\int _0^t \frac{\varepsilon }{1+\tau } E_k(\tau )\,\mathrm{d}\tau \Big )^{1/2} +C\int _0^t \frac{\varepsilon \big (\varepsilon E_k(\tau )+M E_k(\tau )^{1/2})}{(1+\tau )^{3/2}} \\&\quad +\frac{\varepsilon ^2 M E_k(\tau )^{1/2}}{(1\!+\!\tau )^{2-\gamma -2\delta }}\,\mathrm{d}\tau \\&\quad \le \varepsilon S_k(t)+\int _0^t \frac{C^\prime \varepsilon }{1+\tau } E_k(\tau )\,\mathrm{d}\tau +\int _0^t \Big (\frac{C^\prime \varepsilon }{(1+\tau )^{3/2}} \\&\quad +\frac{C^\prime \varepsilon ^2 }{(1\!+\!\tau )^{2-\gamma -2\delta }}\Big )M E_k(\tau )^{1/2}\,\mathrm{d}\tau . \end{aligned}$$

Summing up, we have

$$\begin{aligned}&\int _0^t \!\!\int \!\!\big | \big [ \widetilde{\square }_g \widehat{{\mathcal {L}}}_Z^I- {{\mathcal {L}}}_Z^I\widetilde{\square }_g\big ]h^1_{\mu \nu }\big | \,\big |\partial {{\mathcal {L}}}_Z^I h^1\big | w\, \mathrm{d}x \mathrm{d}\tau \nonumber \\&\quad \le \varepsilon S_k(t)+\int _0^t \frac{C^\prime \varepsilon }{1+\tau } E_k(\tau )\,\mathrm{d}\tau +\int _0^t \Big (\frac{C^\prime \varepsilon }{(1+\tau )^{3/2}} +\frac{C^\prime \varepsilon ^2 }{(1\!+\!\tau )^{2-\gamma -2\delta }}\Big )M E_k(\tau )^{1/2}\,\mathrm{d}\tau .\nonumber \end{aligned}$$

#### Higher Order Energy $L^2$ Estimates

Here we give the proof of Theorem 6.4 using the decay estimates proven in the previous section. We will argue by induction so we assume the energy estimate is true for $k-1$ and we will prove it for *k*. Using the energy inequality Lemma 6.29, we get, from adding up the energy contributions from the inhomogeneous term (6.130), the commutator with the wave equation (6.143) and the Vlasov matter

$$\begin{aligned}&E_k(t)+S_k(t)\le 8 E_k(0)+32 \varepsilon S_k(t) +16 M_k\!\!\int _0^t \frac{M E_k(\tau )^{1/2}}{(1+\tau )^{3/2}}\,\mathrm{d}\tau \\&\quad +C^{\prime \prime }\varepsilon \!\int _0^t\!\!\! \Big ( \frac{ E_{k}(\tau )}{1+\tau }+ \frac{c_{k^\prime }(1\!+\!\tau )^{ c_{k^\prime } \varepsilon }\!\!\!}{1+\tau } E_{k-1}(\tau )\Big ) \,\mathrm{d}\tau \\&\quad +C^{\prime \prime }\int _0^t \Big (\frac{\varepsilon ^2}{(1+\tau )^{2-\gamma -\max \{2\delta ,c_{k'}\varepsilon \}}}+\frac{\varepsilon }{(1+\tau )^{3/2}}\Big )M E_k(\tau )^{1/2}\,\mathrm{d}\tau \\&\quad +16\!\int _0^t\!\!\Big (\sum _{|I|\le k\!\!\!\!}\Vert Z^I T (\tau ,\cdot ) \Vert _{L^{2}}^2\!\Big )^{\!\!1/2}\!E_k(\tau )^{1/2} \,\mathrm{d}\tau \end{aligned}$$

for some universal constant $M_k$. We now choose $\varepsilon$ so small that $32\varepsilon \le 1$ so that $S_k(t)$ in the right can be absorbed into $S_k(t)$ on the left, and so that by $c_{k'}\varepsilon \le 2\delta$ and $C^{\prime \prime } \varepsilon \le M_k$, we obtain

$$\begin{aligned} E_k(t)\le & {} 8 E_k(0) +32 M_k\!\!\int _0^t \frac{M E_k(\tau )^{1/2}}{(1+\tau )^{3/2}}\,\mathrm{d}\tau \\&+C^{\prime \prime }\varepsilon \!\int _0^t\!\!\! \Big ( \frac{ E_{k}(\tau )}{1+\tau }+ \frac{c_{k^\prime }(1\!+\!\tau )^{ c_{k^\prime } \varepsilon }\!\!\!}{1+\tau } E_{k-1}(\tau )\Big ) \,\mathrm{d}\tau \\&+C^{\prime \prime }\int _0^t \frac{\varepsilon ^2 M E_k(\tau )^{1/2}}{(1+\tau )^{2-\gamma -2\delta }} \,\mathrm{d}\tau +16\!\int _0^t\!\!\Big (\sum _{|I|\le k\!\!\!\!}\Vert Z^I T (\tau ,\cdot ) \Vert _{L^{2}}^2\!\Big )^{\!\!1/2}\!E_k(\tau )^{1/2} \,\mathrm{d}\tau . \end{aligned}$$

## The Continuity Argument and the Proof of Theorem 1.2

The proof of Theorem 1.2 is a direct consequence of Theorem 1.3, Propositions 2.1, 6.1, 6.3 and Theorem 6.4, and the following local existence theorem for the reduced Einstein–Vlasov system (the number of derivatives used in the following local existence theorem is far from sharp): for a given time *t*, define

$$\begin{aligned} \mathcal {V}_N(t) = {\sum }_{k + \ell \le N} \Vert \partial _{x}^{k} \partial _{p}^{\ell } f (t,\cdot ,\cdot ) \Vert _{L^2_x L^2_p}. \end{aligned}$$

### Theorem 7.1

(Local existence for the reduced Einstein–Vlasov system) Suppose $N\ge 11$. Given an initial time $T_0$ and initial data $(g\vert _{t=T_0}, \partial _t g \vert _{t=T_0}, f\vert _{t=T_0})$ for the reduced Einstein–Vlasov system (1.2), (1.3), (1.11) such that

$$\begin{aligned} \mathrm {supp}(f\vert _{t=T_0}) \subset \{ \vert x \vert + \vert p \vert \le B \} \end{aligned}$$

for some $B \ge 0$, and

$$\begin{aligned} \mathcal {V}_N (t) + E_N(t)^{\frac{1}{2}} < \infty , \end{aligned}$$

there exists $T_1 > T_0$ such that a solution of the reduced Einstein–Vlasov system (1.2), (1.3), (1.11) exists for all $t \in [T_0,T_1)$ and

7.1 $$\begin{aligned} \mathcal {V}_N (t) + E_N(t)^{\frac{1}{2}} + \sum _{\vert I \vert \le N} \Vert Z^I T (t, \cdot ) \Vert _{L^1} + \sum _{\vert I \vert \le N} \Vert Z^I T (t, \cdot ) \Vert _{L^2} < \infty , \quad \text {for all } t \in [T_0,T_1). \end{aligned}$$

Moreover, the norms in (7.1) are continuous in *t*.

See the work of Choquet-Bruhat [10], and also the textbook of Ringström [42], for related local existence theorems.

The proof of Theorem 1.2 proceeds as follows: let $T_*$ be the supremum of all times $T_1$ such that a solution of the reduced Einstein–Vlasov system (1.2), (1.3), (1.11) attaining the given data exists for all $t\in [0,T_1]$ and satisfies

7.2 $$\begin{aligned} E_N(t)^{\frac{1}{2}} \le C_N \varepsilon (1+t)^{\delta }, \qquad \sum _{\vert I \vert \le N-1} \Vert Z^I \widehat{T}(t,\cdot ) \Vert _{L^1} \le C_N \varepsilon \end{aligned}$$

for all $t\in [0,T_1]$, where $\delta >0$ is such that $\delta< \gamma < 1-8\delta$ and $C_N$ is a fixed large constant, to be determined, depending only on *N*, $\delta$ and $\mathrm {supp}(f_0)$. Recall that $0<\gamma <1$ is fixed in the statement of Theorem 1.2. Recall that $\widehat{T}^{\mu \nu } = T^{\mu \nu } - \frac{1}{2} \text {tr}T g^{\mu \nu }$. Clearly the set of such $T_1$ is non–empty, by Theorem 7.1, and so $T_* > 0$. Suppose $T_*<\infty$.

By Proposition 6.1 the pointwise bounds

7.3 $$\begin{aligned} | h(t,x)|\le & {} \frac{C_N' \varepsilon (1+t)^{2\delta }}{(1+t+r)(1+q_+)^{2\delta }}, \qquad \sum _{\vert I \vert + \vert J \vert \le N-4} |\partial ^I Z^J \partial h(t,x)|\nonumber \\\le & {} \frac{C_N' \varepsilon (1+t)^{2\delta }}{(1+t+r) (1+\vert q \vert ) (1+q_+)^{2\delta }} \end{aligned}$$

hold for some constant $C_N'$ depending only on $C_N$ and on *N*. In particular, the assumptions of Proposition 2.1 are satisfied, and so, provided $\varepsilon$ is sufficiently small,

$$\begin{aligned} \mathrm {supp}( \widehat{T}^{\mu \nu }) \subset \{ (t,x) \mid \vert x \vert \le c t + K\} \end{aligned}$$

for some $$0<c<1$$, $$K \ge 0$$.

The assumptions of Proposition 6.3 are now satisfied, and so Proposition 6.3 and Theorem 6.4 imply that, for $\varepsilon <\varepsilon _N$, we have

7.4 $$\begin{aligned} Q_k(t)\le & {} 8 Q_k(0) + M_k M + C^{\prime \prime \prime }_N\varepsilon \!\int _0^t \frac{Q_{k}(\tau )}{1+\tau } + \frac{Q_{k-1}(\tau )}{(1+\tau )^{1-d_k\varepsilon }} \,\mathrm{d}\tau \nonumber \\&+ M_k \sum _{|I|\le k} \int _0^t \Vert Z^I \widehat{T} (\tau ,\cdot ) \Vert _{L^{2}} \,\mathrm{d}\tau \end{aligned}$$

for each $k=0,1,\ldots ,N$ and for all $t\in [0,T_*]$, where $Q_{-1} \equiv 0$. Here $\varepsilon _N$, $C_N'''$, $d_1,\ldots ,d_N$ are constants which depend only on $C_N^\prime$, on *N* and on *c* and *K* and a lower positive bound for $\min {\{\gamma ,1-\gamma \}}$, whereas $M_0,\ldots ,M_N$ are universal constants (which in particular do not depend on $C_N$).

The pointwise bounds (7.3) in particular imply that

$$\begin{aligned} \sum _{\vert I \vert \le N-4} |Z^I \Gamma (t,x)| \le \frac{C_N' \varepsilon }{(1+t)^{1+a}} \end{aligned}$$

for $t\in [0,T_*]$ and $\vert x \vert \le c t + K$, with $a = 2-2\delta$. The assumptions of Theorem 1.3 are therefore satisfied so that

$$\begin{aligned} \sum _{\vert I \vert \le k} \Vert Z^I T(t,\cdot ) \Vert _{L^2} \lesssim&\frac{\mathcal {V}_{k}(1 + D_N \varepsilon )}{(1+t)^{\frac{3}{2}}} + D_N \mathbb {D}_{\left\lfloor \frac{k}{2} \right\rfloor +1} \left( \frac{E_{k-1}(t)^{\frac{1}{2}}}{(1+t)^{1+a}} \right. \\&\left. + \frac{1}{(1+t)^{\frac{3}{2}}} \int _{0}^t \frac{E_{k}(s)^{\frac{1}{2}}}{(1+s)^{\frac{1}{2}}} \mathrm{d}s \right) \\ \lesssim&\frac{\mathcal {V}_{k}(1 + D_N \varepsilon )}{(1+t)^{\frac{3}{2}}} + D_N \mathbb {D}_{\left\lfloor \frac{k}{2} \right\rfloor +1} \frac{Q_{k}(t)}{(1+t)}, \end{aligned}$$

for all $k=0,1,\ldots ,N$ and for all $t\in [0,T_*]$, where the constant $D_N$ depends on $C_N$.

Now the $L^1$ bounds (7.2) and the Sobolev inequality and Lemma 6.12 imply that

$$\begin{aligned} \sum _{\vert I \vert \le N-4} \Vert Z^I \widehat{T}(t,\cdot ) \Vert _{L^{\infty }} \le \frac{C_N \varepsilon }{(1+t)^{3}}, \end{aligned}$$

and so,

$$\begin{aligned} \sum _{\vert I \vert \le k} \Vert Z^I \widehat{T} (t,\cdot ) \Vert _{L^2}\lesssim & {} \Big ( 1 + \sum _{\vert J \vert \le \left\lfloor \frac{k}{2} \right\rfloor +1} \Vert \psi Z^J h^1 (t,\cdot ) \Vert _{L^{\infty }} \Big ) \sum _{\vert I \vert \le k} \Vert Z^I T (t,\cdot ) \Vert _{L^2}\\&+ \sum _{\vert J \vert \le \left\lfloor \frac{k}{2} \right\rfloor } \Vert Z^J T (t,\cdot ) \Vert _{L^{\infty }} \sum _{1\le \vert I \vert \le k} \Vert \psi Z^I h^1 (t,\cdot ) \Vert _{L^2}, \end{aligned}$$

where $\psi (t,x)$ is the indicator function of the set $\{ \vert x \vert \le c t + K\}$. Since $\sum \Vert Z^J T (t,\cdot ) \Vert _{L^{\infty }} \le 2\sum \Vert Z^J \widehat{T} (t,\cdot ) \Vert _{L^{\infty }}$, provided $\varepsilon$ is sufficiently small, it therefore follows that

$$\begin{aligned} \sum _{\vert I \vert \le k }\Vert Z^I \widehat{T} (t,\cdot ) \Vert _{L^2} \le \frac{\mathcal {V}_{k}(C + D_N \varepsilon )}{(1+t)^{\frac{3}{2}}} + D_N \mathbb {D}_{\left\lfloor \frac{k}{2} \right\rfloor +1} \frac{Q_{k}(t)}{(1+t)} + D_N \varepsilon \frac{Q_{k}(t)}{(1+t)} \end{aligned}$$

for $k=0,1,\ldots ,N$, where the constant *C* is independent of $C_N$ and the constant $D_N$ depends on $C_N$. Inserting into (7.4) and using the fact that

$$\begin{aligned} Q_N(0) + \mathbb {D}_{\left\lfloor N/2 \right\rfloor +1} + \mathcal {V}_N + M < \varepsilon , \end{aligned}$$

and making $M_k$ and $C_N'''$ larger if necessary gives

7.5 $$\begin{aligned} Q_k(t) \le M_k \varepsilon + C_N''' \varepsilon \int _0^t \frac{Q_{k}(\tau )}{1+\tau } + \frac{Q_{k-1}(\tau )}{(1+\tau )^{1-d_k\varepsilon }} \,\mathrm{d}\tau \end{aligned}$$

for $k=0,1,\ldots ,N$. It follows from an inductive argument that the bound (7.5) implies that

7.6 $$\begin{aligned} Q_k(t) \le (M_0 + M_1 + \cdots + M_k) \varepsilon (1+t)^{(d_1+\cdots +d_k + (k+1)C_N''')\varepsilon } \end{aligned}$$

for all $t\in [0,T_*]$ and $k=0,1,\ldots ,N$, using the following form of the Grönwall inequality:

### Lemma 7.2

For $t\!>\!0$ and continuous functions $v,a,b: [0 ,t]\! \rightarrow \mathbb {R}$ such that $a\!\ge \!0$ and *b* is non-decreasing, if

$$\begin{aligned} v(s) \le \int _0^s a(s') v(s') \mathrm{d}s' + b(s) \end{aligned}$$

for $$s\in [0,t]$$, then

$$\begin{aligned} v(s) \le b(s) e^{\int _0^{s} a(s') \mathrm{d}s'}. \end{aligned}$$

Indeed, recall that $Q_{-1} \equiv 0$, and so, from the bound (7.5) with $k=0$, it follows from the Lemma 7.2 with $a(s) = C_N''' \varepsilon (1+s)^{-1}$ and $b(s) = M_0 \varepsilon$ that

$$\begin{aligned} Q_0(t) \le M_0 \varepsilon (1+t)^{C_N'''\varepsilon }. \end{aligned}$$

Now suppose that (7.6) holds for some $0 \le k \le N-1$. Then, since

$$\begin{aligned}&C_N''' \varepsilon \int _0^t \frac{Q_k (\tau )}{(1+\tau )^{1-d_{k+1} \varepsilon }}\,\mathrm{d}\tau \le C_N''' \varepsilon ^2 (M_0 + \cdots + M_k)\\&\qquad \int _0^t (1+\tau )^{(d_1+\cdots +d_{k+1} + (k+1)C_N''')\varepsilon - 1}\,\mathrm{d}\tau \\&\quad \le \frac{C_N''' \varepsilon ^2 (M_0 + \cdots + M_k) (1+t)^{(d_1+\cdots +d_{k+1} + (k+1)C_N''')\varepsilon } }{(d_1+\cdots +d_{k+1} + (k+1)C_N''')\varepsilon } \\&\quad \le (M_0 + \cdots + M_k)\varepsilon (1+t)^{(d_1+\cdots +d_{k+1} + (k+1)C_N''')\varepsilon }, \end{aligned}$$

it follows from (7.5) and Lemma 7.2 with $a(s) = C_N''' \varepsilon (1+s)^{-1}$ and $b(s) = M_{k+1} \varepsilon + (M_0 + \cdots + M_k)\varepsilon (1+s)^{(d_1+\cdots +d_{k+1} + (k_1)C_N''')\varepsilon }$ that

$$\begin{aligned} Q_{k+1}(t)\le & {} \left( M_{k+1} \varepsilon + (M_0 + \cdots + M_k)\varepsilon (1+t)^{(d_1+\cdots +d_{k+1} + (k+1)C_N''')\varepsilon } \right) (1+t)^{C_N''' \varepsilon }\\\le & {} (M_0 + \cdots + M_{k+1})\varepsilon (1+t)^{(d_1+\cdots +d_{k+1} + (k+2)C_N''')\varepsilon }. \end{aligned}$$

Moreover Theorem 1.3 implies that

$$\begin{aligned} \sum _{\vert I \vert \le N-1} \Vert Z^I \widehat{T}(t,\cdot ) \Vert _{L^1}&\lesssim \varepsilon (C + \varepsilon D_N) \\&\quad + \varepsilon D_N \frac{Q_{N-2}(t)}{(1+t)^{\frac{1}{2} - 2 \delta }} + \varepsilon D_N \int _0^t \frac{Q_{N}(s)}{(1+t)^{\frac{3}{2} - 2 \delta }} \mathrm{d}s , \end{aligned}$$

where the constant *C* is independent of $C_N$ and the constant $D_N$ depends on $C_N$. Inserting the above bound for $Q_N$ then gives

$$\begin{aligned} \sum _{\vert I \vert \le N-1} \Vert Z^I \widehat{T}(t,\cdot ) \Vert _{L^1} \lesssim C \varepsilon + D_N \varepsilon ^2, \end{aligned}$$

provided $\varepsilon$ is sufficiently small, for some new *C*, $D_N$, as above. Now, as above,

$$\begin{aligned} \sum _{\vert I \vert \le N-1} \Vert Z^I \widehat{T} (t,\cdot ) \Vert _{L^1}\lesssim & {} \Big ( 1 + \sum _{\vert J \vert \le \left\lfloor \frac{N}{2} \right\rfloor +1} \Vert \psi Z^J h^1 (t,\cdot ) \Vert _{L^{\infty }} \Big ) \sum _{\vert I \vert \le N-1} \Vert Z^I T (t,\cdot ) \Vert _{L^1}\\&+ \sum _{\vert J \vert \le \left\lfloor \frac{N}{2} \right\rfloor } \Vert Z^J T (t,\cdot ) \Vert _{L^{\infty }} \sum _{1\le \vert I \vert \le N-1} \Vert \psi Z^I h^1 (t,\cdot ) \Vert _{L^1}, \end{aligned}$$

and, since

$$\begin{aligned} \sum _{1\le \vert I \vert \le N-1} \Vert \psi Z^I h^1 (t,\cdot ) \Vert _{L^1}\le & {} \Vert \psi ^{\frac{1}{2}} \Vert _{L^2} \sum _{1\le \vert I \vert \le N-1} \Vert \psi ^{\frac{1}{2}} Z^I h^1 (t,\cdot ) \Vert _{L^2} \\\le & {} (1+t)^{\frac{3}{2}} \frac{1}{1+t} E_{\vert I \vert }(t)^{\frac{1}{2}}, \end{aligned}$$

where the equality (2.12) was used, it follows that

$$\begin{aligned} \sum _{\vert I \vert \le N-1} \Vert Z^I \widehat{T} (t,\cdot ) \Vert _{L^1} \le (C + \varepsilon C_N''') (C \varepsilon + D_N \varepsilon ^2) + \frac{C + \varepsilon C_N}{(1+t)^3} (1+t)^{\frac{1}{2}} Q_N(t) , \end{aligned}$$

and so,

7.7 $$\begin{aligned} \sum _{\vert I \vert \le N-1} \Vert Z^I \widehat{T} (t,\cdot ) \Vert _{L^1} \le C' \varepsilon + D_N' \varepsilon ^2, \end{aligned}$$

where $D_N'$ depends on $C_N$ and $C'$ does not.

It follows from the bound (7.6) with $k=N$ and the bound (7.7), provided the constant $C_N$ is chosen so that $C_N \ge \max \{ 2(M_0+\cdots +M_N), 4C \}$ and $\varepsilon$ is chosen so that $\varepsilon < \min \{ \frac{\delta }{2} (d_1+\cdots + d_N + (N+1)C_N''')^{-1}, \frac{C_N}{4D_N'} \}$, that the bounds

$$\begin{aligned} E_N(t)^{\frac{1}{2}} \le \frac{C_N}{2} \varepsilon (1+t)^{\frac{\delta }{2}}, \qquad \sum _{\vert I \vert \le N-1} \Vert Z^I \widehat{T}(t,\cdot ) \Vert _{L^1} \le \frac{C_N}{2} \varepsilon \end{aligned}$$

hold for all $t \in [0,T_*]$. Appealing once again to the local existence theorem (Theorem 7.1), now with $T_0=T_*$, this contradicts the maximality of $T_*$, and hence the solution exists and the estimates hold for all $t\in [0,\infty )$.

## Footnotes

1 See Section 1.4 below for a discussion of the more familiar null condition in the context of wave equations.

2 In fact, due to a regularity issue arising from the use of the double null gauge, the Vlasov equation is not used directly in [46]; instead certain Jacobi fields on the mass shell, endowed with the induced Sasaki metric, are controlled, and it is in the Jacobi equation that the null structure is exploited.

3 Note that the Einstein equations (1.1) are equivalent to $Ric(g)_{\alpha \beta }=T_{\alpha \beta }- \frac{1}{2}g_{\alpha \beta }{\text {tr}}_g T$.

4 In fact in Sections 4 and 5 a new translated time coordinate ${\tilde{t}} = t + t_0$ is introduced, which has the property that $\{ {\tilde{t}} = t_0 \} = \{ t = 0\}$. It is more convenient to use the vector fields defined with respect to ${\tilde{t}}$ than the vector fields defined with respect to *t*. See Section 2.2 for details.

5 Such a parameterisation of $\mathcal {P}$ is not actually used in the proof of Theorem 1.3 but, for the spacetimes of the theorem, the fact that this parameterisation exists can be shown using the bound (5.11).

6 The proof in [46] is based on a double null foliation, and an associated double null coordinate system $(u,v,\theta ^1,\theta ^2)$, of the spacetimes which are constructed, and so the language used there is slightly different. In the coordinate system $(u,v,\theta ^1,\theta ^2,p^{\theta ^1},p^{\theta ^2},p^v)$ conjugate to the double null coordinate system for $\mathcal {M}$, the vector fields $\partial _{\theta ^A}$, for $A=1,2$, are used. Defining appropriate Cartesian coordinates, one can show that $\partial _{\theta ^A}$ take the form of $\overline{\Omega }_{ij}^M$. The proof in [46] in fact reduces to a semi global problem since the matter is shown, as part of the bootstrap argument in the proof, to be supported in a strip of finite retarded *u* length. The vector $(v-u) \partial _v$ is also used which, since *u* remains of size 1 in the support of the matter, agrees to leading order with the vector field $u\partial _u + v\partial _v$ which, when written with respect to an appropriate Cartesian coordinate system, is seen to be equal to $\overline{S}^M$.

7 It should be noted that there are two contributions to this slow interior decay. The first arises from the failure of the Einstein equations in the harmonic gauge to satisfy the classical null condition of [26]. Indeed, it was recently shown by Lindblad [34] that small data solutions of the vacuum Einstein equations in the harmonic gauge satisfy this decay rate (compare with [14] where the Ricci coefficients associated to the maximal–null foliation decay in the interior at a faster rate). The second contribution arises from the presence of the Vlasov matter, in the form of the energy momentum tensor as a source term in the Einstein equations. This fact can be more easily seen in a simplified setting. Indeed, if *T*(*t*, *x*) denotes a function which decays at rate $t^{-3}$ for $\vert x \vert \le ct +K$ and vanishes for $\vert x \vert \ge c t +K$—the sharp behaviour of the components of the energy momentum tensor associated to solutions of the Vlasov equation on Minkowski space—the sharp interior behaviour of solutions of $\square \phi = T$ is $\vert \,\partial \phi (t,x) \vert \lesssim t^{-2}$, for $\vert x \vert \le c t + K.$

8 The notation $X_1$ is later used to denote a cruder approximation to the geodesics.

9 For the boosts, $\overline{B}_i$, it is slightly more convenient to allow the functions $\mathring{B}_i^l$ to depend on ${\,\widehat{\!p\!}\,\,}$. This dependence is in such a way, however, so that $\partial _{{\,\widehat{\!p\!}\,\,}^l} \mathring{B}_i^l$ involves only one derivative of $\Gamma ^{\alpha }_{\beta \gamma }$.

10 We don’t actually use this fact but it follows from (5.11).
